# Genomic reprograming analysis of the Mesothelial to Mesenchymal Transition identifies biomarkers in peritoneal dialysis patients

**DOI:** 10.1038/srep44941

**Published:** 2017-03-22

**Authors:** Vicente Ruiz-Carpio, Pilar Sandoval, Abelardo Aguilera, Patricia Albar-Vizcaíno, María Luisa Perez-Lozano, Guadalupe T. González-Mateo, Adrián Acuña-Ruiz, Jesús García-Cantalejo, Pedro Botías, María Auxiliadora Bajo, Rafael Selgas, José Antonio Sánchez-Tomero, Jutta Passlick-Deetjen, Dorothea Piecha, Janine Büchel, Sonja Steppan, Manuel López-Cabrera

**Affiliations:** 1 Departamento de Biología Celular e Inmunología, Centro de Biología Molecular “Severo Ochoa”, CSIC-UAM, Cantoblanco, Madrid, Spain; 2 Unidad de Biología Molecular y Servicio de Nefrología, Hospital Universitario de La Princesa, Instituto de Investigación Sanitaria Princesa (IP), Madrid, Spain; 3 Unidad de Genómica, CAI de Genómica y Proteómica, Universidad Complutense de Madrid, Madrid, Spain; 4 Servicio de Nefrología, Hospital Universitario La Paz, Instituto de Investigación Sanitaria La Paz (IdiPAZ), Madrid, Spain; 5 Fresenius Medical Care Deutschland GmbH, Else-Kröner-Straβe 1, 61352 Bad Homburg, Germany

## Abstract

Peritoneal dialysis (PD) is an effective renal replacement therapy, but a significant proportion of patients suffer PD-related complications, which limit the treatment duration. Mesothelial-to-mesenchymal transition (MMT) contributes to the PD-related peritoneal dysfunction. We analyzed the genetic reprograming of MMT to identify new biomarkers that may be tested in PD-patients. Microarray analysis revealed a partial overlapping between MMT induced *in vitro* and *ex vivo* in effluent-derived mesothelial cells, and that MMT is mainly a repression process being higher the number of genes that are down-regulated than those that are induced. Cellular morphology and number of altered genes showed that MMT *ex vivo* could be subdivided into two stages: early/epithelioid and advanced/non-epithelioid. RT-PCR array analysis demonstrated that a number of genes differentially expressed in effluent-derived non-epithelioid cells also showed significant differential expression when comparing standard versus low-GDP PD fluids. Thrombospondin-1 (TSP1), collagen-13 (COL13), vascular endothelial growth factor A (VEGFA), and gremlin-1 (GREM1) were measured in PD effluents, and except GREM1, showed significant differences between early and advanced stages of MMT, and their expression was associated with a high peritoneal transport status. The results establish a proof of concept about the feasibility of measuring MMT-associated secreted protein levels as potential biomarkers in PD.

Peritoneal dialysis (PD) is an effective and cost-efficient alternative to hemodialysis in the treatment of end stage renal disease^1^, covering approximately 10 to 15% of the dialysis population^2^. During PD, the peritoneal membrane functions as a semipermeable membrane across which ultrafiltration and diffusion take place^3^. However, the long-term use of PD is still limited. Nearly 50% of patients are forced to switch to hemodialysis within four to five years of PD treatment, as prolonged exposure to non-physiological PD fluids, with high concentrations of glucose and glucose degradations products (GDPs) and low pH, and peritonitis episodes induce degenerative changes of the peritoneal tissue that ultimately lead to ultrafiltration failure^2^,^4^,^5^,^6^. Membrane failure is associated with structural alterations of the peritoneum including chronic inflammation, submesothelial fibrosis and angiogenesis^7^,^8^. In consequence, one of the most important goals in PD is the preservation of the peritoneal membrane integrity^9^,^10^. The use of solutions with neutral pH and with low content of GDPs may represent a potential strategy to attenuate some of the PD-related adverse effects^11^. The impact of these novels, more biocompatible, solutions on the clinical outcomes is currently being recognized^12^,^13^,^14^,^15^,^16^. One possibility to reduce the adverse effects of both classical and novel PD fluids on the peritoneum is by decreasing the dwell time of the dialysate^17^,^18^. Another alternative approach to preserve the peritoneal membrane could be the use of pharmacological agents protecting the mesothelium or targeting inflammation and fibrosis^7^,^19^.

The structure of the PM is composed of a single layer of highly specialized mesothelial cells (MCs) that lines a compact zone of connective tissue containing few fibroblasts, mast cells, macrophages and vessels^20^. During long-term PD, MCs undergo a progressive loss of epithelial characteristics and acquire a myofibroblast-like phenotype^21^. The myofibroblast conversion of the MCs could be observed both in peritoneal tissue biopsies and in *ex vivo* cultures of MCs obtained from the dialysates. Effluent-derived MCs can be easily isolated from PD-patients using standard methods^21^,^22^. It was described that *ex vivo* cultures of effluent MCs showed two main morphologies: epithelioid and non-epithelioid (fibroblast-like)^22^. These morphological changes as well as the down-regulation of epithelial molecules (cytokeratin and E-cadherin) and the induction of mesenchymal markers (snail, N-cadherin, fibronectin, collagen I, α-smooth-muscle actin (α-SMA), and fibroblast specific protein-1 (FSP-1)) were described to be indicative of a mesenchymal conversion^7^,^21^,^22^. This so called mesothelial-to-mesenchymal transition (MMT), a special type of epithelial to mesenchymal transition (EMT), is a complex and stepwise process that is characterized by the loss of apical-basolateral polarity, disruption of intercellular junctions and acquisition of migratory and invasive properties. MCs that undergo MMT acquire the ability to produce extracellular matrix components, as well as inflammatory, fibrogenic and angiogenic factors^7^,^23^,^24^.

Despite considerable improvements in overall PD technique survival, there is a substantial unmet medical need to identify patients on PD that are at highest risk for membrane failure and to guide these patients to personalized PD regime, peritoneal resting, to hemodialysis or to early transplantation. Previously, several effluent biomarkers, such as CA125 (believed to represent MC mass) and IL-6 (indicating local inflammation), have already been shown to be informative in the follow-up of PD patients at the population level^25^,^26^. An alternative individualized approach for the discovery of new biomarkers monitoring peritoneal remodeling is the *ex vivo* study of effluent-derived MCs. Interestingly, peritoneal transport of the PD patients from whom the cells were harvested was associated with the *ex vivo* expression levels of molecules that were over-expressed during MMT in cultured effluent MC^24^,^27^. As a proof of concept, levels of MMT-associated molecules in the PD effluent, including VEGF, CTGF/CCN2, Gremlin-1 (GREM1), and MMP9 were found to correlate with the peritoneal transport status^28^,^29^,^30^,^31^,^32^. Herein, by using whole genome microarray analysis of MCs undergoing MMT *in vitro* and *ex vivo*, we aimed to identify novel molecular biomarkers that may facilitate diagnosis of the integrity and functionality of the patient’s peritoneum and hence allow early adaption of treatment to the patient’s needs.

## Results

### Analysis of gene expression profiles during *in vitro* and *ex vivo* MMT

**Figure 1:**
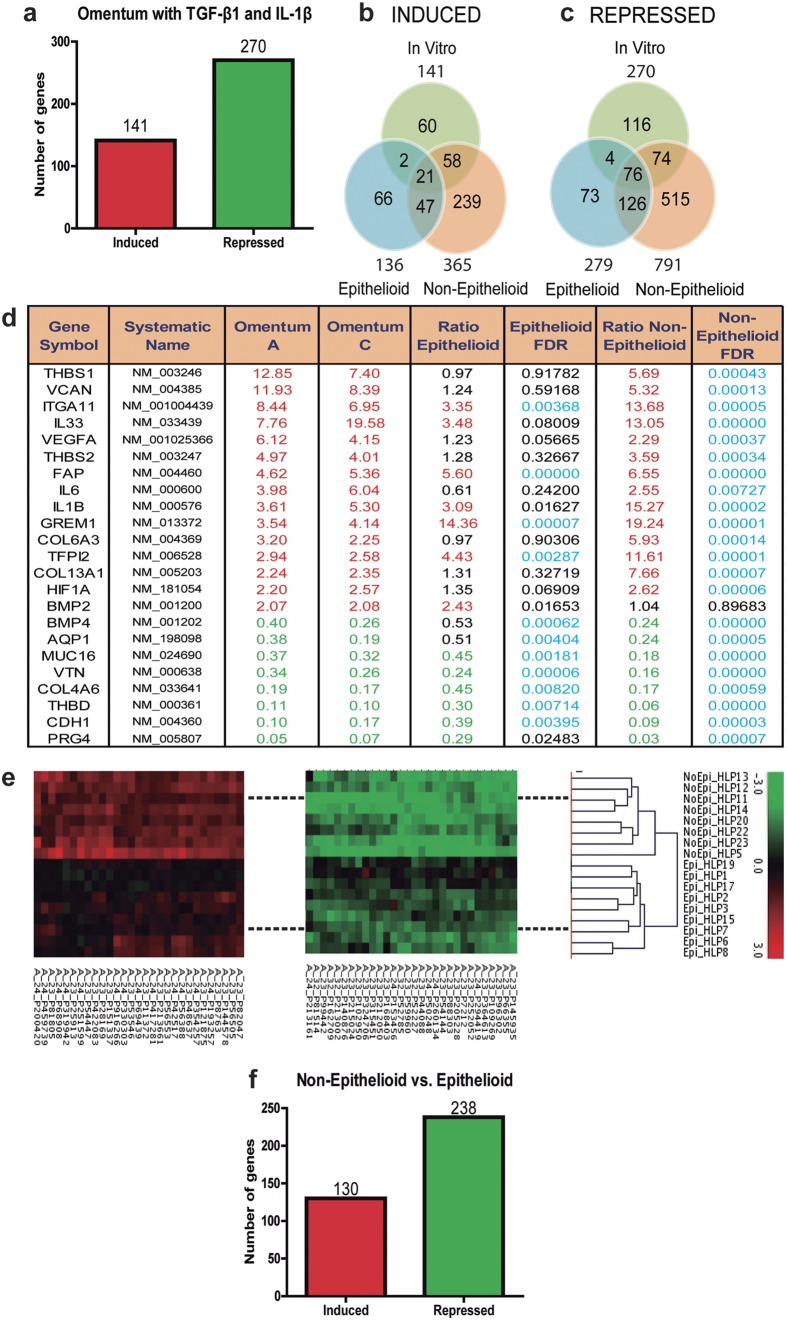
Microarray analysis. (**a**) Number of repressed genes is higher than induced genes in omentum-derived MCs treated with TGF-β1 and IL-1β. Untreated omentum-derived MCs were used as control. (**b**) Induced and (**c**) repressed genes in effluent-derived MCs indicate that repressed genes are higher than induced and the differential gene expression is greater in Non-Epithelioid phenotype compared to Epithelioid and partial overlapping was observed between *ex vivo* and *in vitro* models. (**d**) Overlapping genes in *in vitro* and *ex vivo* models. MCs from Omentum A and C were treated with TGF-β1 and IL-1β and compared with the untreated omentum-derived MCs. Ratios in Non-Epithelioid and Epithelioid depict the expression change in effluent-derived cells, with a respective FDR associated. In red: induced genes; in green: repressed genes; and in blue: statistically significant results. (**e**) Gene fingerprint of effluent-derived MCs grouped correctly the samples using cell phenotype by cluster analysis. (**f**) Number of repressed genes is higher than induce genes when Non-Epithelioid phenotype was compared with Epithelioid phenotype.

To screen novel biomarkers associated with peritoneal membrane failure, we analyzed the changes of gene expression profile along the MMT process by microarray analysis. It was previously described that the treatment of primary cultures of MCs with TGF-β1, alone or in combinations with proinflammatory cytokines such as IL-1β or TNF-α^21^,^33^,^34^, induced a myofibroblast conversion reminiscent of a MMT process. Thus, we first analyzed the gene expression changes in omentum-derived MCs induced with TGF-β1 plus IL-1β for 72 hours, and the expression fingerprint of the MMT *in vitro* was established comprising 141 induced and 270 repressed genes (Fig. 1a, Suppl. Tables S1 and S2).

We next compared the gene expression profiles of omentum-derived MCs and 17 samples of effluent-derived MCs, subdivided into epithelioid (E, n = 9) or non-epithelioid (NE, n = 8) phenotypes. A pull of RNAs obtained from four omental specimens was used as the reference control. The global microarray analysis showed a progressive reprogramming along the *ex vivo* MMT process, being higher the number of changed genes in cells with NE phenotype (induced genes: 136 in E vs 365 in NE; repressed genes: 279 in E vs 791 in NE). Of these altered genes, 68 and 202 were induced or repressed in the two groups (Fig. 1b and c, see list of genes in Suppl. Tables S3–S6). Interestingly, the fingerprint of the MMT *in vitro* overlapped only partially with the expression profiles of MMT *ex vivo* (Fig. 1b–d) suggesting that MMT *in vitro* is a similar but not identical process as MMT *ex vivo*. However, this partial overlap may be considered a cross-validation of genes that change their expression during MMT independently of the stimuli triggering this process. It is worth noting, that the genetic reprogramming during MMT, *in vitro* and *ex vivo*, is predominantly based on a gene repression. Expression data for each gene within each sample was used to create a heatmap for cluster classification, which revealed a clear separation between E and NE phenotypes (Fig. 1e), indicating that the subdivision of effluent MCs according to their elliptical factor was properly done. It was previously shown that high peritoneal transport was associated with the presence of MCs with NE phenotype in the PD effluent and with up-regulated expression *ex vivo* of MMT-related molecules^24^,^27^. In this context, this microarray analysis identified a number of genes (130 induced and 238 repressed) that were differentially regulated in NE cells when compared with E cells (Fig. 1f, Suppl. Tables S7 and S8), which opens new opportunities for the identification of biomarkers in PD.

**Figure 2:**
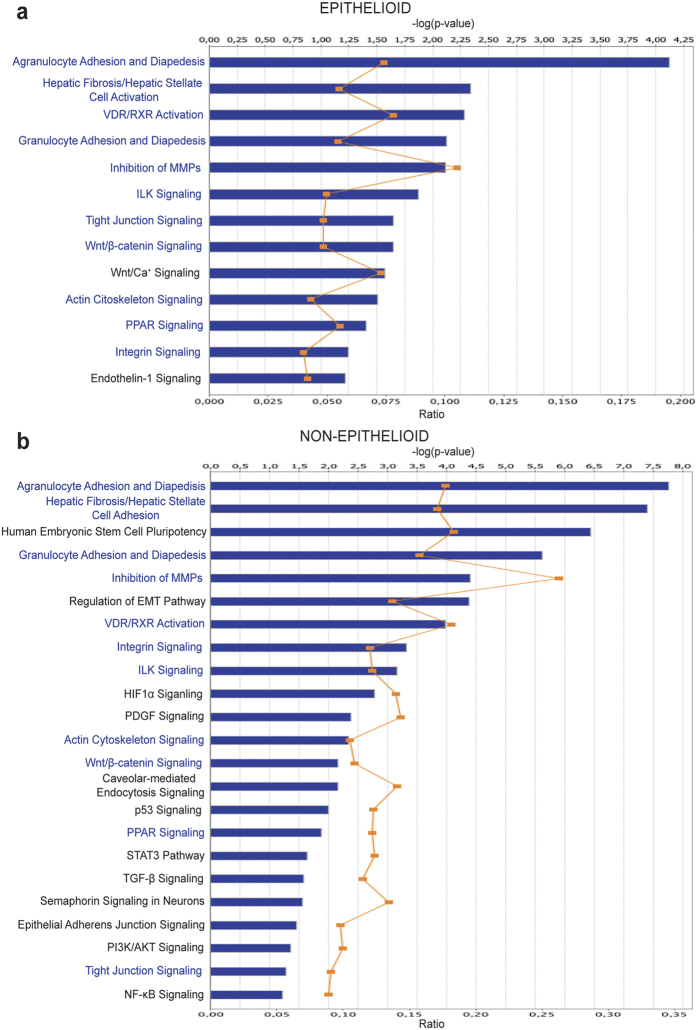
Functional analysis of relevant pathways in (**a**) Epithelioid and (**b**) Non-Epithelioid phenotypes. Although many pathways are common in both phenotypes, others are dependent of Epithelioid or Non-Epithelioid state, indicating a differential expression in pathways during MMT process. Blue: common pathways. Bold: pathways exclusive of Epithelioid or Non-Epithelioid phenotype. Ratio is the number of molecules in a given pathway that meet cutoff criteria, divided by total number of molecules that make up that pathway and that are in the reference set. Selected genes for further analysis in this work are implicated in these pathways (Suppl. Tables S9 and S10).

Agilent ID of differentially expressed genes in effluent cells with E and NE phenotypes were submitted to Ingenuity Pathway Analysis^®^ (IPA) for mapping to canonical pathways and identification of upstream regulators. The analysis revealed again a progressive reprogramming along the *ex vivo* MMT, the number of altered pathways was higher in cells with NE phenotype (52 in E vs 105 in NE) and there was an overlapping of 31 pathways that were deregulated in both groups (Suppl. Tables S9 and S10). Among the canonical pathways that were significantly differentially regulated, many were related to key processes involved in MMT/EMT, including integrin/ILK, intercellular junction, NF-κB, HIF-1α, Wnt/β-catenin, Endothelin-1 (ET-1) and TGF-β signaling pathways (Fig. 2). Pathways endowing MCs with stemness and plasticity were also differentially regulated in cells with E and NE phenotypes, which is in line with recent reports showing that during liver development, MCs act as progenitor cells that produce hepatic stellate cells, fibroblasts around blood vessels, and smooth muscle cells. In addition, during liver injury, MCs migrate inward from the liver surface and produce hepatic stellate cells or myofibroblast depending on the etiology^35^,^36^,^37^. It was also noteworthy that the ET-1 signaling pathway was specifically deregulated in MCs with E phenotype, whereas TGF-β signaling was differentially regulated only in NE cells. This is in agreement with previous data that demonstrated a role for an ET-1/TGF-β axis in the control of the MMT process, in which TGF-β, responsible for the progression and maintaining of the mesenchymal stage, appeared to be downstream of ET-1-mediated signaling^38^.

As expected, the number of upstream regulators was higher in MCs with NE phenotype than in E cells (Table 1). Interestingly, both cell types, E and NE, showed inhibition of E-cadherin (CDH1) as an upstream regulator. It is well known that the disruption of adherent junctions is an early step and one of the key events in MMT^23^. In this context, it has been reported that MMT and peritoneal membrane injury are mediated through cleavage of E-cadherin by MMP-9 and induction of β-catenin signaling^32^. Interestingly, we observed that the Wnt/β-catenin pathway was deregulated in both E and NE cells (Fig. 2). Among the activated upstream regulators in NE cells, we found several inflammatory and pro-fibrotic genes (IL-1B, IL-1A, FGF2, SNAI1 and TGFB2) that are known master molecules in the control of MMT (Table 1). It drew our attention that in MCs with E phenotype the only activated upstream regulator was Peroxisome proliferator-activated receptor gamma (PPARG) (Table 1), suggesting that this nuclear receptor plays a modulatory roll in the early steps of MMT^39^,^40^,^41^. Taken together these data clearly indicated that the genetic fingerprints of effluent-derived MCs discriminated between two stages of MMT: early MMT (E phenotype) and advanced MMT (NE phenotype).

Forty of the top deregulated genes in NE cells from the microarray studies were validated by quantitative RT-PCR analysis (Table 2). In addition, we could demonstrate that a number of these genes were differentially regulated in NE cells when compared with E cells (Suppl. Table S11). It is important to point out that GREM1, a key molecule implicated in EMT in general and MMT in particular^31^,^42^ was further upregulated in NE cells when compared with E cells but the difference between both did not reach statistical significance. This result was in agreement with the microarray studies that showed a similar behavior of GREM1 expression (Suppl. Tables S3 and S5). The early up-regulation of GREM1 suggested that this molecule could act as a master regulator of the MMT process.

**Figure 3:**
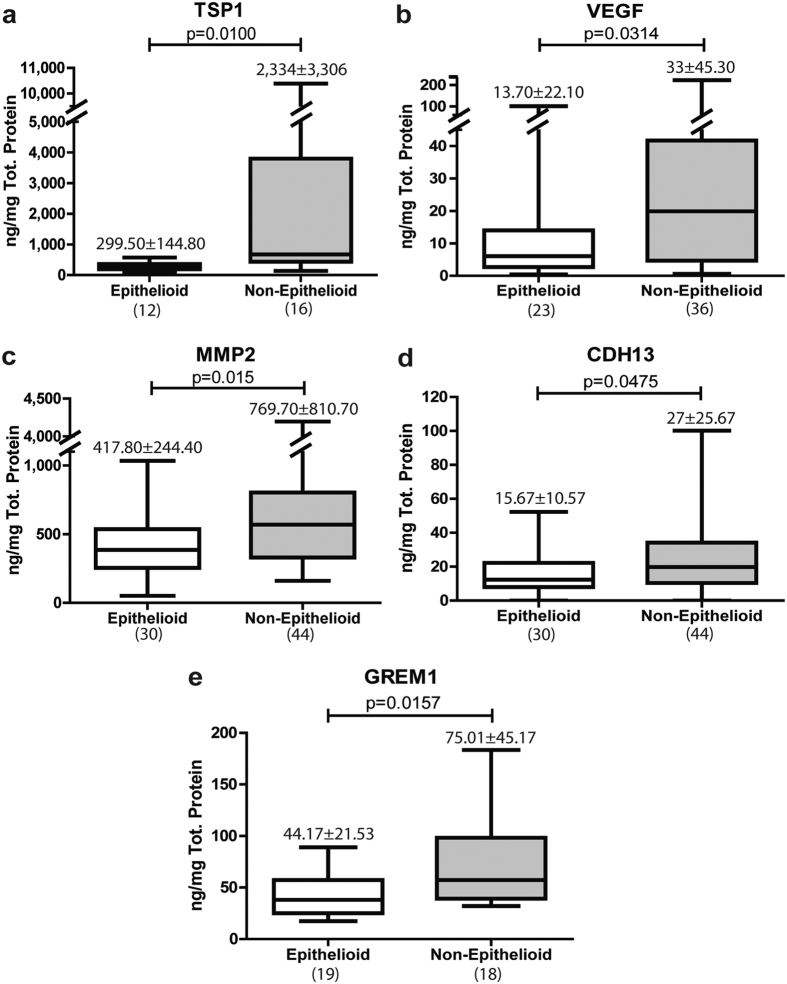
Proteins in supernatants. (**a**) TSP1, (**b**) VEGF, (**c**) MMP2, (**d**) CDH13 and (**e**) GREM1 were measured in culture supernatants of effluent-derived MCs obtained from PD patients. Protein levels were higher in Non-Epithelioid phenotype in these cases. Number on top box-plot represents the mean ± standard deviation. Number in parentheses represents the N of each group. Results were significant with p < 0.05 (Mann-Whitney test).

To validate the study in terms of protein expression, we measured the levels of TSP1, MMP2, CDH13 and GREM1 by ELISA in the culture supernatant of effluent-derived MCs, which were grouped in E and NE phenotypes. VEGF was included as an internal control as we have previously shown the up-regulation of this angiogenic factor along the MMT process^27^,^43^. As shown in Fig. 3, all these MMT-associated proteins, including GREM1, significantly augmented in the culture supernatant of NE cells when compared with MCs bearing E phenotype.

### MMT-associated transcripts discriminate between low-GDPs and high-GDPs PD fluids

We could observe that the prevalence of NE phenotype of MCs tended to be higher in the effluents of PD patients treated with high-GDPs fluids than in patients treated with low-GDPs fluids (Suppl. Table S12). Thus, as a first approach to assess the clinical value of the MMT-associated markers, a subset of 14 genes was analyzed by quantitative RT-PCR in 51 samples of effluent MCs from PD patients treated either with low-GDPs (n = 28) or high-GDPs (n = 23) fluids. As expected, all these genes, except for GREM1, showed significant differences or tendencies between E and NE phenotypes (Table 3). Interestingly, most of these genes showed significant differences (CDH13, COL6A3, COL13A1, KRT34, THBS1 and VEGFA) or a tendency (MMP1 and CDH1) between low-GPDs and high-GDPs fluids (Table 3). However, other genes (KDR, THBD, TFPI2) in spite of showing differences or tendencies between E and NE phenotypes did not appear to be associated with the type of PD fluid (Table 3). Again, GREM1 did not show differences between low-GDPs and high-GDPs fluids.

### Effluent protein levels of TSP1, VEGFA and COL13 are biomarkers for membrane functional decline

**Figure 4:**
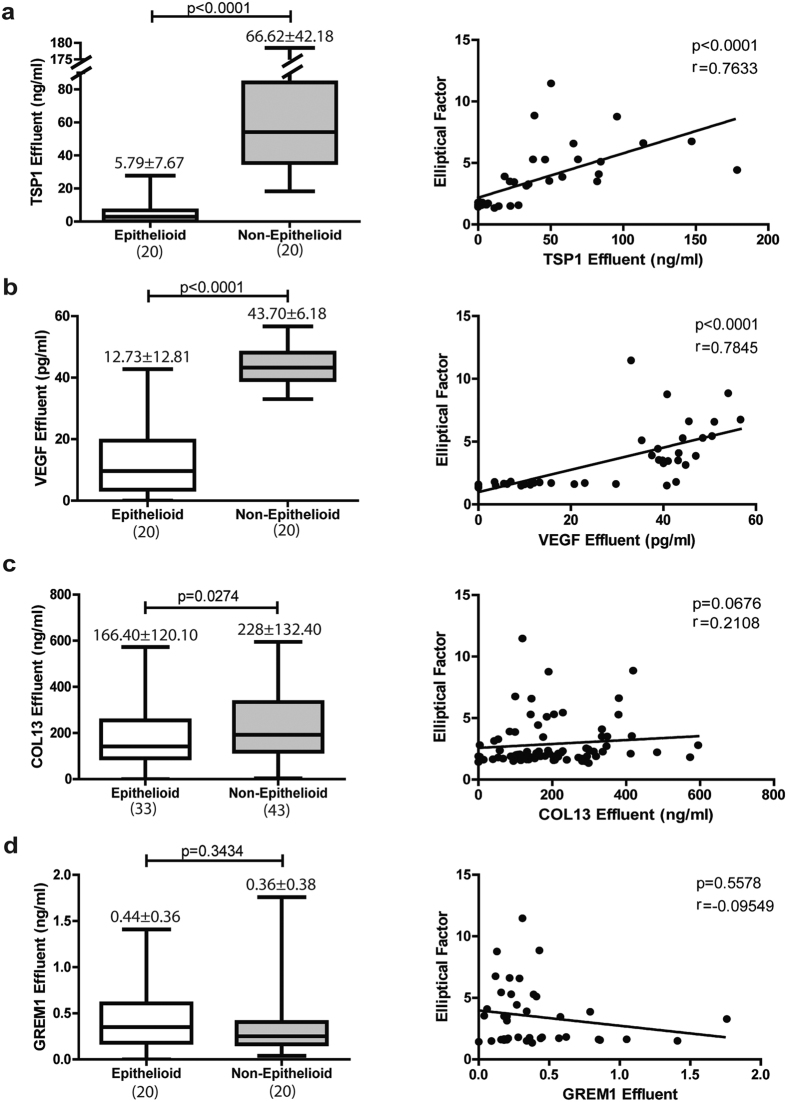
Proteins in effluents by Elliptical Factor. (**a**) TSP1, (**b**) VEGF, (**c**) COL13 and (**d**) GREM1 were measured in effluents. Left panels represent level of proteins grouped by cell phenotype and right show correlations between protein in effluent and Elliptical Factor. Number on top box-plot represents the mean ± standard deviation. Whiskers in box-plot represent maximum and minimum values. Number in parentheses represents the N of each group. Results were significant with p < 0.05 (Mann-Whitney test and Spearman correlation).

**Figure 5:**
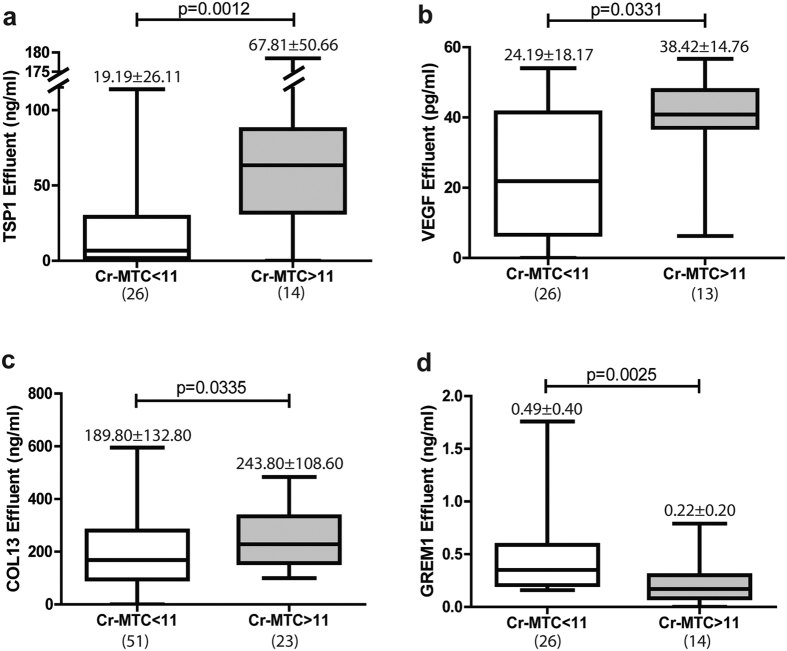
Proteins in effluents by membrane transport. (**a**) TSP1, (**b**) VEGF, (**c**) COL13 and (**d**) GREM1 were measured in effluents. Cr-MTC: Mass Transfer Coefficient of Creatinine (mL/min). Number on top box-plot represents the mean ± standard deviation. Whiskers in box-plot represent maximum and minimum values. Number in parentheses represents the N of each group. Results were significant with p < 0.05 (Mann-Whitney test).

Among the secreted proteins that are up-regulated during MMT *in vitro* and *ex vivo*, thrombospondin-1 (TSP1), vascular endothelial growth factor A (VEGFA), collagen-13 (COL13), and gremlin-1 (GREM1) were selected to be measured in PD effluents. TSP1 and VEGFA levels had marked differences between early (E phenotype) and advanced (NE phenotype) stages of MMT and correlated with the elliptical factor values of effluent-derived MCs (Fig. 4a and b). Effluent levels of COL13 showed significant differences between E and NE phenotypes, but did not reach statistical significance in the correlation with elliptical factor (Fig. 4c). Effluent levels of GREM1 did not show any association with MC phenotype, in agreement with the microarray and RT-PCR studies (Fig. 4d). When PD patients were subdivided into 2 groups according to peritoneal transport characteristics: low/low-average transporters (Cr-MTC < 11 mL/min) and high/high-average transporters (Cr-MTC > 11 mL/min), significantly greater effluent TSP1, VEGF and COL13 concentrations were observed in the last group (Fig. 5a–c). Surprisingly, effluent levels of GREM1 also showed statistical differences between low/low-average and high/high-average transporters, but in the opposite direction to what could be expected with respect to microarray and RT-PCR studies and according to previous reports^31^ (Fig. 5d). It is tempting to speculate that the higher levels of GREM1 in patients with a preserved peritoneal function could be due to a concomitant preservation of the mesothelium.

**Figure 6:**
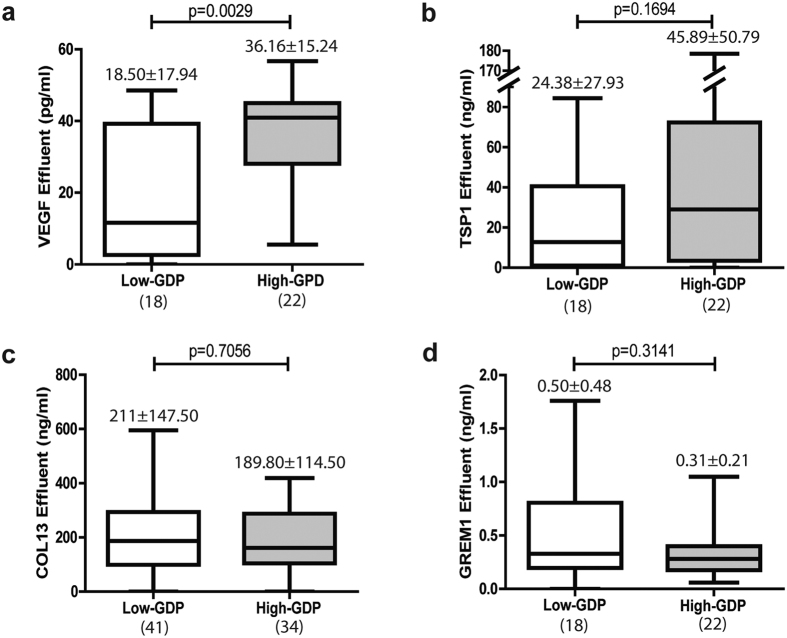
Proteins in effluents by low-GDPs or high-GDPs of Peritoneal Dialysis Fluid. (**a**) VEGF, (**b**) TSP1, (**c**) COL13 and (**d**) GREM1. Number on top box-plot represents the mean ± standard deviation. Whiskers in box-plot represent maximum and minimum values. Number in parentheses represents the N of each group. Only (**a**) VEGF in effluents was significant between low-GDPs and high-GDPs fluids (Mann-Whitney test p-value < 0.05).

Effluent VEGF concentration showed significant difference between low-GDPs and high-GDPs fluids (Fig. 6a), whereas TSP1 levels were increased in patients treated with high-GDPs fluids, compared to those treated with low-GDPs fluids, but the difference did not reach statistical significance (Fig. 6b). In contrast, effluent COL13 did not show differences between both types of fluids (Fig. 6c), which was in sharp contrast with the data obtained in the RT-PCR studies (Table 3). These results suggested that mesenchymal MCs were not the only local source of COL13 during PD-induced peritoneal damage. Again, GREM1 did not show differences between low-GDPs and high-GDPs fluids (Fig. 6d).

## Discussion

Assessment of the progressive peritoneal morphological alterations during PD would require repetitive biopsies, which is not feasible in the clinical routine except during removal of the peritoneal catheter, during kidney transplantation or another laparotomy^14^,^44^,^45^. In addition to a sort of a routine measurement to monitor the peritoneal status for being able to timely intervene and adapt to PD regimen, PD therapy has a substantial need for early biomarkers as a tool to identify patients that are at highest risk for PD-related complications and to guide personalized interventions that may improve clinical outcome in the individual patient. Biomarkers are usually determined in plasma or serum and sometimes in urine. PD offers the unique possibility to investigate peritoneal effluent. PD effluent represents a particularly attractive material for biomarker research as it contains cells and biomolecules that are indicative of the peritoneal transport status as well as of peritoneal health, ongoing pathological processes and even the status of underlying disease and comorbidities of the individual PD patient. It has been proposed that and ideal effluent biomarker for PD should encompass the following properties: 1.- Detectable in peritoneal effluent. 2.- Local release/production within the peritoneal cavity. 3.- Involved in pathology of the peritoneal membrane. 4.- High sensitivity and specificity for the clinical outcome of interest. The research of effluent biomarkers in PD has predominantly been hypothesis-driven and based on patho-mechanisms found to be relevant for the course of disease in PD, such as chronic inflammation and peritoneal membrane remodeling. However, at present the integration of effluent biomarkers into clinical decision making in PD is only modest^46^.

In this study, we hypothesized that MMT is a central process implicated in PD-induced morphological and functional changes of peritoneal membrane and we analyzed and validated, for the first time, the whole genomic reprograming during MMT, *in vitro* and *ex vivo*, with the aim to identify novel biomarkers to be tested in PD-patients. These analyses showed that the number of repressed genes is higher than the number of induced genes, in both *ex vivo* and *in vitro* models, indicating that MMT is primarily a repression process. The analysis also revealed a progressive genetic reprogramming along the MMT *ex vivo*, and discriminated between two stages of MMT in effluent-derived MCs: early MMT (E phenotype) and advanced MMT (NE phenotype). It is generally accepted that the treatment of omentum-derived MCs with TGF-β1, alone or in combination, is a good model of MMT *in vitro* that mimics the mesenchymal conversion of MCs taking place in the peritoneum of PD patients^7^,^21^,^22^. However, the fingerprint of the MMT *in vitro* overlapped only partially with the expression profiles of MMT *ex vivo*. It is worthwhile to point out that MMT may result from an integration of diverse signals triggered by multiple factors operating *in vivo*, being difficult to assign priorities or hierarchies. Thus, the results obtained in cell culture experiments using one or two stimuli only should be interpreted with caution. However, the partial overlapping between *in vitro* and *ex vivo* MMT is valuable for cross-validation of altered genes during the mesenchymal conversion of MCs and may help for the selection of candidate genes as potential biomarkers.

We have previously shown that MCs from effluents with NE phenotype produced higher levels of COX-2 and VEGF than MCs with E phenotype. The levels of *ex vivo* expression of these molecules correlated with the mass transfer area coefficient for creatinine (Cr-MTAC) of PD patients^24^,^27^. Another study demonstrated that high peritoneal transport state was associated with increased CTGF/CCN2 mRNA synthesis by effluent MCs^30^. Recently, it has been described that the level of expression of MMP-9-encoding mRNA by effluent MCs correlated with solute transport data, including dialysate to plasma creatinine ratio (D/P creatinine) and a 4 h to initial PD fluid glucose ratio (D/D0 glucose)^32^. Herein, we demonstrate by RT-PCR array that a subset of genes differentially expressed in effluent-derived NE cells also show differential expression when comparing low-GDPs versus high-GDPs PD fluids. This result is in agreement with previous studies showing that PD fluids with low content of GDPs have less impact *in vivo* and *ex vivo* on MMT than standard non-physiological fluids^14^,^47^,^48^. Taken together, these studies clearly suggest that the acquisition of mesenchymal properties by MCs might be involved in the progression of peritoneal structural alteration and in high transport state, being, therefore, feasible to focus on MMT process *ex vivo* for the search of novel biomarkers in PD.

However, it is no always feasible to employ the *ex vivo* culture of effluent MCs in the regular clinical practice. An alternative approach is the use of the dialysate as a form of “liquid biopsy” in which MMT-associated molecules could be measured directly. As a proof of concept, in this study we have selected three secreted proteins that are up-regulated during MMT (thrombospondin-1; TSP1; collagen-13, COL13; and gremlin-1, GREM1) to be measured in PD effluents. Vascular endothelial growth factor A (VEGFA) was also included as a control since it was previously demonstrated that the levels of this growth factor in *ex vivo* MCs cultures and in PD effluents correlate with the peritoneal transport status and are associated with PD fluid biocompatibility^27^,^28^,^29^,^48^. Effluent levels of TSP1, COL13 and VEGFA levels showed significant differences when comparing patients draining MCs with either early or advanced stages of MMT. In addition, the effluent levels of TSP1, COL13 and VEGFA were clearly associated with high peritoneal transport status (Cr-MTC > 11 mL/min). As expected from the microarray and RT-PCR studies, effluent levels of GREM1 did not show any association with MC phenotype. It is important to point out that GREM1, a key molecule implicated in EMT in general and MMT in particular^31^,^42^ is rapidly up-regulated in effluent-derived MCs, being highly expressed even in early stages of MMT and further augmented in MCs with NE phenotype. Thus, the early up-regulation of GREM1 suggested that this molecule could act as a master regulator of the MMT process. Surprisingly, however, effluent levels of GREM1 were higher in low/low-average than in high/high-average transporters. These results were in sharp contrast with previous reports^31^ and we do not have a definitive explanation for these apparent discrepancies. However, it can be speculated that the higher levels of GREM1 in patients with a preserved peritoneal function could be due to a concomitant preservation of the mesothelial mass. Thus, the ratio between effluent GREM1 and CA125 could represent better the intraperitoneal production of GREM1 per unit mass of MCs and would require further studies^49^.

Of the four selected MMT-related markers, effluent concentrations of TSP1 and VEGFA were associated with dialysis regime (low-GDPs or high-GDPs fluids), whereas effluent COL13, in contrast with the data obtained in the RT-PCR studies, did not show differences between both types of fluids. The weaker correlation of effluent COL13, when compared with TSP1 and VEGFA, with MC phenotypes and peritoneal transport states, and its lack of association with the GDPs content of the fluids suggest that MCs undergoing MMT is not the only source of COL13 during PD-induced peritoneal damage^50^. To reinforce this idea, we could demonstrate that the levels of effluent TSP1 and VEGF, but not of COL13, correlated with the expression of their respective mRNAs by MCs cultured *ex vivo* (Data not shown).

One of the properties for an ideal effluent biomarker is that it must be involved in the pathology of the peritoneal membrane. It is well known that VEGF, which is strongly induced in MCs undergoing MMT during PD, is a key regulator of angiogenesis and its role in peritoneal transport alteration is widely recognized^27^,^28^,^29^,^48^. Conversely, TSP1 has anti-angiogenic properties that are mediated by direct interaction with VEGF and/or by inhibition of MMP9 with the consequent retention of VEGF trapped in ECM^51^. It has been also shown that binding of TSP1 to its receptor CD36 can block the transduction of the VEGF signal through VEGF Receptor 2 (VEGFR2)^51^. In addition, TSP1 is an activator of latent TGF-β1^52^, a master molecule controlling MMT^53^. Thus, during MMT, TSP1 is overexpressed and latent TGF-β1 is activated, further enhancing the MMT process and increasing the synthesis of VEGF, which promotes angiogenesis and peritoneal transport alteration.

Little information is available on the expression of type XIII collagen (COL13) in human diseases. COL13 is a type II trans-membrane protein, with a short intracellular domain and a long extracellular domain that is released by furin proteases and can be incorporated to extracellular matrix^54^. These two forms, trans-membrane and shed protein, have different biological functions and bind the ECM component fibronectin, which is strongly induced during MMT in PD, Nidogen-2, Perlecan or α1β1 integrin^50^,^54^,^55^. It has been demonstrated that factors secreted by tumor cells, in particular TGF-β1, contribute to the induction of COL13 expression by fibroblasts, and that this collagen expression may conversely contribute to tumor progression by modulating cell-matrix interactions^55^.

In conclusion, we provide evidence that measurement of MMT-associated molecules in PD effluents may have clinical value as biomarkers in order to personalize PD treatment and to improve the outcome of PD patients. The next goal would be to develop a combination of MMT-related molecules that can be measured in the PD effluent at once (MMT-Chip) and offer diagnostic value. Finally, well-designed clinical trials are required to prospectively test the proof of concept and clinically confirm the usefulness of the candidate biomarker in independent cohorts.

## Materials and Methods

### Isolation and culture of mesothelial cells

The protocol and informed consent were reviewed and approved by the Ethics Committee of Clinic Investigation of the Centro de Biología Molecular “Severo Ochoa” ref: 2010-0009 (Madrid, Spain) and “Hospital Universitario Puerta de Hierro Majadahonda”, record number 300 (Majadahonda, Madrid, Spain). Informed written consent was obtained from all PD patients and omentum donors prior to the initiation of any study-related activities. This study abides by the Declaration of Helsinki and the research was carried out in accordance with Good Clinical Practice guideline and applicable regulation.

MCs were obtained from omentum taken from patients undergoing elective abdominal surgery as well as from the effluents of PD patients as previously described^22^. Cells were cultured in Earle’s M199 medium (Biological Industries, Kibbutz Beit Haemek, Israel) supplemented with 20% fetal bovine serum (FBS; Thermo Scientific, Cramligton, UK), 50 U/ml penicillin, 50 μg/ml streptomycin (PPA Laboratories GmbH, Pasching, Austria), 2% Biogro-2 (Biological Industries). The purity of the isolated omentum- and effluent-derived MCs was verified by the expression of the standard mesothelial markers intercellular adhesion molecule (ICAM)-1, calretinin and cytokeratins^22^. All cells were negative for the endothelial marker von-Willebrand factor^22^, and passed through CD45-specific columns (CD45 MicroBeads; Miltenyi Biotec GmbH, Bergisch Gladbach, Germany) to eliminate any possible contamination by macrophages^56^.

MCs were classified into epithelioid (E) or non-epithelioid (NE) phenotype according the Elliptical Factor (EF) which represents the relation between the major and the minor cell axis^57^. The dimensions of ten cells, randomly chosen in each culture, were measured using the ImageJ software (National Institutes of Health, Bethesda, Maryland, USA). If the calculated EF ratio was greater than or equal 2, meaning that the cell is twice as long as it is wide, the culture was classified like NE phenotype and, in case the ratio was smaller than 2, it was categorized to have an E phenotype.

### Whole Genome RNA Microarray Analysis

All microarray data were up-loaded in Gene Expression Omnibus (GEO) (www.ncbi.nlm.nig.gov/geo) with reference number GSE92455.

#### Ex vivo

For *ex vivo* RNA microarray analysis, 17 MC samples derived from 16 PD patient’s effluents were cultured and analyzed as described above: 9 populations showed an E phenotype and 8 samples were classified as NE. Four omentum-derived MC samples were taken as controls. As there were no significant differences in their transcriptional patterns, these control cells were pooled for future experiments. Cell lysis and RNA extraction using TRI Reagent^®^ Solution (Ambion, Austin, Texas, USA) was done according to the manufacturer´s instructions. A dye-swap design was used in these experiments, one hybridization sample was labeled in red and the control in green, whereas the other hybridization sample was labeled in green and the control in red, using the Quick Amp Labeling Kit, two-color (Agilent Technologies, Santa Clara, California, USA). RNA microarray analysis was carried out using the Whole Human Genome Microarrays Kit 4 × 44 K (Agilent Technologies) according to the manufacturer’s instructions: 825 ng of cRNA (Cy3-cRNA and Cy5-cRNA) were incubated with blocking agent and fragmentation buffer, 30 minutes, 60 °C. Hybridization buffer was added and hybridization was performed at 65 °C, 17 hours in darkness, stirring at 10 rpm. Finally, slides were introduced in different stringencies buffered saline and in the stabilization solution and drying indicated by Agilent Technologies. Arrays were scanned on the Agilent Technologies G2505B Micro Array Scanner at 5 nm resolution.

#### In vitro

To analyze the gene expression in artificially induced MMT, half of the total cells obtained from omentum-derived MC samples were treated with 0.5 ng/ml human recombinant TGF-β1 and 2 ng/ml IL-1β (R&D Systems, Minneapolis, Minnesota, USA) for 72 h, while the other half was untreated and used as control. RNA extraction and microarray analysis was carried out as described above.

### Microarray data processing and gene expression analysis

The processing and normalization was performed using the Babelomics (http://babelomics.bioinfo.cipf.es) software^58^. Fluorescence raw signal from each probe was corrected with the subtraction of local background and normalized with the loess method (within arrays) or the quantile method (between arrays). Fold change for a gene was determined as a quotient between normalized data from samples under study. Statistics for differential expression was obtained with Limma One Class or Limma Two Class test and corrected for multiple-test with FDR (False Discovery Rate; Benjamini & Hochberg). Genes with fold change ≥ 2 or ≤0.5, and FDR ≤ 0.01, were considered induced or repressed, respectively. Clustering was made by TIGR MeV^59^ v4 software using Euclidian Distance and Average Linkage Ingenuity Pathway Analysis (IPA) software (Qiagen; www.ingenuity.com) was used for determination of Canonical Pathways and Upstream Regulators analysis.

### RT-qPCR microarray data validation

To validate the results obtained by microarray analysis, 7 samples used in the microarray studies (3 with E and 4 with NE phenotype), 11 samples previously not included in the microarrays (2 with E and 9 with NE phenotype) and the control omentum pool (pool of 4 samples) were analyzed by RT-qPCR. For this, RealTime Ready Custom Panel Plates (Roche Applied Science, Penzberg, Germany) were used, and probes and primers were designed using the RealTime Ready Configurator tool (https://configurator.realtimeready.roche.com/assaysupply_cp/login.jsf, Roche Applied Science). 40 genes were validated: AQP1, BMP4, BMP7, CAV1, CD44, CDH1, CDH3, CDH13, CLDN1, COL1A2, COL4A6, COL6A3, COL13A1, CTSK, CXCL14, FAP, FSP1, HSPA2, HSPB8, IL1B, IL6, IL33, ITGA11, ITGB4, KDR, KRT34, LAMA5, MMP1, MMP2, MMP3, PRG4, SNAI1, TFPI2, THBD, THBS1, THBS2, VCAN, VTN and WT1.

RNA was extracted using the RNeasy Mini Kit (Qiagen, Germantown, Maryland, USA) according to the manufacturer’s instructions. cDNA synthesis was done using the Transcriptor First Strand cDNA Synthesis Kit (Roche Applied Science); program as follows: 25 °C, 10 minutes; 55 °C, 30 minutes; 85 °C, 5 minutes; 4 °C. RT-qPCR was conducted using the LightCycler^®^ 480 with SYBR Green system (Roche Applied Science) with the following program: 95 °C, 10 minutes; 45 cycles of 95 °C, 10 seconds; 60 °C, 30 seconds; 72 °C, 1 second; 4 °C. Data normalized to the housekeeping gene TBP were analyzed using the 2^−ΔΔCt^ method.

For GREM1 validation, the TaqMan^®^ Assay System (Applied Biosystems; Foster City, California, USA) was used. Oligos for GREM1 (Hs00171951_m1; Applied Biosystems and 18 S (5’ATCCATTGGAGGGCAAGTC3’ and 5’GCTCCCAAGATCCAACTACG3)’; Roche Applied Science) and TaqMan^®^ FAM™ dye (Applied Biosystems) were used. RT-qPCR was conducted in the LightCycler^®^ 480 (Roche Applied Science) using the FastStart Universal Probe Master (ROX) (Roche Applied Science) with the following program: 50 °C, 2 minutes; 95 °C, 10 minutes; 60 cycles of 95 °C, 15 seconds and 60 °C, 10 seconds; 4 °C.

These data were analyzed by One-sample Limma, a variant of t-test, and a p-value was obtained to validate the microarray studies. FDR multiple testing correction was calculated.

### Gene expression analysis by RT-qPCR

26 patients (Baseline characteristics in Suppl. Table S13) were included in this study and 51 RNA samples from effluent-derived MC taken from these patients at different time points (2 to 24 months) were used for RT-qPCR. Omentum-derived MCs samples were used as control. The patients were either under Low GDPs containing fluid (Balance, Fresenius Medical Care) (N = 16) or High GDPs containing fluid (StaySafe, Fresenius Medical Care) (N = 10) PD regimens. Cell lysis and RNA extraction was done using TRI Reagent^®^ Solution (Ambion,) according to the manufacturer´s instructions. Reverse transcription was realized used High Capacity RNA to cDNA kit (Applied Biosystems). The expression of the following 14 genes (Assay ID; Applied Biosystems) was assessed: CD44 (Hs01075861_m1), CDH1 (Hs01023894_m1), CDH13 (Hs01004530_m1), COL6A3 (Hs00915125_m1), COL13A1 (Hs00193225_m1), GREM1 (Hs00171951_m1), KDR (Hs00911700_m1), KRT34 (Hs02569742_s1), MMP1 (Hs00899658_m1), MMP2 (Hs01548727_m1), TFPI2 (Hs00197918_m1), THBD (Hs00264920_s1), THBS1 (Hs00962908_m1) and VEGF (Hs00900055_m1). The housekeeping gene TBP (Hs00427620_m1) was used as control.

RT-qPCR was conducted using the Taqman^®^ Gene Expression Assay (Applied Biosystems and Life Technologies; Carlsbad, California, USA) in 384 well plates with 2.5 μl of cDNA, 0.5 μl of Assay/primers, 5 μl of Taqman^®^ Gene Expression Master Mix (Applied Biosystems) and 2 μl of water per well. The 7900 HT Fast Real-Time PCR System (Applied Biosystems) was used following conditions: 10’ 95 °C, 40 cycles of 15 s 95 °C, 1’ 60 °C. Data were recovered with SDS Software and analyzed with RQ Manager and DataAssist™ Software (Life Technologies; Carlsbad, California, USA). Data were normalized against TBP by 2^−ΔΔCt^ method were selected analysis.

Two-tailed Mann-Whitney U-test was used to determinate significant differences between groups and corrects with FDR multiple testing correction.

### Quantification of secreted protein by ELISA

Overnight peritoneal dialysis effluents from patients were collected and stored in −80 °C until used for protein analysis by ELISA. Effluent-derived MCs, effluent and supernatant samples were obtained from 26 PD patients (Suppl. Table S13) selected from ‘La Paz’ Hospital (Madrid, Spain) at initiation of PD (N = 25), 12 months (N = 23), 18 months (N = 18) and 24 months (N = 9). Protein levels of potential MMT markers TSP-1, VEGF (both R&D Systems), Collagen-13, Cadherin 13, Gremlin 1 (all of USCN Life Science Inc; Houston, Texas, USA), and MMP2 (Abnova Corporation; Taipei, Taiwan), which were identified in the microarray analysis, were analyzed in culture supernatant of effluent-derived MC by enzyme-linked immunosorbent assay (ELISA). ELISAs were performed according to the respective manufacturer’s instructions. TSP-1, Collagen-13, Cadherin 13 and Gremlin1 were selected by their high mRNA expression in Non-Epithelioid phenotype, their expression in both *ex vivo* and *in vitro* models, their potentially secretable nature and consequently detectable in peritoneal effluents. Their relative novelty in this context also was considered. VEGF was selected as an internal control.

Two-tailed Mann-Whitney U-test was used to determinate significant differences between groups and Spearman correlation to linear regression between proteins in effluents and the Elliptical Factor. No correction for multiple testing was applied because this is still an exploratory study.

### Statistical analysis

For statistical analysis, GraphPad Prism v5 (GraphPad Software, Inc; La Jolla, California, USA) was used. Results are presented as mean values and SD. D’Agostino & Pearson normality test showed that the data were not normally distributed and indicated the use of non-parametric tests. One-sample Limma was used in microarray validation by RT-PCR, Mann-Whitney U-test was made for comparison between different groups in supernatants, effluents and patient mRNAs, Chi-Square Test was used to analyze the relation between phenotype and GDP-content, and a Spearman correlation was made to determinate linear regressions. Comparisons between groups in patient’s mRNAs were corrected with FDR multiple testing correction (TIGR MeV v4 software). p ≤ 0.05 was considered statistically significant.

## Additional Information

**How to cite this article**: Ruiz-Carpio, V. *et al*. Genomic reprograming analysis of the Mesothelial to Mesenchymal Transition identifies biomarkers in peritoneal dialysis patients. *Sci. Rep.* **7**, 44941; doi: 10.1038/srep44941 (2017).

**Publisher's note:** Springer Nature remains neutral with regard to jurisdictional claims in published maps and institutional affiliations.

## Supplementary Material

Supplementary Information

## Figures and Tables

**Table 1:** Upstream Regulators.

Cell Phenotype	Upstream Regulator	Exp Fold Change	Molecule Type	Predicted Activation State	Activation z-score	p-value of overlap
**Epithelioid**	PPARG	2.32	ligand-dependent nuclear receptor	Activated	2.69	6.86E-04
	**CDH1**	−2.54	other	Inhibited	−1.59	1.25E-02
**Non-Epithelioid**	IL1B	15.27	cytokine	Activated	2.95	7.79E-30
	MMP3	9.08	peptidase	Activated	2.19	4.47E-01
	F2R	4.46	g-protein coupled receptor	Activated	2.78	6.05E-06
	PTGS2	4.03	enzyme	Activated	3.01	1.23E-09
	SPP1	3.57	cytokine	Activated	3.40	3.72E-07
	ITGA2	3.08	transmembrane receptor	Activated	2.43	6.28E-04
	FGF2	2.93	growth factor	Activated	2.64	4.55E-19
	IL1A	2.73	cytokine	Activated	4.73	1.02E-10
	ADORA2B	2.57	g-protein coupled receptor	Activated	2.34	5.42E-05
	NRIP1	2.53	transcription regulator	Activated	2.38	4.39E-03
	SNAI1	2.41	transcription regulator	Activated	2.25	3.92E-07
	TGFB2	2.23	growth factor	Activated	2.09	5.88E-08
	HMGB1	2.01	transcription regulator	Activated	3.08	5.35E-03
	**WNT4**	−4.56	cytokine	Inhibited	−2	1.03E-04
	**GHR**	−2.11	transmembrane receptor	Inhibited	−2.19	1.47E-02
	**CDH1**	−11.24	other	Inhibited	−2.87	1.43E-07

Table columns (left to right): Cell phenotype; Upstream regulator genes; Gene’s fold change determined in microarray analysis; Molecule type; Predicted activation state of upstream regulator; Z-score: if z-score ≥ 2 then upstream regulator is activated, and if z-score ≤ −2 then is inhibited; p-value, calculated by Fisher’s Exact Test, indicates the statistical significance of genes in the data set that are downstream of de upstream regulator. Normal letter: up-regulated genes; bold: down-regulated genes in microarrays.

**Table 2:** Microarray validation by PCR-Array.

GENE	Non-Epithelioid		
	Fold change	p-value	FDR
GREM1	7.82	<0.001	
IL1B	27.70	<0.001	<0.001
KRT34	37.36	<0.001	<0.001
CDH13	65.76	<0.001	<0.001
THBS1	25.87	<0.001	<0.001
COL6A3	43.34	<0.001	<0.001
MMP1	16.81	<0.001	<0.001
COL13A1	7.71	<0.001	<0.001
IL6	2.52	0.015	0.017
FSP1	11.32	<0.001	<0.001
TFPI2	26.61	<0.001	<0.001
CAV1	4.51	<0.001	<0.001
VCAN	4.94	<0.001	<0.001
IL33	8.08	<0.001	<0.001
ITGA11	7.26	<0.001	<0.001
THBS2	3.93	<0.001	<0.001
MMP3	10.50	<0.001	<0.001
SNAI1	3.65	<0.001	<0.001
COL1A2	2.12	0.021	0.024
CD44	8.41	<0.001	<0.001
MMP2	4.28	<0.001	<0.001
FAP	3.04	<0.001	<0.001
CTSK	2.94	<0.001	<0.001
CLDN1	0.32	<0.001	<0.001
COL4A6	0.43	<0.001	<0.001
WT1	0.36	<0.001	<0.001
HSPB8	0.48	<0.001	<0.001
ITGB4	0.38	<0.001	<0.001
AQP1	0.19	<0.001	<0.001
VTN	0.20	<0.001	<0.001
BMP4	0.21	<0.001	<0.001
CDH3	0.28	<0.001	<0.001
HSPA2	0.42	<0.001	<0.001
KDR	0.21	<0.001	<0.001
CDH1	0.08	<0.001	<0.001
LAMA5	0.24	<0.001	<0.001
CXCL14	0.19	<0.001	<0.001
BMP7	0.07	<0.001	<0.001
THBD	0.05	<0.001	<0.001
PRG4	0.06	<0.001	<0.001

A total of 40 genes, 22 induced and 18 repressed, were selected from Non-Epithelioid group in microarray and validated by RT-qPCR. Fold change, p-value (One-sample Limma test) and associated FDR multiple testing correction are show. Normal letter: up-regulated genes; bold: down-regulated genes in microarrays.

**Table 3:** mRNA of patients.

GEN	Epithelioid (N = 23)		Non-Epithelioid (N = 28)		p value	FDR	Low-GDP (N = 28)		High-GDP (N = 23)		p value	FDR
	Mean	SD	Mean	SD			Mean	SD	Mean	SD		
GREM1	4.03	4.14	7.61	11.97	0.798	0.798	5.45	9.19	6.66	9.80	0.977	0.977
CD44	2.88	2.94	4.87	3.34	**0.001**	**0.011**	3.53	2.94	4.51	3.66	0.135	0.185
CDH13	15.07	13.96	26.16	23.03	**0.049**	0.062	12.39	8.67	31.84	24.64	**0.003**	**0.016**
COL6A3	8.80	10.07	18.03	20.91	**0.020**	0.038	8.06	8.19	20.93	22.57	**0.006**	**0.016**
COL13A1	1.39	1.34	3.16	3.54	**0.016**	**0.038**	1.37	1.27	3.57	3.77	**0.006**	**0.016**
KRT34	7.59	8.23	27.21	33.15	**0.012**	**0.038**	8.73	10.64	30.08	35.17	**0.010**	**0.020**
MMP1	1.37	1.25	6.73	9.83	**0.002**	0.011	4.27	9.49	4.36	5.12	0.066	0.114
MMP2	3.97	3.90	4.64	3.27	0.116	0.125	3.61	3.02	5.23	3.98	0.096	0.149
TFPI2	5.80	5.35	10.57	9.91	0.016	0.038	8.42	6.66	8.42	10.38	0.691	0.798
THBS1	7.36	8.54	19.41	24.98	0.022	0.038	5.78	5.31	23.96	26.41	**0.000**	**0.002**
VEGFA	1.84	1.32	3.32	3.29	0.088	0.105	1.90	1.85	3.57	3.24	**0.007**	**0.016**
**CDH1**	0.61	0.46	0.25	0.25	**0.001**	**0.038**	0.38	0.43	0.46	0.35	0.061	**0.016**
**KDR**	0.87	0.55	0.63	0.52	**0.041**	0.062	0.66	0.48	0.83	0.60	0.325	0.408
**THBD**	0.46	0.53	0.19	0.12	**0.047**	0.062	0.37	0.50	0.24	0.18	1.000	0.977

A total of 14 genes, 11 induced and 3 repressed in microarray studies, were analyzed in 51 samples of effluent-derived MCs in PD patients. Genes grouped by cell phenotype: A statistically significant difference (or tendency to significance) was observed in 12 mRNAs. Induced genes were higher in Non-Epithelioid phenotype, whilst in repressed genes were higher in Epithelioid phenotype. Genes grouped by content of GDPs in Peritoneal Dialysis Fluid: 8 mRNAs showed significant difference (or tendency) between low-GDP and high-GDP fluids. Normal letter: up-regulated genes; bold: down-regulated genes. Mann-Whitney U-test was used to compare between groups and corrected with multiple testing FDR. Statistically significant differences are marked in bold.
