# Supplementary material for: Genomic reprograming analysis of the Mesothelial to Mesenchymal Transition identifies biomarkers in peritoneal dialysis patients
Source: Sci Rep. 2017 Mar 22;7:44941. doi: 10.1038/srep44941 (PMC5361179; doi:10.1038/srep44941)
Supplement: Supplementary Information [file srep44941-s1.pdf]

Genomic reprograming analysis of the Mesothelial to Mesenchymal Transition identifies biomarkers in peritoneal dialysis patients

Vicente Ruiz-Carpio[1], Pilar Sandoval[1], Abelardo Aguilera[2,*], Patricia Albar-Vizcaíno[2], María Luisa Perez-Lozano[1], Guadalupe T. González-Mateo[1], Adrián Acuña-Ruiz[1], Jesús García-Cantalejo[3], Pedro Botías[3], María Auxiliadora Bajo[4], Rafael Selgas[4], José Antonio Sánchez-Tomero[2], Jutta Passlick-Deetjen[5], Dorothea Piecha[5], Janine Büchel[5], Sonja Steppan[5], Manuel López-Cabrera[1,*].

1 Departamento de Biología Celular e Inmunología, Centro de Biología Molecular "Severo Ochoa", CSIC-UAM, Cantoblanco, Madrid, Spain.

2 Unidad de Biología Molecular y Servicio de Nefrología, Hospital Universitario de La Princesa, Instituto de Investigación Sanitaria Princesa (IP), Madrid, Spain.

3 Unidad de Genómica, CAI de Genómica y Proteómica, Universidad Complutense de Madrid, Madrid, Spain.

4 Servicio de Nefrología, Hospital Universitario La Paz, Instituto de Investigación Sanitaria La Paz (IdiPAZ), Madrid, Spain.

5 Fresenius Medical Care Deutschland GmbH, Else-Kröner-Straβe 1, 61352 Bad Homburg, Germany

*** Corresponding Author:**

Dr. Manuel López-Cabrera

Centro de Biología Molecular "Severo Ochoa", CSIC-UAM

Campus de Cantoblanco, Universidad Autónoma de Madrid,

C/ Nicolás Cabrera, 1. 28049 Madrid. Spain

Phone: +34 911964604

e-mail: mlcabrera@cbm.csic.es

Dr. Abelardo Aguilera

Unidad de Biología Molecular y Servicio de Nefrología,

Instituto de Investigación Sanitaria Princesa (IP),

C/ Diego de León, 62. 28006-Madrid. Spain.

Phone: +34 915202200 (Ext. 19506)

e-mail: abelardo.aguilera@salud.madrid.org

Supplementary Table S1. Microarray induced genes in vitro.

Omentum A	Omentum C	Gene Symbol	Systematic Name	Description
17,84	7,75	SERPINE2	NM_006216	Homo sapiens serpin peptidase inhibitor, clade E (nexin, plasminogen activator inhibitor type 1), member 2 (SERPINE2), mRNA [NM_006216]
14,09	9,06	XRCC4	NM_022550	Homo sapiens X-ray repair complementing defective repair in Chinese hamster cells 4 (XRCC4), transcript variant 3, mRNA [NM_022550]
12,85	7,40	THBS1	NM_003246	Homo sapiens thrombospondin 1 (THBS1), mRNA [NM_003246]
11,93	8,39	VCAN	NM_004385	Homo sapiens versican (VCAN), mRNA [NM_004385]
11,34	10,45	ANGPTL4	NM_139314	Homo sapiens angiopoietin-like 4 (ANGPTL4), transcript variant 1, mRNA [NM_139314]
9,81	10,63	FN1	NM_212482	Homo sapiens fibronectin 1 (FN1), transcript variant 1, mRNA [NM_212482]
9,78	12,43	GPC6	ENST00000377047	Glypican-6 precursor. [Source:Uniprot/SWISSPROT;Acc:Q9Y625] [ENST00000377047]
9,47	11,49	TNC	NM_002160	Homo sapiens tenascin C (hexabrachion) (TNC), mRNA [NM_002160]
8,44	6,95	ITGA11	NM_001004439	Homo sapiens integrin, alpha 11 (ITGA11), transcript variant 1, mRNA [NM_001004439]
7,76	19,58	IL33	NM_033439	Homo sapiens interleukin 33 (IL33), mRNA [NM_033439]
7,28	7,96	INHBA	NM_002192	Homo sapiens inhibin, beta A (activin A, activin AB alpha polypeptide) (INHBA), mRNA [NM_002192]
7,01	5,21	COL1A1	Z74615	H.sapiens mRNA for prepro-alpha1(I) collagen. [Z74615]
6,37	4,30	DAPP1	NM_014395	Homo sapiens dual adaptor of phosphotyrosine and 3-phosphoinositides (DAPP1), mRNA [NM_014395]
6,19	2,30	LTBP2	NM_000428	Homo sapiens latent transforming growth factor beta binding protein 2 (LTBP2), mRNA [NM_000428]
6,12	4,15	VEGFA	NM_001025366	Homo sapiens vascular endothelial growth factor A (VEGFA), transcript variant 1, mRNA [NM_001025366]
6,07	4,94	ADAM12	NM_003474	Homo sapiens ADAM metallopeptidase domain 12 (meltrin alpha) (ADAM12), transcript variant 1, mRNA [NM_003474]
5,66	5,73	TGFBI	NM_000358	Homo sapiens transforming growth factor, beta-induced, 68kDa (TGFBI), mRNA [NM_000358]
5,65	9,94	AMIGO2	NM_181847	Homo sapiens adhesion molecule with Ig-like domain 2 (AMIGO2), mRNA [NM_181847]
5,50	5,73	FHOD3	NM_025135	Homo sapiens formin homology 2 domain containing 3 (FHOD3), mRNA [NM_025135]
5,50	2,65	PLAU	NM_002658	Homo sapiens plasminogen activator, urokinase (PLAU), mRNA [NM_002658]

5,36	6,30	RGS4	NM_005613	Homo sapiens regulator of G-protein signalling 4 (RGS4), mRNA [NM_005613]
4,97	4,01	THBS2	NM_003247	Homo sapiens thrombospondin 2 (THBS2), mRNA [NM_003247]
4,82	2,34	SERPINE1	NM_000602	Homo sapiens serpin peptidase inhibitor, clade E (nexin, plasminogen activator inhibitor type 1), member 1 (SERPINE1), mRNA [NM_000602]
4,79	5,58	VDR	NM_001017535	Homo sapiens vitamin D (1,25- dihydroxyvitamin D3) receptor (VDR), transcript variant 2, mRNA [NM_001017535]
4,66	8,29	EGR1	NM_001964	Homo sapiens early growth response 1 (EGR1), mRNA [NM_001964]
4,62	5,36	FAP	NM_004460	Homo sapiens fibroblast activation protein, alpha (FAP), mRNA [NM_004460]
4,58	4,62	FN1	NM_054034	Homo sapiens fibronectin 1 (FN1), transcript variant 7, mRNA [NM_054034]
4,57	5,73	SLC2A1	NM_006516	Homo sapiens solute carrier family 2 (facilitated glucose transporter), member 1 (SLC2A1), mRNA [NM_006516]
4,56	12,07	ENST00000358356	ENST00000358356	Runt-related transcription factor 1 (Core-binding factor, alpha 2 subunit) (CBF-alpha 2) (Acute myeloid leukemia 1 protein) (Oncogene AML-1) (Polyomavirus enhancer-binding protein 2 alpha B subunit) (PEBP2-alpha B) (PEA2-alpha B) (SL3-3 enhancer...
4,27	4,88	RRAD	NM_004165	Homo sapiens Ras-related associated with diabetes (RRAD), mRNA [NM_004165]
4,18	3,19	EDIL3	NM_005711	Homo sapiens EGF-like repeats and discoidin I-like domains 3 (EDIL3), mRNA [NM_005711]
4,14	3,98	DDIT4	NM_019058	Homo sapiens DNA-damage-inducible transcript 4 (DDIT4), mRNA [NM_019058]
4,01	2,04	CALU	NM_001219	Homo sapiens calumenin (CALU), mRNA [NM_001219]
4,00	3,10	IGFBP3	NM_001013398	Homo sapiens insulin-like growth factor binding protein 3 (IGFBP3), transcript variant 1, mRNA [NM_001013398]
3,98	6,04	IL6	NM_000600	Homo sapiens interleukin 6 (interferon, beta 2) (IL6), mRNA [NM_000600]
3,94	3,20	P4HA2	NM_001017973	Homo sapiens procollagen-proline, 2-oxoglutarate 4-dioxygenase (proline 4-hydroxylase), alpha polypeptide II (P4HA2), transcript variant 2, mRNA [NM_001017973]
3,88	4,25	DACT1	NM_016651	Homo sapiens dapper, antagonist of beta-catenin, homolog 1 (Xenopus laevis) (DACT1), transcript variant 1, mRNA [NM_016651]
3,79	4,08	MST150	NM_032947	Homo sapiens MSTP150 (MST150), mRNA [NM_032947]
3,78	2,67	P4HA3	NM_182904	Homo sapiens procollagen-proline, 2-oxoglutarate 4-dioxygenase (proline 4-hydroxylase), alpha polypeptide III (P4HA3), mRNA [NM_182904]
3,62	5,42	CCL2	NM_002982	Homo sapiens chemokine (C-C motif) ligand 2 (CCL2), mRNA [NM_002982]
3,61	5,30	IL1B	NM_000576	Homo sapiens interleukin 1, beta (IL1B), mRNA [NM_000576]
3,57	3,46	SLC2A5	NM_003039	Homo sapiens solute carrier family 2 (facilitated glucose/fructose transporter), member 5 (SLC2A5), mRNA [NM_003039]

3,54	4,14	GREM1	NM_013372	Homo sapiens gremlin 1, cysteine knot superfamily, homolog (Xenopus laevis) (GREM1), mRNA [NM_013372]
3,52	3,31	THBS2	L12350	Human thrombospondin 2 (THBS2) mRNA, complete cds. [L12350]
3,34	4,90	ALOX5AP	NM_001629	Homo sapiens arachidonate 5-lipoxygenase-activating protein (ALOX5AP), mRNA [NM_001629]
3,29	2,74	ADAM19	NM_033274	Homo sapiens ADAM metallopeptidase domain 19 (meltrin beta) (ADAM19), transcript variant 2, mRNA [NM_033274]
3,28	2,69	ITGA2	NM_002203	Homo sapiens integrin, alpha 2 (CD49B, alpha 2 subunit of VLA-2 receptor) (ITGA2), mRNA [NM_002203]
3,28	2,51	ENO2	NM_001975	Homo sapiens enolase 2 (gamma, neuronal) (ENO2), mRNA [NM_001975]
3,27	4,46	LOX	NM_002317	Homo sapiens lysyl oxidase (LOX), mRNA [NM_002317]
3,25	2,65	CRLF1	NM_004750	Homo sapiens cytokine receptor-like factor 1 (CRLF1), mRNA [NM_004750]
3,25	2,17	HNT	NM_016522	Homo sapiens neurotrimin (HNT), transcript variant 1, mRNA [NM_016522]
3,20	2,25	COL6A3	NM_004369	Homo sapiens collagen, type VI, alpha 3 (COL6A3), transcript variant 1, mRNA [NM_004369]
3,20	2,75	KLHDC7B	NM_138433	Homo sapiens kelch domain containing 7B (KLHDC7B), mRNA [NM_138433]
3,19	5,15	ZC3H12A	NM_025079	Homo sapiens zinc finger CCCH-type containing 12A (ZC3H12A), mRNA [NM_025079]
3,17	2,02	GPC6	NM_005708	Homo sapiens glypican 6 (GPC6), mRNA [NM_005708]
3,15	4,92	PSAT1	NM_058179	Homo sapiens phosphoserine aminotransferase 1 (PSAT1), transcript variant 1, mRNA [NM_058179]
3,15	3,18	POSTN	NM_006475	Homo sapiens periostin, osteoblast specific factor (POSTN), mRNA [NM_006475]
3,14	3,78	SPRR2G	NM_001014291	Homo sapiens small proline-rich protein 2G (SPRR2G), mRNA [NM_001014291]
3,11	2,88	SULF1	NM_015170	Homo sapiens sulfatase 1 (SULF1), mRNA [NM_015170]
3,09	5,45	COL16A1	NM_001856	Homo sapiens collagen, type XVI, alpha 1 (COL16A1), mRNA [NM_001856]
3,07	3,69	LOXL2	NM_002318	Homo sapiens lysyl oxidase-like 2 (LOXL2), mRNA [NM_002318]
3,07	2,19	TNFRSF6B	NM_032945	Homo sapiens tumor necrosis factor receptor superfamily, member 6b, decoy (TNFRSF6B), transcript variant M68C, mRNA [NM_032945]
3,06	2,95	SYNPO	ENST00000307662	Synaptopodin. [Source:Uniprot/SWISSPROT;Acc:Q8N3V7] [ENST00000307662]
3,05	2,94	SCG2	NM_003469	Homo sapiens secretogranin II (chromogranin C) (SCG2), mRNA [NM_003469]
3,05	2,52	LOC497190	NM_001011880	Homo sapiens secretory protein LOC497190 (LOC497190), mRNA [NM_001011880]
3,04	3,47	PID1	NM_017933	Homo sapiens phosphotyrosine interaction domain containing 1 (PID1), mRNA [NM_017933]

3,00	4,45	MTHFD2	NM_006636	Homo sapiens methylenetetrahydrofolate dehydrogenase (NADP+ dependent) 2, methenyltetrahydrofolate cyclohydrolase (MTHFD2), nuclear gene encoding mitochondrial protein, transcript variant 1, mRNA [NM_006636]
2,96	2,57	HS3ST3B1	ENST00000360954	Heparan sulfate glucosamine 3-O-sulfotransferase 3B1 (EC 2.8.2.30) (Heparan sulfate D-glucosaminyl 3-O-sulfotransferase 3B1) (Heparan sulfate 3-O-sulfotransferase 3B1) (h3-OST-3B). [Source:Uniprot/SWISSPROT;Acc:Q9Y662] [ENST00000360954]
2,96	7,35	TNFAIP6	NM_007115	Homo sapiens tumor necrosis factor, alpha-induced protein 6 (TNFAIP6), mRNA [NM_007115]
2,94	2,58	TFPI2	NM_006528	Homo sapiens tissue factor pathway inhibitor 2 (TFPI2), mRNA [NM_006528]
2,91	5,73	ABCC3	NM_003786	Homo sapiens ATP-binding cassette, sub-family C (CFTR/MRP), member 3 (ABCC3), mRNA [NM_003786]
2,90	2,40	TREM1	NM_018643	Homo sapiens triggering receptor expressed on myeloid cells 1 (TREM1), mRNA [NM_018643]
2,86	2,63	P2RY6	NM_176798	Homo sapiens pyrimidinergic receptor P2Y, G-protein coupled, 6 (P2RY6), transcript variant 2, mRNA [NM_176798]
2,86	2,30	FAM101A	NM_181709	Homo sapiens family with sequence similarity 101, member A (FAM101A), mRNA [NM_181709]
2,84	3,28	MXRA5	NM_015419	Homo sapiens matrix-remodelling associated 5 (MXRA5), mRNA [NM_015419]
2,83	2,20	CDKN2B	NM_078487	Homo sapiens cyclin-dependent kinase inhibitor 2B (p15, inhibits CDK4) (CDKN2B), transcript variant 2, mRNA [NM_078487]
2,83	2,58	P4HA1	NM_000917	Homo sapiens procollagen-proline, 2-oxoglutarate 4-dioxygenase (proline 4-hydroxylase), alpha polypeptide I (P4HA1), transcript variant 1, mRNA [NM_000917]
2,83	2,32	F2R	NM_001992	Homo sapiens coagulation factor II (thrombin) receptor (F2R), mRNA [NM_001992]
2,80	4,10	P4HA2	NM_004199	Homo sapiens procollagen-proline, 2-oxoglutarate 4-dioxygenase (proline 4-hydroxylase), alpha polypeptide II (P4HA2), transcript variant 1, mRNA [NM_004199]
2,78	3,01	NFKBIZ	NM_031419	Homo sapiens nuclear factor of kappa light polypeptide gene enhancer in B-cells inhibitor, zeta (NFKBIZ), transcript variant 1, mRNA [NM_031419]
2,74	2,34	IDS	NM_000202	Homo sapiens iduronate 2-sulfatase (Hunter syndrome) (IDS), transcript variant 1, mRNA [NM_000202]
2,73	3,00	DUSP6	NM_001946	Homo sapiens dual specificity phosphatase 6 (DUSP6), transcript variant 1, mRNA [NM_001946]
2,71	2,04	PFKFB4	NM_004567	Homo sapiens 6-phosphofructo-2-kinase/fructose-2,6-biphosphatase 4 (PFKFB4), mRNA [NM_004567]
2,65	3,96	GDF15	NM_004864	Homo sapiens growth differentiation factor 15 (GDF15), mRNA [NM_004864]

2,65	2,49	PTGS2	NM_000963	Homo sapiens prostaglandin-endoperoxide synthase 2 (prostaglandin G/H synthase and cyclooxygenase) (PTGS2), mRNA [NM_000963]
2,63	3,11	SKIL	NM_005414	Homo sapiens SKI-like oncogene (SKIL), mRNA [NM_005414]
2,62	2,12	TXNDC5	NM_022085	Homo sapiens thioredoxin domain containing 5 (TXNDC5), transcript variant 2, mRNA [NM_022085]
2,62	3,75	SNAI2	NM_003068	Homo sapiens snail homolog 2 (Drosophila) (SNAI2), mRNA [NM_003068]
2,58	2,29	PPAPDC1A	NM_001030059	Homo sapiens phosphatidic acid phosphatase type 2 domain containing 1A (PPAPDC1A), mRNA [NM_001030059]
2,58	2,06	CCPG1	NM_020739	Homo sapiens cell cycle progression 1 (CCPG1), transcript variant 2, mRNA [NM_020739]
2,57	2,52	MGC23985	NM_206966	Homo sapiens similar to AVLV472 (MGC23985), mRNA [NM_206966]
2,53	2,11	CCPG1	NM_004748	Homo sapiens cell cycle progression 1 (CCPG1), transcript variant 1, mRNA [NM_004748]
2,53	2,61	CTHRC1	NM_138455	Homo sapiens collagen triple helix repeat containing 1 (CTHRC1), mRNA [NM_138455]
2,53	2,79	SLC16A3	NM_004207	Homo sapiens solute carrier family 16, member 3 (monocarboxylic acid transporter 4) (SLC16A3), transcript variant 2, mRNA [NM_004207]
2,51	2,33	IER3	NM_003897	Homo sapiens immediate early response 3 (IER3), mRNA [NM_003897]
2,50	2,98	NUAK1	NM_014840	Homo sapiens NUAK family, SNF1-like kinase, 1 (NUAK1), mRNA [NM_014840]
2,48	2,31	HAAO	NM_012205	Homo sapiens 3-hydroxyanthranilate 3,4-dioxygenase (HAAO), mRNA [NM_012205]
2,47	3,98	TGFB2	ENST00000366930	Transforming growth factor beta-2 precursor (TGF-beta-2) (Glioblastoma-derived T-cell suppressor factor) (G-TSF) (BSC-1 cell growth inhibitor) (Polyergin) (Cetermin). [Source:Uniprot/SWISSPROT;Acc:P61812] [ENST00000366930]
2,45	2,08	BHLHB2	NM_003670	Homo sapiens basic helix-loop-helix domain containing, class B, 2 (BHLHB2), mRNA [NM_003670]
2,43	2,01	TMEPAI	NM_020182	Homo sapiens transmembrane, prostate androgen induced RNA (TMEPAI), transcript variant 1, mRNA [NM_020182]
2,42	2,32	NT5E	NM_002526	Homo sapiens 5'-nucleotidase, ecto (CD73) (NT5E), mRNA [NM_002526]
2,37	3,27	KIAA1462	ENST00000375377	Novel protein. [Source:Uniprot/SPTREMBL;Acc:Q5T992] [ENST00000375377]
2,35	3,66	SPOCD1	NM_144569	Homo sapiens SPOC domain containing 1 (SPOCD1), mRNA [NM_144569]
2,33	6,88	ACTG2	NM_001615	Homo sapiens actin, gamma 2, smooth muscle, enteric (ACTG2), mRNA [NM_001615]
2,33	2,26	HSD17B3	NM_000197	Homo sapiens hydroxysteroid (17-beta) dehydrogenase 3 (HSD17B3), mRNA [NM_000197]
2,33	2,28	MMD	NM_012329	Homo sapiens monocyte to macrophage differentiation-associated (MMD), mRNA [NM_012329]

2,32	2,01	KIF26B	NM_018012	Homo sapiens kinesin family member 26B (KIF26B), mRNA [NM_018012]
2,32	2,83	IL4I1	NM_172374	Homo sapiens interleukin 4 induced 1 (IL4I1), transcript variant 2, mRNA [NM_172374]
2,30	5,74	HSD17B6	NM_003725	Homo sapiens hydroxysteroid (17-beta) dehydrogenase 6 homolog (mouse) (HSD17B6), mRNA [NM_003725]
2,30	5,17	PBEF1	NM_005746	Homo sapiens pre-B-cell colony enhancing factor 1 (PBEF1), mRNA [NM_005746]
2,24	2,35	COL13A1	NM_005203	Homo sapiens collagen, type XIII, alpha 1 (COL13A1), transcript variant 1, mRNA [NM_005203]
2,23	2,38	TPST1	NM_003596	Homo sapiens tyrosylprotein sulfotransferase 1 (TPST1), mRNA [NM_003596]
2,23	2,83	SLC19A2	NM_006996	Homo sapiens solute carrier family 19 (thiamine transporter), member 2 (SLC19A2), mRNA [NM_006996]
2,22	3,11	NRIP1	NM_003489	Homo sapiens nuclear receptor interacting protein 1 (NRIP1), mRNA [NM_003489]
2,21	2,03	PRICKLE1	NM_153026	Homo sapiens prickle homolog 1 (Drosophila) (PRICKLE1), mRNA [NM_153026]
2,20	2,02	IL1A	NM_000575	Homo sapiens interleukin 1, alpha (IL1A), mRNA [NM_000575]
2,20	2,33	RUNX1	NM_001001890	Homo sapiens runt-related transcription factor 1 (acute myeloid leukemia 1; aml1 oncogene) (RUNX1), transcript variant 2, mRNA [NM_001001890]
2,20	2,57	HIF1A	NM_181054	Homo sapiens hypoxia-inducible factor 1, alpha subunit (basic helix-loop-helix transcription factor) (HIF1A), transcript variant 2, mRNA [NM_181054]
2,20	2,40	FAM46A	NM_017633	Homo sapiens family with sequence similarity 46, member A (FAM46A), mRNA [NM_017633]
2,20	2,48	BHLHB3	NM_030762	Homo sapiens basic helix-loop-helix domain containing, class B, 3 (BHLHB3), mRNA [NM_030762]
2,18	2,37	PFKP	NM_002627	Homo sapiens phosphofructokinase, platelet (PFKP), mRNA [NM_002627]
2,17	3,22	ASNS	NM_001673	Homo sapiens asparagine synthetase (ASNS), transcript variant 2, mRNA [NM_001673]
2,16	2,54	SRPX	NM_006307	Homo sapiens sushi-repeat-containing protein, X-linked (SRPX), mRNA [NM_006307]
2,16	3,18	EGF	NM_001963	Homo sapiens epidermal growth factor (beta-urogastrone) (EGF), mRNA [NM_001963]
2,15	2,29	ENST00000229270	ENST00000229270	Triosephosphate isomerase (EC 5.3.1.1) (TIM) (Triose-phosphate isomerase). [Source:Uniprot/SWISSPROT;Acc:P60174] [ENST00000229270]
2,15	2,35	CDR2	NM_001802	Homo sapiens cerebellar degeneration-related protein 2, 62kDa (CDR2), mRNA [NM_001802]
2,15	2,42	ITGB3	S70348	Homo sapiens integrin beta 3 mRNA, partial cds, alternatively spliced. [S70348]

2,13	2,47	AF126109	AF126109	Homo sapiens clone TA40 untranslated mRNA, complete sequence. [AF126109]
2,12	2,16	KCNG1	NM_002237	Homo sapiens potassium voltage-gated channel, subfamily G, member 1 (KCNG1), transcript variant 1, mRNA [NM_002237]
2,12	2,19	YIPF5	NM_030799	Homo sapiens Yip1 domain family, member 5 (YIPF5), transcript variant 2, mRNA [NM_030799]
2,11	3,03	BNIP3	NM_004052	Homo sapiens BCL2/adenovirus E1B 19kDa interacting protein 3 (BNIP3), nuclear gene encoding mitochondrial protein, mRNA [NM_004052]
2,11	3,72	GBE1	NM_000158	Homo sapiens glucan (1,4-alpha-), branching enzyme 1 (glycogen branching enzyme, Andersen disease, glycogen storage disease type IV) (GBE1), mRNA [NM_000158]
2,10	4,95	PBEF1	NM_182790	Homo sapiens pre-B-cell colony enhancing factor 1 (PBEF1), transcript variant 2, mRNA [NM_182790]
2,09	2,12	ADAM12	NM_021641	Homo sapiens ADAM metallopeptidase domain 12 (meltrin alpha) (ADAM12), transcript variant 2, mRNA [NM_021641]
2,08	2,22	PDGFA	NM_002607	Homo sapiens platelet-derived growth factor alpha polypeptide (PDGFA), transcript variant 1, mRNA [NM_002607]
2,07	2,08	BMP2	NM_001200	Homo sapiens bone morphogenetic protein 2 (BMP2), mRNA [NM_001200]
2,06	2,23	CNN1	NM_001299	Homo sapiens calponin 1, basic, smooth muscle (CNN1), mRNA [NM_001299]
2,06	2,33	ST6GALNAC5	NM_030965	Homo sapiens ST6 (alpha-N-acetyl-neuraminyl-2,3-beta-galactosyl-1,3)-N-acetylgalactosaminide alpha-2,6-sialyltransferase 5 (ST6GALNAC5), mRNA [NM_030965]
2,04	2,08	PPFIA4	NM_015053	Homo sapiens protein tyrosine phosphatase, receptor type, f polypeptide (PTPRF), interacting protein (liprin), alpha 4 (PPFIA4), mRNA [NM_015053]
2,02	2,17	RAB23	NM_016277	Homo sapiens RAB23, member RAS oncogene family (RAB23), transcript variant 1, mRNA [NM_016277]
2,00	2,05	SMYD3	NM_022743	Homo sapiens SET and MYND domain containing 3 (SMYD3), mRNA [NM_022743]

Supplementary Table S2. Microarray repressed genes in vitro.

Omentum A	Omentum C	Gene Symbol	Systematic Name	Description
0,50	0,40	LAMA3	NM_198129	Homo sapiens laminin, alpha 3 (LAMA3), transcript variant 1, mRNA [NM_198129]
0,50	0,41	ADRA1B	NM_000679	Homo sapiens adrenergic, alpha-1B-, receptor (ADRA1B), mRNA [NM_000679]
0,49	0,41	ALDH3B1	NM_000694	Homo sapiens aldehyde dehydrogenase 3 family, member B1 (ALDH3B1), transcript variant 1, mRNA [NM_000694]
0,49	0,29	APOA1	NM_000039	Homo sapiens apolipoprotein A-I (APOA1), mRNA [NM_000039]
0,49	0,48	RAD54L	NM_003579	Homo sapiens RAD54-like (S. cerevisiae) (RAD54L), mRNA [NM_003579]
0,49	0,50	SLAIN1	NM_144595	Homo sapiens SLAIN motif family, member 1 (SLAIN1), transcript variant 2, mRNA [NM_144595]
0,49	0,33	NPHS1	NM_004646	Homo sapiens nephrosis 1, congenital, Finnish type (nephrin) (NPHS1), mRNA [NM_004646]
0,49	0,44	CDKN2C	NM_001262	Homo sapiens cyclin-dependent kinase inhibitor 2C (p18, inhibits CDK4) (CDKN2C), transcript variant 1, mRNA [NM_001262]
0,48	0,42	DKFZp761P0423	ENST00000330777	Tyrosine-protein kinase SgK223 (EC 2.7.10.2) (Sugen kinase 223). [Source:Uniprot/SWISSPROT;Acc:Q86YV5] [ENST00000330777]
0,48	0,49	EXO1	NM_003686	Homo sapiens exonuclease 1 (EXO1), transcript variant 3, mRNA [NM_003686]
0,48	0,48	SMARCD3	NM_003078	Homo sapiens SWI/SNF related, matrix associated, actin dependent regulator of chromatin, subfamily d, member 3 (SMARCD3), transcript variant 2, mRNA [NM_003078]
0,48	0,42	KIF11	NM_004523	Homo sapiens kinesin family member 11 (KIF11), mRNA [NM_004523]
0,48	0,41	SLC9A3R1	NM_004252	Homo sapiens solute carrier family 9 (sodium/hydrogen exchanger), member 3 regulator 1 (SLC9A3R1), mRNA [NM_004252]
0,48	0,39	TMEM37	NM_183240	Homo sapiens transmembrane protein 37 (TMEM37), mRNA [NM_183240]
0,48	0,37	MID1	NM_033290	Homo sapiens midline 1 (Opitz/BBB syndrome) (MID1), transcript variant 3, mRNA [NM_033290]
0,48	0,45	KCNQ1	NM_000218	Homo sapiens potassium voltage-gated channel, KQT-like subfamily, member 1 (KCNQ1), transcript variant 1, mRNA [NM_000218]
0,48	0,48	PARD6B	NM_032521	Homo sapiens par-6 partitioning defective 6 homolog beta (C. elegans) (PARD6B), mRNA [NM_032521]
0,48	0,35	DFNA5	NM_004403	Homo sapiens deafness, autosomal dominant 5 (DFNA5), mRNA [NM_004403]
0,47	0,48	HBD	NM_000519	Homo sapiens hemoglobin, delta (HBD), mRNA [NM_000519]

0,47	0,33	PSG6	NM_002782	Homo sapiens pregnancy specific beta-1-glycoprotein 6 (PSG6), transcript variant 1, mRNA [NM_002782]
0,47	0,43	MCM3	NM_002388	Homo sapiens MCM3 minichromosome maintenance deficient 3 (S. cerevisiae) (MCM3), mRNA [NM_002388]
0,47	0,42	SLC27A2	NM_003645	Homo sapiens solute carrier family 27 (fatty acid transporter), member 2 (SLC27A2), mRNA [NM_003645]
0,47	0,50	CENPM	NM_001002876	Homo sapiens centromere protein M (CENPM), transcript variant 2, mRNA [NM_001002876]
0,47	0,41	CENPA	NM_001809	Homo sapiens centromere protein A (CENPA), transcript variant 1, mRNA [NM_001809]
0,47	0,43	DTL	NM_016448	Homo sapiens denticleless homolog (Drosophila) (DTL), mRNA [NM_016448]
0,47	0,32	C4B	NM_001002029	Homo sapiens complement component 4B (Childo blood group) (C4B), mRNA [NM_001002029]
0,47	0,30	AIF1	NM_004847	Homo sapiens allograft inflammatory factor 1 (AIF1), transcript variant 2, mRNA [NM_004847]
0,46	0,47	KIF4A	NM_012310	Homo sapiens kinesin family member 4A (KIF4A), mRNA [NM_012310]
0,46	0,45	CTSH	NM_148979	Homo sapiens cathepsin H (CTSH), transcript variant 2, mRNA [NM_148979]
0,46	0,39	H2AFX	NM_002105	Homo sapiens H2A histone family, member X (H2AFX), mRNA [NM_002105]
0,46	0,46	KIF23	NM_138555	Homo sapiens kinesin family member 23 (KIF23), transcript variant 1, mRNA [NM_138555]
0,46	0,47	PDGFD	NM_025208	Homo sapiens platelet derived growth factor D (PDGFD), transcript variant 1, mRNA [NM_025208]
0,46	0,36	MAF	AF055376	Homo sapiens short form transcription factor C-MAF (c-maf) mRNA, complete cds. [AF055376]
0,46	0,34	CD248	NM_020404	Homo sapiens CD248 molecule, endosialin (CD248), mRNA [NM_020404]
0,45	0,45	SPC25	NM_020675	Homo sapiens SPC25, NDC80 kinetochore complex component, homolog (S. cerevisiae) (SPC25), mRNA [NM_020675]
0,45	0,47	ITPR1	NM_002222	Homo sapiens inositol 1,4,5-triphosphate receptor, type 1 (ITPR1), mRNA [NM_002222]
0,45	0,41	TNFRSF11B	NM_002546	Homo sapiens tumor necrosis factor receptor superfamily, member 11b (osteoprotegerin) (TNFRSF11B), mRNA [NM_002546]
0,45	0,44	LATS2	NM_014572	Homo sapiens LATS, large tumor suppressor, homolog 2 (Drosophila) (LATS2), mRNA [NM_014572]
0,45	0,37	GPRC5A	NM_003979	Homo sapiens G protein-coupled receptor, family C, group 5, member A (GPRC5A), mRNA [NM_003979]
0,45	0,39	SERPINB7	NM_003784	Homo sapiens serpin peptidase inhibitor, clade B (ovalbumin), member 7 (SERPINB7), transcript variant 1, mRNA [NM_003784]
0,45	0,49	ITIH3	NM_002217	Homo sapiens inter-alpha (globulin) inhibitor H3 (ITIH3), mRNA [NM_002217]

0,45	0,34	MCM5	NM_006739	Homo sapiens MCM5 minichromosome maintenance deficient 5, cell division cycle 46 (S. cerevisiae) (MCM5), mRNA [NM_006739]
0,44	0,43	ASF1B	NM_018154	Homo sapiens ASF1 anti-silencing function 1 homolog B (S. cerevisiae) (ASF1B), mRNA [NM_018154]
0,44	0,48	MMP23B	NM_006983	Homo sapiens matrix metallopeptidase 23B (MMP23B), mRNA [NM_006983]
0,44	0,40	RAD51AP1	NM_006479	Homo sapiens RAD51 associated protein 1 (RAD51AP1), mRNA [NM_006479]
0,44	0,39	SPINT2	NM_021102	Homo sapiens serine peptidase inhibitor, Kunitz type, 2 (SPINT2), mRNA [NM_021102]
0,44	0,34	KCNA4	NM_002233	Homo sapiens potassium voltage-gated channel, shaker-related subfamily, member 4 (KCNA4), mRNA [NM_002233]
0,44	0,28	SDPR	NM_004657	Homo sapiens serum deprivation response (phosphatidylserine binding protein) (SDPR), mRNA [NM_004657]
0,43	0,39	SLC16A4	NM_004696	Homo sapiens solute carrier family 16, member 4 (monocarboxylic acid transporter 5) (SLC16A4), mRNA [NM_004696]
0,43	0,35	PTPN13	NM_080685	Homo sapiens protein tyrosine phosphatase, non-receptor type 13 (APO-1/CD95 (Fas)-associated phosphatase) (PTPN13), transcript variant 4, mRNA [NM_080685]
0,43	0,44	AURKA	NM_198433	Homo sapiens aurora kinase A (AURKA), transcript variant 1, mRNA [NM_198433]
0,43	0,28	NLRP1	BC051787	Homo sapiens NLR family, pyrin domain containing 1, mRNA (cDNA clone MGC:57544 IMAGE:5756099), complete cds. [BC051787]
0,43	0,40	MCM7	NM_182776	Homo sapiens MCM7 minichromosome maintenance deficient 7 (S. cerevisiae) (MCM7), transcript variant 2, mRNA [NM_182776]
0,42	0,49	APOBEC3D	NM_152426	Homo sapiens apolipoprotein B mRNA editing enzyme, catalytic polypeptide-like 3D (putative) (APOBEC3D), mRNA [NM_152426]
0,42	0,46	TROAP	NM_005480	Homo sapiens trophinin associated protein (tastin) (TROAP), mRNA [NM_005480]
0,42	0,38	ADRA2A	NM_000681	Homo sapiens adrenergic, alpha-2A-, receptor (ADRA2A), mRNA [NM_000681]
0,42	0,22	DES	NM_001927	Homo sapiens desmin (DES), mRNA [NM_001927]
0,42	0,47	CPA1	NM_001868	Homo sapiens carboxypeptidase A1 (pancreatic) (CPA1), mRNA [NM_001868]
0,42	0,29	FAM110B	NM_147189	Homo sapiens family with sequence similarity 110, member B (FAM110B), mRNA [NM_147189]
0,42	0,36	ITGA7	NM_002206	Homo sapiens integrin, alpha 7 (ITGA7), mRNA [NM_002206]
0,42	0,48	PHF19	NM_001009936	Homo sapiens PHD finger protein 19 (PHF19), transcript variant 2, mRNA [NM_001009936]
0,42	0,48	EPS8	NM_004447	Homo sapiens epidermal growth factor receptor pathway substrate 8 (EPS8), mRNA [NM_004447]

0,41	0,47	ALDH8A1	NM_022568	Homo sapiens aldehyde dehydrogenase 8 family, member A1 (ALDH8A1), transcript variant 1, mRNA [NM_022568]
0,41	0,46	DISP1	NM_032890	Homo sapiens dispatched homolog 1 (Drosophila) (DISP1), mRNA [NM_032890]
0,41	0,45	MELK	NM_014791	Homo sapiens maternal embryonic leucine zipper kinase (MELK), mRNA [NM_014791]
0,41	0,28	CFB	NM_001710	Homo sapiens complement factor B (CFB), mRNA [NM_001710]
0,41	0,42	CDCA2	NM_152562	Homo sapiens cell division cycle associated 2 (CDCA2), mRNA [NM_152562]
0,41	0,42	MCM6	NM_005915	Homo sapiens minichromosome maintenance deficient 6 homolog (S. cerevisiae) (MCM6), mRNA [NM_005915]
0,41	0,38	FOXM1	NM_202002	Homo sapiens forkhead box M1 (FOXM1), transcript variant 1, mRNA [NM_202002]
0,41	0,15	UPK3B	NM_030570	Homo sapiens uroplakin 3B (UPK3B), transcript variant 1, mRNA [NM_030570]
0,41	0,36	BUB1B	NM_001211	Homo sapiens BUB1 budding uninhibited by benzimidazoles 1 homolog beta (yeast) (BUB1B), mRNA [NM_001211]
0,41	0,35	ALDH3A2	NM_000382	Homo sapiens aldehyde dehydrogenase 3 family, member A2 (ALDH3A2), transcript variant 2, mRNA [NM_000382]
0,41	0,44	MST1	NM_020998	Homo sapiens macrophage stimulating 1 (hepatocyte growth factor-like) (MST1), mRNA [NM_020998]
0,41	0,34	HPD	NM_002150	Homo sapiens 4-hydroxyphenylpyruvate dioxygenase (HPD), mRNA [NM_002150]
0,41	0,43	FCRLB	NM_001002901	Homo sapiens Fc receptor-like B (FCRLB), mRNA [NM_001002901]
0,40	0,30	SLC40A1	NM_014585	Homo sapiens solute carrier family 40 (iron-regulated transporter), member 1 (SLC40A1), mRNA [NM_014585]
0,40	0,38	TSPAN3	NM_005724	Homo sapiens tetraspanin 3 (TSPAN3), transcript variant 1, mRNA [NM_005724]
0,40	0,39	ID3	NM_002167	Homo sapiens inhibitor of DNA binding 3, dominant negative helix-loop-helix protein (ID3), mRNA [NM_002167]
0,40	0,48	TMPRSS3	NM_032405	Homo sapiens transmembrane protease, serine 3 (TMPRSS3), transcript variant D, mRNA [NM_032405]
0,40	0,26	BMP4	NM_001202	Homo sapiens bone morphogenetic protein 4 (BMP4), transcript variant 1, mRNA [NM_001202]
0,40	0,40	RAB11FIP1	NM_001002233	Homo sapiens RAB11 family interacting protein 1 (class I) (RAB11FIP1), transcript variant 2, mRNA [NM_001002233]
0,40	0,35	IGSF9	NM_020789	Homo sapiens immunoglobulin superfamily, member 9 (IGSF9), mRNA [NM_020789]
0,40	0,50	A2M	NM_000014	Homo sapiens alpha-2-macroglobulin (A2M), mRNA [NM_000014]
0,40	0,44	CXCR7	NM_020311	Homo sapiens chemokine (C-X-C motif) receptor 7 (CXCR7), transcript variant 2, mRNA [NM_020311]

0,39	0,43	THC2603259	THC2603259	Q96IM5_HUMAN (Q96IM5) RAB7B protein, complete [THC2603259]
0,39	0,45	NUF2	NM_145697	Homo sapiens NUF2, NDC80 kinetochore complex component, homolog (S. cerevisiae) (NUF2), transcript variant 1, mRNA [NM_145697]
0,39	0,41	GAL3ST1	NM_004861	Homo sapiens galactose-3-O-sulfotransferase 1 (GAL3ST1), mRNA [NM_004861]
0,39	0,43	ANXA3	NM_005139	Homo sapiens annexin A3 (ANXA3), mRNA [NM_005139]
0,39	0,45	SPANXA1	NM_013453	Homo sapiens sperm protein associated with the nucleus, X-linked, family member A1 (SPANXA1), mRNA [NM_013453]
0,39	0,31	ATP7B	NM_000053	Homo sapiens ATPase, Cu++ transporting, beta polypeptide (ATP7B), transcript variant 1, mRNA [NM_000053]
0,39	0,32	NDRG4	NM_022910	Homo sapiens NDRG family member 4 (NDRG4), mRNA [NM_022910]
0,39	0,39	DIAPH3	NM_030932	Homo sapiens diaphanous homolog 3 (Drosophila) (DIAPH3), transcript variant 2, mRNA [NM_030932]
0,38	0,30	LRP2	NM_004525	Homo sapiens low density lipoprotein-related protein 2 (LRP2), mRNA [NM_004525]
0,38	0,40	AP1S3	NM_001039569	Homo sapiens adaptor-related protein complex 1, sigma 3 subunit (AP1S3), mRNA [NM_001039569]
0,38	0,38	E2F1	NM_005225	Homo sapiens E2F transcription factor 1 (E2F1), mRNA [NM_005225]
0,38	0,19	AQP1	NM_198098	Homo sapiens aquaporin 1 (Colton blood group) (AQP1), mRNA [NM_198098]
0,38	0,40	ADM	NM_001124	Homo sapiens adrenomedullin (ADM), mRNA [NM_001124]
0,38	0,37	CDT1	NM_030928	Homo sapiens chromatin licensing and DNA replication factor 1 (CDT1), mRNA [NM_030928]
0,38	0,44	VCAM1	NM_001078	Homo sapiens vascular cell adhesion molecule 1 (VCAM1), transcript variant 1, mRNA [NM_001078]
0,38	0,37	PLEKHG4	NM_015432	Homo sapiens pleckstrin homology domain containing, family G (with RhoGef domain) member 4 (PLEKHG4), mRNA [NM_015432]
0,38	0,38	TTK	NM_003318	Homo sapiens TTK protein kinase (TTK), mRNA [NM_003318]
0,38	0,47	CAND2	NM_012298	Homo sapiens cullin-associated and neddylation-dissociated 2 (putative) (CAND2), mRNA [NM_012298]
0,37	0,29	NID2	NM_007361	Homo sapiens nidogen 2 (osteonidogen) (NID2), mRNA [NM_007361]
0,37	0,40	FAM102A	NM_203305	Homo sapiens family with sequence similarity 102, member A (FAM102A), transcript variant 2, mRNA [NM_203305]
0,37	0,32	MUC16	NM_024690	Homo sapiens mucin 16, cell surface associated (MUC16), mRNA [NM_024690]
0,37	0,35	LAYN	NM_178834	Homo sapiens layilin (LAYN), mRNA [NM_178834]

0,37	0,28	ENPP1	ENST00000367994	Ectonucleotide pyrophosphatase/phosphodiesterase 1 (E-NPP 1) (Phosphodiesterase I/nucleotide pyrophosphatase 1) (Plasma-cell membrane glycoprotein PC-1) [Includes: Alkaline phosphodiesterase I (EC 3.1.4.1); Nucleotide pyrophosphatase (EC 3.6.1.9)...
0,37	0,50	MAD2L1	NM_002358	Homo sapiens MAD2 mitotic arrest deficient-like 1 (yeast) (MAD2L1), mRNA [NM_002358]
0,37	0,30	KLK11	NM_144947	Homo sapiens kallikrein-related peptidase 11 (KLK11), transcript variant 2, mRNA [NM_144947]
0,36	0,47	KCNJ8	NM_004982	Homo sapiens potassium inwardly-rectifying channel, subfamily J, member 8 (KCNJ8), mRNA [NM_004982]
0,36	0,42	REEP1	NM_022912	Homo sapiens receptor accessory protein 1 (REEP1), mRNA [NM_022912]
0,36	0,48	AF086124	AF086124	Homo sapiens full length insert cDNA clone ZA79C08. [AF086124]
0,36	0,35	MAL2	NM_052886	Homo sapiens mal, T-cell differentiation protein 2 (MAL2), mRNA [NM_052886]
0,36	0,32	FRY	NM_023037	Homo sapiens furry homolog (Drosophila) (FRY), mRNA [NM_023037]
0,36	0,49	FAM119A	NM_145280	Homo sapiens family with sequence similarity 119, member A (FAM119A), mRNA [NM_145280]
0,36	0,37	SPOCK2	NM_014767	Homo sapiens sparc/osteonectin, cwcv and kazal-like domains proteoglycan (testican) 2 (SPOCK2), mRNA [NM_014767]
0,36	0,46	HPR	NM_020995	Homo sapiens haptoglobin-related protein (HPR), mRNA [NM_020995]
0,36	0,38	OLFML1	NM_198474	Homo sapiens olfactomedin-like 1 (OLFML1), mRNA [NM_198474]
0,36	0,41	ITLN2	NM_080878	Homo sapiens intelectin 2 (ITLN2), mRNA [NM_080878]
0,35	0,23	ZFP36L2	NM_006887	Homo sapiens zinc finger protein 36, C3H type-like 2 (ZFP36L2), mRNA [NM_006887]
0,35	0,36	NDC80	NM_006101	Homo sapiens NDC80 homolog, kinetochore complex component (S. cerevisiae) (NDC80), mRNA [NM_006101]
0,35	0,34	CCNA2	NM_001237	Homo sapiens cyclin A2 (CCNA2), mRNA [NM_001237]
0,35	0,40	PYGM	ENST00000377444	Glycogen phosphorylase, muscle form (EC 2.4.1.1) (Myophosphorylase). [Source:Uniprot/SWISSPROT;Acc:P11217] [ENST00000377444]
0,35	0,24	CRISPLD1	NM_031461	Homo sapiens cysteine-rich secretory protein LCCL domain containing 1 (CRISPLD1), mRNA [NM_031461]
0,35	0,39	SELENBP1	NM_003944	Homo sapiens selenium binding protein 1 (SELENBP1), mRNA [NM_003944]
0,35	0,40	DIAPH3	NM_001042517	Homo sapiens diaphanous homolog 3 (Drosophila) (DIAPH3), transcript variant 1, mRNA [NM_001042517]
0,35	0,46	MT1JP	AF348994	Homo sapiens MTB (MTB) mRNA, complete cds. [AF348994]
0,35	0,30	LMNB1	NM_005573	Homo sapiens lamin B1 (LMNB1), mRNA [NM_005573]
0,34	0,45	TMEM14B	NM_030969	Homo sapiens transmembrane protein 14B (TMEM14B), mRNA [NM_030969]
0,34	0,32	EMILIN2	NM_032048	Homo sapiens elastin microfibril interfacer 2 (EMILIN2), mRNA [NM_032048]

0,34	0,31	SHCBP1	NM_024745	Homo sapiens SHC SH2-domain binding protein 1 (SHCBP1), mRNA [NM_024745]
0,34	0,29	MARCO	NM_006770	Homo sapiens macrophage receptor with collagenous structure (MARCO), mRNA [NM_006770]
0,34	0,46	SGEF	AB073386	Homo sapiens infant liver cDNA, clone:HMFN1864, full insert sequence. [AB073386]
0,34	0,39	RAD51	NM_002875	Homo sapiens RAD51 homolog (RecA homolog, E. coli) (S. cerevisiae) (RAD51), transcript variant 1, mRNA [NM_002875]
0,34	0,43	LAT2	NM_032463	Homo sapiens linker for activation of T cells family, member 2 (LAT2), transcript variant 2, mRNA [NM_032463]
0,34	0,34	KIFC1	NM_002263	Homo sapiens kinesin family member C1 (KIFC1), mRNA [NM_002263]
0,34	0,44	SLC13A3	NM_001011554	Homo sapiens solute carrier family 13 (sodium-dependent dicarboxylate transporter), member 3 (SLC13A3), transcript variant 2, mRNA [NM_001011554]
0,34	0,26	VTN	NM_000638	Homo sapiens vitronectin (VTN), mRNA [NM_000638]
0,34	0,16	TXNIP	NM_006472	Homo sapiens thioredoxin interacting protein (TXNIP), mRNA [NM_006472]
0,34	0,40	ITM2A	NM_004867	Homo sapiens integral membrane protein 2A (ITM2A), mRNA [NM_004867]
0,34	0,42	EFEMP1	NM_004105	Homo sapiens EGF-containing fibulin-like extracellular matrix protein 1 (EFEMP1), transcript variant 1, mRNA [NM_004105]
0,34	0,42	AP1S3	BC021898	Homo sapiens adaptor-related protein complex 1, sigma 3 subunit, mRNA (cDNA clone MGC:17284 IMAGE:4340257), complete cds. [BC021898]
0,33	0,33	SYNPO2L	NM_024875	Homo sapiens synaptopodin 2-like (SYNPO2L), mRNA [NM_024875]
0,33	0,47	ANKRD35	NM_144698	Homo sapiens ankyrin repeat domain 35 (ANKRD35), mRNA [NM_144698]
0,33	0,31	CDC20	NM_001255	Homo sapiens cell division cycle 20 homolog (S. cerevisiae) (CDC20), mRNA [NM_001255]
0,33	0,48	GLRX	NM_002064	Homo sapiens glutaredoxin (thioltransferase) (GLRX), mRNA [NM_002064]
0,33	0,26	LITAF	NM_004862	Homo sapiens lipopolysaccharide-induced TNF factor (LITAF), mRNA [NM_004862]
0,33	0,31	SEMA5A	ENST00000382496	Semaphorin-5A precursor (Semaphorin F) (Sema F). [Source:Uniprot/SWISSPROT;Acc:Q13591] [ENST00000382496]
0,33	0,38	FLRT3	NM_198391	Homo sapiens fibronectin leucine rich transmembrane protein 3 (FLRT3), transcript variant 2, mRNA [NM_198391]
0,32	0,34	CDCA5	NM_080668	Homo sapiens cell division cycle associated 5 (CDCA5), mRNA [NM_080668]
0,32	0,31	NUSAP1	NM_016359	Homo sapiens nucleolar and spindle associated protein 1 (NUSAP1), transcript variant 1, mRNA [NM_016359]
0,32	0,40	RRM1	NM_001033	Homo sapiens ribonucleotide reductase M1 polypeptide (RRM1), mRNA [NM_001033]

0,32	0,24	TPX2	NM_012112	Homo sapiens TPX2, microtubule-associated, homolog (Xenopus laevis) (TPX2), mRNA [NM_012112]
0,32	0,47	MT1M	NM_176870	Homo sapiens metallothionein 1M (MT1M), mRNA [NM_176870]
0,32	0,30	BUB1	NM_004336	Homo sapiens BUB1 budding uninhibited by benzimidazoles 1 homolog (yeast) (BUB1), mRNA [NM_004336]
0,32	0,31	SASH1	NM_015278	Homo sapiens SAM and SH3 domain containing 1 (SASH1), mRNA [NM_015278]
0,32	0,27	ABLIM2	NM_032432	Homo sapiens actin binding LIM protein family, member 2 (ABLIM2), mRNA [NM_032432]
0,31	0,29	KCNK5	NM_003740	Homo sapiens potassium channel, subfamily K, member 5 (KCNK5), mRNA [NM_003740]
0,31	0,50	HTRA4	NM_153692	Homo sapiens HtrA serine peptidase 4 (HTRA4), mRNA [NM_153692]
0,31	0,36	NCAPH	NM_015341	Homo sapiens non-SMC condensin I complex, subunit H (NCAPH), mRNA [NM_015341]
0,31	0,27	TJP2	NM_004817	Homo sapiens tight junction protein 2 (zona occludens 2) (TJP2), transcript variant 1, mRNA [NM_004817]
0,31	0,47	LONRF2	NM_198461	Homo sapiens LON peptidase N-terminal domain and ring finger 2 (LONRF2), mRNA [NM_198461]
0,31	0,30	AOX1	NM_001159	Homo sapiens aldehyde oxidase 1 (AOX1), mRNA [NM_001159]
0,31	0,33	CDC6	NM_001254	Homo sapiens cell division cycle 6 homolog (S. cerevisiae) (CDC6), mRNA [NM_001254]
0,31	0,33	ENPP1	NM_006208	Homo sapiens ectonucleotide pyrophosphatase/phosphodiesterase 1 (ENPP1), mRNA [NM_006208]
0,30	0,31	STMN1	NM_203401	Homo sapiens stathmin 1/oncoprotein 18 (STMN1), transcript variant 1, mRNA [NM_203401]
0,30	0,38	OLR1	NM_002543	Homo sapiens oxidized low density lipoprotein (lectin-like) receptor 1 (OLR1), mRNA [NM_002543]
0,30	0,43	IL18	NM_001562	Homo sapiens interleukin 18 (interferon-gamma-inducing factor) (IL18), mRNA [NM_001562]
0,30	0,33	FAM64A	NM_019013	Homo sapiens family with sequence similarity 64, member A (FAM64A), mRNA [NM_019013]
0,30	0,26	FGF9	NM_002010	Homo sapiens fibroblast growth factor 9 (glia-activating factor) (FGF9), mRNA [NM_002010]
0,30	0,46	TESC	NM_017899	Homo sapiens tescalcin (TESC), mRNA [NM_017899]
0,29	0,27	TYMS	NM_001071	Homo sapiens thymidylate synthetase (TYMS), mRNA [NM_001071]
0,29	0,33	NMU	NM_006681	Homo sapiens neuromedin U (NMU), mRNA [NM_006681]
0,29	0,29	FAM83D	NM_030919	Homo sapiens family with sequence similarity 83, member D (FAM83D), mRNA [NM_030919]

0,29	0,35	CDC45L	NM_003504	Homo sapiens CDC45 cell division cycle 45-like (S. cerevisiae) (CDC45L), mRNA [NM_003504]
0,29	0,25	TSPAN7	NM_004615	Homo sapiens tetraspanin 7 (TSPAN7), mRNA [NM_004615]
0,29	0,30	SLC39A8	NM_022154	Homo sapiens solute carrier family 39 (zinc transporter), member 8 (SLC39A8), mRNA [NM_022154]
0,29	0,29	PKMYT1	NM_182687	Homo sapiens protein kinase, membrane associated tyrosine/threonine 1 (PKMYT1), transcript variant 2, mRNA [NM_182687]
0,29	0,28	DAB2	NM_001343	Homo sapiens disabled homolog 2, mitogen-responsive phosphoprotein (Drosophila) (DAB2), mRNA [NM_001343]
0,28	0,27	GINS2	NM_016095	Homo sapiens GINS complex subunit 2 (Psf2 homolog) (GINS2), mRNA [NM_016095]
0,28	0,24	OGDHL	NM_018245	Homo sapiens oxoglutarate dehydrogenase-like (OGDHL), mRNA [NM_018245]
0,28	0,43	CD38	NM_001775	Homo sapiens CD38 molecule (CD38), mRNA [NM_001775]
0,28	0,38	PTTG2	NM_006607	Homo sapiens pituitary tumor-transforming 2 (PTTG2), mRNA [NM_006607]
0,28	0,28	SFRP4	NM_003014	Homo sapiens secreted frizzled-related protein 4 (SFRP4), mRNA [NM_003014]
0,27	0,36	ASPM	NM_018136	Homo sapiens asp (abnormal spindle) homolog, microcephaly associated (Drosophila) (ASPM), mRNA [NM_018136]
0,27	0,34	HMGB2	NM_002129	Homo sapiens high-mobility group box 2 (HMGB2), mRNA [NM_002129]
0,27	0,36	KIAA0746	NM_015187	Homo sapiens KIAA0746 protein (KIAA0746), mRNA [NM_015187]
0,27	0,29	CAPN6	NM_014289	Homo sapiens calpain 6 (CAPN6), mRNA [NM_014289]
0,27	0,28	CIT	NM_007174	Homo sapiens citron (rho-interacting, serine/threonine kinase 21) (CIT), mRNA [NM_007174]
0,27	0,37	ENST00000320378	ENST00000320378	Epithelial membrane protein 2 (EMP-2) (Protein XMP). [Source:Uniprot/SWISSPROT;Acc:P54851] [ENST00000320378]
0,27	0,37	SCN4B	NM_174934	Homo sapiens sodium channel, voltage-gated, type IV, beta (SCN4B), mRNA [NM_174934]
0,27	0,26	KIF2C	NM_006845	Homo sapiens kinesin family member 2C (KIF2C), mRNA [NM_006845]
0,27	0,38	MAF	NM_005360	Homo sapiens v-maf musculoaponeurotic fibrosarcoma oncogene homolog (avian) (MAF), transcript variant 1, mRNA [NM_005360]
0,26	0,25	FRAS1	NM_025074	Homo sapiens Fraser syndrome 1 (FRAS1), mRNA [NM_025074]
0,26	0,28	CDKN3	NM_005192	Homo sapiens cyclin-dependent kinase inhibitor 3 (CDK2-associated dual specificity phosphatase) (CDKN3), mRNA [NM_005192]
0,26	0,26	ZWINT	NM_001005414	Homo sapiens ZW10 interactor (ZWINT), transcript variant 4, mRNA [NM_001005414]
0,26	0,36	MLF1IP	NM_024629	Homo sapiens MLF1 interacting protein (MLF1IP), mRNA [NM_024629]
0,26	0,27	SEPP1	NM_005410	Homo sapiens selenoprotein P, plasma, 1 (SEPP1), mRNA [NM_005410]

0,26	0,27	IMPA2	NM_014214	Homo sapiens inositol(myo)-1(or 4)-monophosphatase 2 (IMPA2), mRNA [NM_014214]
0,26	0,27	SPAG5	NM_006461	Homo sapiens sperm associated antigen 5 (SPAG5), mRNA [NM_006461]
0,25	0,35	EMP2	NM_001424	Homo sapiens epithelial membrane protein 2 (EMP2), mRNA [NM_001424]
0,25	0,25	EPS8L1	NM_133180	Homo sapiens EPS8-like 1 (EPS8L1), transcript variant 1, mRNA [NM_133180]
0,25	0,31	CCNB1	NM_031966	Homo sapiens cyclin B1 (CCNB1), mRNA [NM_031966]
0,25	0,26	PRC1	NM_003981	Homo sapiens protein regulator of cytokinesis 1 (PRC1), transcript variant 1, mRNA [NM_003981]
0,25	0,33	OIP5	NM_007280	Homo sapiens Opa interacting protein 5 (OIP5), mRNA [NM_007280]
0,25	0,29	KCNB1	NM_004975	Homo sapiens potassium voltage-gated channel, Shab-related subfamily, member 1 (KCNB1), mRNA [NM_004975]
0,25	0,35	DOK7	NM_173660	Homo sapiens docking protein 7 (DOK7), mRNA [NM_173660]
0,24	0,24	CPA4	NM_016352	Homo sapiens carboxypeptidase A4 (CPA4), mRNA [NM_016352]
0,23	0,26	HMMR	NM_012484	Homo sapiens hyaluronan-mediated motility receptor (RHAMM) (HMMR), transcript variant 1, mRNA [NM_012484]
0,23	0,32	CTNNAL1	NM_003798	Homo sapiens catenin (cadherin-associated protein), alpha-like 1 (CTNNAL1), mRNA [NM_003798]
0,23	0,21	KIF20A	NM_005733	Homo sapiens kinesin family member 20A (KIF20A), mRNA [NM_005733]
0,23	0,20	ITLN1	NM_017625	Homo sapiens intelectin 1 (galactofuranose binding) (ITLN1), mRNA [NM_017625]
0,23	0,29	IGFL2	NM_001002915	Homo sapiens IGF-like family member 2 (IGFL2), mRNA [NM_001002915]
0,23	0,17	SMTNL2	NM_198501	Homo sapiens smoothelin-like 2 (SMTNL2), mRNA [NM_198501]
0,23	0,16	ENPP2	NM_006209	Homo sapiens ectonucleotide pyrophosphatase/phosphodiesterase 2 (autotaxin) (ENPP2), transcript variant 1, mRNA [NM_006209]
0,23	0,20	GAS1	NM_002048	Homo sapiens growth arrest-specific 1 (GAS1), mRNA [NM_002048]
0,22	0,28	PTTG1	NM_004219	Homo sapiens pituitary tumor-transforming 1 (PTTG1), mRNA [NM_004219]
0,21	0,23	CLDN15	NM_014343	Homo sapiens claudin 15 (CLDN15), transcript variant 1, mRNA [NM_014343]
0,21	0,27	CDCA8	NM_018101	Homo sapiens cell division cycle associated 8 (CDCA8), mRNA [NM_018101]
0,21	0,24	PBK	NM_018492	Homo sapiens PDZ binding kinase (PBK), mRNA [NM_018492]
0,21	0,15	KLK5	NM_012427	Homo sapiens kallikrein-related peptidase 5 (KLK5), transcript variant 1, mRNA [NM_012427]
0,21	0,27	TMSL8	NM_021992	Homo sapiens thymosin-like 8 (TMSL8), mRNA [NM_021992]
0,21	0,21	KLK10	NM_002776	Homo sapiens kallikrein-related peptidase 10 (KLK10), transcript variant 1, mRNA [NM_002776]
0,21	0,21	TOP2A	NM_001067	Homo sapiens topoisomerase (DNA) II alpha 170kDa (TOP2A), mRNA [NM_001067]

0,21	0,27	WDR69	NM_178821	Homo sapiens WD repeat domain 69 (WDR69), mRNA [NM_178821]
0,21	0,32	BCHE	NM_000055	Homo sapiens butyrylcholinesterase (BCHE), mRNA [NM_000055]
0,21	0,24	SMOC2	NM_022138	Homo sapiens SPARC related modular calcium binding 2 (SMOC2), mRNA [NM_022138]
0,21	0,23	CST6	NM_001323	Homo sapiens cystatin E/M (CST6), mRNA [NM_001323]
0,21	0,23	CDC2	NM_001786	Homo sapiens cell division cycle 2, G1 to S and G2 to M (CDC2), transcript variant 1, mRNA [NM_001786]
0,20	0,13	PODXL	NM_005397	Homo sapiens podocalyxin-like (PODXL), transcript variant 2, mRNA [NM_005397]
0,20	0,21	DLG7	NM_014750	Homo sapiens discs, large homolog 7 (Drosophila) (DLG7), mRNA [NM_014750]
0,20	0,18	ANLN	NM_018685	Homo sapiens anillin, actin binding protein (ANLN), mRNA [NM_018685]
0,20	0,26	CHI3L2	NM_004000	Homo sapiens chitinase 3-like 2 (CHI3L2), transcript variant 1, mRNA [NM_004000]
0,19	0,22	NCAPG	NM_022346	Homo sapiens non-SMC condensin I complex, subunit G (NCAPG), mRNA [NM_022346]
0,19	0,17	COL4A6	NM_033641	Homo sapiens collagen, type IV, alpha 6 (COL4A6), transcript variant B, mRNA [NM_033641]
0,19	0,16	ENST00000376682	ENST00000376682	Probable G-protein coupled receptor 133 precursor (G-protein coupled receptor PGR25). [Source:Uniprot/SWISSPROT;Acc:Q6QNK2] [ENST00000376682]
0,18	0,19	ID4	NM_001546	Homo sapiens inhibitor of DNA binding 4, dominant negative helix-loop-helix protein (ID4), mRNA [NM_001546]
0,18	0,27	ID1	NM_002165	Homo sapiens inhibitor of DNA binding 1, dominant negative helix-loop-helix protein (ID1), transcript variant 1, mRNA [NM_002165]
0,18	0,25	KLK8	NM_144505	Homo sapiens kallikrein-related peptidase 8 (KLK8), transcript variant 2, mRNA [NM_144505]
0,17	0,24	CCNB2	NM_004701	Homo sapiens cyclin B2 (CCNB2), mRNA [NM_004701]
0,16	0,23	MT1F	NM_005949	Homo sapiens metallothionein 1F (MT1F), mRNA [NM_005949]
0,16	0,34	SLPI	NM_003064	Homo sapiens secretory leukocyte peptidase inhibitor (SLPI), mRNA [NM_003064]
0,15	0,18	CEP55	NM_018131	Homo sapiens centrosomal protein 55kDa (CEP55), mRNA [NM_018131]
0,15	0,23	RPESP	NM_153225	Homo sapiens RPE-spondin (RPESP), mRNA [NM_153225]
0,15	0,17	SLC7A7	NM_003982	Homo sapiens solute carrier family 7 (cationic amino acid transporter, y+ system), member 7 (SLC7A7), mRNA [NM_003982]
0,15	0,25	SPANXD	NM_032417	Homo sapiens SPANX family, member D (SPANXD), mRNA [NM_032417]
0,15	0,18	ZBED2	NM_024508	Homo sapiens zinc finger, BED-type containing 2 (ZBED2), mRNA [NM_024508]

0,14	0,24	RARRES1	NM_002888	Homo sapiens retinoic acid receptor responder (tazarotene induced) 1 (RARRES1), transcript variant 2, mRNA [NM_002888]
0,14	0,22	NOX1	NM_013954	Homo sapiens NADPH oxidase 1 (NOX1), transcript variant NOH-1S, mRNA [NM_013954]
0,14	0,14	UBE2C	NM_181803	Homo sapiens ubiquitin-conjugating enzyme E2C (UBE2C), transcript variant 6, mRNA [NM_181803]
0,14	0,14	NOX1	NM_013955	Homo sapiens NADPH oxidase 1 (NOX1), transcript variant NOH-1Lv, mRNA [NM_013955]
0,14	0,16	NOX1	NM_007052	Homo sapiens NADPH oxidase 1 (NOX1), transcript variant NOH-1L, mRNA [NM_007052]
0,13	0,17	HHIP	NM_022475	Homo sapiens hedgehog interacting protein (HHIP), mRNA [NM_022475]
0,12	0,13	MYH3	NM_002470	Homo sapiens myosin, heavy chain 3, skeletal muscle, embryonic (MYH3), mRNA [NM_002470]
0,12	0,15	CRIP1	NM_001311	Homo sapiens cysteine-rich protein 1 (intestinal) (CRIP1), mRNA [NM_001311]
0,12	0,16	KIAA0101	NM_014736	Homo sapiens KIAA0101 (KIAA0101), transcript variant 1, mRNA [NM_014736]
0,12	0,20	SPANXB2	NM_145664	Homo sapiens SPANX family, member B2 (SPANXB2), mRNA [NM_145664]
0,11	0,10	THBD	NM_000361	Homo sapiens thrombomodulin (THBD), mRNA [NM_000361]
0,11	0,15	CYP17A1	NM_000102	Homo sapiens cytochrome P450, family 17, subfamily A, polypeptide 1 (CYP17A1), mRNA [NM_000102]
0,11	0,15	SFTPD	NM_003019	Homo sapiens surfactant, pulmonary-associated protein D (SFTPD), mRNA [NM_003019]
0,10	0,25	NPY	NM_000905	Homo sapiens neuropeptide Y (NPY), mRNA [NM_000905]
0,10	0,17	CDH1	NM_004360	Homo sapiens cadherin 1, type 1, E-cadherin (epithelial) (CDH1), mRNA [NM_004360]
0,10	0,14	UPK1B	NM_006952	Homo sapiens uroplakin 1B (UPK1B), mRNA [NM_006952]
0,10	0,11	BIRC5	NM_001012271	Homo sapiens baculoviral IAP repeat-containing 5 (survivin) (BIRC5), transcript variant 3, mRNA [NM_001012271]
0,10	0,16	TM4SF1	NM_014220	Homo sapiens transmembrane 4 L six family member 1 (TM4SF1), mRNA [NM_014220]
0,09	0,19	HP	NM_005143	Homo sapiens haptoglobin (HP), mRNA [NM_005143]
0,07	0,10	TK1	NM_003258	Homo sapiens thymidine kinase 1, soluble (TK1), mRNA [NM_003258]
0,06	0,08	TGM1	NM_000359	Homo sapiens transglutaminase 1 (K polypeptide epidermal type I, protein-glutamine-gamma-glutamyltransferase) (TGM1), mRNA [NM_000359]
0,05	0,07	PRG4	NM_005807	Homo sapiens proteoglycan 4 (PRG4), mRNA [NM_005807]
0,04	0,06	KLK7	NM_005046	Homo sapiens kallikrein-related peptidase 7 (KLK7), transcript variant 1, mRNA [NM_005046]

Supplementary Table S3. Microarray induced genes in Epithelioid phenotype.

Ratio Epithelioid	Epithelioid FDR	Gene Symbol	Systematic Name	Description
14,36	0,00007	GREM1	NM_013372	Homo sapiens gremlin 1, cysteine knot superfamily, homolog (Xenopus laevis) (GREM1), mRNA [NM_013372]
7,93	0,00002	CTSK	NM_000396	Homo sapiens cathepsin K (CTSK), mRNA [NM_000396]
7,66	0,00446	MMP1	NM_002421	Homo sapiens matrix metallopeptidase 1 (interstitial collagenase) (MMP1), mRNA [NM_002421]
7,34	0,00001	LUM	NM_002345	Homo sapiens lumican (LUM), mRNA [NM_002345]
6,98	0,00000	TDO2	NM_005651	Homo sapiens tryptophan 2,3-dioxygenase (TDO2), mRNA [NM_005651]
6,63	0,00010	MME	NM_007289	Homo sapiens membrane metallo-endopeptidase (MME), transcript variant 2b, mRNA [NM_007289]
6,23	0,00016	LIMS3	NM_033514	Homo sapiens LIM and senescent cell antigen-like domains 3 (LIMS3), mRNA [NM_033514]
5,60	0,00000	FAP	NM_004460	Homo sapiens fibroblast activation protein, alpha (FAP), mRNA [NM_004460]
5,35	0,00002	TncRNA	NR_002802	Homo sapiens trophoblast-derived noncoding RNA (TncRNA) on chromosome 11 [NR_002802]
5,08	0,00037	RSPO3	NM_032784	Homo sapiens R-spondin 3 homolog (Xenopus laevis) (RSPO3), mRNA [NM_032784]
4,68	0,00039	EVI2A	NM_001003927	Homo sapiens ecotropic viral integration site 2A (EVI2A), transcript variant 1, mRNA [NM_001003927]
4,43	0,00285	TFPI2	NM_006528	Homo sapiens tissue factor pathway inhibitor 2 (TFPI2), mRNA [NM_006528]
4,39	0,00177	POSTN	NM_006475	Homo sapiens periostin, osteoblast specific factor (POSTN), mRNA [NM_006475]
4,39	0,00054	GDF15	NM_004864	Homo sapiens growth differentiation factor 15 (GDF15), mRNA [NM_004864]
4,35	0,00290	TFPI2	ENST00000222543	Tissue factor pathway inhibitor 2 precursor (TFPI-2) (Placental protein 5) (PP5). [Source:Uniprot/SWISSPROT;Acc:P48307] [ENST00000222543]
4,09	0,00017	PLA2G7	NM_005084	Homo sapiens phospholipase A2, group VII (platelet-activating factor acetylhydrolase, plasma) (PLA2G7), mRNA [NM_005084]
4,04	0,00014	SCG2	NM_003469	Homo sapiens secretogranin II (chromogranin C) (SCG2), mRNA [NM_003469]

4,03	0,00004	ADAM12	NM_003474	Homo sapiens ADAM metallopeptidase domain 12 (meltrin alpha) (ADAM12), transcript variant 1, mRNA [NM_003474]
3,92	0,00017	PAG1	NM_018440	Homo sapiens phosphoprotein associated with glycosphingolipid microdomains 1 (PAG1), mRNA [NM_018440]
3,85	0,00194	HTR2B	NM_000867	Homo sapiens 5-hydroxytryptamine (serotonin) receptor 2B (HTR2B), mRNA [NM_000867]
3,84	0,00007	ARL4C	NM_005737	Homo sapiens ADP-ribosylation factor-like 4C (ARL4C), mRNA [NM_005737]
3,60	0,00135	FOS	NM_005252	Homo sapiens v-fos FBJ murine osteosarcoma viral oncogene homolog (FOS), mRNA [NM_005252]
3,55	0,00011	DKK1	NM_012242	Homo sapiens dickkopf homolog 1 (Xenopus laevis) (DKK1), mRNA [NM_012242]
3,55	0,00303	PBEF1	NM_005746	Homo sapiens pre-B-cell colony enhancing factor 1 (PBEF1), mRNA [NM_005746]
3,35	0,00371	ITGA11	NM_001004439	Homo sapiens integrin, alpha 11 (ITGA11), transcript variant 1, mRNA [NM_001004439]
3,28	0,00003	FAM129A	NM_052966	Homo sapiens family with sequence similarity 129, member A (FAM129A), transcript variant 2, mRNA [NM_052966]
3,26	0,00003	HNMT	NM_006895	Homo sapiens histamine N-methyltransferase (HNMT), transcript variant 1, mRNA [NM_006895]
3,26	0,00057	FOXN2	NM_002158	Homo sapiens forkhead box N2 (FOXN2), mRNA [NM_002158]
3,21	0,00006	LIMS1	NM_004987	Homo sapiens LIM and senescent cell antigen-like domains 1 (LIMS1), mRNA [NM_004987]
3,19	0,00021	PPM2C	NM_018444	Homo sapiens protein phosphatase 2C, magnesium-dependent, catalytic subunit (PPM2C), nuclear gene encoding mitochondrial protein, mRNA [NM_018444]
3,08	0,00912	CXCL2	NM_002089	Homo sapiens chemokine (C-X-C motif) ligand 2 (CXCL2), mRNA [NM_002089]
3,01	0,00002	ARMC9	NM_025139	Homo sapiens armadillo repeat containing 9 (ARMC9), mRNA [NM_025139]
3,01	0,00010	TNIK	AB011123	Homo sapiens mRNA for KIAA0551 protein, partial cds. [AB011123]
2,99	0,00009	IL4I1	NM_172374	Homo sapiens interleukin 4 induced 1 (IL4I1), transcript variant 2, mRNA [NM_172374]
2,96	0,00006	CTSL1	NM_001912	Homo sapiens cathepsin L1 (CTSL1), transcript variant 1, mRNA [NM_001912]
2,87	0,00013	SAMD3	NM_001017373	Homo sapiens sterile alpha motif domain containing 3 (SAMD3), transcript variant 1, mRNA [NM_001017373]
2,85	0,00476	LCP1	NM_002298	Homo sapiens lymphocyte cytosolic protein 1 (L-plastin) (LCP1), mRNA [NM_002298]

2,83	0,00348	FOSB	NM_006732	Homo sapiens FBJ murine osteosarcoma viral oncogene homolog B (FOSB), mRNA [NM_006732]
2,82	0,00015	SPHK1	NM_021972	Homo sapiens sphingosine kinase 1 (SPHK1), transcript variant 1, mRNA [NM_021972]
2,81	0,00004	HES1	NM_005524	Homo sapiens hairy and enhancer of split 1, (Drosophila) (HES1), mRNA [NM_005524]
2,79	0,00000	MALAT1	NR_002819	Homo sapiens metastasis associated lung adenocarcinoma transcript 1 (non-coding RNA) (MALAT1) on chromosome 11 [NR_002819]
2,76	0,00094	SMYD3	NM_022743	Homo sapiens SET and MYND domain containing 3 (SMYD3), mRNA [NM_022743]
2,74	0,00240	IVL	NM_005547	Homo sapiens involucrin (IVL), mRNA [NM_005547]
2,71	0,00135	HOXA9	NM_152739	Homo sapiens homeobox A9 (HOXA9), mRNA [NM_152739]
2,71	0,00120	ALDH1A1	NM_000689	Homo sapiens aldehyde dehydrogenase 1 family, member A1 (ALDH1A1), mRNA [NM_000689]
2,68	0,00498	XRCC4	NM_022550	Homo sapiens X-ray repair complementing defective repair in Chinese hamster cells 4 (XRCC4), transcript variant 3, mRNA [NM_022550]
2,67	0,00428	DCN	NM_001920	Homo sapiens decorin (DCN), transcript variant A1, mRNA [NM_001920]
2,64	0,00007	OSBPL8	NM_020841	Homo sapiens oxysterol binding protein-like 8 (OSBPL8), transcript variant 1, mRNA [NM_020841]
2,61	0,00002	ENST00000288911	ENST00000288911	Ankyrin repeat domain-containing protein 36A. [Source:Uniprot/SWISSPROT;Acc:Q6UX02] [ENST00000288911]
2,56	0,00003	KIAA1212	NM_018084	Homo sapiens KIAA1212 (KIAA1212), mRNA [NM_018084]
2,54	0,00081	ABI3BP	NM_015429	Homo sapiens ABI gene family, member 3 (NESH) binding protein (ABI3BP), mRNA [NM_015429]
2,51	0,00024	KLHDC7B	NM_138433	Homo sapiens kelch domain containing 7B (KLHDC7B), mRNA [NM_138433]
2,51	0,00306	HOXA11S	NR_002795	Homo sapiens homeo box A11, antisense (HOXA11S) on chromosome 7 [NR_002795]
2,51	0,00019	VDR	NM_001017535	Homo sapiens vitamin D (1,25- dihydroxyvitamin D3) receptor (VDR), transcript variant 2, mRNA [NM_001017535]
2,49	0,00003	ETS2	NM_005239	Homo sapiens v-ets erythroblastosis virus E26 oncogene homolog 2 (avian) (ETS2), mRNA [NM_005239]
2,49	0,00014	ST3GAL6	NM_006100	Homo sapiens ST3 beta-galactoside alpha-2,3-sialyltransferase 6 (ST3GAL6), mRNA [NM_006100]
2,47	0,00016	MOXD1	NM_015529	Homo sapiens monooxygenase, DBH-like 1 (MOXD1), transcript variant 2, mRNA [NM_015529]
2,46	0,00005	FOXF2	NM_001452	Homo sapiens forkhead box F2 (FOXF2), mRNA [NM_001452]

2,45	0,00091	ADAM8	NM_001109	Homo sapiens ADAM metallopeptidase domain 8 (ADAM8), mRNA [NM_001109]
2,44	0,00000	GOLGA4	NM_002078	Homo sapiens golgi autoantigen, golgin subfamily a, 4 (GOLGA4), mRNA [NM_002078]
2,43	0,00000	HNMT	NM_001024074	Homo sapiens histamine N-methyltransferase (HNMT), transcript variant 2, mRNA [NM_001024074]
2,43	0,00400	TOR3A	NM_022371	Homo sapiens torsin family 3, member A (TOR3A), mRNA [NM_022371]
2,41	0,00002	RRAGD	NM_021244	Homo sapiens Ras-related GTP binding D (RRAGD), mRNA [NM_021244]
2,41	0,00013	GOLGA8B	NM_001023567	Homo sapiens golgi autoantigen, golgin subfamily a, 8B (GOLGA8B), mRNA [NM_001023567]
2,40	0,00036	RGS2	NM_002923	Homo sapiens regulator of G-protein signalling 2, 24kDa (RGS2), mRNA [NM_002923]
2,40	0,00187	APOE	NM_000041	Homo sapiens apolipoprotein E (APOE), mRNA [NM_000041]
2,39	0,00225	SLC22A4	NM_003059	Homo sapiens solute carrier family 22 (organic cation transporter), member 4 (SLC22A4), mRNA [NM_003059]
2,38	0,00001	HEXA	NM_000520	Homo sapiens hexosaminidase A (alpha polypeptide) (HEXA), mRNA [NM_000520]
2,36	0,00040	HK2	NM_000189	Homo sapiens hexokinase 2 (HK2), mRNA [NM_000189]
2,32	0,00003	PPARG	NM_138711	Homo sapiens peroxisome proliferator-activated receptor gamma (PPARG), transcript variant 3, mRNA [NM_138711]
2,32	0,00805	CTHRC1	NM_138455	Homo sapiens collagen triple helix repeat containing 1 (CTHRC1), mRNA [NM_138455]
2,31	0,00068	AEBP1	NM_001129	Homo sapiens AE binding protein 1 (AEBP1), mRNA [NM_001129]
2,31	0,00037	AASS	NM_005763	Homo sapiens aminoadipate-semialdehyde synthase (AASS), mRNA [NM_005763]
2,31	0,00002	PTGDS	NM_000954	Homo sapiens prostaglandin D2 synthase 21kDa (brain) (PTGDS), mRNA [NM_000954]
2,30	0,00286	ENST00000358984	ENST00000358984	Ankyrin repeat domain 30B. [Source:Uniprot/SPTREMBL;Acc:Q4G175] [ENST00000358984]
2,27	0,00098	IL4R	NM_000418	Homo sapiens interleukin 4 receptor (IL4R), transcript variant 1, mRNA [NM_000418]
2,27	0,00818	PHLDA1	NM_007350	Homo sapiens pleckstrin homology-like domain, family A, member 1 (PHLDA1), mRNA [NM_007350]
2,27	0,00329	IL1A	NM_000575	Homo sapiens interleukin 1, alpha (IL1A), mRNA [NM_000575]
2,26	0,00002	HEXA	S76980	HEXA {HEXA4bpDeltaA mutation, exon 11} [human, Tay-Sachs disease patient, mRNA Partial Mutant, 78 nt]. [S76980]

2,26	0,00013	IL13RA1	NM_001560	Homo sapiens interleukin 13 receptor, alpha 1 (IL13RA1), mRNA [NM_001560]
2,26	0,00010	ASAH1	NM_004315	Homo sapiens N-acylsphingosine amidohydrolase (acid ceramidase) 1 (ASAH1), transcript variant 2, mRNA [NM_004315]
2,26	0,00683	PID1	NM_017933	Homo sapiens phosphotyrosine interaction domain containing 1 (PID1), mRNA [NM_017933]
2,25	0,00041	BRUNOL6	NM_052840	Homo sapiens bruno-like 6, RNA binding protein (Drosophila) (BRUNOL6), mRNA [NM_052840]
2,25	0,00087	G0S2	NM_015714	Homo sapiens G0/G1switch 2 (G0S2), mRNA [NM_015714]
2,24	0,00192	CUL4B	NM_003588	Homo sapiens cullin 4B (CUL4B), transcript variant 1, mRNA [NM_003588]
2,24	0,00354	KIAA1913	BC044246	Homo sapiens KIAA1913, mRNA (cDNA clone MGC:50847 IMAGE:5760073), complete cds. [BC044246]
2,23	0,00033	MACF1	NM_012090	Homo sapiens microtubule-actin crosslinking factor 1 (MACF1), transcript variant 1, mRNA [NM_012090]
2,23	0,00002	GOLGA8A	NM_181077	Homo sapiens golgi autoantigen, golgin subfamily a, 8A (GOLGA8A), mRNA [NM_181077]
2,22	0,00000	SEC31B	NM_015490	Homo sapiens SEC31 homolog B (S. cerevisiae) (SEC31B), mRNA [NM_015490]
2,21	0,00062	SNAI2	NM_003068	Homo sapiens snail homolog 2 (Drosophila) (SNAI2), mRNA [NM_003068]
2,21	0,00004	GALE	NM_000403	Homo sapiens UDP-galactose-4-epimerase (GALE), transcript variant 1, mRNA [NM_000403]
2,21	0,00287	CABLES1	NM_138375	Homo sapiens Cdk5 and Abl enzyme substrate 1 (CABLES1), mRNA [NM_138375]
2,19	0,00325	KIAA1913	NM_052913	Homo sapiens KIAA1913 (KIAA1913), mRNA [NM_052913]
2,18	0,00024	GNPDA1	NM_005471	Homo sapiens glucosamine-6-phosphate deaminase 1 (GNPDA1), mRNA [NM_005471]
2,17	0,00061	F2R	NM_001992	Homo sapiens coagulation factor II (thrombin) receptor (F2R), mRNA [NM_001992]
2,17	0,00168	AF119889	AF119889	Homo sapiens PRO2667 mRNA, complete cds. [AF119889]
2,16	0,00607	HMGA1	NM_002131	Homo sapiens high mobility group AT-hook 1 (HMGA1), transcript variant 2, mRNA [NM_002131]
2,16	0,00015	VWCE	NM_152718	Homo sapiens von Willebrand factor C and EGF domains (VWCE), mRNA [NM_152718]
2,16	0,00125	HABP4	NM_014282	Homo sapiens hyaluronan binding protein 4 (HABP4), mRNA [NM_014282]
2,16	0,00013	DKK3	NM_015881	Homo sapiens dickkopf homolog 3 (Xenopus laevis) (DKK3), transcript variant 1, mRNA [NM_015881]

2,15	0,00000	MEIS2	NM_170676	Homo sapiens Meis homeobox 2 (MEIS2), transcript variant d, mRNA [NM_170676]
2,15	0,00126	UPP1	BC047030	Homo sapiens uridine phosphorylase 1, mRNA (cDNA clone MGC:54255 IMAGE:5549432), complete cds. [BC047030]
2,14	0,00005	LAMP2	NM_013995	Homo sapiens lysosomal-associated membrane protein 2 (LAMP2), transcript variant LAMP2B, mRNA [NM_013995]
2,14	0,00226	ZC3H12A	NM_025079	Homo sapiens zinc finger CCCH-type containing 12A (ZC3H12A), mRNA [NM_025079]
2,14	0,00032	ENST00000369326	ENST00000369326	Notch homolog 2 N-terminal like protein [Source:RefSeq_peptide;Acc:NP_982283] [ENST00000369326]
2,14	0,00056	EIF3S10	NM_003750	Homo sapiens eukaryotic translation initiation factor 3, subunit 10 theta, 150/170kDa (EIF3S10), mRNA [NM_003750]
2,13	0,00001	SLFN5	NM_144975	Homo sapiens schlafen family member 5 (SLFN5), mRNA [NM_144975]
2,13	0,00199	HS3ST3A1	NM_006042	Homo sapiens heparan sulfate (glucosamine) 3-O-sulfotransferase 3A1 (HS3ST3A1), mRNA [NM_006042]
2,11	0,00001	HSD17B14	NM_016246	Homo sapiens hydroxysteroid (17-beta) dehydrogenase 14 (HSD17B14), mRNA [NM_016246]
2,11	0,00043	HOXA7	NM_006896	Homo sapiens homeobox A7 (HOXA7), mRNA [NM_006896]
2,11	0,00001	KIAA1641	NM_025190	Homo sapiens KIAA1641 (KIAA1641), mRNA [NM_025190]
2,10	0,00011	HOXC8	NM_022658	Homo sapiens homeobox C8 (HOXC8), mRNA [NM_022658]
2,10	0,00104	SLC19A2	NM_006996	Homo sapiens solute carrier family 19 (thiamine transporter), member 2 (SLC19A2), mRNA [NM_006996]
2,10	0,00006	ANTXR2	NM_058172	Homo sapiens anthrax toxin receptor 2 (ANTXR2), mRNA [NM_058172]
2,09	0,00309	NMB	NM_021077	Homo sapiens neuromedin B (NMB), transcript variant 1, mRNA [NM_021077]
2,09	0,00001	GPNMB	NM_001005340	Homo sapiens glycoprotein (transmembrane) nmb (GPNMB), transcript variant 1, mRNA [NM_001005340]
2,09	0,00003	GRN	NM_002087	Homo sapiens granulin (GRN), mRNA [NM_002087]
2,08	0,00011	NEXN	NM_144573	Homo sapiens nexilin (F actin binding protein) (NEXN), mRNA [NM_144573]
2,08	0,00049	COL9A3	NM_001853	Homo sapiens collagen, type IX, alpha 3 (COL9A3), mRNA [NM_001853]
2,07	0,00006	AF187554	AF187554	Homo sapiens sperm antigen-36 mRNA, complete cds. [AF187554]
2,07	0,00528	EVI2B	NM_006495	Homo sapiens ecotropic viral integration site 2B (EVI2B), mRNA [NM_006495]
2,07	0,00001	COL27A1	AK021957	Homo sapiens cDNA FLJ11895 fis, clone HEMBA1007301, weakly similar to COLLAGEN ALPHA 1(III) CHAIN. [AK021957]

2,07	0,00035	TG	NM_003235	Homo sapiens thyroglobulin (TG), mRNA [NM_003235]
2,06	0,00235	AKAP12	NM_144497	Homo sapiens A kinase (PRKA) anchor protein (gravin) 12 (AKAP12), transcript variant 2, mRNA [NM_144497]
2,04	0,00006	BRI3	NM_015379	Homo sapiens brain protein I3 (BRI3), mRNA [NM_015379]
2,04	0,00020	FBLN5	NM_006329	Homo sapiens fibulin 5 (FBLN5), mRNA [NM_006329]
2,03	0,00004	PXK	NM_017771	Homo sapiens PX domain containing serine/threonine kinase (PXK), mRNA [NM_017771]
2,03	0,00037	SLC43A3	NM_199329	Homo sapiens solute carrier family 43, member 3 (SLC43A3), mRNA [NM_199329]
2,03	0,00138	CDKN1C	NM_000076	Homo sapiens cyclin-dependent kinase inhibitor 1C (p57, Kip2) (CDKN1C), mRNA [NM_000076]
2,03	0,00476	AMPD3	NM_000480	Homo sapiens adenosine monophosphate deaminase (isoform E) (AMPD3), transcript variant 1, mRNA [NM_000480]
2,02	0,00004	GUSBL2	BC065547	Homo sapiens glucuronidase, beta-like 2, mRNA (cDNA clone IMAGE:6047050). [BC065547]
2,02	0,00002	LZTS2	NM_032429	Homo sapiens leucine zipper, putative tumor suppressor 2 (LZTS2), mRNA [NM_032429]
2,02	0,00011	NPC1	NM_000271	Homo sapiens Niemann-Pick disease, type C1 (NPC1), mRNA [NM_000271]
2,01	0,00379	STEAP1	NM_012449	Homo sapiens six transmembrane epithelial antigen of the prostate 1 (STEAP1), mRNA [NM_012449]
2,01	0,00161	TNFRSF6B	NM_032945	Homo sapiens tumor necrosis factor receptor superfamily, member 6b, decoy (TNFRSF6B), transcript variant M68C, mRNA [NM_032945]
2,01	0,00004	CENTB2	NM_012287	Homo sapiens centaurin, beta 2 (CENTB2), mRNA [NM_012287]

Supplementary Table S4. Microarray repressed genes in Epithelioid phenotype.

Ratio Epithelioid	Epithelioid FDR	Gene Symbol	Systematic Name	Description
0,07	0,00000	MYL7	NM_021223	Homo sapiens myosin, light chain 7, regulatory (MYL7), mRNA [NM_021223]
0,09	0,00003	KLK7	NM_005046	Homo sapiens kallikrein-related peptidase 7 (KLK7), transcript variant 1, mRNA [NM_005046]
0,09	0,00012	TGM1	NM_000359	Homo sapiens transglutaminase 1 (K polypeptide epidermal type I, protein-glutamine-gamma-glutamyltransferase) (TGM1), mRNA [NM_000359]
0,11	0,00000	MYLPF	NM_013292	Homo sapiens fast skeletal myosin light chain 2 (MYLPF), mRNA [NM_013292]
0,11	0,00000	APOA1	NM_000039	Homo sapiens apolipoprotein A-I (APOA1), mRNA [NM_000039]
0,11	0,00001	CST6	NM_001323	Homo sapiens cystatin E/M (CST6), mRNA [NM_001323]
0,11	0,00059	PODXL	NM_005397	Homo sapiens podocalyxin-like (PODXL), transcript variant 2, mRNA [NM_005397]
0,12	0,00005	CLIC5	NM_016929	Homo sapiens chloride intracellular channel 5 (CLIC5), mRNA [NM_016929]
0,12	0,00000	PAX8	NM_003466	Homo sapiens paired box gene 8 (PAX8), transcript variant PAX8A, mRNA [NM_003466]
0,12	0,00009	IGFL2	NM_001002915	Homo sapiens IGF-like family member 2 (IGFL2), mRNA [NM_001002915]
0,13	0,00093	TXNIP	NM_006472	Homo sapiens thioredoxin interacting protein (TXNIP), mRNA [NM_006472]
0,14	0,00000	CYP17A1	NM_000102	Homo sapiens cytochrome P450, family 17, subfamily A, polypeptide 1 (CYP17A1), mRNA [NM_000102]
0,15	0,00002	KLK5	NM_012427	Homo sapiens kallikrein-related peptidase 5 (KLK5), transcript variant 1, mRNA [NM_012427]
0,15	0,00083	SFTPD	NM_003019	Homo sapiens surfactant, pulmonary-associated protein D (SFTPD), mRNA [NM_003019]
0,15	0,00008	MYH3	NM_002470	Homo sapiens myosin, heavy chain 3, skeletal muscle, embryonic (MYH3), mRNA [NM_002470]
0,16	0,00001	SLPI	NM_003064	Homo sapiens secretory leukocyte peptidase inhibitor (SLPI), mRNA [NM_003064]
0,16	0,00001	MAL2	NM_052886	Homo sapiens mal, T-cell differentiation protein 2 (MAL2), mRNA [NM_052886]

0,16	0,00000	TM4SF4	NM_004617	Homo sapiens transmembrane 4 L six family member 4 (TM4SF4), mRNA [NM_004617]
0,17	0,00012	HHIP	NM_022475	Homo sapiens hedgehog interacting protein (HHIP), mRNA [NM_022475]
0,18	0,00000	SPOCK2	NM_014767	Homo sapiens sparc/osteonectin, cwcv and kazal-like domains proteoglycan (testican) 2 (SPOCK2), mRNA [NM_014767]
0,19	0,00050	TIMP3	NM_000362	Homo sapiens TIMP metallopeptidase inhibitor 3 (Sorsby fundus dystrophy, pseudoinflammatory) (TIMP3), mRNA [NM_000362]
0,19	0,00008	KLK8	NM_144505	Homo sapiens kallikrein-related peptidase 8 (KLK8), transcript variant 2, mRNA [NM_144505]
0,20	0,00003	ADRA2A	NM_000681	Homo sapiens adrenergic, alpha-2A-, receptor (ADRA2A), mRNA [NM_000681]
0,21	0,00000	TMOD1	NM_003275	Homo sapiens tropomodulin 1 (TMOD1), mRNA [NM_003275]
0,21	0,00019	CRIP1	NM_001311	Homo sapiens cysteine-rich protein 1 (intestinal) (CRIP1), mRNA [NM_001311]
0,21	0,00020	ZBED2	NM_024508	Homo sapiens zinc finger, BED-type containing 2 (ZBED2), mRNA [NM_024508]
0,22	0,00235	EPS8L1	NM_133180	Homo sapiens EPS8-like 1 (EPS8L1), transcript variant 1, mRNA [NM_133180]
0,22	0,00009	ISLR	NM_005545	Homo sapiens immunoglobulin superfamily containing leucine-rich repeat (ISLR), transcript variant 1, mRNA [NM_005545]
0,22	0,00010	SMTNL2	NM_198501	Homo sapiens smoothelin-like 2 (SMTNL2), mRNA [NM_198501]
0,23	0,00004	NPHS1	NM_004646	Homo sapiens nephrosis 1, congenital, Finnish type (nephrin) (NPHS1), mRNA [NM_004646]
0,24	0,00018	HP	NM_005143	Homo sapiens haptoglobin (HP), mRNA [NM_005143]
0,24	0,00006	VTN	NM_000638	Homo sapiens vitronectin (VTN), mRNA [NM_000638]
0,24	0,00000	BARX1	NM_021570	Homo sapiens BarH-like homeobox 1 (BARX1), mRNA [NM_021570]
0,24	0,00002	UPK1B	NM_006952	Homo sapiens uroplakin 1B (UPK1B), mRNA [NM_006952]
0,25	0,00002	CLEC4M	NM_214677	Homo sapiens C-type lectin domain family 4, member M (CLEC4M), transcript variant 4, mRNA [NM_214677]
0,26	0,00204	TSPAN8	NM_004616	Homo sapiens tetraspanin 8 (TSPAN8), mRNA [NM_004616]
0,26	0,00021	ANGPTL7	NM_021146	Homo sapiens angiopoietin-like 7 (ANGPTL7), mRNA [NM_021146]
0,27	0,00000	CHI3L2	NM_004000	Homo sapiens chitinase 3-like 2 (CHI3L2), transcript variant 1, mRNA [NM_004000]
0,27	0,00027	KCNB1	NM_004975	Homo sapiens potassium voltage-gated channel, Shab-related subfamily, member 1 (KCNB1), mRNA [NM_004975]
0,27	0,00001	CLDN1	NM_021101	Homo sapiens claudin 1 (CLDN1), mRNA [NM_021101]
0,27	0,00044	NOX1	NM_013955	Homo sapiens NADPH oxidase 1 (NOX1), transcript variant NOH-1Lv, mRNA [NM_013955]

0,28	0,00014	ENST00000376682	ENST00000376682	Probable G-protein coupled receptor 133 precursor (G-protein coupled receptor PGR25). [Source:Uniprot/SWISSPROT;Acc:Q6QNK2] [ENST00000376682]
0,28	0,00009	HPR	NM_020995	Homo sapiens haptoglobin-related protein (HPR), mRNA [NM_020995]
0,28	0,00083	MFAP5	NM_003480	Homo sapiens microfibrillar associated protein 5 (MFAP5), mRNA [NM_003480]
0,29	0,00012	RARRES1	NM_002888	Homo sapiens retinoic acid receptor responder (tazarotene induced) 1 (RARRES1), transcript variant 2, mRNA [NM_002888]
0,29	0,00013	DSCR1	NM_004414	Homo sapiens Down syndrome critical region gene 1 (DSCR1), transcript variant 1, mRNA [NM_004414]
0,30	0,00215	NPPB	NM_002521	Homo sapiens natriuretic peptide precursor B (NPPB), mRNA [NM_002521]
0,30	0,00000	D4S234E	NM_014392	Homo sapiens DNA segment on chromosome 4 (unique) 234 expressed sequence (D4S234E), transcript variant 1, mRNA [NM_014392]
0,30	0,00718	THBD	NM_000361	Homo sapiens thrombomodulin (THBD), mRNA [NM_000361]
0,30	0,00020	SYNPO	ENST00000307662	Synaptopodin. [Source:Uniprot/SWISSPROT;Acc:Q8N3V7] [ENST00000307662]
0,30	0,00025	ARRDC4	NM_183376	Homo sapiens arrestin domain containing 4 (ARRDC4), mRNA [NM_183376]
0,30	0,00012	GPR56	NM_201525	Homo sapiens G protein-coupled receptor 56 (GPR56), transcript variant 3, mRNA [NM_201525]
0,31	0,00031	CPA4	NM_016352	Homo sapiens carboxypeptidase A4 (CPA4), mRNA [NM_016352]
0,31	0,00031	SPANXD	NM_032417	Homo sapiens SPANX family, member D (SPANXD), mRNA [NM_032417]
0,31	0,00102	HSD17B6	NM_003725	Homo sapiens hydroxysteroid (17-beta) dehydrogenase 6 homolog (mouse) (HSD17B6), mRNA [NM_003725]
0,31	0,00036	NOX1	NM_007052	Homo sapiens NADPH oxidase 1 (NOX1), transcript variant NOH-1L, mRNA [NM_007052]
0,31	0,00010	DKFZp761P0423	ENST00000330777	Tyrosine-protein kinase SgK223 (EC 2.7.10.2) (Sugen kinase 223). [Source:Uniprot/SWISSPROT;Acc:Q86YV5] [ENST00000330777]
0,31	0,00008	HIST1H2AC	ENST00000314088	Histone H2A type 1-C. [Source:Uniprot/SWISSPROT;Acc:Q93077] [ENST00000314088]
0,31	0,00005	TSPAN7	NM_004615	Homo sapiens tetraspanin 7 (TSPAN7), mRNA [NM_004615]
0,31	0,00000	ARMC4	NM_018076	Homo sapiens armadillo repeat containing 4 (ARMC4), mRNA [NM_018076]
0,31	0,00885	CRLF1	NM_004750	Homo sapiens cytokine receptor-like factor 1 (CRLF1), mRNA [NM_004750]
0,31	0,00064	CAPN6	NM_014289	Homo sapiens calpain 6 (CAPN6), mRNA [NM_014289]

0,31	0,00031	CXCL14	NM_004887	Homo sapiens chemokine (C-X-C motif) ligand 14 (CXCL14), mRNA [NM_004887]
0,32	0,00062	TM4SF1	NM_014220	Homo sapiens transmembrane 4 L six family member 1 (TM4SF1), mRNA [NM_014220]
0,32	0,00003	EPPK1	NM_031308	Homo sapiens epiplakin 1 (EPPK1), mRNA [NM_031308]
0,32	0,00100	ITM2A	NM_004867	Homo sapiens integral membrane protein 2A (ITM2A), mRNA [NM_004867]
0,32	0,00040	PPP1R12B	NM_032105	Homo sapiens protein phosphatase 1, regulatory (inhibitor) subunit 12B (PPP1R12B), transcript variant 2, mRNA [NM_032105]
0,32	0,00031	SFRP1	NM_003012	Homo sapiens secreted frizzled-related protein 1 (SFRP1), mRNA [NM_003012]
0,32	0,00148	FGF9	NM_002010	Homo sapiens fibroblast growth factor 9 (glia-activating factor) (FGF9), mRNA [NM_002010]
0,33	0,00095	SPANXB2	NM_145664	Homo sapiens SPANX family, member B2 (SPANXB2), mRNA [NM_145664]
0,33	0,00000	JAM2	NM_021219	Homo sapiens junctional adhesion molecule 2 (JAM2), mRNA [NM_021219]
0,33	0,00006	RNF182	NM_152737	Homo sapiens ring finger protein 182 (RNF182), mRNA [NM_152737]
0,33	0,00000	MYH2	NM_017534	Homo sapiens myosin, heavy chain 2, skeletal muscle, adult (MYH2), mRNA [NM_017534]
0,33	0,00000	TMEM130	NM_152913	Homo sapiens transmembrane protein 130 (TMEM130), mRNA [NM_152913]
0,34	0,00047	CNN1	NM_001299	Homo sapiens calponin 1, basic, smooth muscle (CNN1), mRNA [NM_001299]
0,34	0,00048	WNT4	NM_030761	Homo sapiens wingless-type MMTV integration site family, member 4 (WNT4), mRNA [NM_030761]
0,34	0,00016	RSPO1	NM_001038633	Homo sapiens R-spondin homolog (Xenopus laevis) (RSPO1), mRNA [NM_001038633]
0,34	0,00007	IL18	NM_001562	Homo sapiens interleukin 18 (interferon-gamma-inducing factor) (IL18), mRNA [NM_001562]
0,34	0,00020	KCNJ8	NM_004982	Homo sapiens potassium inwardly-rectifying channel, subfamily J, member 8 (KCNJ8), mRNA [NM_004982]
0,34	0,00000	GSG1	NM_031289	Homo sapiens germ cell associated 1 (GSG1), transcript variant 1, mRNA [NM_031289]
0,34	0,00004	IGFBP6	NM_002178	Homo sapiens insulin-like growth factor binding protein 6 (IGFBP6), mRNA [NM_002178]
0,35	0,00001	AS3MT	NM_020682	Homo sapiens arsenic (+3 oxidation state) methyltransferase (AS3MT), mRNA [NM_020682]

0,35	0,00171	NOX1	NM_013954	Homo sapiens NADPH oxidase 1 (NOX1), transcript variant NOH-1S, mRNA [NM_013954]
0,35	0,00001	KCNA4	NM_002233	Homo sapiens potassium voltage-gated channel, shaker-related subfamily, member 4 (KCNA4), mRNA [NM_002233]
0,35	0,00060	CDH10	NM_006727	Homo sapiens cadherin 10, type 2 (T2-cadherin) (CDH10), mRNA [NM_006727]
0,35	0,00002	XAGE2	NM_130777	Homo sapiens X antigen family, member 2 (XAGE2), mRNA [NM_130777]
0,35	0,00046	CGN	NM_020770	Homo sapiens cingulin (CGN), mRNA [NM_020770]
0,35	0,00000	GALNTL2	NM_054110	Homo sapiens UDP-N-acetyl-alpha-D-galactosamine:polypeptide N-acetylgalactosaminyltransferase-like 2 (GALNTL2), mRNA [NM_054110]
0,36	0,00010	GLT8D4	NM_001080393	Homo sapiens similar to CG9996-PA (LOC727936), mRNA [NM_001080393]
0,36	0,00009	PRPH	NM_006262	Homo sapiens peripherin (PRPH), mRNA [NM_006262]
0,36	0,00024	HBD	NM_000519	Homo sapiens hemoglobin, delta (HBD), mRNA [NM_000519]
0,36	0,00048	WFDC2	NM_080734	Homo sapiens WAP four-disulfide core domain 2 (WFDC2), transcript variant 4, mRNA [NM_080734]
0,36	0,00045	ADAMTS5	NM_007038	Homo sapiens ADAM metallopeptidase with thrombospondin type 1 motif, 5 (aggrecanase-2) (ADAMTS5), mRNA [NM_007038]
0,36	0,00000	DAPP1	NM_014395	Homo sapiens dual adaptor of phosphotyrosine and 3-phosphoinositides (DAPP1), mRNA [NM_014395]
0,37	0,00070	DIRAS3	NM_004675	Homo sapiens DIRAS family, GTP-binding RAS-like 3 (DIRAS3), mRNA [NM_004675]
0,37	0,00027	PLCB1	NM_182734	Homo sapiens phospholipase C, beta 1 (phosphoinositide-specific) (PLCB1), transcript variant 2, mRNA [NM_182734]
0,37	0,00501	EXTL1	NM_004455	Homo sapiens exostoses (multiple)-like 1 (EXTL1), mRNA [NM_004455]
0,37	0,00070	LMOD1	NM_012134	Homo sapiens leiomodin 1 (smooth muscle) (LMOD1), mRNA [NM_012134]
0,37	0,00025	RBP1	NM_002899	Homo sapiens retinol binding protein 1, cellular (RBP1), mRNA [NM_002899]
0,37	0,00033	PHGDH	NM_006623	Homo sapiens phosphoglycerate dehydrogenase (PHGDH), mRNA [NM_006623]
0,37	0,00002	NLRP1	BC051787	Homo sapiens NLR family, pyrin domain containing 1, mRNA (cDNA clone MGC:57544 IMAGE:5756099), complete cds. [BC051787]
0,38	0,00019	CLDN15	NM_014343	Homo sapiens claudin 15 (CLDN15), transcript variant 1, mRNA [NM_014343]

0,38	0,00017	LRRN2	NM_201630	Homo sapiens leucine rich repeat neuronal 2 (LRRN2), transcript variant 2, mRNA [NM_201630]
0,38	0,00113	ZNF652	NM_014897	Homo sapiens zinc finger protein 652 (ZNF652), mRNA [NM_014897]
0,38	0,00020	ITIH3	NM_002217	Homo sapiens inter-alpha (globulin) inhibitor H3 (ITIH3), mRNA [NM_002217]
0,38	0,00022	SH3GL3	NM_003027	Homo sapiens SH3-domain GRB2-like 3 (SH3GL3), mRNA [NM_003027]
0,38	0,00110	GALNT14	NM_024572	Homo sapiens UDP-N-acetyl-alpha-D-galactosamine:polypeptide N-acetylgalactosaminyltransferase 14 (GalNAc-T14) (GALNT14), mRNA [NM_024572]
0,39	0,00036	SARM1	NM_015077	Homo sapiens sterile alpha and TIR motif containing 1 (SARM1), mRNA [NM_015077]
0,39	0,00081	CCDC71	NM_022903	Homo sapiens coiled-coil domain containing 71 (CCDC71), mRNA [NM_022903]
0,39	0,00028	GAS1	NM_002048	Homo sapiens growth arrest-specific 1 (GAS1), mRNA [NM_002048]
0,39	0,00014	IFI27	NM_005532	Homo sapiens interferon, alpha-inducible protein 27 (IFI27), mRNA [NM_005532]
0,39	0,00016	CHRNA3	NM_000743	Homo sapiens cholinergic receptor, nicotinic, alpha 3 (CHRNA3), mRNA [NM_000743]
0,39	0,00085	PDZK1IP1	NM_005764	Homo sapiens PDZK1 interacting protein 1 (PDZK1IP1), mRNA [NM_005764]
0,39	0,00003	HS6ST2	NM_147175	Homo sapiens heparan sulfate 6-O-sulfotransferase 2 (HS6ST2), transcript variant S, mRNA [NM_147175]
0,39	0,00041	GATA5	NM_080473	Homo sapiens GATA binding protein 5 (GATA5), mRNA [NM_080473]
0,39	0,00000	NMNAT2	NM_015039	Homo sapiens nicotinamide nucleotide adenylyltransferase 2 (NMNAT2), transcript variant 1, mRNA [NM_015039]
0,39	0,00002	OSAP	NM_032623	Homo sapiens ovary-specific acidic protein (OSAP), mRNA [NM_032623]
0,39	0,00002	AP1S1	NM_057089	Homo sapiens adaptor-related protein complex 1, sigma 1 subunit (AP1S1), transcript variant 2, mRNA [NM_057089]
0,39	0,00003	AP1S1	NM_001283	Homo sapiens adaptor-related protein complex 1, sigma 1 subunit (AP1S1), transcript variant 1, mRNA [NM_001283]
0,39	0,00394	CDH1	NM_004360	Homo sapiens cadherin 1, type 1, E-cadherin (epithelial) (CDH1), mRNA [NM_004360]
0,39	0,00001	CYP26B1	NM_019885	Homo sapiens cytochrome P450, family 26, subfamily B, polypeptide 1 (CYP26B1), mRNA [NM_019885]
0,40	0,00013	SMPDL3B	NM_001009568	Homo sapiens sphingomyelin phosphodiesterase, acid-like 3B (SMPDL3B), transcript variant 2, mRNA [NM_001009568]

0,40	0,00002	ARHGDIG	NM_001176	Homo sapiens Rho GDP dissociation inhibitor (GDI) gamma (ARHGDIG), mRNA [NM_001176]
0,40	0,00001	THC2522889	THC2522889	GPR27_HUMAN (Q9NS67) Probable G-protein coupled receptor 27 (Super conserved receptor expressed in brain 1), complete [THC2522889]
0,40	0,00488	PLAT	NM_000930	Homo sapiens plasminogen activator, tissue (PLAT), transcript variant 1, mRNA [NM_000930]
0,40	0,00012	TMEM139	NM_153345	Homo sapiens transmembrane protein 139 (TMEM139), mRNA [NM_153345]
0,40	0,00008	RERG	NM_032918	Homo sapiens RAS-like, estrogen-regulated, growth inhibitor (RERG), mRNA [NM_032918]
0,40	0,00001	S100A10	NM_002966	Homo sapiens S100 calcium binding protein A10 (S100A10), mRNA [NM_002966]
0,40	0,00020	CYTL1	NM_018659	Homo sapiens cytokine-like 1 (CYTL1), mRNA [NM_018659]
0,40	0,00001	ATP6V1G2	NM_130463	Homo sapiens ATPase, H+ transporting, lysosomal 13kDa, V1 subunit G2 (ATP6V1G2), transcript variant 1, mRNA [NM_130463]
0,40	0,00005	PROCR	NM_006404	Homo sapiens protein C receptor, endothelial (EPCR) (PROCR), mRNA [NM_006404]
0,40	0,00003	RPL31	BC070210	Homo sapiens ribosomal protein L31, mRNA (cDNA clone MGC:88191 IMAGE:4714258), complete cds. [BC070210]
0,41	0,00029	SLC16A1	NM_003051	Homo sapiens solute carrier family 16, member 1 (monocarboxylic acid transporter 1) (SLC16A1), mRNA [NM_003051]
0,41	0,00035	ITPR1	NM_002222	Homo sapiens inositol 1,4,5-triphosphate receptor, type 1 (ITPR1), mRNA [NM_002222]
0,41	0,00045	LGALS2	NM_006498	Homo sapiens lectin, galactoside-binding, soluble, 2 (LGALS2), mRNA [NM_006498]
0,41	0,00002	CCND2	NM_001759	Homo sapiens cyclin D2 (CCND2), mRNA [NM_001759]
0,41	0,00002	EEF1A2	NM_001958	Homo sapiens eukaryotic translation elongation factor 1 alpha 2 (EEF1A2), mRNA [NM_001958]
0,41	0,00078	PTGS1	NM_000962	Homo sapiens prostaglandin-endoperoxide synthase 1 (prostaglandin G/H synthase and cyclooxygenase) (PTGS1), transcript variant 1, mRNA [NM_000962]
0,41	0,00008	CCL26	NM_006072	Homo sapiens chemokine (C-C motif) ligand 26 (CCL26), mRNA [NM_006072]
0,42	0,00048	GAL3ST1	NM_004861	Homo sapiens galactose-3-O-sulfotransferase 1 (GAL3ST1), mRNA [NM_004861]
0,42	0,00043	EGFLAM	NM_152403	Homo sapiens EGF-like, fibronectin type III and laminin G domains (EGFLAM), transcript variant 1, mRNA [NM_152403]

0,42	0,00000	DCDC2	NM_016356	Homo sapiens doublecortin domain containing 2 (DCDC2), mRNA [NM_016356]
0,42	0,00008	CYP2J2	NM_000775	Homo sapiens cytochrome P450, family 2, subfamily J, polypeptide 2 (CYP2J2), mRNA [NM_000775]
0,42	0,00001	TMEM16B	NM_020373	Homo sapiens transmembrane protein 16B (TMEM16B), mRNA [NM_020373]
0,42	0,00097	MAOB	NM_000898	Homo sapiens monoamine oxidase B (MAOB), nuclear gene encoding mitochondrial protein, mRNA [NM_000898]
0,42	0,00005	KRT8	NM_002273	Homo sapiens keratin 8 (KRT8), mRNA [NM_002273]
0,42	0,00012	CADM3	NM_021189	Homo sapiens cell adhesion molecule 3 (CADM3), mRNA [NM_021189]
0,42	0,00021	WFDC2	NM_080736	Homo sapiens WAP four-disulfide core domain 2 (WFDC2), transcript variant 2, mRNA [NM_080736]
0,42	0,00033	TNFRSF8	NM_001243	Homo sapiens tumor necrosis factor receptor superfamily, member 8 (TNFRSF8), transcript variant 1, mRNA [NM_001243]
0,42	0,00608	CXCL6	NM_002993	Homo sapiens chemokine (C-X-C motif) ligand 6 (granulocyte chemotactic protein 2) (CXCL6), mRNA [NM_002993]
0,42	0,00007	CRYAB	NM_001885	Homo sapiens crystallin, alpha B (CRYAB), mRNA [NM_001885]
0,43	0,00207	SPARC	NM_003118	Homo sapiens secreted protein, acidic, cysteine-rich (osteonectin) (SPARC), mRNA [NM_003118]
0,43	0,00025	GPR81	ENST00000356987	Probable G-protein coupled receptor 81 (G-protein coupled receptor 104). [Source:Uniprot/SWISSPROT;Acc:Q9BXC0] [ENST00000356987]
0,43	0,00010	CD24	L33930	Homo sapiens CD24 signal transducer mRNA, complete cds and 3' region. [L33930]
0,43	0,00049	PDIA2	NM_006849	Homo sapiens protein disulfide isomerase family A, member 2 (PDIA2), mRNA [NM_006849]
0,43	0,00056	AIF1	NM_004847	Homo sapiens allograft inflammatory factor 1 (AIF1), transcript variant 2, mRNA [NM_004847]
0,43	0,00066	KLK10	NM_002776	Homo sapiens kallikrein-related peptidase 10 (KLK10), transcript variant 1, mRNA [NM_002776]
0,43	0,00001	WNT10A	NM_025216	Homo sapiens wingless-type MMTV integration site family, member 10A (WNT10A), mRNA [NM_025216]
0,43	0,00000	LHFPL4	NM_198560	Homo sapiens lipoma HMGIC fusion partner-like 4 (LHFPL4), mRNA [NM_198560]
0,43	0,00008	SCARA3	NM_016240	Homo sapiens scavenger receptor class A, member 3 (SCARA3), transcript variant 1, mRNA [NM_016240]
0,43	0,00002	CREB5	NM_182898	Homo sapiens cAMP responsive element binding protein 5 (CREB5), transcript variant 1, mRNA [NM_182898]

0,43	0,00013	CLU	NM_203339	Homo sapiens clusterin (CLU), transcript variant 2, mRNA [NM_203339]
0,43	0,00055	DKFZP586H2123	NM_015430	Homo sapiens regeneration associated muscle protease (DKFZP586H2123), transcript variant 1, mRNA [NM_015430]
0,43	0,00186	TMPRSS3	NM_032405	Homo sapiens transmembrane protease, serine 3 (TMPRSS3), transcript variant D, mRNA [NM_032405]
0,43	0,00024	ETV7	NM_016135	Homo sapiens ets variant gene 7 (TEL2 oncogene) (ETV7), mRNA [NM_016135]
0,43	0,00000	BCAS1	NM_003657	Homo sapiens breast carcinoma amplified sequence 1 (BCAS1), mRNA [NM_003657]
0,44	0,00689	CDKN2A	NM_058197	Homo sapiens cyclin-dependent kinase inhibitor 2A (melanoma, p16, inhibits CDK4) (CDKN2A), transcript variant 3, mRNA [NM_058197]
0,44	0,00038	SLC7A4	NM_004173	Homo sapiens solute carrier family 7 (cationic amino acid transporter, y+ system), member 4 (SLC7A4), mRNA [NM_004173]
0,44	0,00108	PDGFRB	NM_002609	Homo sapiens platelet-derived growth factor receptor, beta polypeptide (PDGFRB), mRNA [NM_002609]
0,44	0,00000	NKX2-5	NM_004387	Homo sapiens NK2 transcription factor related, locus 5 (Drosophila) (NKX2-5), mRNA [NM_004387]
0,44	0,00083	CCNYL1	NM_152523	Homo sapiens cyclin Y-like 1 (CCNYL1), mRNA [NM_152523]
0,44	0,00109	SPRR2D	NM_006945	Homo sapiens small proline-rich protein 2D (SPRR2D), mRNA [NM_006945]
0,44	0,00000	AFAP1L2	NM_001001936	Homo sapiens actin filament associated protein 1-like 2 (AFAP1L2), transcript variant 1, mRNA [NM_001001936]
0,44	0,00004	RAB17	NM_022449	Homo sapiens RAB17, member RAS oncogene family (RAB17), mRNA [NM_022449]
0,44	0,00001	AHR	NM_001621	Homo sapiens aryl hydrocarbon receptor (AHR), mRNA [NM_001621]
0,44	0,00043	REEP1	NM_022912	Homo sapiens receptor accessory protein 1 (REEP1), mRNA [NM_022912]
0,44	0,00042	FSTL1	NM_007085	Homo sapiens follistatin-like 1 (FSTL1), mRNA [NM_007085]
0,44	0,00612	CA9	NM_001216	Homo sapiens carbonic anhydrase IX (CA9), mRNA [NM_001216]
0,45	0,00180	MUC16	NM_024690	Homo sapiens mucin 16, cell surface associated (MUC16), mRNA [NM_024690]
0,45	0,00002	FCRLB	NM_001002901	Homo sapiens Fc receptor-like B (FCRLB), mRNA [NM_001002901]
0,45	0,00009	GLYATL1	NM_080661	Homo sapiens glycine-N-acyltransferase-like 1 (GLYATL1), mRNA [NM_080661]
0,45	0,00003	FSCN1	NM_003088	Homo sapiens fascin homolog 1, actin-bundling protein (Strongylocentrotus purpuratus) (FSCN1), mRNA [NM_003088]
0,45	0,00002	MID1	NM_033290	Homo sapiens midline 1 (Opitz/BBB syndrome) (MID1), transcript variant 3, mRNA [NM_033290]

0,45	0,00826	COL4A6	NM_033641	Homo sapiens collagen, type IV, alpha 6 (COL4A6), transcript variant B, mRNA [NM_033641]
0,45	0,00283	C3	NM_000064	Homo sapiens complement component 3 (C3), mRNA [NM_000064]
0,45	0,00014	SAMD4A	AB028976	Homo sapiens mRNA for KIAA1053 protein, partial cds. [AB028976]
0,45	0,00051	AQP9	NM_020980	Homo sapiens aquaporin 9 (AQP9), mRNA [NM_020980]
0,45	0,00094	METTL7A	NM_014033	Homo sapiens methyltransferase like 7A (METTL7A), mRNA [NM_014033]
0,45	0,00570	CFB	NM_001710	Homo sapiens complement factor B (CFB), mRNA [NM_001710]
0,45	0,00135	PLCE1	NM_016341	Homo sapiens phospholipase C, epsilon 1 (PLCE1), mRNA [NM_016341]
0,45	0,00165	KCNK5	NM_003740	Homo sapiens potassium channel, subfamily K, member 5 (KCNK5), mRNA [NM_003740]
0,45	0,00001	HPD	NM_002150	Homo sapiens 4-hydroxyphenylpyruvate dioxygenase (HPD), mRNA [NM_002150]
0,45	0,00225	HOXB9	NM_024017	Homo sapiens homeobox B9 (HOXB9), mRNA [NM_024017]
0,46	0,00010	FST	NM_013409	Homo sapiens follistatin (FST), transcript variant FST344, mRNA [NM_013409]
0,46	0,00001	SCRG1	NM_007281	Homo sapiens scrapie responsive protein 1 (SCRG1), mRNA [NM_007281]
0,46	0,00000	ALDH8A1	NM_022568	Homo sapiens aldehyde dehydrogenase 8 family, member A1 (ALDH8A1), transcript variant 1, mRNA [NM_022568]
0,46	0,00002	AGXT2L1	NM_031279	Homo sapiens alanine-glyoxylate aminotransferase 2-like 1 (AGXT2L1), mRNA [NM_031279]
0,46	0,00006	TNFRSF9	NM_001561	Homo sapiens tumor necrosis factor receptor superfamily, member 9 (TNFRSF9), mRNA [NM_001561]
0,46	0,00036	MAP1B	NM_005909	Homo sapiens microtubule-associated protein 1B (MAP1B), transcript variant 1, mRNA [NM_005909]
0,46	0,00083	CDKN2B	NM_078487	Homo sapiens cyclin-dependent kinase inhibitor 2B (p15, inhibits CDK4) (CDKN2B), transcript variant 2, mRNA [NM_078487]
0,46	0,00043	ANKRD35	NM_144698	Homo sapiens ankyrin repeat domain 35 (ANKRD35), mRNA [NM_144698]
0,46	0,00220	PFDN6	NM_014260	Homo sapiens prefoldin subunit 6 (PFDN6), mRNA [NM_014260]
0,46	0,00003	CYFIP2	NM_001037332	Homo sapiens cytoplasmic FMR1 interacting protein 2 (CYFIP2), transcript variant 2, mRNA [NM_001037332]
0,46	0,00523	MEST	NM_002402	Homo sapiens mesoderm specific transcript homolog (mouse) (MEST), transcript variant 1, mRNA [NM_002402]
0,46	0,00017	TNNT1	BC107798	Homo sapiens troponin T type 1 (skeletal, slow), mRNA (cDNA clone MGC:104241 IMAGE:4247379), complete cds. [BC107798]

0,46	0,00503	CXCR7	NM_020311	Homo sapiens chemokine (C-X-C motif) receptor 7 (CXCR7), transcript variant 2, mRNA [NM_020311]
0,46	0,00230	WNT2B	NM_004185	Homo sapiens wingless-type MMTV integration site family, member 2B (WNT2B), transcript variant WNT-2B1, mRNA [NM_004185]
0,46	0,00191	CRYBB2	NM_000496	Homo sapiens crystallin, beta B2 (CRYBB2), mRNA [NM_000496]
0,46	0,00079	MGAM	NM_004668	Homo sapiens maltase-glucoamylase (alpha-glucosidase) (MGAM), mRNA [NM_004668]
0,46	0,00021	HIST1H2BK	NM_080593	Homo sapiens histone cluster 1, H2bk (HIST1H2BK), mRNA [NM_080593]
0,46	0,00007	TUFT1	NM_020127	Homo sapiens tuftelin 1 (TUFT1), mRNA [NM_020127]
0,46	0,00003	CTNND2	NM_001332	Homo sapiens catenin (cadherin-associated protein), delta 2 (neural plakophilin-related arm-repeat protein) (CTNND2), mRNA [NM_001332]
0,46	0,00304	FBXL16	NM_153350	Homo sapiens F-box and leucine-rich repeat protein 16 (FBXL16), mRNA [NM_153350]
0,46	0,00006	HIST2H4A	NM_003548	Homo sapiens histone cluster 2, H4a (HIST2H4A), mRNA [NM_003548]
0,46	0,00001	THSD3	NM_199265	Homo sapiens thrombospondin, type I, domain containing 3 (THSD3), transcript variant 2, mRNA [NM_199265]
0,47	0,00013	LONRF2	NM_198461	Homo sapiens LON peptidase N-terminal domain and ring finger 2 (LONRF2), mRNA [NM_198461]
0,47	0,00000	TMEM108	NM_023943	Homo sapiens transmembrane protein 108 (TMEM108), mRNA [NM_023943]
0,47	0,00000	HAPLN1	NM_001884	Homo sapiens hyaluronan and proteoglycan link protein 1 (HAPLN1), mRNA [NM_001884]
0,47	0,00009	MYO5C	NM_018728	Homo sapiens myosin VC (MYO5C), mRNA [NM_018728]
0,47	0,00169	AOX1	NM_001159	Homo sapiens aldehyde oxidase 1 (AOX1), mRNA [NM_001159]
0,47	0,00123	KLK11	NM_144947	Homo sapiens kallikrein-related peptidase 11 (KLK11), transcript variant 2, mRNA [NM_144947]
0,47	0,00002	ENST00000219169	ENST00000219169	Nuclear transport factor 2 (NTF-2) (Placental protein 15) (PP15). [Source:Uniprot/SWISSPROT;Acc:P61970] [ENST00000219169]
0,47	0,00064	MED18	NM_017638	Homo sapiens mediator of RNA polymerase II transcription, subunit 18 homolog (S. cerevisiae) (MED18), mRNA [NM_017638]
0,48	0,00060	PLCB4	NM_000933	Homo sapiens phospholipase C, beta 4 (PLCB4), transcript variant 1, mRNA [NM_000933]
0,48	0,00021	RGN	NM_004683	Homo sapiens regucalcin (senescence marker protein-30) (RGN), transcript variant 1, mRNA [NM_004683]
0,48	0,00000	UTS2	NM_021995	Homo sapiens urotensin 2 (UTS2), transcript variant 1, mRNA [NM_021995]

0,48	0,00067	FAM5B	NM_021165	Homo sapiens family with sequence similarity 5, member B (FAM5B), mRNA [NM_021165]
0,48	0,00011	ABCA8	NM_007168	Homo sapiens ATP-binding cassette, sub-family A (ABC1), member 8 (ABCA8), mRNA [NM_007168]
0,48	0,00633	SAMD5	NM_001030060	Homo sapiens SAM domain containing 1 (LOC389432), mRNA [NM_001030060]
0,48	0,00007	TRAPPC2	NM_001011658	Homo sapiens trafficking protein particle complex 2 (TRAPPC2), transcript variant 1, mRNA [NM_001011658]
0,48	0,00215	NANOS1	NM_199461	Homo sapiens nanos homolog 1 (Drosophila) (NANOS1), transcript variant 1, mRNA [NM_199461]
0,48	0,00231	PDGFRL	NM_006207	Homo sapiens platelet-derived growth factor receptor-like (PDGFRL), mRNA [NM_006207]
0,48	0,00010	HIST1H2BH	NM_003524	Homo sapiens histone cluster 1, H2bh (HIST1H2BH), mRNA [NM_003524]
0,48	0,00041	TRIM58	NM_015431	Homo sapiens tripartite motif-containing 58 (TRIM58), mRNA [NM_015431]
0,48	0,00009	HIST1H2BD	NM_021063	Homo sapiens histone cluster 1, H2bd (HIST1H2BD), transcript variant 1, mRNA [NM_021063]
0,48	0,00110	UPK3B	NM_030570	Homo sapiens uroplakin 3B (UPK3B), transcript variant 1, mRNA [NM_030570]
0,48	0,00083	LITAF	NM_004862	Homo sapiens lipopolysaccharide-induced TNF factor (LITAF), mRNA [NM_004862]
0,48	0,00005	ZC3H7B	NM_017590	Homo sapiens zinc finger CCCH-type containing 7B (ZC3H7B), mRNA [NM_017590]
0,48	0,00004	VAV1	NM_005428	Homo sapiens vav 1 oncogene (VAV1), mRNA [NM_005428]
0,48	0,00052	ZFHX2	AB051549	Homo sapiens mRNA for KIAA1762 protein, partial cds. [AB051549]
0,49	0,00012	HIST1H2BL	NM_003519	Homo sapiens histone cluster 1, H2bl (HIST1H2BL), mRNA [NM_003519]
0,49	0,00010	KAZALD1	ENST00000224809	Kazal-type serine protease inhibitor domain-containing protein 1 precursor. [Source:Uniprot/SWISSPROT;Acc:Q96I82] [ENST00000224809]
0,49	0,00018	ADRA2C	NM_000683	Homo sapiens adrenergic, alpha-2C-, receptor (ADRA2C), mRNA [NM_000683]
0,49	0,00013	HIST1H2BF	NM_003522	Homo sapiens histone cluster 1, H2bf (HIST1H2BF), mRNA [NM_003522]
0,49	0,00308	CKB	NM_001823	Homo sapiens creatine kinase, brain (CKB), mRNA [NM_001823]
0,49	0,00011	HIST1H2BO	NM_003527	Homo sapiens histone cluster 1, H2bo (HIST1H2BO), mRNA [NM_003527]

0,49	0,00103	GALNTL1	NM_020692	Homo sapiens UDP-N-acetyl-alpha-D-galactosamine:polypeptide N-acetylgalactosaminyltransferase-like 1 (GALNTL1), mRNA [NM_020692]
0,49	0,00012	DMRTC1	NM_033053	Homo sapiens DMRT-like family C1 (DMRTC1), mRNA [NM_033053]
0,49	0,00215	PSORS1C1	NM_014068	Homo sapiens psoriasis susceptibility 1 candidate 1 (PSORS1C1), mRNA [NM_014068]
0,49	0,00013	DISP1	NM_032890	Homo sapiens dispatched homolog 1 (Drosophila) (DISP1), mRNA [NM_032890]
0,49	0,00042	PTPN13	NM_080685	Homo sapiens protein tyrosine phosphatase, non-receptor type 13 (APO-1/CD95 (Fas)-associated phosphatase) (PTPN13), transcript variant 4, mRNA [NM_080685]
0,49	0,00147	KALRN	NM_003947	Homo sapiens kalirin, RhoGEF kinase (KALRN), transcript variant 2, mRNA [NM_003947]
0,49	0,00075	SLAIN1	NM_144595	Homo sapiens SLAIN motif family, member 1 (SLAIN1), transcript variant 2, mRNA [NM_144595]
0,49	0,00036	SLC13A3	NM_001011554	Homo sapiens solute carrier family 13 (sodium-dependent dicarboxylate transporter), member 3 (SLC13A3), transcript variant 2, mRNA [NM_001011554]
0,49	0,00006	KCND3	ENST00000369697	Potassium voltage-gated channel subfamily D member 3 (Voltage-gated potassium channel subunit Kv4.3). [Source:Uniprot/SWISSPROT;Acc:Q9UK17] [ENST00000369697]
0,49	0,00012	LY6E	NM_002346	Homo sapiens lymphocyte antigen 6 complex, locus E (LY6E), mRNA [NM_002346]
0,49	0,00000	DLG2	NM_001364	Homo sapiens discs, large homolog 2, chapsyn-110 (Drosophila) (DLG2), mRNA [NM_001364]
0,49	0,00020	CSTA	NM_005213	Homo sapiens cystatin A (stefin A) (CSTA), mRNA [NM_005213]
0,49	0,00809	DACT2	NM_214462	Homo sapiens dapper, antagonist of beta-catenin, homolog 2 (Xenopus laevis) (DACT2), mRNA [NM_214462]
0,50	0,00255	ITGA7	NM_002206	Homo sapiens integrin, alpha 7 (ITGA7), mRNA [NM_002206]
0,50	0,00054	ENSA	NM_207042	Homo sapiens endosulfine alpha (ENSA), transcript variant 1, mRNA [NM_207042]
0,50	0,00032	CLIC3	NM_004669	Homo sapiens chloride intracellular channel 3 (CLIC3), mRNA [NM_004669]
0,50	0,00499	PDZD2	NM_178140	Homo sapiens PDZ domain containing 2 (PDZD2), mRNA [NM_178140]
0,50	0,00019	SASH1	NM_015278	Homo sapiens SAM and SH3 domain containing 1 (SASH1), mRNA [NM_015278]
0,50	0,00009	HIST1H2BC	NM_003526	Homo sapiens histone cluster 1, H2bc (HIST1H2BC), mRNA [NM_003526]

0,50	0,00339	B4GALT1	ENST00000379731	Beta-1,4-galactosyltransferase 1 (EC 2.4.1.-) (Beta-1,4-GalTase 1) (Beta4Gal-T1) (b4Gal-T1) (UDP-galactose:beta-N-acetylglucosamine beta-1,4-galactosyltransferase 1) (UDP-Gal:beta-GlcNAc beta-1,4-galactosyltransferase 1) [Includes: Lactose synthase...
0,50	0,00075	PCDHB2	NM_018936	Homo sapiens protocadherin beta 2 (PCDHB2), mRNA [NM_018936]
0,50	0,00444	ENST00000372045	ENST00000372045	Chordin-like protein 1 precursor (Neuralin-1) (Ventroptin) (Neurogenesin-1). [Source:Uniprot/SWISSPROT;Acc:Q9BU40] [ENST00000372045]
0,50	0,00001	RTN3	NM_006054	Homo sapiens reticulon 3 (RTN3), transcript variant 1, mRNA [NM_006054]
0,50	0,00415	BTBD11	NM_152322	Homo sapiens BTB (POZ) domain containing 11 (BTBD11), transcript variant 1, mRNA [NM_152322]
0,50	0,00002	KIAA0774	NM_001033602	Homo sapiens KIAA0774 (KIAA0774), transcript variant 1, mRNA [NM_001033602]
0,50	0,00011	CYFIP2	NM_014376	Homo sapiens cytoplasmic FMR1 interacting protein 2 (CYFIP2), transcript variant 3, mRNA [NM_014376]
0,50	0,00024	LBH	NM_030915	Homo sapiens limb bud and heart development homolog (mouse) (LBH), mRNA [NM_030915]
0,50	0,00009	ZFP42	NM_174900	Homo sapiens zinc finger protein 42 homolog (mouse) (ZFP42), mRNA [NM_174900]
0,50	0,00009	WT1	NM_024424	Homo sapiens Wilms tumor 1 (WT1), transcript variant B, mRNA [NM_024424]
0,50	0,00009	GJB2	NM_004004	Homo sapiens gap junction protein, beta 2, 26kDa (GJB2), mRNA [NM_004004]
0,50	0,00536	RP6-213H19.1	NM_016542	Homo sapiens serine/threonine protein kinase MST4 (MST4), transcript variant 1, mRNA [NM_016542]
0,50	0,00011	HIST1H1C	NM_005319	Homo sapiens histone cluster 1, H1c (HIST1H1C), mRNA [NM_005319]
0,50	0,00000	NAP1L2	NM_021963	Homo sapiens nucleosome assembly protein 1-like 2 (NAP1L2), mRNA [NM_021963]

Supplementary Table S5. Microarray induced genes in Non-Epithelioid phenotype.

Ratio Non-Epithelioid	Non-Epithelioid FDR	Gene Symbol	Systematic Name	Description
19,24	0,00001	GREM1	NM_013372	Homo sapiens gremlin 1, cysteine knot superfamily, homolog (Xenopus laevis) (GREM1), mRNA [NM_013372]
15,27	0,00002	IL1B	NM_000576	Homo sapiens interleukin 1, beta (IL1B), mRNA [NM_000576]
13,97	0,00001	TFPI2	ENST00000222543	Tissue factor pathway inhibitor 2 precursor (TFPI-2) (Placental protein 5) (PP5). [Source:Uniprot/SWISSPROT;Acc:P48307] [ENST00000222543]
13,68	0,00005	ITGA11	NM_001004439	Homo sapiens integrin, alpha 11 (ITGA11), transcript variant 1, mRNA [NM_001004439]
13,43	0,00006	MMP1	NM_002421	Homo sapiens matrix metallopeptidase 1 (interstitial collagenase) (MMP1), mRNA [NM_002421]
13,05	0,00000	IL33	NM_033439	Homo sapiens interleukin 33 (IL33), mRNA [NM_033439]
11,61	0,00001	TFPI2	NM_006528	Homo sapiens tissue factor pathway inhibitor 2 (TFPI2), mRNA [NM_006528]
10,61	0,00006	CTHRC1	NM_138455	Homo sapiens collagen triple helix repeat containing 1 (CTHRC1), mRNA [NM_138455]
10,03	0,00000	MME	NM_007289	Homo sapiens membrane metallo-endopeptidase (MME), transcript variant 2b, mRNA [NM_007289]
9,73	0,00001	LUM	NM_002345	Homo sapiens lumican (LUM), mRNA [NM_002345]
9,29	0,00002	CTSK	NM_000396	Homo sapiens cathepsin K (CTSK), mRNA [NM_000396]
9,08	0,00003	MMP3	NM_002422	Homo sapiens matrix metallopeptidase 3 (stromelysin 1, progelatinase) (MMP3), mRNA [NM_002422]
8,66	0,00001	CDH13	NM_001257	Homo sapiens cadherin 13, H-cadherin (heart) (CDH13), mRNA [NM_001257]
7,73	0,00002	HMGA2	NM_003483	Homo sapiens high mobility group AT-hook 2 (HMGA2), transcript variant 1, mRNA [NM_003483]
7,66	0,00007	COL13A1	NM_005203	Homo sapiens collagen, type XIII, alpha 1 (COL13A1), transcript variant 1, mRNA [NM_005203]
7,61	0,00113	KCNE4	NM_080671	Homo sapiens potassium voltage-gated channel, Isk-related family, member 4 (KCNE4), mRNA [NM_080671]
7,23	0,00052	KRT34	NM_021013	Homo sapiens keratin 34 (KRT34), mRNA [NM_021013]
6,83	0,00000	SMYD3	NM_022743	Homo sapiens SET and MYND domain containing 3 (SMYD3), mRNA [NM_022743]

6,55	0,00000	FAP	NM_004460	Homo sapiens fibroblast activation protein, alpha (FAP), mRNA [NM_004460]
6,48	0,00007	G0S2	NM_015714	Homo sapiens G0/G1switch 2 (G0S2), mRNA [NM_015714]
6,42	0,00004	TNFAIP6	NM_007115	Homo sapiens tumor necrosis factor, alpha-induced protein 6 (TNFAIP6), mRNA [NM_007115]
6,41	0,00001	XRCC4	NM_022550	Homo sapiens X-ray repair complementing defective repair in Chinese hamster cells 4 (XRCC4), transcript variant 3, mRNA [NM_022550]
6,24	0,00004	ADAM12	NM_003474	Homo sapiens ADAM metallopeptidase domain 12 (meltrin alpha) (ADAM12), transcript variant 1, mRNA [NM_003474]
6,08	0,00004	GPC6	ENST00000377047	Glypican-6 precursor. [Source:Uniprot/SWISSPROT;Acc:Q9Y625] [ENST00000377047]
5,99	0,00001	PLAU	NM_002658	Homo sapiens plasminogen activator, urokinase (PLAU), mRNA [NM_002658]
5,93	0,00014	COL6A3	NM_004369	Homo sapiens collagen, type VI, alpha 3 (COL6A3), transcript variant 1, mRNA [NM_004369]
5,88	0,00011	LIMS3	NM_033514	Homo sapiens LIM and senescent cell antigen-like domains 3 (LIMS3), mRNA [NM_033514]
5,85	0,00012	ABI3BP	NM_015429	Homo sapiens ABI gene family, member 3 (NESH) binding protein (ABI3BP), mRNA [NM_015429]
5,69	0,00042	THBS1	NM_003246	Homo sapiens thrombospondin 1 (THBS1), mRNA [NM_003246]
5,57	0,00286	CLDN14	NM_144492	Homo sapiens claudin 14 (CLDN14), transcript variant 1, mRNA [NM_144492]
5,50	0,00013	PID1	NM_017933	Homo sapiens phosphotyrosine interaction domain containing 1 (PID1), mRNA [NM_017933]
5,35	0,00001	PHLDA1	NM_007350	Homo sapiens pleckstrin homology-like domain, family A, member 1 (PHLDA1), mRNA [NM_007350]
5,32	0,00013	VCAN	NM_004385	Homo sapiens versican (VCAN), mRNA [NM_004385]
5,26	0,00029	ANGPTL4	NM_139314	Homo sapiens angiopoietin-like 4 (ANGPTL4), transcript variant 1, mRNA [NM_139314]
5,05	0,00001	DCN	NM_001920	Homo sapiens decorin (DCN), transcript variant A1, mRNA [NM_001920]
5,00	0,00007	TNFSF4	NM_003326	Homo sapiens tumor necrosis factor (ligand) superfamily, member 4 (tax-transcriptionally activated glycoprotein 1, 34kDa) (TNFSF4), mRNA [NM_003326]
4,94	0,00001	OSBPL6	NM_032523	Homo sapiens oxysterol binding protein-like 6 (OSBPL6), transcript variant 1, mRNA [NM_032523]
4,76	0,00000	MLPH	NM_024101	Homo sapiens melanophilin (MLPH), transcript variant 1, mRNA [NM_024101]

4,67	0,00001	FOSB	NM_006732	Homo sapiens FBJ murine osteosarcoma viral oncogene homolog B (FOSB), mRNA [NM_006732]
4,66	0,00001	GDF15	NM_004864	Homo sapiens growth differentiation factor 15 (GDF15), mRNA [NM_004864]
4,59	0,00026	SLC2A1	NM_006516	Homo sapiens solute carrier family 2 (facilitated glucose transporter), member 1 (SLC2A1), mRNA [NM_006516]
4,55	0,00000	PHLDA2	NM_003311	Homo sapiens pleckstrin homology-like domain, family A, member 2 (PHLDA2), mRNA [NM_003311]
4,53	0,00003	MTHFD2	NM_006636	Homo sapiens methylenetetrahydrofolate dehydrogenase (NADP+ dependent) 2, methenyltetrahydrofolate cyclohydrolase (MTHFD2), nuclear gene encoding mitochondrial protein, transcript variant 1, mRNA [NM_006636]
4,47	0,00000	CD44	NM_000610	Homo sapiens CD44 molecule (Indian blood group) (CD44), transcript variant 1, mRNA [NM_000610]
4,46	0,00001	F2R	NM_001992	Homo sapiens coagulation factor II (thrombin) receptor (F2R), mRNA [NM_001992]
4,43	0,00027	THBS2	L12350	Human thrombospondin 2 (THBS2) mRNA, complete cds. [L12350]
4,37	0,00020	HNT	NM_016522	Homo sapiens neurotrimin (HNT), transcript variant 1, mRNA [NM_016522]
4,29	0,00017	CNIH3	NM_152495	Homo sapiens cornichon homolog 3 (Drosophila) (CNIH3), mRNA [NM_152495]
4,25	0,00071	MMP9	NM_004994	Homo sapiens matrix metallopeptidase 9 (gelatinase B, 92kDa gelatinase, 92kDa type IV collagenase) (MMP9), mRNA [NM_004994]
4,20	0,00105	NEFM	NM_005382	Homo sapiens neurofilament, medium polypeptide 150kDa (NEFM), mRNA [NM_005382]
4,17	0,00020	HSD11B1	NM_181755	Homo sapiens hydroxysteroid (11-beta) dehydrogenase 1 (HSD11B1), transcript variant 2, mRNA [NM_181755]
4,16	0,00009	TNFRSF6B	NM_032945	Homo sapiens tumor necrosis factor receptor superfamily, member 6b, decoy (TNFRSF6B), transcript variant M68C, mRNA [NM_032945]
4,09	0,00006	TGFBI	NM_000358	Homo sapiens transforming growth factor, beta-induced, 68kDa (TGFBI), mRNA [NM_000358]
4,06	0,00000	SAMD3	NM_001017373	Homo sapiens sterile alpha motif domain containing 3 (SAMD3), transcript variant 1, mRNA [NM_001017373]
4,04	0,00145	SPON2	NM_012445	Homo sapiens spondin 2, extracellular matrix protein (SPON2), mRNA [NM_012445]
4,03	0,00032	ITGBL1	NM_004791	Homo sapiens integrin, beta-like 1 (with EGF-like repeat domains) (ITGBL1), mRNA [NM_004791]
4,03	0,00051	PTGS2	NM_000963	Homo sapiens prostaglandin-endoperoxide synthase 2 (prostaglandin G/H synthase and cyclooxygenase) (PTGS2), mRNA [NM_000963]

3,97	0,00003	VDR	NM_001017535	Homo sapiens vitamin D (1,25- dihydroxyvitamin D3) receptor (VDR), transcript variant 2, mRNA [NM_001017535]
3,96	0,00001	TRPV2	NM_016113	Homo sapiens transient receptor potential cation channel, subfamily V, member 2 (TRPV2), mRNA [NM_016113]
3,96	0,00002	DOCK10	NM_014689	Homo sapiens dedicator of cytokinesis 10 (DOCK10), mRNA [NM_014689]
3,94	0,00008	SULF1	NM_015170	Homo sapiens sulfatase 1 (SULF1), mRNA [NM_015170]
3,92	0,00000	KIAA1212	NM_018084	Homo sapiens KIAA1212 (KIAA1212), mRNA [NM_018084]
3,86	0,00067	FHOD3	NM_025135	Homo sapiens formin homology 2 domain containing 3 (FHOD3), mRNA [NM_025135]
3,84	0,00002	ACTG2	NM_001615	Homo sapiens actin, gamma 2, smooth muscle, enteric (ACTG2), mRNA [NM_001615]
3,82	0,00017	LPXN	NM_004811	Homo sapiens leupaxin (LPXN), mRNA [NM_004811]
3,81	0,00263	GPAM	NM_020918	Homo sapiens glycerol-3-phosphate acyltransferase, mitochondrial (GPAM), mRNA [NM_020918]
3,79	0,00009	ELTD1	BC025721	Homo sapiens EGF, latrophilin and seven transmembrane domain containing 1, mRNA (cDNA clone MGC:34204 IMAGE:5229055), complete cds. [BC025721]
3,75	0,00000	POPDC3	NM_022361	Homo sapiens popeye domain containing 3 (POPDC3), mRNA [NM_022361]
3,72	0,00006	ARHGAP22	NM_021226	Homo sapiens Rho GTPase activating protein 22 (ARHGAP22), mRNA [NM_021226]
3,70	0,00071	ANXA10	NM_007193	Homo sapiens annexin A10 (ANXA10), mRNA [NM_007193]
3,69	0,00001	PAG1	NM_018440	Homo sapiens phosphoprotein associated with glycosphingolipid microdomains 1 (PAG1), mRNA [NM_018440]
3,68	0,00003	DKK1	NM_012242	Homo sapiens dickkopf homolog 1 (Xenopus laevis) (DKK1), mRNA [NM_012242]
3,68	0,00001	STK17B	NM_004226	Homo sapiens serine/threonine kinase 17b (STK17B), mRNA [NM_004226]
3,68	0,00003	SRPX	NM_006307	Homo sapiens sushi-repeat-containing protein, X-linked (SRPX), mRNA [NM_006307]
3,64	0,00056	EMP1	BC017854	Homo sapiens epithelial membrane protein 1, mRNA (cDNA clone IMAGE:4691099), partial cds. [BC017854]
3,59	0,00034	THBS2	NM_003247	Homo sapiens thrombospondin 2 (THBS2), mRNA [NM_003247]
3,58	0,00002	SNAI2	NM_003068	Homo sapiens snail homolog 2 (Drosophila) (SNAI2), mRNA [NM_003068]
3,58	0,00003	IL11	NM_000641	Homo sapiens interleukin 11 (IL11), mRNA [NM_000641]
3,57	0,00002	RGS4	NM_005613	Homo sapiens regulator of G-protein signalling 4 (RGS4), mRNA [NM_005613]

3,57	0,00168	SPP1	NM_000582	Homo sapiens secreted phosphoprotein 1 (osteopontin, bone sialoprotein I, early T-lymphocyte activation 1) (SPP1), transcript variant 2, mRNA [NM_000582]
3,56	0,00086	CCDC85A	NM_001080433	Homo sapiens coiled-coil domain containing 85A (CCDC85A), mRNA [NM_001080433]
3,55	0,00070	E2F7	NM_203394	Homo sapiens E2F transcription factor 7 (E2F7), mRNA [NM_203394]
3,55	0,00012	PBEF1	NM_005746	Homo sapiens pre-B-cell colony enhancing factor 1 (PBEF1), mRNA [NM_005746]
3,54	0,00011	EVI2A	NM_001003927	Homo sapiens ecotropic viral integration site 2A (EVI2A), transcript variant 1, mRNA [NM_001003927]
3,52	0,00011	DCBLD2	NM_080927	Homo sapiens discoidin, CUB and LCCL domain containing 2 (DCBLD2), mRNA [NM_080927]
3,50	0,00000	STEAP1	NM_012449	Homo sapiens six transmembrane epithelial antigen of the prostate 1 (STEAP1), mRNA [NM_012449]
3,48	0,00170	LTBP1	NM_206943	Homo sapiens latent transforming growth factor beta binding protein 1 (LTBP1), transcript variant 1, mRNA [NM_206943]
3,48	0,00000	HNMT	NM_006895	Homo sapiens histamine N-methyltransferase (HNMT), transcript variant 1, mRNA [NM_006895]
3,47	0,00017	MST150	NM_032947	Homo sapiens MSTP150 (MST150), mRNA [NM_032947]
3,47	0,00004	TncRNA	NR_002802	Homo sapiens trophoblast-derived noncoding RNA (TncRNA) on chromosome 11 [NR_002802]
3,46	0,00112	AMIGO2	NM_181847	Homo sapiens adhesion molecule with Ig-like domain 2 (AMIGO2), mRNA [NM_181847]
3,45	0,00000	SLC22A4	NM_003059	Homo sapiens solute carrier family 22 (organic cation transporter), member 4 (SLC22A4), mRNA [NM_003059]
3,45	0,00004	MMP2	NM_004530	Homo sapiens matrix metallopeptidase 2 (gelatinase A, 72kDa gelatinase, 72kDa type IV collagenase) (MMP2), mRNA [NM_004530]
3,45	0,00042	SERPINE2	NM_006216	Homo sapiens serpin peptidase inhibitor, clade E (nexin, plasminogen activator inhibitor type 1), member 2 (SERPINE2), mRNA [NM_006216]
3,44	0,00006	FAM129A	NM_052966	Homo sapiens family with sequence similarity 129, member A (FAM129A), transcript variant 2, mRNA [NM_052966]
3,43	0,00461	PTX3	NM_002852	Homo sapiens pentraxin-related gene, rapidly induced by IL-1 beta (PTX3), mRNA [NM_002852]
3,41	0,00017	PPP1R3C	NM_005398	Homo sapiens protein phosphatase 1, regulatory (inhibitor) subunit 3C (PPP1R3C), mRNA [NM_005398]
3,29	0,00033	TSLP	NM_033035	Homo sapiens thymic stromal lymphopoietin (TSLP), transcript variant 1, mRNA [NM_033035]

3,28	0,00002	PRNP	NM_000311	Homo sapiens prion protein (p27-30) (Creutzfeldt-Jakob disease, Gerstmann-Strausler-Scheinker syndrome, fatal familial insomnia) (PRNP), transcript variant 1, mRNA [NM_000311]
3,27	0,00346	POSTN	NM_006475	Homo sapiens periostin, osteoblast specific factor (POSTN), mRNA [NM_006475]
3,24	0,00476	CYP1B1	NM_000104	Homo sapiens cytochrome P450, family 1, subfamily B, polypeptide 1 (CYP1B1), mRNA [NM_000104]
3,22	0,00001	ECM1	NM_004425	Homo sapiens extracellular matrix protein 1 (ECM1), transcript variant 1, mRNA [NM_004425]
3,21	0,00159	S100A4	NM_002961	Homo sapiens S100 calcium binding protein A4 (S100A4), transcript variant 1, mRNA [NM_002961]
3,21	0,00038	HTR2B	NM_000867	Homo sapiens 5-hydroxytryptamine (serotonin) receptor 2B (HTR2B), mRNA [NM_000867]
3,20	0,00008	KLHDC7B	NM_138433	Homo sapiens kelch domain containing 7B (KLHDC7B), mRNA [NM_138433]
3,14	0,00007	KIAA1913	BC044246	Homo sapiens KIAA1913, mRNA (cDNA clone MGC:50847 IMAGE:5760073), complete cds. [BC044246]
3,13	0,00000	GEM	NM_005261	Homo sapiens GTP binding protein overexpressed in skeletal muscle (GEM), transcript variant 1, mRNA [NM_005261]
3,11	0,00029	CSPG4	NM_001897	Homo sapiens chondroitin sulfate proteoglycan 4 (CSPG4), mRNA [NM_001897]
3,11	0,00000	GNG11	NM_004126	Homo sapiens guanine nucleotide binding protein (G protein), gamma 11 (GNG11), mRNA [NM_004126]
3,08	0,00060	ITGA2	NM_002203	Homo sapiens integrin, alpha 2 (CD49B, alpha 2 subunit of VLA-2 receptor) (ITGA2), mRNA [NM_002203]
3,08	0,00002	CDC42BPA	NM_014826	Homo sapiens CDC42 binding protein kinase alpha (DMPK-like) (CDC42BPA), transcript variant A, mRNA [NM_014826]
3,08	0,00000	ITGB1	NM_002211	Homo sapiens integrin, beta 1 (fibronectin receptor, beta polypeptide, antigen CD29 includes MDF2, MSK12) (ITGB1), transcript variant 1A, mRNA [NM_002211]
3,04	0,00009	SHC3	NM_016848	Homo sapiens SHC (Src homology 2 domain containing) transforming protein 3 (SHC3), mRNA [NM_016848]
3,03	0,00101	COL1A2	NM_000089	Homo sapiens collagen, type I, alpha 2 (COL1A2), mRNA [NM_000089]
3,03	0,00021	PSAT1	NM_058179	Homo sapiens phosphoserine aminotransferase 1 (PSAT1), transcript variant 1, mRNA [NM_058179]
3,01	0,00046	ABCG2	NM_004827	Homo sapiens ATP-binding cassette, sub-family G (WHITE), member 2 (ABCG2), mRNA [NM_004827]

3,01	0,00052	ENST00000376155	ENST00000376155	integrin, beta-like 1 (with EGF-like repeat domains) [Source:RefSeq_peptide;Acc:NP_004782] [ENST00000376155]
3,01	0,00002	HS3ST3A1	NM_006042	Homo sapiens heparan sulfate (glucosamine) 3-O-sulfotransferase 3A1 (HS3ST3A1), mRNA [NM_006042]
3,01	0,00008	ABCC3	NM_003786	Homo sapiens ATP-binding cassette, sub-family C (CFTR/MRP), member 3 (ABCC3), mRNA [NM_003786]
3,01	0,00002	NRP1	NM_003873	Homo sapiens neuropilin 1 (NRP1), transcript variant 1, mRNA [NM_003873]
2,99	0,00032	EDIL3	NM_005711	Homo sapiens EGF-like repeats and discoidin I-like domains 3 (EDIL3), mRNA [NM_005711]
2,98	0,00002	GPR68	NM_003485	Homo sapiens G protein-coupled receptor 68 (GPR68), mRNA [NM_003485]
2,97	0,00006	DUSP6	NM_001946	Homo sapiens dual specificity phosphatase 6 (DUSP6), transcript variant 1, mRNA [NM_001946]
2,97	0,00012	UGCG	ENST00000374279	Ceramide glucosyltransferase (EC 2.4.1.80) (Glucosylceramide synthase) (GCS) (UDP-glucose:N-acylsphingosine D-glucosyltransferase) (UDP-glucose ceramide glucosyltransferase) (GLCT-1). [Source:Uniprot/SWISSPROT;Acc:Q16739] [ENST00000374279]
2,96	0,00000	SRGAP1	BC029919	Homo sapiens SLIT-ROBO Rho GTPase activating protein 1, mRNA (cDNA clone IMAGE:4153945), with apparent retained intron. [BC029919]
2,94	0,00505	PPM2C	NM_018444	Homo sapiens protein phosphatase 2C, magnesium-dependent, catalytic subunit (PPM2C), nuclear gene encoding mitochondrial protein, mRNA [NM_018444]
2,93	0,00007	FGF2	NM_002006	Homo sapiens fibroblast growth factor 2 (basic) (FGF2), mRNA [NM_002006]
2,92	0,00037	VEGFA	NM_003376	Homo sapiens vascular endothelial growth factor A (VEGFA), transcript variant 2, mRNA [NM_003376]
2,92	0,00040	COL8A2	NM_005202	Homo sapiens collagen, type VIII, alpha 2 (COL8A2), mRNA [NM_005202]
2,92	0,00106	TEK	NM_000459	Homo sapiens TEK tyrosine kinase, endothelial (venous malformations, multiple cutaneous and mucosal) (TEK), mRNA [NM_000459]
2,90	0,00020	LOC401152	NM_001001701	Homo sapiens HCV F-transactivated protein 1 (LOC401152), mRNA [NM_001001701]
2,89	0,00002	HRH1	NM_000861	Homo sapiens histamine receptor H1 (HRH1), mRNA [NM_000861]
2,89	0,00003	ITGB1	AF086249	Homo sapiens full length insert cDNA clone ZD39G09. [AF086249]

2,89	0,00245	TDO2	NM_005651	Homo sapiens tryptophan 2,3-dioxygenase (TDO2), mRNA [NM_005651]
2,88	0,00003	SLC19A2	NM_006996	Homo sapiens solute carrier family 19 (thiamine transporter), member 2 (SLC19A2), mRNA [NM_006996]
2,88	0,00005	KIAA1913	NM_052913	Homo sapiens KIAA1913 (KIAA1913), mRNA [NM_052913]
2,88	0,00212	PITX2	NM_153426	Homo sapiens paired-like homeodomain transcription factor 2 (PITX2), transcript variant 2, mRNA [NM_153426]
2,86	0,00005	ADAMTS4	NM_005099	Homo sapiens ADAM metallopeptidase with thrombospondin type 1 motif, 4 (ADAMTS4), mRNA [NM_005099]
2,86	0,00002	ITGB1BP2	NM_012278	Homo sapiens integrin beta 1 binding protein (melusin) 2 (ITGB1BP2), mRNA [NM_012278]
2,86	0,00021	KIAA1462	ENST00000375377	Novel protein. [Source:Uniprot/SPTREMBL;Acc:Q5T992] [ENST00000375377]
2,84	0,00224	IFI30	NM_006332	Homo sapiens interferon, gamma-inducible protein 30 (IFI30), mRNA [NM_006332]
2,84	0,00219	DACT1	NM_016651	Homo sapiens dapper, antagonist of beta-catenin, homolog 1 (Xenopus laevis) (DACT1), transcript variant 1, mRNA [NM_016651]
2,83	0,00002	KCNJ15	NM_170736	Homo sapiens potassium inwardly-rectifying channel, subfamily J, member 15 (KCNJ15), transcript variant 1, mRNA [NM_170736]
2,82	0,00030	EMP1	NM_001423	Homo sapiens epithelial membrane protein 1 (EMP1), mRNA [NM_001423]
2,81	0,00001	ASAM	NM_024769	Homo sapiens adipocyte-specific adhesion molecule (ASAM), mRNA [NM_024769]
2,81	0,00004	DLEU1	NR_002605	Homo sapiens deleted in lymphocytic leukemia, 1 (DLEU1) on chromosome 13 [NR_002605]
2,81	0,00020	HS3ST3B1	ENST00000360954	Heparan sulfate glucosamine 3-O-sulfotransferase 3B1 (EC 2.8.2.30) (Heparan sulfate D-glucosaminyl 3-O-sulfotransferase 3B1) (Heparan sulfate 3-O-sulfotransferase 3B1) (h3-OST-3B). [Source:Uniprot/SWISSPROT;Acc:Q9Y662] [ENST00000360954]
2,80	0,00000	NAV3	NM_014903	Homo sapiens neuron navigator 3 (NAV3), mRNA [NM_014903]
2,79	0,00003	NT5E	NM_002526	Homo sapiens 5'-nucleotidase, ecto (CD73) (NT5E), mRNA [NM_002526]
2,78	0,00017	NP	NM_000270	Homo sapiens nucleoside phosphorylase (NP), mRNA [NM_000270]
2,77	0,00017	EFEMP1	NM_004105	Homo sapiens EGF-containing fibulin-like extracellular matrix protein 1 (EFEMP1), transcript variant 1, mRNA [NM_004105]
2,75	0,00003	BAIAP2L2	NM_025045	Homo sapiens BAI1-associated protein 2-like 2 (BAIAP2L2), mRNA [NM_025045]
2,73	0,00005	RGMB	NM_001012761	Homo sapiens RGM domain family, member B (RGMB), transcript variant 1, mRNA [NM_001012761]

2,73	0,00058	IL1A	NM_000575	Homo sapiens interleukin 1, alpha (IL1A), mRNA [NM_000575]
2,70	0,00096	COL7A1	NM_000094	Homo sapiens collagen, type VII, alpha 1 (epidermolysis bullosa, dystrophic, dominant and recessive) (COL7A1), mRNA [NM_000094]
2,70	0,00030	FBN1	NM_000138	Homo sapiens fibrillin 1 (FBN1), mRNA [NM_000138]
2,69	0,00011	YIPF5	NM_030799	Homo sapiens Yip1 domain family, member 5 (YIPF5), transcript variant 2, mRNA [NM_030799]
2,69	0,00001	RALA	NM_005402	Homo sapiens v-ral simian leukemia viral oncogene homolog A (ras related) (RALA), mRNA [NM_005402]
2,69	0,00002	SPHK1	NM_021972	Homo sapiens sphingosine kinase 1 (SPHK1), transcript variant 1, mRNA [NM_021972]
2,67	0,00000	PTPRB	NM_002837	Homo sapiens protein tyrosine phosphatase, receptor type, B (PTPRB), mRNA [NM_002837]
2,66	0,00057	LRIG1	NM_015541	Homo sapiens leucine-rich repeats and immunoglobulin-like domains 1 (LRIG1), mRNA [NM_015541]
2,66	0,00087	COL16A1	NM_001856	Homo sapiens collagen, type XVI, alpha 1 (COL16A1), mRNA [NM_001856]
2,66	0,00003	DGKI	NM_004717	Homo sapiens diacylglycerol kinase, iota (DGKI), mRNA [NM_004717]
2,63	0,00289	TFPI	NM_001032281	Homo sapiens tissue factor pathway inhibitor (lipoprotein-associated coagulation inhibitor) (TFPI), transcript variant 2, mRNA [NM_001032281]
2,62	0,00006	HIF1A	NM_181054	Homo sapiens hypoxia-inducible factor 1, alpha subunit (basic helix-loop-helix transcription factor) (HIF1A), transcript variant 2, mRNA [NM_181054]
2,62	0,00003	ST3GAL6	NM_006100	Homo sapiens ST3 beta-galactoside alpha-2,3-sialyltransferase 6 (ST3GAL6), mRNA [NM_006100]
2,62	0,00097	RAB27B	NM_004163	Homo sapiens RAB27B, member RAS oncogene family (RAB27B), mRNA [NM_004163]
2,60	0,00002	HNMT	NM_001024074	Homo sapiens histamine N-methyltransferase (HNMT), transcript variant 2, mRNA [NM_001024074]
2,59	0,00092	MPP4	NM_033066	Homo sapiens membrane protein, palmitoylated 4 (MAGUK p55 subfamily member 4) (MPP4), mRNA [NM_033066]
2,59	0,00478	SLC2A5	NM_003039	Homo sapiens solute carrier family 2 (facilitated glucose/fructose transporter), member 5 (SLC2A5), mRNA [NM_003039]
2,59	0,00040	GBE1	NM_000158	Homo sapiens glucan (1,4-alpha-), branching enzyme 1 (glycogen branching enzyme, Andersen disease, glycogen storage disease type IV) (GBE1), mRNA [NM_000158]
2,58	0,00001	GOLGA4	NM_002078	Homo sapiens golgi autoantigen, golgin subfamily a, 4 (GOLGA4), mRNA [NM_002078]

2,57	0,00019	KCNN4	NM_002250	Homo sapiens potassium intermediate/small conductance calcium-activated channel, subfamily N, member 4 (KCNN4), mRNA [NM_002250]
2,57	0,00003	ADORA2B	NM_000676	Homo sapiens adenosine A2b receptor (ADORA2B), mRNA [NM_000676]
2,57	0,00017	MYB	NM_005375	Homo sapiens v-myb myeloblastosis viral oncogene homolog (avian) (MYB), mRNA [NM_005375]
2,57	0,00144	CAV1	NM_001753	Homo sapiens caveolin 1, caveolae protein, 22kDa (CAV1), mRNA [NM_001753]
2,56	0,00008	SLC7A11	NM_014331	Homo sapiens solute carrier family 7, (cationic amino acid transporter, y+ system) member 11 (SLC7A11), mRNA [NM_014331]
2,56	0,00195	INHBA	NM_002192	Homo sapiens inhibin, beta A (activin A, activin AB alpha polypeptide) (INHBA), mRNA [NM_002192]
2,55	0,00011	PTPN22	NM_015967	Homo sapiens protein tyrosine phosphatase, non-receptor type 22 (lymphoid) (PTPN22), transcript variant 1, mRNA [NM_015967]
2,55	0,00722	IL6	NM_000600	Homo sapiens interleukin 6 (interferon, beta 2) (IL6), mRNA [NM_000600]
2,55	0,00070	VIM	NM_003380	Homo sapiens vimentin (VIM), mRNA [NM_003380]
2,55	0,00001	ADAMTS6	NM_197941	Homo sapiens ADAM metallopeptidase with thrombospondin type 1 motif, 6 (ADAMTS6), mRNA [NM_197941]
2,54	0,00001	SP100	L79989	Homo sapiens nuclear autoantigen mRNA, partial cds; alternatively spliced. [L79989]
2,53	0,00034	FAM46A	NM_017633	Homo sapiens family with sequence similarity 46, member A (FAM46A), mRNA [NM_017633]
2,53	0,00650	EGR2	NM_000399	Homo sapiens early growth response 2 (Krox-20 homolog, Drosophila) (EGR2), mRNA [NM_000399]
2,52	0,00004	NRIP1	NM_003489	Homo sapiens nuclear receptor interacting protein 1 (NRIP1), mRNA [NM_003489]
2,52	0,00003	TXN	NM_003329	Homo sapiens thioredoxin (TXN), mRNA [NM_003329]
2,50	0,00067	U25029	U25029	Human glucocorticoid receptor alpha mRNA, variant 3' UTR. [U25029]
2,50	0,00000	DKK3	NM_015881	Homo sapiens dickkopf homolog 3 (Xenopus laevis) (DKK3), transcript variant 1, mRNA [NM_015881]
2,49	0,00014	MYOHD1	NM_001033580	Homo sapiens myosin head domain containing 1 (MYOHD1), transcript variant 3, mRNA [NM_001033580]
2,48	0,00031	SLC20A1	NM_005415	Homo sapiens solute carrier family 20 (phosphate transporter), member 1 (SLC20A1), mRNA [NM_005415]
2,47	0,00037	LZTS1	NM_021020	Homo sapiens leucine zipper, putative tumor suppressor 1 (LZTS1), mRNA [NM_021020]

2,47	0,00005	UPP1	BC047030	Homo sapiens uridine phosphorylase 1, mRNA (cDNA clone MGC:54255 IMAGE:5549432), complete cds. [BC047030]
2,47	0,00267	MANBA	NM_005908	Homo sapiens mannosidase, beta A, lysosomal (MANBA), mRNA [NM_005908]
2,46	0,00074	SLC22A3	NM_021977	Homo sapiens solute carrier family 22 (extraneuronal monoamine transporter), member 3 (SLC22A3), mRNA [NM_021977]
2,46	0,00000	CHSY-2	NM_175856	Homo sapiens chondroitin synthase-2 (CHSY-2), mRNA [NM_175856]
2,45	0,00759	NOTCH2NL	NM_203458	Homo sapiens Notch homolog 2 (Drosophila) N-terminal like (NOTCH2NL), mRNA [NM_203458]
2,45	0,00004	SNAPC1	NM_003082	Homo sapiens small nuclear RNA activating complex, polypeptide 1, 43kDa (SNAPC1), mRNA [NM_003082]
2,44	0,00041	WNT5B	NM_030775	Homo sapiens wingless-type MMTV integration site family, member 5B (WNT5B), transcript variant 2, mRNA [NM_030775]
2,44	0,00002	TMEM55A	NM_018710	Homo sapiens transmembrane protein 55A (TMEM55A), mRNA [NM_018710]
2,44	0,00018	PDGFRA	NM_006206	Homo sapiens platelet-derived growth factor receptor, alpha polypeptide (PDGFRA), mRNA [NM_006206]
2,44	0,00001	KLF12	NM_007249	Homo sapiens Kruppel-like factor 12 (KLF12), mRNA [NM_007249]
2,44	0,00264	CDCP1	NM_178181	Homo sapiens CUB domain containing protein 1 (CDCP1), transcript variant 2, mRNA [NM_178181]
2,43	0,00130	FOS	NM_005252	Homo sapiens v-fos FBJ murine osteosarcoma viral oncogene homolog (FOS), mRNA [NM_005252]
2,43	0,00014	NR3C1	NM_000176	Homo sapiens nuclear receptor subfamily 3, group C, member 1 (glucocorticoid receptor) (NR3C1), transcript variant 5, mRNA [NM_000176]
2,42	0,00000	GREM2	NM_022469	Homo sapiens gremlin 2, cysteine knot superfamily, homolog (Xenopus laevis) (GREM2), mRNA [NM_022469]
2,42	0,00008	ARL6IP6	NM_152522	Homo sapiens ADP-ribosylation-like factor 6 interacting protein 6 (ARL6IP6), mRNA [NM_152522]
2,41	0,00105	SNAI1	NM_005985	Homo sapiens snail homolog 1 (Drosophila) (SNAI1), mRNA [NM_005985]
2,41	0,00015	B3GNT5	NM_032047	Homo sapiens UDP-GlcNAc:betaGal beta-1,3-N-acetylglucosaminyltransferase 5 (B3GNT5), mRNA [NM_032047]
2,41	0,00142	TMEM132B	NM_052907	Homo sapiens transmembrane protein 132B (TMEM132B), mRNA [NM_052907]
2,41	0,00037	TPST1	NM_003596	Homo sapiens tyrosylprotein sulfotransferase 1 (TPST1), mRNA [NM_003596]

2,40	0,00015	KCNMA1	NM_001014797	Homo sapiens potassium large conductance calcium-activated channel, subfamily M, alpha member 1 (KCNMA1), transcript variant 1, mRNA [NM_001014797]
2,39	0,00000	SCLT1	NM_144643	Homo sapiens sodium channel and clathrin linker 1 (SCLT1), mRNA [NM_144643]
2,38	0,00000	ITGA4	NM_000885	Homo sapiens integrin, alpha 4 (antigen CD49D, alpha 4 subunit of VLA-4 receptor) (ITGA4), mRNA [NM_000885]
2,38	0,00019	CFLAR	AF009616	Homo sapiens FLAME-1 mRNA, complete cds. [AF009616]
2,38	0,00005	FRMPD4	ENST00000380682	PDZ domain containing 10 [Source:RefSeq_peptide;Acc:NP_055543] [ENST00000380682]
2,38	0,00015	CV573177	CV573177	od33b11.y1 Human keratoconus cornea, unamplified, (od [CV573177]
2,38	0,00128	NUAK1	NM_014840	Homo sapiens NUAK family, SNF1-like kinase, 1 (NUAK1), mRNA [NM_014840]
2,37	0,00030	EPGN	NM_001013442	Homo sapiens epithelial mitogen homolog (mouse) (EPGN), mRNA [NM_001013442]
2,37	0,00612	TFPI	NM_006287	Homo sapiens tissue factor pathway inhibitor (lipoprotein-associated coagulation inhibitor) (TFPI), transcript variant 1, mRNA [NM_006287]
2,37	0,00003	FTL	NM_000146	Homo sapiens ferritin, light polypeptide (FTL), mRNA [NM_000146]
2,35	0,00008	UCHL1	NM_004181	Homo sapiens ubiquitin carboxyl-terminal esterase L1 (ubiquitin thiolesterase) (UCHL1), mRNA [NM_004181]
2,35	0,00007	ITGB1	NM_133376	Homo sapiens integrin, beta 1 (fibronectin receptor, beta polypeptide, antigen CD29 includes MDF2, MSK12) (ITGB1), transcript variant 1E, mRNA [NM_133376]
2,35	0,00141	CXCL2	NM_002089	Homo sapiens chemokine (C-X-C motif) ligand 2 (CXCL2), mRNA [NM_002089]
2,33	0,00000	LEPREL2	NM_014262	Homo sapiens leprecan-like 2 (LEPREL2), mRNA [NM_014262]
2,33	0,00018	MT2A	NM_005953	Homo sapiens metallothionein 2A (MT2A), mRNA [NM_005953]
2,33	0,00003	RHOJ	NM_020663	Homo sapiens ras homolog gene family, member J (RHOJ), mRNA [NM_020663]
2,32	0,00042	PDGFA	NM_002607	Homo sapiens platelet-derived growth factor alpha polypeptide (PDGFA), transcript variant 1, mRNA [NM_002607]
2,32	0,00043	CAV3	NM_001234	Homo sapiens caveolin 3 (CAV3), transcript variant 2, mRNA [NM_001234]
2,32	0,00095	ENST00000288911	ENST00000288911	Ankyrin repeat domain-containing protein 36A. [Source:Uniprot/SWISSPROT;Acc:Q6UX02] [ENST00000288911]
2,31	0,00888	XG	NM_175569	Homo sapiens Xg blood group (XG), mRNA [NM_175569]
2,31	0,00021	PFKP	NM_002627	Homo sapiens phosphofructokinase, platelet (PFKP), mRNA [NM_002627]

2,30	0,00255	WISP1	NM_003882	Homo sapiens WNT1 inducible signaling pathway protein 1 (WISP1), transcript variant 1, mRNA [NM_003882]
2,30	0,00033	CCPG1	AF011794	Homo sapiens cell cycle progression restoration 8 protein (CPR8) mRNA, complete cds. [AF011794]
2,29	0,00015	CCPG1	NM_004748	Homo sapiens cell cycle progression 1 (CCPG1), transcript variant 1, mRNA [NM_004748]
2,29	0,00092	SIRPG	NM_080816	Homo sapiens signal-regulatory protein gamma (SIRPG), transcript variant 2, mRNA [NM_080816]
2,29	0,00036	VEGFA	NM_001025366	Homo sapiens vascular endothelial growth factor A (VEGFA), transcript variant 1, mRNA [NM_001025366]
2,29	0,00016	PLA2G4A	NM_024420	Homo sapiens phospholipase A2, group IVA (cytosolic, calcium-dependent) (PLA2G4A), mRNA [NM_024420]
2,29	0,00071	ARNTL2	AF256215	Homo sapiens cycle-like factor CLIF mRNA, complete cds. [AF256215]
2,29	0,00037	LAMB3	NM_001017402	Homo sapiens laminin, beta 3 (LAMB3), transcript variant 2, mRNA [NM_001017402]
2,29	0,00003	TNS1	NM_022648	Homo sapiens tensin 1 (TNS1), mRNA [NM_022648]
2,29	0,00005	LOXL2	NM_002318	Homo sapiens lysyl oxidase-like 2 (LOXL2), mRNA [NM_002318]
2,28	0,00001	SLC22A15	NM_018420	Homo sapiens solute carrier family 22 (organic cation transporter), member 15 (SLC22A15), mRNA [NM_018420]
2,28	0,00000	ANGPT1	NM_001146	Homo sapiens angiopoietin 1 (ANGPT1), mRNA [NM_001146]
2,28	0,00202	CENPK	NM_022145	Homo sapiens centromere protein K (CENPK), mRNA [NM_022145]
2,28	0,00006	UACA	NM_001008224	Homo sapiens uveal autoantigen with coiled-coil domains and ankyrin repeats (UACA), transcript variant 2, mRNA [NM_001008224]
2,27	0,00151	LMO7	NM_005358	Homo sapiens LIM domain 7 (LMO7), mRNA [NM_005358]
2,27	0,00566	STC2	NM_003714	Homo sapiens stanniocalcin 2 (STC2), mRNA [NM_003714]
2,27	0,00493	GPR4	NM_005282	Homo sapiens G protein-coupled receptor 4 (GPR4), mRNA [NM_005282]
2,27	0,00223	VWCE	NM_152718	Homo sapiens von Willebrand factor C and EGF domains (VWCE), mRNA [NM_152718]
2,26	0,00206	ARHGDIB	NM_001175	Homo sapiens Rho GDP dissociation inhibitor (GDI) beta (ARHGDIB), mRNA [NM_001175]
2,26	0,00012	VEPH1	NM_024621	Homo sapiens ventricular zone expressed PH domain homolog 1 (zebrafish) (VEPH1), mRNA [NM_024621]
2,25	0,00038	NLRP3	NM_004895	Homo sapiens NLR family, pyrin domain containing 3 (NLRP3), transcript variant 1, mRNA [NM_004895]
2,25	0,00018	IFNE1	NM_176891	Homo sapiens interferon epsilon 1 (IFNE1), mRNA [NM_176891]
2,25	0,00001	JMJD1C	NM_032776	Homo sapiens jumonji domain containing 1C (JMJD1C), transcript variant 1, mRNA [NM_032776]

2,25	0,00004	HK2	NM_000189	Homo sapiens hexokinase 2 (HK2), mRNA [NM_000189]
2,25	0,00001	GNPDA2	NM_138335	Homo sapiens glucosamine-6-phosphate deaminase 2 (GNPDA2), mRNA [NM_138335]
2,24	0,00127	OPCML	NM_001012393	Homo sapiens opioid binding protein/cell adhesion molecule-like (OPCML), transcript variant 2, mRNA [NM_001012393]
2,24	0,00089	ANKRD37	NM_181726	Homo sapiens ankyrin repeat domain 37 (ANKRD37), mRNA [NM_181726]
2,24	0,00006	PTPRR	NM_002849	Homo sapiens protein tyrosine phosphatase, receptor type, R (PTPRR), transcript variant 1, mRNA [NM_002849]
2,24	0,00001	GSTO1	NM_004832	Homo sapiens glutathione S-transferase omega 1 (GSTO1), mRNA [NM_004832]
2,24	0,00000	LAMP2	NM_013995	Homo sapiens lysosomal-associated membrane protein 2 (LAMP2), transcript variant LAMP2B, mRNA [NM_013995]
2,24	0,00011	ADAMTSL1	NM_052866	Homo sapiens ADAMTS-like 1 (ADAMTSL1), transcript variant 2, mRNA [NM_052866]
2,23	0,00000	AP1S2	NM_003916	Homo sapiens adaptor-related protein complex 1, sigma 2 subunit (AP1S2), mRNA [NM_003916]
2,23	0,00006	AXL	NM_021913	Homo sapiens AXL receptor tyrosine kinase (AXL), transcript variant 1, mRNA [NM_021913]
2,23	0,00094	TGFB2	ENST00000366930	Transforming growth factor beta-2 precursor (TGF-beta-2) (Glioblastoma-derived T-cell suppressor factor) (G-TSF) (BSC-1 cell growth inhibitor) (Polyergin) (Cetermin). [Source:Uniprot/SWISSPROT;Acc:P61812] [ENST00000366930]
2,23	0,00003	SPOCD1	NM_144569	Homo sapiens SPOC domain containing 1 (SPOCD1), mRNA [NM_144569]
2,23	0,00119	NEXN	NM_144573	Homo sapiens nexilin (F actin binding protein) (NEXN), mRNA [NM_144573]
2,23	0,00013	ZNF175	BC007778	Homo sapiens zinc finger protein 175, mRNA (cDNA clone IMAGE:4301632), partial cds. [BC007778]
2,23	0,00054	NRIP3	NM_020645	Homo sapiens nuclear receptor interacting protein 3 (NRIP3), mRNA [NM_020645]
2,22	0,00004	TBXAS1	BC014117	Homo sapiens thromboxane A synthase 1 (platelet, cytochrome P450, family 5, subfamily A), mRNA (cDNA clone MGC:20885 IMAGE:4548935), complete cds. [BC014117]
2,22	0,00296	LINGO2	NM_152570	Homo sapiens leucine rich repeat and Ig domain containing 2 (LINGO2), mRNA [NM_152570]
2,21	0,00027	COL27A1	AK021957	Homo sapiens cDNA FLJ11895 fis, clone HEMBA1007301, weakly similar to COLLAGEN ALPHA 1(III) CHAIN. [AK021957]

2,21	0,00002	CR622110	CR622110	full-length cDNA clone CS0DC025YP03 of Neuroblastoma Cot 25-normalized of Homo sapiens (human) [CR622110]
2,21	0,00011	TUBA1B	NM_006082	Homo sapiens tubulin, alpha 1b (TUBA1B), mRNA [NM_006082]
2,21	0,00009	DNAJC9	NM_015190	Homo sapiens DnaJ (Hsp40) homolog, subfamily C, member 9 (DNAJC9), mRNA [NM_015190]
2,21	0,00002	PTPN12	NM_002835	Homo sapiens protein tyrosine phosphatase, non-receptor type 12 (PTPN12), mRNA [NM_002835]
2,20	0,00050	MT2A	ENST00000245185	Metallothionein-2 (MT-2) (Metallothionein-II) (MT-II) (Metallothionein-2A). [Source:Uniprot/SWISSPROT;Acc:P02795] [ENST00000245185]
2,20	0,00001	AB062480	AB062480	Homo sapiens OK/SW-cl.79 mRNA, complete cds. [AB062480]
2,20	0,00489	TIPARP	NM_015508	Homo sapiens TCDD-inducible poly(ADP-ribose) polymerase (TIPARP), mRNA [NM_015508]
2,18	0,00001	SYT15	NM_181519	Homo sapiens synaptotagmin XV (SYT15), transcript variant b, mRNA [NM_181519]
2,18	0,00455	CENPE	NM_001813	Homo sapiens centromere protein E, 312kDa (CENPE), mRNA [NM_001813]
2,18	0,00001	SSR3	NM_007107	Homo sapiens signal sequence receptor, gamma (translocon-associated protein gamma) (SSR3), mRNA [NM_007107]
2,18	0,00047	NFAT5	NM_173215	Homo sapiens nuclear factor of activated T-cells 5, tonicity-responsive (NFAT5), transcript variant 5, mRNA [NM_173215]
2,18	0,00003	CSF2	NM_000758	Homo sapiens colony stimulating factor 2 (granulocyte-macrophage) (CSF2), mRNA [NM_000758]
2,17	0,00061	OSBPL8	NM_020841	Homo sapiens oxysterol binding protein-like 8 (OSBPL8), transcript variant 1, mRNA [NM_020841]
2,17	0,00907	P4HA3	NM_182904	Homo sapiens procollagen-proline, 2-oxoglutarate 4-dioxygenase (proline 4-hydroxylase), alpha polypeptide III (P4HA3), mRNA [NM_182904]
2,17	0,00015	TOX2	NM_032883	Homo sapiens TOX high mobility group box family member 2 (TOX2), mRNA [NM_032883]
2,17	0,00004	AF126109	AF126109	Homo sapiens clone TA40 untranslated mRNA, complete sequence. [AF126109]
2,17	0,00001	GLI1	NM_005269	Homo sapiens glioma-associated oncogene homolog 1 (zinc finger protein) (GLI1), mRNA [NM_005269]
2,16	0,00011	TMEM38B	NM_018112	Homo sapiens transmembrane protein 38B (TMEM38B), mRNA [NM_018112]
2,16	0,00001	PTPRK	NM_002844	Homo sapiens protein tyrosine phosphatase, receptor type, K (PTPRK), mRNA [NM_002844]
2,15	0,00023	TNIP3	NM_024873	Homo sapiens TNFAIP3 interacting protein 3 (TNIP3), mRNA [NM_024873]

2,15	0,00453	RORA	NM_134260	Homo sapiens RAR-related orphan receptor A (RORA), transcript variant 2, mRNA [NM_134260]
2,15	0,00005	UPP1	NM_181597	Homo sapiens uridine phosphorylase 1 (UPP1), transcript variant 2, mRNA [NM_181597]
2,14	0,00006	METTL8	NM_024770	Homo sapiens methyltransferase like 8 (METTL8), mRNA [NM_024770]
2,14	0,00002	TGM2	NM_198951	Homo sapiens transglutaminase 2 (C polypeptide, protein-glutamine-gamma-glutamyltransferase) (TGM2), transcript variant 2, mRNA [NM_198951]
2,14	0,00075	HSP90AB3P	AY956764	Homo sapiens heat shock protein 90Bc (HSP90Bc) mRNA, complete cds. [AY956764]
2,14	0,00682	CENPQ	NM_018132	Homo sapiens centromere protein Q (CENPQ), mRNA [NM_018132]
2,14	0,00454	CALD1	AF247820	Homo sapiens NAG22 protein mRNA, complete cds. [AF247820]
2,14	0,00004	TMEM65	NM_194291	Homo sapiens transmembrane protein 65 (TMEM65), mRNA [NM_194291]
2,13	0,00001	ZEB2	NM_014795	Homo sapiens zinc finger E-box binding homeobox 2 (ZEB2), mRNA [NM_014795]
2,13	0,00002	ANTXR2	NM_058172	Homo sapiens anthrax toxin receptor 2 (ANTXR2), mRNA [NM_058172]
2,13	0,00005	SLC37A2	NM_198277	Homo sapiens solute carrier family 37 (glycerol-3-phosphate transporter), member 2 (SLC37A2), mRNA [NM_198277]
2,12	0,00005	IL4I1	NM_172374	Homo sapiens interleukin 4 induced 1 (IL4I1), transcript variant 2, mRNA [NM_172374]
2,12	0,00023	ITGA1	NM_181501	Homo sapiens integrin, alpha 1 (ITGA1), mRNA [NM_181501]
2,12	0,00139	AK5	NM_174858	Homo sapiens adenylate kinase 5 (AK5), transcript variant 1, mRNA [NM_174858]
2,12	0,00364	TREM1	NM_018643	Homo sapiens triggering receptor expressed on myeloid cells 1 (TREM1), mRNA [NM_018643]
2,12	0,00000	RAP2A	NM_021033	Homo sapiens RAP2A, member of RAS oncogene family (RAP2A), mRNA [NM_021033]
2,11	0,00001	FAM129B	AF151783	Homo sapiens MEG3 (MEG3) mRNA, complete cds. [AF151783]
2,10	0,00005	SLC25A32	NM_030780	Homo sapiens solute carrier family 25, member 32 (SLC25A32), mRNA [NM_030780]
2,10	0,00331	SMC4	NM_005496	Homo sapiens structural maintenance of chromosomes 4 (SMC4), transcript variant 1, mRNA [NM_005496]
2,09	0,00001	TUBB6	NM_032525	Homo sapiens tubulin, beta 6 (TUBB6), mRNA [NM_032525]
2,09	0,00701	FAM40B	NM_020704	Homo sapiens family with sequence similarity 40, member B (FAM40B), mRNA [NM_020704]

2,09	0,00301	PKIA	NM_006823	Homo sapiens protein kinase (cAMP-dependent, catalytic) inhibitor alpha (PKIA), transcript variant 6, mRNA [NM_006823]
2,09	0,00012	LOC283345	AY672103	Homo sapiens RRPL13L mRNA, partial sequence. [AY672103]
2,09	0,00064	VAC14	U25801	Human Tax1 binding protein mRNA, partial cds. [U25801]
2,09	0,00056	KCTD12	NM_138444	Homo sapiens potassium channel tetramerisation domain containing 12 (KCTD12), mRNA [NM_138444]
2,09	0,00026	MTMR11	NM_181873	Homo sapiens myotubularin related protein 11 (MTMR11), mRNA [NM_181873]
2,09	0,00043	CD33L3	NM_213602	Homo sapiens CD33 molecule-like 3 (CD33L3), mRNA [NM_213602]
2,09	0,00243	CKAP4	NM_006825	Homo sapiens cytoskeleton-associated protein 4 (CKAP4), mRNA [NM_006825]
2,09	0,00607	RKHD3	NM_032246	Homo sapiens ring finger and KH domain containing 3 (RKHD3), mRNA [NM_032246]
2,08	0,00041	CCDC99	NM_017785	Homo sapiens coiled-coil domain containing 99 (CCDC99), mRNA [NM_017785]
2,08	0,00006	CD68	NM_001251	Homo sapiens CD68 molecule (CD68), transcript variant 1, mRNA [NM_001251]
2,08	0,00014	FRMD6	NM_152330	Homo sapiens FERM domain containing 6 (FRMD6), transcript variant 2, mRNA [NM_152330]
2,08	0,00874	KRT32	NM_002278	Homo sapiens keratin 32 (KRT32), mRNA [NM_002278]
2,08	0,00024	U52054	U52054	Human S6 H-8 mRNA expressed in chromosome 6-suppressed melanoma cells. [U52054]
2,08	0,00181	GRASP	NM_181711	Homo sapiens GRP1 (general receptor for phosphoinositides 1)-associated scaffold protein (GRASP), mRNA [NM_181711]
2,08	0,00003	CD109	NM_133493	Homo sapiens CD109 molecule (CD109), mRNA [NM_133493]
2,07	0,00007	AF275804	AF275804	Homo sapiens PNAS-108 mRNA, partial sequence. [AF275804]
2,07	0,00006	KDELR2	NM_006854	Homo sapiens KDEL (Lys-Asp-Glu-Leu) endoplasmic reticulum protein retention receptor 2 (KDELR2), mRNA [NM_006854]
2,07	0,00052	ZNF143	U09850	Human zinc finger protein (ZNF143) mRNA, complete cds. [U09850]
2,07	0,00004	ARSJ	NM_024590	Homo sapiens arylsulfatase family, member J (ARSJ), mRNA [NM_024590]
2,06	0,00484	HOXA11S	NR_002795	Homo sapiens homeo box A11, antisense (HOXA11S) on chromosome 7 [NR_002795]
2,06	0,00011	RAGE	NM_014226	Homo sapiens renal tumor antigen (RAGE), mRNA [NM_014226]
2,06	0,00000	AF109189	AF109189	Homo sapiens FWP007 mRNA, complete cds. [AF109189]
2,06	0,00342	CUGBP1	NM_198700	Homo sapiens CUG triplet repeat, RNA binding protein 1 (CUGBP1), transcript variant 2, mRNA [NM_198700]
2,06	0,00001	HERC4	NM_001017972	Homo sapiens hect domain and RLD 4 (HERC4), transcript variant 3, mRNA [NM_001017972]

2,05	0,00001	GPR180	NM_180989	Homo sapiens G protein-coupled receptor 180 (GPR180), mRNA [NM_180989]
2,05	0,00020	ENST00000356931	ENST00000356931	MGC10850 protein. [Source:Uniprot/SPTREMBL;Acc:Q96C21] [ENST00000356931]
2,05	0,00001	ENST00000377666	ENST00000377666	Krueppel-like factor 12 (Transcriptional repressor AP-2rep). [Source:Uniprot/SWISSPROT;Acc:Q9Y4X4] [ENST00000377666]
2,05	0,00019	NTF3	NM_002527	Homo sapiens neurotrophin 3 (NTF3), mRNA [NM_002527]
2,05	0,00001	HISPPD1	NM_015216	Homo sapiens histidine acid phosphatase domain containing 1 (HISPPD1), mRNA [NM_015216]
2,05	0,00006	CABLES1	NM_138375	Homo sapiens Cdk5 and Abl enzyme substrate 1 (CABLES1), mRNA [NM_138375]
2,04	0,00006	HOXC8	NM_022658	Homo sapiens homeobox C8 (HOXC8), mRNA [NM_022658]
2,04	0,00006	ENST00000229270	ENST00000229270	Triosephosphate isomerase (EC 5.3.1.1) (TIM) (Triose-phosphate isomerase). [Source:Uniprot/SWISSPROT;Acc:P60174] [ENST00000229270]
2,04	0,00649	SERPINB2	ENST00000299502	Plasminogen activator inhibitor 2 precursor (PAI-2) (Placental plasminogen activator inhibitor) (Monocyte Arg-serpin) (Urokinase inhibitor). [Source:Uniprot/SWISSPROT;Acc:P05120] [ENST00000299502]
2,04	0,00861	NETO2	NM_018092	Homo sapiens neuropilin (NRP) and tolloid (TLL)-like 2 (NETO2), mRNA [NM_018092]
2,04	0,00004	RP11-11C5.2	NM_001071775	Homo sapiens similar to RIKEN cDNA 2410129H14 (LOC440145), mRNA [NM_001071775]
2,03	0,00011	MPHOSPH6	NM_005792	Homo sapiens M-phase phosphoprotein 6 (MPHOSPH6), mRNA [NM_005792]
2,03	0,00002	DNAL1	NM_031427	Homo sapiens dynein, axonemal, light chain 1 (DNAL1), mRNA [NM_031427]
2,03	0,00028	LHFP	NM_005780	Homo sapiens lipoma HMGIC fusion partner (LHFP), mRNA [NM_005780]
2,02	0,00001	BRI3	NM_015379	Homo sapiens brain protein I3 (BRI3), mRNA [NM_015379]
2,02	0,00017	HPRT1	NM_000194	Homo sapiens hypoxanthine phosphoribosyltransferase 1 (Lesch-Nyhan syndrome) (HPRT1), mRNA [NM_000194]
2,02	0,00049	FKBP14	NM_017946	Homo sapiens FK506 binding protein 14, 22 kDa (FKBP14), mRNA [NM_017946]
2,01	0,00015	HMG1L1	NM_001008735	Homo sapiens high-mobility group (nonhistone chromosomal) protein 1-like 1 (HMG1L1), mRNA [NM_001008735]
2,01	0,00012	GALNT5	NM_014568	Homo sapiens UDP-N-acetyl-alpha-D-galactosamine:polypeptide N-acetylgalactosaminyltransferase 5 (GalNAc-T5) (GALNT5), mRNA [NM_014568]

2,01	0,00037	PTPRG	NM_002841	Homo sapiens protein tyrosine phosphatase, receptor type, G (PTPRG), mRNA [NM_002841]
2,01	0,00373	PPFIA4	NM_015053	Homo sapiens protein tyrosine phosphatase, receptor type, f polypeptide (PTPRF), interacting protein (liprin), alpha 4 (PPFIA4), mRNA [NM_015053]
2,01	0,00005	KIAA0754	AB018297	Homo sapiens mRNA for KIAA0754 protein, partial cds. [AB018297]
2,01	0,00004	PLEC1	NM_201380	Homo sapiens plectin 1, intermediate filament binding protein 500kDa (PLEC1), transcript variant 6, mRNA [NM_201380]
2,01	0,00001	MTR	NM_000254	Homo sapiens 5-methyltetrahydrofolate-homocysteine methyltransferase (MTR), mRNA [NM_000254]
2,01	0,00006	CEP290	NM_025114	Homo sapiens centrosomal protein 290kDa (CEP290), mRNA [NM_025114]
2,00	0,00003	TXNDC10	NM_019022	Homo sapiens thioredoxin domain containing 10 (TXNDC10), mRNA [NM_019022]
2,00	0,00007	NEK7	ENST00000367385	Serine/threonine-protein kinase Nek7 (EC 2.7.11.1) (NimA-related protein kinase 7). [Source:Uniprot/SWISSPROT;Acc:Q8TDX7] [ENST00000367385]

Supplementary Table S6. Microarray repressed genes in Non-Epithelioid phenotype.

Ratio Non-Epithelioid	Non-Epithelioid FDR	Gene Symbol	Systematic Name	Description
0,03	0,00006	PRG4	NM_005807	Homo sapiens proteoglycan 4 (PRG4), mRNA [NM_005807]
0,03	0,00000	KLK7	NM_005046	Homo sapiens kallikrein-related peptidase 7 (KLK7), transcript variant 1, mRNA [NM_005046]
0,05	0,00000	TGM1	NM_000359	Homo sapiens transglutaminase 1 (K polypeptide epidermal type I, protein-glutamine-gamma-glutamyltransferase) (TGM1), mRNA [NM_000359]
0,06	0,00001	KLK8	NM_144505	Homo sapiens kallikrein-related peptidase 8 (KLK8), transcript variant 2, mRNA [NM_144505]
0,06	0,00000	THBD	NM_000361	Homo sapiens thrombomodulin (THBD), mRNA [NM_000361]
0,06	0,00000	CLIC5	NM_016929	Homo sapiens chloride intracellular channel 5 (CLIC5), mRNA [NM_016929]
0,06	0,00000	HHIP	NM_022475	Homo sapiens hedgehog interacting protein (HHIP), mRNA [NM_022475]
0,07	0,00000	EPS8L1	NM_133180	Homo sapiens EPS8-like 1 (EPS8L1), transcript variant 1, mRNA [NM_133180]
0,07	0,00002	RPESP	NM_153225	Homo sapiens RPE-spondin (RPESP), mRNA [NM_153225]
0,07	0,00000	MYL7	NM_021223	Homo sapiens myosin, light chain 7, regulatory (MYL7), mRNA [NM_021223]
0,08	0,00000	HP	NM_005143	Homo sapiens haptoglobin (HP), mRNA [NM_005143]
0,08	0,00000	SFTPD	NM_003019	Homo sapiens surfactant, pulmonary-associated protein D (SFTPD), mRNA [NM_003019]
0,09	0,00098	SEPP1	NM_005410	Homo sapiens selenoprotein P, plasma, 1 (SEPP1), mRNA [NM_005410]
0,09	0,00000	SLPI	NM_003064	Homo sapiens secretory leukocyte peptidase inhibitor (SLPI), mRNA [NM_003064]
0,09	0,00000	KLK5	NM_012427	Homo sapiens kallikrein-related peptidase 5 (KLK5), transcript variant 1, mRNA [NM_012427]
0,09	0,00003	CDH1	NM_004360	Homo sapiens cadherin 1, type 1, E-cadherin (epithelial) (CDH1), mRNA [NM_004360]
0,09	0,00000	CST6	NM_001323	Homo sapiens cystatin E/M (CST6), mRNA [NM_001323]
0,09	0,00000	IGFL2	NM_001002915	Homo sapiens IGF-like family member 2 (IGFL2), mRNA [NM_001002915]

0,09	0,00000	KCNB1	NM_004975	Homo sapiens potassium voltage-gated channel, Shab-related subfamily, member 1 (KCNB1), mRNA [NM_004975]
0,09	0,00003	ITLN1	NM_017625	Homo sapiens intelectin 1 (galactofuranose binding) (ITLN1), mRNA [NM_017625]
0,10	0,00000	NOX1	NM_013955	Homo sapiens NADPH oxidase 1 (NOX1), transcript variant NOH-1Lv, mRNA [NM_013955]
0,10	0,00004	RARRES1	NM_002888	Homo sapiens retinoic acid receptor responder (tazarotene induced) 1 (RARRES1), transcript variant 2, mRNA [NM_002888]
0,10	0,00000	SMTNL2	NM_198501	Homo sapiens smoothelin-like 2 (SMTNL2), mRNA [NM_198501]
0,11	0,00003	KLK11	NM_144947	Homo sapiens kallikrein-related peptidase 11 (KLK11), transcript variant 2, mRNA [NM_144947]
0,11	0,00000	MYLPF	NM_013292	Homo sapiens fast skeletal myosin light chain 2 (MYLPF), mRNA [NM_013292]
0,12	0,00000	AIF1	NM_004847	Homo sapiens allograft inflammatory factor 1 (AIF1), transcript variant 2, mRNA [NM_004847]
0,12	0,00001	CFB	NM_001710	Homo sapiens complement factor B (CFB), mRNA [NM_001710]
0,12	0,00000	SPOCK2	NM_014767	Homo sapiens sparc/osteonectin, cwcv and kazal-like domains proteoglycan (testican) 2 (SPOCK2), mRNA [NM_014767]
0,12	0,00006	SPANXB2	NM_145664	Homo sapiens SPANX family, member B2 (SPANXB2), mRNA [NM_145664]
0,12	0,00000	ABLIM2	NM_032432	Homo sapiens actin binding LIM protein family, member 2 (ABLIM2), mRNA [NM_032432]
0,13	0,00000	NPHS1	NM_004646	Homo sapiens nephrosis 1, congenital, Finnish type (nephrin) (NPHS1), mRNA [NM_004646]
0,13	0,00005	OGDHL	NM_018245	Homo sapiens oxoglutarate dehydrogenase-like (OGDHL), mRNA [NM_018245]
0,13	0,00000	APOA1	NM_000039	Homo sapiens apolipoprotein A-I (APOA1), mRNA [NM_000039]
0,13	0,00000	MAOB	NM_000898	Homo sapiens monoamine oxidase B (MAOB), nuclear gene encoding mitochondrial protein, mRNA [NM_000898]
0,14	0,00000	TMOD1	NM_003275	Homo sapiens tropomodulin 1 (TMOD1), mRNA [NM_003275]
0,14	0,00001	PODXL	NM_005397	Homo sapiens podocalyxin-like (PODXL), transcript variant 2, mRNA [NM_005397]
0,14	0,00005	SPANXD	NM_032417	Homo sapiens SPANX family, member D (SPANXD), mRNA [NM_032417]
0,14	0,00000	NOX1	NM_007052	Homo sapiens NADPH oxidase 1 (NOX1), transcript variant NOH-1L, mRNA [NM_007052]
0,14	0,00006	CXCL14	NM_004887	Homo sapiens chemokine (C-X-C motif) ligand 14 (CXCL14), mRNA [NM_004887]

0,14	0,00000	CHI3L2	NM_004000	Homo sapiens chitinase 3-like 2 (CHI3L2), transcript variant 1, mRNA [NM_004000]
0,14	0,00000	CYP17A1	NM_000102	Homo sapiens cytochrome P450, family 17, subfamily A, polypeptide 1 (CYP17A1), mRNA [NM_000102]
0,14	0,00002	ITM2A	NM_004867	Homo sapiens integral membrane protein 2A (ITM2A), mRNA [NM_004867]
0,14	0,00013	TSPAN8	NM_004616	Homo sapiens tetraspanin 8 (TSPAN8), mRNA [NM_004616]
0,14	0,00012	MYH3	NM_002470	Homo sapiens myosin, heavy chain 3, skeletal muscle, embryonic (MYH3), mRNA [NM_002470]
0,14	0,00000	CGN	NM_020770	Homo sapiens cingulin (CGN), mRNA [NM_020770]
0,15	0,00000	KLK10	NM_002776	Homo sapiens kallikrein-related peptidase 10 (KLK10), transcript variant 1, mRNA [NM_002776]
0,15	0,00000	SPINT2	NM_021102	Homo sapiens serine peptidase inhibitor, Kunitz type, 2 (SPINT2), mRNA [NM_021102]
0,15	0,00003	SLC40A1	NM_014585	Homo sapiens solute carrier family 40 (iron-regulated transporter), member 1 (SLC40A1), mRNA [NM_014585]
0,15	0,00000	UPK3B	NM_030570	Homo sapiens uroplakin 3B (UPK3B), transcript variant 1, mRNA [NM_030570]
0,15	0,00000	KCNQ1	NM_000218	Homo sapiens potassium voltage-gated channel, KQT-like subfamily, member 1 (KCNQ1), transcript variant 1, mRNA [NM_000218]
0,15	0,00000	CPA4	NM_016352	Homo sapiens carboxypeptidase A4 (CPA4), mRNA [NM_016352]
0,15	0,00000	HPR	NM_020995	Homo sapiens haptoglobin-related protein (HPR), mRNA [NM_020995]
0,15	0,00003	ANGPTL7	NM_021146	Homo sapiens angiopoietin-like 7 (ANGPTL7), mRNA [NM_021146]
0,16	0,00001	SLC7A7	NM_003982	Homo sapiens solute carrier family 7 (cationic amino acid transporter, y+ system), member 7 (SLC7A7), mRNA [NM_003982]
0,16	0,00000	NOX1	NM_013954	Homo sapiens NADPH oxidase 1 (NOX1), transcript variant NOH-1S, mRNA [NM_013954]
0,16	0,00001	MAL2	NM_052886	Homo sapiens mal, T-cell differentiation protein 2 (MAL2), mRNA [NM_052886]
0,16	0,00000	VTN	NM_000638	Homo sapiens vitronectin (VTN), mRNA [NM_000638]
0,16	0,00017	ZBED2	NM_024508	Homo sapiens zinc finger, BED-type containing 2 (ZBED2), mRNA [NM_024508]
0,16	0,00000	ENST00000376682	ENST00000376682	Probable G-protein coupled receptor 133 precursor (G-protein coupled receptor PGR25). [Source:Uniprot/SWISSPROT;Acc:Q6QNK2] [ENST00000376682]
0,16	0,00001	UPK1B	NM_006952	Homo sapiens uroplakin 1B (UPK1B), mRNA [NM_006952]
0,17	0,00058	COL4A6	NM_033641	Homo sapiens collagen, type IV, alpha 6 (COL4A6), transcript variant B, mRNA [NM_033641]

0,17	0,00001	ENPP1	NM_006208	Homo sapiens ectonucleotide pyrophosphatase/phosphodiesterase 1 (ENPP1), mRNA [NM_006208]
0,17	0,00011	WNT2B	NM_004185	Homo sapiens wingless-type MMTV integration site family, member 2B (WNT2B), transcript variant WNT-2B1, mRNA [NM_004185]
0,17	0,00001	PDZK1IP1	NM_005764	Homo sapiens PDZK1 interacting protein 1 (PDZK1IP1), mRNA [NM_005764]
0,18	0,00002	FGF9	NM_002010	Homo sapiens fibroblast growth factor 9 (glia-activating factor) (FGF9), mRNA [NM_002010]
0,18	0,00006	CAPN6	NM_014289	Homo sapiens calpain 6 (CAPN6), mRNA [NM_014289]
0,18	0,00000	MUC16	NM_024690	Homo sapiens mucin 16, cell surface associated (MUC16), mRNA [NM_024690]
0,19	0,00000	SASH1	NM_015278	Homo sapiens SAM and SH3 domain containing 1 (SASH1), mRNA [NM_015278]
0,19	0,00059	MT1F	NM_005949	Homo sapiens metallothionein 1F (MT1F), mRNA [NM_005949]
0,19	0,00003	GAS1	NM_002048	Homo sapiens growth arrest-specific 1 (GAS1), mRNA [NM_002048]
0,20	0,00255	RGS5	NM_003617	Homo sapiens regulator of G-protein signalling 5 (RGS5), mRNA [NM_003617]
0,20	0,00032	NDRG4	NM_022910	Homo sapiens NDRG family member 4 (NDRG4), mRNA [NM_022910]
0,20	0,00001	TXNIP	NM_006472	Homo sapiens thioredoxin interacting protein (TXNIP), mRNA [NM_006472]
0,20	0,00000	RAB11FIP1	NM_001002233	Homo sapiens RAB11 family interacting protein 1 (class I) (RAB11FIP1), transcript variant 2, mRNA [NM_001002233]
0,20	0,00000	CLDN15	NM_014343	Homo sapiens claudin 15 (CLDN15), transcript variant 1, mRNA [NM_014343]
0,20	0,00015	HBD	NM_000519	Homo sapiens hemoglobin, delta (HBD), mRNA [NM_000519]
0,20	0,00000	CHRNA3	NM_000743	Homo sapiens cholinergic receptor, nicotinic, alpha 3 (CHRNA3), mRNA [NM_000743]
0,20	0,00000	TSPAN7	NM_004615	Homo sapiens tetraspanin 7 (TSPAN7), mRNA [NM_004615]
0,20	0,00001	CRB2	NM_173689	Homo sapiens crumbs homolog 2 (Drosophila) (CRB2), mRNA [NM_173689]
0,20	0,00001	NEUROG3	NM_020999	Homo sapiens neurogenin 3 (NEUROG3), mRNA [NM_020999]
0,20	0,00006	TM4SF4	NM_004617	Homo sapiens transmembrane 4 L six family member 4 (TM4SF4), mRNA [NM_004617]
0,21	0,00063	ID4	NM_001546	Homo sapiens inhibitor of DNA binding 4, dominant negative helix-loop-helix protein (ID4), mRNA [NM_001546]
0,21	0,00001	TMPRSS3	NM_032405	Homo sapiens transmembrane protease, serine 3 (TMPRSS3), transcript variant D, mRNA [NM_032405]
0,21	0,00000	ADRA2A	NM_000681	Homo sapiens adrenergic, alpha-2A-, receptor (ADRA2A), mRNA [NM_000681]

0,21	0,00001	WFDC2	NM_080734	Homo sapiens WAP four-disulfide core domain 2 (WFDC2), transcript variant 4, mRNA [NM_080734]
0,21	0,00001	ENPP1	ENST00000367994	Ectonucleotide pyrophosphatase/phosphodiesterase 1 (E-NPP 1) (Phosphodiesterase I/nucleotide pyrophosphatase 1) (Plasma-cell membrane glycoprotein PC-1) [Includes: Alkaline phosphodiesterase I (EC 3.1.4.1); Nucleotide pyrophosphatase (EC 3.6.1.9)...
0,21	0,00000	FGF18	NM_003862	Homo sapiens fibroblast growth factor 18 (FGF18), mRNA [NM_003862]
0,21	0,00001	EEF1A2	NM_001958	Homo sapiens eukaryotic translation elongation factor 1 alpha 2 (EEF1A2), mRNA [NM_001958]
0,21	0,00000	SAMD5	NM_001030060	Homo sapiens SAM domain containing 1 (LOC389432), mRNA [NM_001030060]
0,21	0,00000	SMPDL3B	NM_001009568	Homo sapiens sphingomyelin phosphodiesterase, acid-like 3B (SMPDL3B), transcript variant 2, mRNA [NM_001009568]
0,21	0,00014	DES	NM_001927	Homo sapiens desmin (DES), mRNA [NM_001927]
0,22	0,00000	DACT2	NM_214462	Homo sapiens dapper, antagonist of beta-catenin, homolog 2 (Xenopus laevis) (DACT2), mRNA [NM_214462]
0,22	0,00014	ST6GALNAC3	NM_152996	Homo sapiens ST6 (alpha-N-acetyl-neuraminyl-2,3-beta-galactosyl-1,3)-N-acetylgalactosaminide alpha-2,6-sialyltransferase 3 (ST6GALNAC3), mRNA [NM_152996]
0,22	0,00003	WNT4	NM_030761	Homo sapiens wingless-type MMTV integration site family, member 4 (WNT4), mRNA [NM_030761]
0,22	0,00000	SYN1	NM_133499	Homo sapiens synapsin I (SYN1), transcript variant Ib, mRNA [NM_133499]
0,22	0,00001	MYO5C	NM_018728	Homo sapiens myosin VC (MYO5C), mRNA [NM_018728]
0,22	0,00000	CRISPLD1	NM_031461	Homo sapiens cysteine-rich secretory protein LCCL domain containing 1 (CRISPLD1), mRNA [NM_031461]
0,22	0,00000	PPP1R12B	NM_032105	Homo sapiens protein phosphatase 1, regulatory (inhibitor) subunit 12B (PPP1R12B), transcript variant 2, mRNA [NM_032105]
0,22	0,00026	TMEM176B	NM_014020	Homo sapiens transmembrane protein 176B (TMEM176B), mRNA [NM_014020]
0,23	0,00000	CLEC4M	NM_214677	Homo sapiens C-type lectin domain family 4, member M (CLEC4M), transcript variant 4, mRNA [NM_214677]
0,23	0,00000	NTRK2	NM_001007097	Homo sapiens neurotrophic tyrosine kinase, receptor, type 2 (NTRK2), transcript variant b, mRNA [NM_001007097]
0,23	0,00000	TMEM139	NM_153345	Homo sapiens transmembrane protein 139 (TMEM139), mRNA [NM_153345]
0,23	0,00003	FBXO2	NM_012168	Homo sapiens F-box protein 2 (FBXO2), mRNA [NM_012168]

0,23	0,00013	OLR1	NM_002543	Homo sapiens oxidized low density lipoprotein (lectin-like) receptor 1 (OLR1), mRNA [NM_002543]
0,23	0,00045	NMU	NM_006681	Homo sapiens neuromedin U (NMU), mRNA [NM_006681]
0,23	0,00034	TM4SF1	NM_014220	Homo sapiens transmembrane 4 L six family member 1 (TM4SF1), mRNA [NM_014220]
0,23	0,00000	SMPD3	NM_018667	Homo sapiens sphingomyelin phosphodiesterase 3, neutral membrane (neutral sphingomyelinase II) (SMPD3), mRNA [NM_018667]
0,23	0,00000	FAAH2	NM_174912	Homo sapiens fatty acid amide hydrolase 2 (FAAH2), mRNA [NM_174912]
0,24	0,00005	AQP1	NM_198098	Homo sapiens aquaporin 1 (Colton blood group) (AQP1), mRNA [NM_198098]
0,24	0,00001	RSPO1	NM_001038633	Homo sapiens R-spondin homolog (Xenopus laevis) (RSPO1), mRNA [NM_001038633]
0,24	0,00013	TPD52L1	NM_001003395	Homo sapiens tumor protein D52-like 1 (TPD52L1), transcript variant 2, mRNA [NM_001003395]
0,24	0,00324	SMOC2	NM_022138	Homo sapiens SPARC related modular calcium binding 2 (SMOC2), mRNA [NM_022138]
0,24	0,00000	DOK7	NM_173660	Homo sapiens docking protein 7 (DOK7), mRNA [NM_173660]
0,24	0,00005	SLAIN1	NM_144595	Homo sapiens SLAIN motif family, member 1 (SLAIN1), transcript variant 2, mRNA [NM_144595]
0,24	0,00000	LRRN2	NM_201630	Homo sapiens leucine rich repeat neuronal 2 (LRRN2), transcript variant 2, mRNA [NM_201630]
0,24	0,00002	C3	NM_000064	Homo sapiens complement component 3 (C3), mRNA [NM_000064]
0,24	0,00000	SCN4B	NM_174934	Homo sapiens sodium channel, voltage-gated, type IV, beta (SCN4B), mRNA [NM_174934]
0,24	0,00000	KCNA4	NM_002233	Homo sapiens potassium voltage-gated channel, shaker-related subfamily, member 4 (KCNA4), mRNA [NM_002233]
0,24	0,00000	FBXL17	BC063316	Homo sapiens F-box and leucine-rich repeat protein 17, mRNA (cDNA clone MGC:71854 IMAGE:30341140), complete cds. [BC063316]
0,24	0,00000	BMP4	NM_001202	Homo sapiens bone morphogenetic protein 4 (BMP4), transcript variant 1, mRNA [NM_001202]
0,24	0,00002	DKFZp761P0423	ENST00000330777	Tyrosine-protein kinase SgK223 (EC 2.7.10.2) (Sugen kinase 223). [Source:Uniprot/SWISSPROT;Acc:Q86YV5] [ENST00000330777]
0,24	0,00002	SARM1	NM_015077	Homo sapiens sterile alpha and TIR motif containing 1 (SARM1), mRNA [NM_015077]
0,25	0,00594	BTNL8	NM_024850	Homo sapiens butyrophilin-like 8 (BTNL8), transcript variant 1, mRNA [NM_024850]
0,25	0,00001	COL8A1	NM_001850	Homo sapiens collagen, type VIII, alpha 1 (COL8A1), transcript variant 1, mRNA [NM_001850]

0,25	0,00014	ITIH5	ENST00000256861	inter-alpha trypsin inhibitor heavy chain precursor 5 isoform 1 [Source:RefSeq_peptide;Acc:NP_085046] [ENST00000256861]
0,25	0,00000	ITIH5	NM_030569	Homo sapiens inter-alpha (globulin) inhibitor H5 (ITIH5), transcript variant 1, mRNA [NM_030569]
0,25	0,00000	SH3GL3	NM_003027	Homo sapiens SH3-domain GRB2-like 3 (SH3GL3), mRNA [NM_003027]
0,25	0,00004	KCNH2	NM_000238	Homo sapiens potassium voltage-gated channel, subfamily H (eag-related), member 2 (KCNH2), transcript variant 1, mRNA [NM_000238]
0,25	0,00001	CDH24	NM_022478	Homo sapiens cadherin-like 24 (CDH24), transcript variant 1, mRNA [NM_022478]
0,25	0,00005	FAM5B	NM_021165	Homo sapiens family with sequence similarity 5, member B (FAM5B), mRNA [NM_021165]
0,26	0,00001	VIPR2	NM_003382	Homo sapiens vasoactive intestinal peptide receptor 2 (VIPR2), mRNA [NM_003382]
0,26	0,00000	NMNAT3	NM_178177	Homo sapiens nicotinamide nucleotide adenylyltransferase 3 (NMNAT3), mRNA [NM_178177]
0,26	0,00016	MARCO	NM_006770	Homo sapiens macrophage receptor with collagenous structure (MARCO), mRNA [NM_006770]
0,26	0,00005	RP6-213H19.1	NM_016542	Homo sapiens serine/threonine protein kinase MST4 (MST4), transcript variant 1, mRNA [NM_016542]
0,26	0,00001	KDR	NM_002253	Homo sapiens kinase insert domain receptor (a type III receptor tyrosine kinase) (KDR), mRNA [NM_002253]
0,26	0,00000	ALDH8A1	NM_022568	Homo sapiens aldehyde dehydrogenase 8 family, member A1 (ALDH8A1), transcript variant 1, mRNA [NM_022568]
0,26	0,00005	FAM69B	NM_152421	Homo sapiens family with sequence similarity 69, member B (FAM69B), mRNA [NM_152421]
0,26	0,00183	ITGA7	NM_002206	Homo sapiens integrin, alpha 7 (ITGA7), mRNA [NM_002206]
0,26	0,00005	CXCR7	NM_020311	Homo sapiens chemokine (C-X-C motif) receptor 7 (CXCR7), transcript variant 2, mRNA [NM_020311]
0,26	0,00002	EPPK1	NM_031308	Homo sapiens epiplakin 1 (EPPK1), mRNA [NM_031308]
0,26	0,00004	GLT25D2	NM_015101	Homo sapiens glycosyltransferase 25 domain containing 2 (GLT25D2), mRNA [NM_015101]
0,26	0,00001	FAM110B	NM_147189	Homo sapiens family with sequence similarity 110, member B (FAM110B), mRNA [NM_147189]
0,26	0,00001	PPP1R9A	NM_017650	Homo sapiens protein phosphatase 1, regulatory (inhibitor) subunit 9A (PPP1R9A), mRNA [NM_017650]
0,26	0,00139	SLC39A8	NM_022154	Homo sapiens solute carrier family 39 (zinc transporter), member 8 (SLC39A8), mRNA [NM_022154]

0,26	0,00000	LITAF	NM_004862	Homo sapiens lipopolysaccharide-induced TNF factor (LITAF), mRNA [NM_004862]
0,26	0,00000	CCND2	NM_001759	Homo sapiens cyclin D2 (CCND2), mRNA [NM_001759]
0,26	0,00086	MAF	NM_005360	Homo sapiens v-maf musculoaponeurotic fibrosarcoma oncogene homolog (avian) (MAF), transcript variant 1, mRNA [NM_005360]
0,27	0,00000	CYFIP2	NM_014376	Homo sapiens cytoplasmic FMR1 interacting protein 2 (CYFIP2), transcript variant 3, mRNA [NM_014376]
0,27	0,00000	SPRR2D	NM_006945	Homo sapiens small proline-rich protein 2D (SPRR2D), mRNA [NM_006945]
0,27	0,00000	DAAM2	NM_015345	Homo sapiens dishevelled associated activator of morphogenesis 2 (DAAM2), mRNA [NM_015345]
0,27	0,00068	IL18	NM_001562	Homo sapiens interleukin 18 (interferon-gamma-inducing factor) (IL18), mRNA [NM_001562]
0,27	0,00001	GPR81	ENST00000356987	Probable G-protein coupled receptor 81 (G-protein coupled receptor 104). [Source:Uniprot/SWISSPROT;Acc:Q9BXC0] [ENST00000356987]
0,27	0,00000	WT1	NM_024424	Homo sapiens Wilms tumor 1 (WT1), transcript variant B, mRNA [NM_024424]
0,27	0,00000	INPP5D	NM_001017915	Homo sapiens inositol polyphosphate-5-phosphatase, 145kDa (INPP5D), transcript variant 1, mRNA [NM_001017915]
0,27	0,00002	CGNL1	NM_032866	Homo sapiens cingulin-like 1 (CGNL1), mRNA [NM_032866]
0,27	0,00000	D4S234E	NM_014392	Homo sapiens DNA segment on chromosome 4 (unique) 234 expressed sequence (D4S234E), transcript variant 1, mRNA [NM_014392]
0,28	0,00022	ECHDC3	NM_024693	Homo sapiens enoyl Coenzyme A hydratase domain containing 3 (ECHDC3), mRNA [NM_024693]
0,28	0,00000	C4BPA	NM_000715	Homo sapiens complement component 4 binding protein, alpha (C4BPA), mRNA [NM_000715]
0,28	0,00001	GDNF	ENST00000381826	Glial cell line-derived neurotrophic factor precursor (Astrocyte- derived trophic factor 1) (ATF-1). [Source:Uniprot/SWISSPROT;Acc:P39905] [ENST00000381826]
0,28	0,00001	Y10152	Y10152	H.sapiens mRNA for CRF2 receptor, beta isoform, aberrantly spliced, (94bp deletion). [Y10152]
0,28	0,00009	BAX	NM_138763	Homo sapiens BCL2-associated X protein (BAX), transcript variant delta, mRNA [NM_138763]
0,28	0,00003	LONRF2	NM_198461	Homo sapiens LON peptidase N-terminal domain and ring finger 2 (LONRF2), mRNA [NM_198461]
0,28	0,00009	SCRT2	NM_033129	Homo sapiens scratch homolog 2, zinc finger protein (Drosophila) (SCRT2), mRNA [NM_033129]

0,28	0,00004	BCHE	NM_000055	Homo sapiens butyrylcholinesterase (BCHE), mRNA [NM_000055]
0,28	0,00011	KCNK5	NM_003740	Homo sapiens potassium channel, subfamily K, member 5 (KCNK5), mRNA [NM_003740]
0,28	0,00001	KISS1R	NM_032551	Homo sapiens KISS1 receptor (KISS1R), mRNA [NM_032551]
0,28	0,00003	BMP7	NM_001719	Homo sapiens bone morphogenetic protein 7 (osteogenic protein 1) (BMP7), mRNA [NM_001719]
0,28	0,00000	ARHGDIG	NM_001176	Homo sapiens Rho GDP dissociation inhibitor (GDI) gamma (ARHGDIG), mRNA [NM_001176]
0,28	0,00004	ENST00000330598	ENST00000330598	Homo sapiens HSPC088 mRNA, partial cds. [AF161351]
0,28	0,00001	FLJ45717	NM_207401	Homo sapiens FLJ45717 protein (FLJ45717), mRNA [NM_207401]
0,28	0,00004	CCNYL1	NM_152523	Homo sapiens cyclin Y-like 1 (CCNYL1), mRNA [NM_152523]
0,28	0,00023	TESC	NM_017899	Homo sapiens tescalcin (TESC), mRNA [NM_017899]
0,28	0,00001	DLGAP3	NM_001080418	Homo sapiens discs, large (Drosophila) homolog-associated protein 3 (DLGAP3), mRNA [NM_001080418]
0,29	0,00000	XAGE2	NM_130777	Homo sapiens X antigen family, member 2 (XAGE2), mRNA [NM_130777]
0,29	0,00000	SLC13A3	NM_001011554	Homo sapiens solute carrier family 13 (sodium-dependent dicarboxylate transporter), member 3 (SLC13A3), transcript variant 2, mRNA [NM_001011554]
0,29	0,00000	DFNA5	NM_004403	Homo sapiens deafness, autosomal dominant 5 (DFNA5), mRNA [NM_004403]
0,29	0,00012	KIRREL2	NM_199180	Homo sapiens kin of IRRE like 2 (Drosophila) (KIRREL2), transcript variant 3, mRNA [NM_199180]
0,29	0,00001	SLC7A4	NM_004173	Homo sapiens solute carrier family 7 (cationic amino acid transporter, y+ system), member 4 (SLC7A4), mRNA [NM_004173]
0,29	0,00002	GBP2	NM_004120	Homo sapiens guanylate binding protein 2, interferon-inducible (GBP2), mRNA [NM_004120]
0,29	0,00004	TCEAL2	NM_080390	Homo sapiens transcription elongation factor A (SII)-like 2 (TCEAL2), mRNA [NM_080390]
0,29	0,00001	FAM110C	NM_001077710	Homo sapiens family with sequence similarity 110, member C (FAM110C), mRNA [NM_001077710]
0,29	0,00001	THC2522889	THC2522889	GPR27_HUMAN (Q9NS67) Probable G-protein coupled receptor 27 (Super conserved receptor expressed in brain 1), complete [THC2522889]
0,29	0,00000	LAMA5	NM_005560	Homo sapiens laminin, alpha 5 (LAMA5), mRNA [NM_005560]
0,29	0,00000	ARMC4	NM_018076	Homo sapiens armadillo repeat containing 4 (ARMC4), mRNA [NM_018076]
0,29	0,00005	GAL3ST1	NM_004861	Homo sapiens galactose-3-O-sulfotransferase 1 (GAL3ST1), mRNA [NM_004861]

0,30	0,00001	CCDC108	NM_194302	Homo sapiens coiled-coil domain containing 108 (CCDC108), transcript variant 1, mRNA [NM_194302]
0,30	0,00001	FLT3	NM_004119	Homo sapiens fms-related tyrosine kinase 3 (FLT3), mRNA [NM_004119]
0,30	0,00003	PAX8	NM_003466	Homo sapiens paired box gene 8 (PAX8), transcript variant PAX8A, mRNA [NM_003466]
0,30	0,00036	MGAT4C	NM_013244	Homo sapiens mannosyl (alpha-1,3-)-glycoprotein beta-1,4-N-acetylglucosaminyltransferase, isozyme C (putative) (MGAT4C), mRNA [NM_013244]
0,30	0,00000	LCE5A	NM_178438	Homo sapiens late cornified envelope 5A (LCE5A), mRNA [NM_178438]
0,30	0,00001	EN2	NM_001427	Homo sapiens engrailed homeobox 2 (EN2), mRNA [NM_001427]
0,30	0,00000	ATP7B	NM_000053	Homo sapiens ATPase, Cu++ transporting, beta polypeptide (ATP7B), transcript variant 1, mRNA [NM_000053]
0,30	0,00003	CACNA1E	NM_000721	Homo sapiens calcium channel, voltage-dependent, R type, alpha 1E subunit (CACNA1E), mRNA [NM_000721]
0,30	0,00000	HCN4	NM_005477	Homo sapiens hyperpolarization activated cyclic nucleotide-gated potassium channel 4 (HCN4), mRNA [NM_005477]
0,30	0,00000	WFDC2	NM_080736	Homo sapiens WAP four-disulfide core domain 2 (WFDC2), transcript variant 2, mRNA [NM_080736]
0,30	0,00002	NKX2-8	NM_014360	Homo sapiens NK2 transcription factor related, locus 8 (Drosophila) (NKX2-8), mRNA [NM_014360]
0,30	0,00144	NGFRAP1L1	NM_001012978	Homo sapiens NGFRAP1-like 1 (NGFRAP1L1), mRNA [NM_001012978]
0,30	0,00001	PRR15	NM_175887	Homo sapiens proline rich 15 (PRR15), mRNA [NM_175887]
0,30	0,00001	THC2603259	THC2603259	Q96IM5_HUMAN (Q96IM5) RAB7B protein, complete [THC2603259]
0,30	0,00001	GRIN2D	NM_000836	Homo sapiens glutamate receptor, ionotropic, N-methyl D-aspartate 2D (GRIN2D), mRNA [NM_000836]
0,30	0,00000	LRP2	NM_004525	Homo sapiens low density lipoprotein-related protein 2 (LRP2), mRNA [NM_004525]
0,30	0,00004	ZDHHC11	NM_024786	Homo sapiens zinc finger, DHHC-type containing 11 (ZDHHC11), mRNA [NM_024786]
0,30	0,00000	HSPA2	NM_021979	Homo sapiens heat shock 70kDa protein 2 (HSPA2), mRNA [NM_021979]
0,30	0,00163	SYNPO2L	NM_024875	Homo sapiens synaptopodin 2-like (SYNPO2L), mRNA [NM_024875]
0,30	0,00025	ITIH3	NM_002217	Homo sapiens inter-alpha (globulin) inhibitor H3 (ITIH3), mRNA [NM_002217]
0,30	0,00003	SYNGR4	NM_012451	Homo sapiens synaptogyrin 4 (SYNGR4), mRNA [NM_012451]

0,30	0,00006	ENPP5	NM_021572	Homo sapiens ectonucleotide pyrophosphatase/phosphodiesterase 5 (putative function) (ENPP5), mRNA [NM_021572]
0,31	0,00000	SPRY1	NM_199327	Homo sapiens sprouty homolog 1, antagonist of FGF signaling (Drosophila) (SPRY1), transcript variant 2, mRNA [NM_199327]
0,31	0,00001	RDH10	NM_172037	Homo sapiens retinol dehydrogenase 10 (all-trans) (RDH10), mRNA [NM_172037]
0,31	0,00001	CFI	NM_000204	Homo sapiens complement factor I (CFI), mRNA [NM_000204]
0,31	0,00000	DNM1DN8-2	AF357221	Homo sapiens FKSG88 mRNA, complete cds. [AF357221]
0,31	0,00001	REEP1	NM_022912	Homo sapiens receptor accessory protein 1 (REEP1), mRNA [NM_022912]
0,31	0,00001	TNFRSF8	NM_001243	Homo sapiens tumor necrosis factor receptor superfamily, member 8 (TNFRSF8), transcript variant 1, mRNA [NM_001243]
0,31	0,00000	NPAS3	NM_173159	Homo sapiens neuronal PAS domain protein 3 (NPAS3), transcript variant 2, mRNA [NM_173159]
0,31	0,00001	LRP3	NM_002333	Homo sapiens low density lipoprotein receptor-related protein 3 (LRP3), mRNA [NM_002333]
0,31	0,00001	PDIA2	NM_006849	Homo sapiens protein disulfide isomerase family A, member 2 (PDIA2), mRNA [NM_006849]
0,31	0,00017	PLEKHG3	NM_015549	Homo sapiens pleckstrin homology domain containing, family G (with RhoGef domain) member 3 (PLEKHG3), mRNA [NM_015549]
0,31	0,00003	KIAA0774	NM_001033602	Homo sapiens KIAA0774 (KIAA0774), transcript variant 1, mRNA [NM_001033602]
0,31	0,00024	TMEM176A	NM_018487	Homo sapiens transmembrane protein 176A (TMEM176A), mRNA [NM_018487]
0,31	0,00002	HSPB8	NM_014365	Homo sapiens heat shock 22kDa protein 8 (HSPB8), mRNA [NM_014365]
0,31	0,00006	MAFA	NM_201589	Homo sapiens v-maf musculoaponeurotic fibrosarcoma oncogene homolog A (avian) (MAFA), mRNA [NM_201589]
0,31	0,00008	ZFP36L2	NM_006887	Homo sapiens zinc finger protein 36, C3H type-like 2 (ZFP36L2), mRNA [NM_006887]
0,31	0,00000	SPPL2B	NM_001077238	Homo sapiens signal peptide peptidase-like 2B (SPPL2B), transcript variant 3, mRNA [NM_001077238]
0,31	0,00071	ENST00000343959	ENST00000343959	Protein FAM25. [Source:Uniprot/SWISSPROT;Acc:Q5VTM1] [ENST00000343959]
0,31	0,00002	THC2723346	THC2723346	Q5VZL5_HUMAN (Q5VZL5) Zinc finger protein 262, complete [THC2723346]
0,31	0,00007	C4B	NM_001002029	Homo sapiens complement component 4B (Childo blood group) (C4B), mRNA [NM_001002029]

0,31	0,00001	COX6A2	NM_005205	Homo sapiens cytochrome c oxidase subunit VIa polypeptide 2 (COX6A2), nuclear gene encoding mitochondrial protein, mRNA [NM_005205]
0,31	0,00003	ABCA3	NM_001089	Homo sapiens ATP-binding cassette, sub-family A (ABC1), member 3 (ABCA3), mRNA [NM_001089]
0,31	0,00018	CPVL	NM_031311	Homo sapiens carboxypeptidase, vitellogenic-like (CPVL), transcript variant 1, mRNA [NM_031311]
0,31	0,00001	FILIP1L	NM_182909	Homo sapiens filamin A interacting protein 1-like (FILIP1L), transcript variant 1, mRNA [NM_182909]
0,31	0,00001	SEMA3B	NM_004636	Homo sapiens sema domain, immunoglobulin domain (Ig), short basic domain, secreted, (semaphorin) 3B (SEMA3B), transcript variant 1, mRNA [NM_004636]
0,31	0,00006	CFH	NM_000186	Homo sapiens complement factor H (CFH), transcript variant 1, mRNA [NM_000186]
0,31	0,00001	A2BP1	NM_018723	Homo sapiens ataxin 2-binding protein 1 (A2BP1), transcript variant 4, mRNA [NM_018723]
0,31	0,00001	FCRLB	BC067080	Homo sapiens Fc receptor-like B, mRNA (cDNA clone MGC:71141 IMAGE:3529386), complete cds. [BC067080]
0,31	0,00002	KLK3	AF335478	Homo sapiens prostate-specific antigen variant 2 mRNA, complete cds, alternatively spliced. [AF335478]
0,31	0,00001	STON2	NM_033104	Homo sapiens stonin 2 (STON2), mRNA [NM_033104]
0,32	0,00464	GBP3	NM_018284	Homo sapiens guanylate binding protein 3 (GBP3), mRNA [NM_018284]
0,32	0,00017	HSPB9	NM_033194	Homo sapiens heat shock protein, alpha-crystallin-related, B9 (HSPB9), mRNA [NM_033194]
0,32	0,00000	CYFIP2	NM_001037332	Homo sapiens cytoplasmic FMR1 interacting protein 2 (CYFIP2), transcript variant 2, mRNA [NM_001037332]
0,32	0,00003	SYNPO	NM_007286	Homo sapiens synaptopodin (SYNPO), mRNA [NM_007286]
0,32	0,00000	RAB17	NM_022449	Homo sapiens RAB17, member RAS oncogene family (RAB17), mRNA [NM_022449]
0,32	0,00199	NAT8L	NM_178557	Homo sapiens N-acetyltransferase 8-like (NAT8L), mRNA [NM_178557]
0,32	0,00000	ABCA8	NM_007168	Homo sapiens ATP-binding cassette, sub-family A (ABC1), member 8 (ABCA8), mRNA [NM_007168]
0,32	0,00033	MRVI1	NM_130385	Homo sapiens murine retrovirus integration site 1 homolog (MRVI1), transcript variant 2, mRNA [NM_130385]
0,32	0,00006	LRRC1	NM_018214	Homo sapiens leucine rich repeat containing 1 (LRRC1), mRNA [NM_018214]

0,32	0,00002	AGXT2L1	NM_031279	Homo sapiens alanine-glyoxylate aminotransferase 2-like 1 (AGXT2L1), mRNA [NM_031279]
0,32	0,00000	DAPP1	NM_014395	Homo sapiens dual adaptor of phosphotyrosine and 3-phosphoinositides (DAPP1), mRNA [NM_014395]
0,32	0,00031	CDH3	NM_001793	Homo sapiens cadherin 3, type 1, P-cadherin (placental) (CDH3), mRNA [NM_001793]
0,32	0,00001	SOCS2	NM_003877	Homo sapiens suppressor of cytokine signaling 2 (SOCS2), mRNA [NM_003877]
0,32	0,00000	PHOX2A	NM_005169	Homo sapiens paired-like (aristaless) homeobox 2a (PHOX2A), mRNA [NM_005169]
0,32	0,00000	EPHB6	NM_004445	Homo sapiens EPH receptor B6 (EPHB6), mRNA [NM_004445]
0,32	0,00000	SERPING1	NM_000062	Homo sapiens serpin peptidase inhibitor, clade G (C1 inhibitor), member 1, (angioedema, hereditary) (SERPING1), transcript variant 1, mRNA [NM_000062]
0,33	0,00002	GPR150	NM_199243	Homo sapiens G protein-coupled receptor 150 (GPR150), mRNA [NM_199243]
0,33	0,00000	DMRTC1	NM_033053	Homo sapiens DMRT-like family C1 (DMRTC1), mRNA [NM_033053]
0,33	0,00008	EGLN3	NM_022073	Homo sapiens egl nine homolog 3 (C. elegans) (EGLN3), mRNA [NM_022073]
0,33	0,00006	DAB2	NM_001343	Homo sapiens disabled homolog 2, mitogen-responsive phosphoprotein (Drosophila) (DAB2), mRNA [NM_001343]
0,33	0,00000	HES7	NM_032580	Homo sapiens hairy and enhancer of split 7 (Drosophila) (HES7), mRNA [NM_032580]
0,33	0,00000	LBX1	NM_006562	Homo sapiens ladybird homeobox 1 (LBX1), mRNA [NM_006562]
0,33	0,00000	PPL	NM_002705	Homo sapiens periplakin (PPL), mRNA [NM_002705]
0,33	0,00001	FBP1	NM_000507	Homo sapiens fructose-1,6-bisphosphatase 1 (FBP1), mRNA [NM_000507]
0,33	0,00015	CR593784	CR593784	full-length cDNA clone CS0DI036YE11 of Placenta Cot 25-normalized of Homo sapiens (human) [CR593784]
0,33	0,00000	GPR153	NM_207370	Homo sapiens G protein-coupled receptor 153 (GPR153), mRNA [NM_207370]
0,33	0,00007	ENPP2	NM_006209	Homo sapiens ectonucleotide pyrophosphatase/phosphodiesterase 2 (autotaxin) (ENPP2), transcript variant 1, mRNA [NM_006209]
0,33	0,00018	DSCR1L1	NM_005822	Homo sapiens Down syndrome critical region gene 1-like 1 (DSCR1L1), mRNA [NM_005822]
0,33	0,00114	EGFL6	NM_015507	Homo sapiens EGF-like-domain, multiple 6 (EGFL6), mRNA [NM_015507]
0,33	0,00011	PDZD2	NM_178140	Homo sapiens PDZ domain containing 2 (PDZD2), mRNA [NM_178140]

0,33	0,00001	RBP1	NM_002899	Homo sapiens retinol binding protein 1, cellular (RBP1), mRNA [NM_002899]
0,33	0,00001	PDLIM1	NM_020992	Homo sapiens PDZ and LIM domain 1 (elfin) (PDLIM1), mRNA [NM_020992]
0,33	0,00000	ZNF579	NM_152600	Homo sapiens zinc finger protein 579 (ZNF579), mRNA [NM_152600]
0,33	0,00004	HS3ST1	NM_005114	Homo sapiens heparan sulfate (glucosamine) 3-O-sulfotransferase 1 (HS3ST1), mRNA [NM_005114]
0,33	0,00001	CLDN1	NM_021101	Homo sapiens claudin 1 (CLDN1), mRNA [NM_021101]
0,33	0,00001	FAM84B	NM_174911	Homo sapiens family with sequence similarity 84, member B (FAM84B), mRNA [NM_174911]
0,33	0,00001	ZSCAN10	NM_032805	Homo sapiens zinc finger and SCAN domain containing 10 (ZSCAN10), mRNA [NM_032805]
0,33	0,00000	DCDC2	NM_016356	Homo sapiens doublecortin domain containing 2 (DCDC2), mRNA [NM_016356]
0,33	0,00005	ENST00000371276	ENST00000371276	Tetratricopeptide repeat protein 22 (TPR repeat protein 22). [Source:Uniprot/SWISSPROT;Acc:Q5TAA0] [ENST00000371276]
0,33	0,00182	HSD3B1	NM_000862	Homo sapiens hydroxy-delta-5-steroid dehydrogenase, 3 beta- and steroid delta-isomerase 1 (HSD3B1), mRNA [NM_000862]
0,33	0,00000	ALDOA	NM_184041	Homo sapiens aldolase A, fructose-bisphosphate (ALDOA), transcript variant 2, mRNA [NM_184041]
0,34	0,00003	CYP2J2	NM_000775	Homo sapiens cytochrome P450, family 2, subfamily J, polypeptide 2 (CYP2J2), mRNA [NM_000775]
0,34	0,00001	CENTG3	NM_001042535	Homo sapiens centaurin, gamma 3 (CENTG3), transcript variant 2, mRNA [NM_001042535]
0,34	0,00183	NR4A3	NM_173198	Homo sapiens nuclear receptor subfamily 4, group A, member 3 (NR4A3), transcript variant 2, mRNA [NM_173198]
0,34	0,00009	B4GALT1	ENST00000379731	Beta-1,4-galactosyltransferase 1 (EC 2.4.1.-) (Beta-1,4-GalTase 1) (Beta4Gal-T1) (b4Gal-T1) (UDP-galactose:beta-N-acetylglucosamine beta-1,4-galactosyltransferase 1) (UDP-Gal:beta-GlcNAc beta-1,4-galactosyltransferase 1) [Includes: Lactose synthase...
0,34	0,00001	RHBDL1	NM_003961	Homo sapiens rhomboid, veinlet-like 1 (Drosophila) (RHBDL1), mRNA [NM_003961]
0,34	0,00001	KALRN	NM_003947	Homo sapiens kalirin, RhoGEF kinase (KALRN), transcript variant 2, mRNA [NM_003947]
0,34	0,00001	NLRP1	BC051787	Homo sapiens NLR family, pyrin domain containing 1, mRNA (cDNA clone MGC:57544 IMAGE:5756099), complete cds. [BC051787]
0,34	0,00004	ZNF331	NM_018555	Homo sapiens zinc finger protein 331 (ZNF331), transcript variant 1, mRNA [NM_018555]
0,34	0,00001	GALR3	NM_003614	Homo sapiens galanin receptor 3 (GALR3), mRNA [NM_003614]

0,34	0,00002	MX1	NM_002462	Homo sapiens myxovirus (influenza virus) resistance 1, interferon-inducible protein p78 (mouse) (MX1), mRNA [NM_002462]
0,34	0,00068	SFRP1	NM_003012	Homo sapiens secreted frizzled-related protein 1 (SFRP1), mRNA [NM_003012]
0,34	0,00010	ITGB2	NM_000211	Homo sapiens integrin, beta 2 (complement component 3 receptor 3 and 4 subunit) (ITGB2), mRNA [NM_000211]
0,34	0,00076	PARD6B	NM_032521	Homo sapiens par-6 partitioning defective 6 homolog beta (C. elegans) (PARD6B), mRNA [NM_032521]
0,34	0,00000	TMEM125	NM_144626	Homo sapiens transmembrane protein 125 (TMEM125), mRNA [NM_144626]
0,34	0,00010	FOXE1	X94553	H.sapiens HFKH4 mRNA for fork head like protein. [X94553]
0,34	0,00002	IFITM5	NM_001025295	Homo sapiens interferon induced transmembrane protein 5 (IFITM5), mRNA [NM_001025295]
0,34	0,00019	GPR156	NM_153002	Homo sapiens G protein-coupled receptor 156 (GPR156), mRNA [NM_153002]
0,34	0,00001	UTS2R	NM_018949	Homo sapiens urotensin 2 receptor (UTS2R), mRNA [NM_018949]
0,34	0,00000	ALDH1A2	NM_170697	Homo sapiens aldehyde dehydrogenase 1 family, member A2 (ALDH1A2), transcript variant 3, mRNA [NM_170697]
0,34	0,00005	EVX1	NM_001989	Homo sapiens even-skipped homeobox 1 (EVX1), mRNA [NM_001989]
0,34	0,00422	CALB2	NM_001740	Homo sapiens calbindin 2, 29kDa (calretinin) (CALB2), transcript variant CALB2, mRNA [NM_001740]
0,34	0,00000	CA11	NM_001217	Homo sapiens carbonic anhydrase XI (CA11), mRNA [NM_001217]
0,34	0,00001	NPBWR1	NM_005285	Homo sapiens neuropeptides B/W receptor 1 (NPBWR1), mRNA [NM_005285]
0,34	0,00007	ITGB4	NM_000213	Homo sapiens integrin, beta 4 (ITGB4), transcript variant 1, mRNA [NM_000213]
0,34	0,00015	RAB7B	NM_177403	Homo sapiens RAB7B, member RAS oncogene family (RAB7B), mRNA [NM_177403]
0,34	0,00066	ENST00000372045	ENST00000372045	Chordin-like protein 1 precursor (Neuralin-1) (Ventroptin) (Neurogenesin-1). [Source:Uniprot/SWISSPROT;Acc:Q9BU40] [ENST00000372045]
0,34	0,00003	LHX1	NM_005568	Homo sapiens LIM homeobox 1 (LHX1), mRNA [NM_005568]
0,34	0,00000	BCAN	BC005081	Homo sapiens brevican, mRNA (cDNA clone IMAGE:3618761), partial cds. [BC005081]
0,34	0,00002	AMHR2	NM_020547	Homo sapiens anti-Mullerian hormone receptor, type II (AMHR2), mRNA [NM_020547]
0,34	0,00000	ZFPM1	NM_153813	Homo sapiens zinc finger protein, multitype 1 (ZFPM1), mRNA [NM_153813]
0,34	0,00010	SLC27A2	NM_003645	Homo sapiens solute carrier family 27 (fatty acid transporter), member 2 (SLC27A2), mRNA [NM_003645]

0,34	0,00003	ETV7	NM_016135	Homo sapiens ets variant gene 7 (TEL2 oncogene) (ETV7), mRNA [NM_016135]
0,34	0,00000	RERG	NM_032918	Homo sapiens RAS-like, estrogen-regulated, growth inhibitor (RERG), mRNA [NM_032918]
0,34	0,00000	NPR3	NM_000908	Homo sapiens natriuretic peptide receptor C/guanylate cyclase C (atrionatriuretic peptide receptor C) (NPR3), mRNA [NM_000908]
0,34	0,00010	TSGA2	NM_080860	Homo sapiens testis specific A2 homolog (mouse) (TSGA2), mRNA [NM_080860]
0,34	0,00000	ZFP42	NM_174900	Homo sapiens zinc finger protein 42 homolog (mouse) (ZFP42), mRNA [NM_174900]
0,35	0,00008	GPRC5A	NM_003979	Homo sapiens G protein-coupled receptor, family C, group 5, member A (GPRC5A), mRNA [NM_003979]
0,35	0,00000	SCN2B	NM_004588	Homo sapiens sodium channel, voltage-gated, type II, beta (SCN2B), mRNA [NM_004588]
0,35	0,00004	KIAA1545	AF490258	Homo sapiens XTP9 (XTP9) mRNA, complete cds. [AF490258]
0,35	0,00001	DUX4	NM_033178	Homo sapiens double homeobox, 4 (DUX4), mRNA [NM_033178]
0,35	0,00018	DSCR1	NM_004414	Homo sapiens Down syndrome critical region gene 1 (DSCR1), transcript variant 1, mRNA [NM_004414]
0,35	0,00045	HTRA4	NM_153692	Homo sapiens HtrA serine peptidase 4 (HTRA4), mRNA [NM_153692]
0,35	0,00005	AVP	NM_000490	Homo sapiens arginine vasopressin (neurophysin II, antidiuretic hormone, diabetes insipidus, neurohypophyseal) (AVP), mRNA [NM_000490]
0,35	0,00000	HCN2	NM_001194	Homo sapiens hyperpolarization activated cyclic nucleotide-gated potassium channel 2 (HCN2), mRNA [NM_001194]
0,35	0,00005	SLC34A3	NM_080877	Homo sapiens solute carrier family 34 (sodium phosphate), member 3 (SLC34A3), mRNA [NM_080877]
0,35	0,00000	RASD1	NM_016084	Homo sapiens RAS, dexamethasone-induced 1 (RASD1), mRNA [NM_016084]
0,35	0,00000	TMEM16B	NM_020373	Homo sapiens transmembrane protein 16B (TMEM16B), mRNA [NM_020373]
0,35	0,00000	CHP	NM_007236	Homo sapiens calcium binding protein P22 (CHP), mRNA [NM_007236]
0,35	0,00005	NY-REN-7	AB018295	Homo sapiens mRNA for KIAA0752 protein, partial cds. [AB018295]
0,35	0,00000	ABCG1	NM_207630	Homo sapiens ATP-binding cassette, sub-family G (WHITE), member 1 (ABCG1), transcript variant 1, mRNA [NM_207630]
0,35	0,00001	TPSG1	NM_012467	Homo sapiens tryptase gamma 1 (TPSG1), mRNA [NM_012467]
0,35	0,00000	TCF21	NM_003206	Homo sapiens transcription factor 21 (TCF21), transcript variant 2, mRNA [NM_003206]

0,35	0,00000	POU3F3	NM_006236	Homo sapiens POU domain, class 3, transcription factor 3 (POU3F3), mRNA [NM_006236]
0,35	0,00004	CLU	NM_203339	Homo sapiens clusterin (CLU), transcript variant 2, mRNA [NM_203339]
0,35	0,00001	CXADR	NM_001338	Homo sapiens coxsackie virus and adenovirus receptor (CXADR), mRNA [NM_001338]
0,35	0,00006	CRYAB	NM_001885	Homo sapiens crystallin, alpha B (CRYAB), mRNA [NM_001885]
0,35	0,00000	SPINT1	NM_003710	Homo sapiens serine peptidase inhibitor, Kunitz type 1 (SPINT1), transcript variant 2, mRNA [NM_003710]
0,35	0,00006	TMEM66	NM_016127	Homo sapiens transmembrane protein 66 (TMEM66), mRNA [NM_016127]
0,36	0,00006	KIAA0746	NM_015187	Homo sapiens KIAA0746 protein (KIAA0746), mRNA [NM_015187]
0,36	0,00001	PRPH	NM_006262	Homo sapiens peripherin (PRPH), mRNA [NM_006262]
0,36	0,00095	FRY	NM_023037	Homo sapiens furry homolog (Drosophila) (FRY), mRNA [NM_023037]
0,36	0,00004	ZNF784	NM_203374	Homo sapiens zinc finger protein 784 (ZNF784), mRNA [NM_203374]
0,36	0,00002	LOC339809	ENST00000331807	Homo sapiens mRNA for KIAA2012 protein. [AB095932]
0,36	0,00003	TXNRD1	NM_003330	Homo sapiens thioredoxin reductase 1 (TXNRD1), transcript variant 1, mRNA [NM_003330]
0,36	0,00000	DUSP8	NM_004420	Homo sapiens dual specificity phosphatase 8 (DUSP8), mRNA [NM_004420]
0,36	0,00003	FOXB1	NM_012182	Homo sapiens forkhead box B1 (FOXB1), mRNA [NM_012182]
0,36	0,00006	TM7SF2	NM_003273	Homo sapiens transmembrane 7 superfamily member 2 (TM7SF2), mRNA [NM_003273]
0,36	0,00001	BC070091	BC070091	Homo sapiens caspase recruitment domain family, member 9, mRNA (cDNA clone MGC:87491 IMAGE:30343821), complete cds. [BC070091]
0,36	0,00001	VSIG2	NM_014312	Homo sapiens V-set and immunoglobulin domain containing 2 (VSIG2), mRNA [NM_014312]
0,36	0,00165	ENST00000299694	ENST00000299694	BEAN protein (Fragment). [Source:Uniprot/SPTREMBL;Acc:Q3B7T3] [ENST00000299694]
0,36	0,00000	CDC42EP5	NM_145057	Homo sapiens CDC42 effector protein (Rho GTPase binding) 5 (CDC42EP5), mRNA [NM_145057]
0,36	0,00002	NEUROG1	NM_006161	Homo sapiens neurogenin 1 (NEUROG1), mRNA [NM_006161]
0,36	0,00098	FLJ31568	NM_152509	Homo sapiens FLJ31568 protein (FLJ31568), mRNA [NM_152509]
0,36	0,00001	ENST00000324745	ENST00000324745	Homo sapiens mRNA for FLJ00388 protein. [AK090467]
0,36	0,00003	LCE1D	NM_178352	Homo sapiens late cornified envelope 1D (LCE1D), mRNA [NM_178352]
0,36	0,00241	ANXA8	BC008813	Homo sapiens annexin A8, mRNA (cDNA clone MGC:10405 IMAGE:3958020), complete cds. [BC008813]

0,36	0,00158	FBXL16	NM_153350	Homo sapiens F-box and leucine-rich repeat protein 16 (FBXL16), mRNA [NM_153350]
0,36	0,00000	NEDD9	NM_006403	Homo sapiens neural precursor cell expressed, developmentally down-regulated 9 (NEDD9), transcript variant 1, mRNA [NM_006403]
0,36	0,00001	CADM3	NM_021189	Homo sapiens cell adhesion molecule 3 (CADM3), mRNA [NM_021189]
0,36	0,00003	SRCRB4D	NM_080744	Homo sapiens scavenger receptor cysteine rich domain containing, group B (4 domains) (SRCRB4D), mRNA [NM_080744]
0,36	0,00000	ENST00000342773	ENST00000342773	Homo sapiens mRNA for FLJ00248 protein. [AK074175]
0,36	0,00049	METTL7A	NM_014033	Homo sapiens methyltransferase like 7A (METTL7A), mRNA [NM_014033]
0,36	0,00001	MMP15	NM_002428	Homo sapiens matrix metallopeptidase 15 (membrane-inserted) (MMP15), mRNA [NM_002428]
0,36	0,00000	LHFPL4	NM_198560	Homo sapiens lipoma HMGIC fusion partner-like 4 (LHFPL4), mRNA [NM_198560]
0,36	0,00001	FAM129B	NM_022833	Homo sapiens family with sequence similarity 129, member B (FAM129B), transcript variant 1, mRNA [NM_022833]
0,36	0,00012	CAND2	NM_012298	Homo sapiens cullin-associated and neddylation-dissociated 2 (putative) (CAND2), mRNA [NM_012298]
0,36	0,00006	GPRC5C	NM_022036	Homo sapiens G protein-coupled receptor, family C, group 5, member C (GPRC5C), transcript variant 1, mRNA [NM_022036]
0,37	0,00067	ANXA3	NM_005139	Homo sapiens annexin A3 (ANXA3), mRNA [NM_005139]
0,37	0,00008	SPATA2L	NM_152339	Homo sapiens spermatogenesis associated 2-like (SPATA2L), mRNA [NM_152339]
0,37	0,00007	PRG2	NM_024888	Homo sapiens plasticity-related gene 2 (PRG2), mRNA [NM_024888]
0,37	0,00000	MTUS1	NM_001001927	Homo sapiens mitochondrial tumor suppressor 1 (MTUS1), nuclear gene encoding mitochondrial protein, transcript variant 3, mRNA [NM_001001927]
0,37	0,00023	REC8	NM_005132	Homo sapiens REC8-like 1 (yeast) (REC8L1), transcript variant 1, mRNA [NM_005132]
0,37	0,00771	DISP2	NM_033510	Homo sapiens dispatched homolog 2 (Drosophila) (DISP2), mRNA [NM_033510]
0,37	0,00044	TMEM37	NM_183240	Homo sapiens transmembrane protein 37 (TMEM37), mRNA [NM_183240]
0,37	0,00001	RPRM	NM_019845	Homo sapiens reprimo, TP53 dependent G2 arrest mediator candidate (RPRM), mRNA [NM_019845]
0,37	0,00130	IBRDC2	NM_182757	Homo sapiens IBR domain containing 2 (IBRDC2), mRNA [NM_182757]
0,37	0,00575	GKN1	NM_019617	Homo sapiens gastrokine 1 (GKN1), mRNA [NM_019617]

0,37	0,00004	TSPAN3	NM_005724	Homo sapiens tetraspanin 3 (TSPAN3), transcript variant 1, mRNA [NM_005724]
0,37	0,00018	KRT8	NM_002273	Homo sapiens keratin 8 (KRT8), mRNA [NM_002273]
0,37	0,00014	CLSTN2	NM_022131	Homo sapiens calsyntenin 2 (CLSTN2), mRNA [NM_022131]
0,37	0,00000	SH3BP4	NM_014521	Homo sapiens SH3-domain binding protein 4 (SH3BP4), mRNA [NM_014521]
0,37	0,00001	PRNP	NM_001080122	Homo sapiens prion protein (p27-30) (Creutzfeldt-Jakob disease, Gerstmann-Strausler-Scheinker syndrome, fatal familial insomnia) (PRNP), transcript variant 4, mRNA [NM_001080122]
0,37	0,00003	SPANXA1	NM_013453	Homo sapiens sperm protein associated with the nucleus, X-linked, family member A1 (SPANXA1), mRNA [NM_013453]
0,37	0,00000	HAPLN1	NM_001884	Homo sapiens hyaluronan and proteoglycan link protein 1 (HAPLN1), mRNA [NM_001884]
0,37	0,00024	AY090769	AY090769	Homo sapiens ribosomal protein S18/S6-like mRNA, complete sequence. [AY090769]
0,37	0,00010	HS6ST2	NM_147175	Homo sapiens heparan sulfate 6-O-sulfotransferase 2 (HS6ST2), transcript variant S, mRNA [NM_147175]
0,37	0,00590	AKR1B1	NM_001628	Homo sapiens aldo-keto reductase family 1, member B1 (aldose reductase) (AKR1B1), mRNA [NM_001628]
0,37	0,00000	PCSK1N	NM_013271	Homo sapiens proprotein convertase subtilisin/kexin type 1 inhibitor (PCSK1N), mRNA [NM_013271]
0,37	0,00012	HRK	NM_003806	Homo sapiens harakiri, BCL2 interacting protein (contains only BH3 domain) (HRK), mRNA [NM_003806]
0,37	0,00016	GATA5	NM_080473	Homo sapiens GATA binding protein 5 (GATA5), mRNA [NM_080473]
0,37	0,00005	CD79A	NM_001783	Homo sapiens CD79a molecule, immunoglobulin-associated alpha (CD79A), transcript variant 1, mRNA [NM_001783]
0,37	0,00325	ITLN2	NM_080878	Homo sapiens intelectin 2 (ITLN2), mRNA [NM_080878]
0,37	0,00009	DBP	U06936	Human albumin D-box binding protein (DBP) mRNA, complete cds. [U06936]
0,37	0,00002	ENST00000326831	ENST00000326831	Probable phospholipid-transporting ATPase VB (EC 3.6.3.1). [Source:Uniprot/SWISSPROT;Acc:O94823] [ENST00000326831]
0,37	0,00000	BHLHB4	NM_080606	Homo sapiens basic helix-loop-helix domain containing, class B, 4 (BHLHB4), mRNA [NM_080606]
0,37	0,00003	SLC16A4	NM_004696	Homo sapiens solute carrier family 16, member 4 (monocarboxylic acid transporter 5) (SLC16A4), mRNA [NM_004696]
0,37	0,00004	ADRA1B	NM_000679	Homo sapiens adrenergic, alpha-1B-, receptor (ADRA1B), mRNA [NM_000679]
0,37	0,00010	RGN	NM_004683	Homo sapiens regucalcin (senescence marker protein-30) (RGN), transcript variant 1, mRNA [NM_004683]

0,37	0,00024	TMEM130	NM_152913	Homo sapiens transmembrane protein 130 (TMEM130), mRNA [NM_152913]
0,37	0,00000	WNT10A	NM_025216	Homo sapiens wingless-type MMTV integration site family, member 10A (WNT10A), mRNA [NM_025216]
0,37	0,00002	ENST00000219169	ENST00000219169	Nuclear transport factor 2 (NTF-2) (Placental protein 15) (PP15). [Source:Uniprot/SWISSPROT;Acc:P61970] [ENST00000219169]
0,38	0,00558	ESPNL	NM_194312	Homo sapiens espin-like (ESPNL), mRNA [NM_194312]
0,38	0,00000	CTNND2	NM_001332	Homo sapiens catenin (cadherin-associated protein), delta 2 (neural plakophilin-related arm-repeat protein) (CTNND2), mRNA [NM_001332]
0,38	0,00030	OSAP	NM_032623	Homo sapiens ovary-specific acidic protein (OSAP), mRNA [NM_032623]
0,38	0,00011	CDH10	NM_006727	Homo sapiens cadherin 10, type 2 (T2-cadherin) (CDH10), mRNA [NM_006727]
0,38	0,00000	SOX18	NM_018419	Homo sapiens SRY (sex determining region Y)-box 18 (SOX18), mRNA [NM_018419]
0,38	0,00000	BCAS1	NM_003657	Homo sapiens breast carcinoma amplified sequence 1 (BCAS1), mRNA [NM_003657]
0,38	0,00004	PDGFD	NM_025208	Homo sapiens platelet derived growth factor D (PDGFD), transcript variant 1, mRNA [NM_025208]
0,38	0,00000	IER5	NM_016545	Homo sapiens immediate early response 5 (IER5), mRNA [NM_016545]
0,38	0,00003	RNF182	NM_152737	Homo sapiens ring finger protein 182 (RNF182), mRNA [NM_152737]
0,38	0,00002	SYNGR2	NM_004710	Homo sapiens synaptogyrin 2 (SYNGR2), mRNA [NM_004710]
0,38	0,00039	IMPA2	NM_014214	Homo sapiens inositol(myo)-1(or 4)-monophosphatase 2 (IMPA2), mRNA [NM_014214]
0,38	0,00000	GPR92	NM_020400	Homo sapiens G protein-coupled receptor 92 (GPR92), mRNA [NM_020400]
0,38	0,00095	VIL2	NM_003379	Homo sapiens villin 2 (ezrin) (VIL2), mRNA [NM_003379]
0,38	0,00005	FAM19A5	NM_015381	Homo sapiens family with sequence similarity 19 (chemokine (C-C motif)-like), member A5 (FAM19A5), transcript variant 2, mRNA [NM_015381]
0,38	0,00256	KRT18	NM_000224	Homo sapiens keratin 18 (KRT18), transcript variant 1, mRNA [NM_000224]
0,38	0,00003	CYP4F2	NM_001082	Homo sapiens cytochrome P450, family 4, subfamily F, polypeptide 2 (CYP4F2), mRNA [NM_001082]
0,38	0,00000	LYNX1	NM_177458	Homo sapiens Ly6/neurotoxin 1 (LYNX1), transcript variant SLURP2, mRNA [NM_177458]
0,38	0,00005	ABLIM1	NM_001003408	Homo sapiens actin binding LIM protein 1 (ABLIM1), transcript variant 3, mRNA [NM_001003408]

0,38	0,00001	BCAM	NM_005581	Homo sapiens basal cell adhesion molecule (Lutheran blood group) (BCAM), transcript variant 1, mRNA [NM_005581]
0,38	0,00012	SCAMP5	NM_138967	Homo sapiens secretory carrier membrane protein 5 (SCAMP5), mRNA [NM_138967]
0,38	0,00221	SLC9A3R1	NM_004252	Homo sapiens solute carrier family 9 (sodium/hydrogen exchanger), member 3 regulator 1 (SLC9A3R1), mRNA [NM_004252]
0,38	0,00000	MANSC1	NM_018050	Homo sapiens MANSC domain containing 1 (MANSC1), mRNA [NM_018050]
0,38	0,00012	EXPH5	NM_015065	Homo sapiens exophilin 5 (EXPH5), mRNA [NM_015065]
0,38	0,00040	AW961597	AW961597	EST373669 MAGE resequences, MAGG Homo sapiens cDNA, mRNA sequence [AW961597]
0,38	0,00003	FCHO1	NM_015122	Homo sapiens FCH domain only 1 (FCHO1), mRNA [NM_015122]
0,38	0,00000	FAM3B	NM_058186	Homo sapiens family with sequence similarity 3, member B (FAM3B), transcript variant 1, mRNA [NM_058186]
0,38	0,00233	KRT33A	NM_004138	Homo sapiens keratin 33A (KRT33A), mRNA [NM_004138]
0,38	0,00034	STOX2	NM_020225	Homo sapiens storkhead box 2 (STOX2), mRNA [NM_020225]
0,38	0,00010	UTF1	NM_003577	Homo sapiens undifferentiated embryonic cell transcription factor 1 (UTF1), mRNA [NM_003577]
0,38	0,00001	TMEM151	NM_153266	Homo sapiens transmembrane protein 151 (TMEM151), mRNA [NM_153266]
0,38	0,00009	BTNL3	NM_197975	Homo sapiens butyrophilin-like 3 (BTNL3), transcript variant 1, mRNA [NM_197975]
0,39	0,00000	ACPL2	NM_152282	Homo sapiens acid phosphatase-like 2 (ACPL2), transcript variant 1, mRNA [NM_152282]
0,39	0,00007	BCL2	M13995	Human B-cell leukemia/lymphoma 2 (bcl-2) proto-oncogene mRNA encoding bcl-2-beta protein, complete cds. [M13995]
0,39	0,00001	HOOK1	NM_015888	Homo sapiens hook homolog 1 (Drosophila) (HOOK1), mRNA [NM_015888]
0,39	0,00018	K03200	K03200	Human melanoma-associated antigen p97 (melanotransferrin) mRNA, 3' flank. [K03200]
0,39	0,00362	KRT19P2	AB041269	Homo sapiens mRNA for keratin 19, partial cds, isolate:K19-141. [AB041269]
0,39	0,00000	HPD	NM_002150	Homo sapiens 4-hydroxyphenylpyruvate dioxygenase (HPD), mRNA [NM_002150]
0,39	0,00078	ARHGEF3	NM_019555	Homo sapiens Rho guanine nucleotide exchange factor (GEF) 3 (ARHGEF3), mRNA [NM_019555]
0,39	0,00003	RAB11FIP4	NM_032932	Homo sapiens RAB11 family interacting protein 4 (class II) (RAB11FIP4), mRNA [NM_032932]

0,39	0,00026	MMP17	NM_016155	Homo sapiens matrix metallopeptidase 17 (membrane-inserted) (MMP17), mRNA [NM_016155]
0,39	0,00003	SGPP2	NM_152386	Homo sapiens sphingosine-1-phosphate phosphotase 2 (SGPP2), mRNA [NM_152386]
0,39	0,00001	RIMS3	NM_014747	Homo sapiens regulating synaptic membrane exocytosis 3 (RIMS3), mRNA [NM_014747]
0,39	0,00000	GRB7	NM_005310	Homo sapiens growth factor receptor-bound protein 7 (GRB7), transcript variant 1, mRNA [NM_005310]
0,39	0,00011	PIP5K1B	NM_003558	Homo sapiens phosphatidylinositol-4-phosphate 5-kinase, type I, beta (PIP5K1B), transcript variant 2, mRNA [NM_003558]
0,39	0,00001	SPTAN1	NM_003127	Homo sapiens spectrin, alpha, non-erythrocytic 1 (alpha-fodrin) (SPTAN1), mRNA [NM_003127]
0,39	0,00006	MOP-1	AB014771	Homo sapiens mRNA for MOP-1, complete cds. [AB014771]
0,39	0,00003	AMN	NM_030943	Homo sapiens amnionless homolog (mouse) (AMN), mRNA [NM_030943]
0,39	0,00004	HLA-DPA1	NM_033554	Homo sapiens major histocompatibility complex, class II, DP alpha 1 (HLA-DPA1), mRNA [NM_033554]
0,39	0,00017	SEMA5A	NM_003966	Homo sapiens sema domain, seven thrombospondin repeats (type 1 and type 1-like), transmembrane domain (TM) and short cytoplasmic domain, (semaphorin) 5A (SEMA5A), mRNA [NM_003966]
0,39	0,00000	CDON	NM_016952	Homo sapiens Cdon homolog (mouse) (CDON), mRNA [NM_016952]
0,39	0,00002	PAK6	NM_020168	Homo sapiens p21(CDKN1A)-activated kinase 6 (PAK6), mRNA [NM_020168]
0,39	0,00014	GLT8D4	NM_001080393	Homo sapiens similar to CG9996-PA (LOC727936), mRNA [NM_001080393]
0,39	0,00837	RBP7	NM_052960	Homo sapiens retinol binding protein 7, cellular (RBP7), mRNA [NM_052960]
0,39	0,00007	SCARA3	NM_016240	Homo sapiens scavenger receptor class A, member 3 (SCARA3), transcript variant 1, mRNA [NM_016240]
0,39	0,00001	THSD3	NM_199265	Homo sapiens thrombospondin, type I, domain containing 3 (THSD3), transcript variant 2, mRNA [NM_199265]
0,39	0,00001	PALM	NM_002579	Homo sapiens paralemmin (PALM), transcript variant 1, mRNA [NM_002579]
0,40	0,00452	MAF	AF055376	Homo sapiens short form transcription factor C-MAF (c-maf) mRNA, complete cds. [AF055376]
0,40	0,00011	PRKCZ	NM_002744	Homo sapiens protein kinase C, zeta (PRKCZ), transcript variant 1, mRNA [NM_002744]
0,40	0,00073	DPYSL4	NM_006426	Homo sapiens dihydropyrimidinase-like 4 (DPYSL4), mRNA [NM_006426]

0,40	0,00000	DAPK1	NM_004938	Homo sapiens death-associated protein kinase 1 (DAPK1), mRNA [NM_004938]
0,40	0,00296	CYTL1	NM_018659	Homo sapiens cytokine-like 1 (CYTL1), mRNA [NM_018659]
0,40	0,00007	MYH2	NM_017534	Homo sapiens myosin, heavy chain 2, skeletal muscle, adult (MYH2), mRNA [NM_017534]
0,40	0,00102	MYOZ2	NM_016599	Homo sapiens myozenin 2 (MYOZ2), mRNA [NM_016599]
0,40	0,00047	PROCR	NM_006404	Homo sapiens protein C receptor, endothelial (EPCR) (PROCR), mRNA [NM_006404]
0,40	0,00000	SYT17	NM_016524	Homo sapiens synaptotagmin XVII (SYT17), mRNA [NM_016524]
0,40	0,00017	ADAMTS7	NM_014272	Homo sapiens ADAM metallopeptidase with thrombospondin type 1 motif, 7 (ADAMTS7), mRNA [NM_014272]
0,40	0,00002	RTN3	NM_201430	Homo sapiens reticulon 3 (RTN3), transcript variant 4, mRNA [NM_201430]
0,40	0,00001	TAS2R1	NM_019599	Homo sapiens taste receptor, type 2, member 1 (TAS2R1), mRNA [NM_019599]
0,40	0,00000	ERRFI1	NM_018948	Homo sapiens ERBB receptor feedback inhibitor 1 (ERRFI1), mRNA [NM_018948]
0,40	0,00003	JUP	NM_002230	Homo sapiens junction plakoglobin (JUP), transcript variant 1, mRNA [NM_002230]
0,40	0,00003	KIAA1394	BC036557	Homo sapiens KIAA1394 protein, mRNA (cDNA clone IMAGE:4310128), complete cds. [BC036557]
0,40	0,00033	MST1R	NM_002447	Homo sapiens macrophage stimulating 1 receptor (c-met-related tyrosine kinase) (MST1R), mRNA [NM_002447]
0,40	0,00001	GBP1	NM_002053	Homo sapiens guanylate binding protein 1, interferon-inducible, 67kDa (GBP1), mRNA [NM_002053]
0,40	0,00017	FRAS1	NM_025074	Homo sapiens Fraser syndrome 1 (FRAS1), mRNA [NM_025074]
0,40	0,00000	ADAMTS3	NM_014243	Homo sapiens ADAM metallopeptidase with thrombospondin type 1 motif, 3 (ADAMTS3), mRNA [NM_014243]
0,40	0,00023	NUAK2	NM_030952	Homo sapiens NUAK family, SNF1-like kinase, 2 (NUAK2), mRNA [NM_030952]
0,40	0,00005	FZD3	ENST00000380239	Frizzled-3 precursor (Fz-3) (hFz3). [Source:Uniprot/SWISSPROT;Acc:Q9NPG1] [ENST00000380239]
0,40	0,00094	SEMA5A	ENST00000382496	Semaphorin-5A precursor (Semaphorin F) (Sema F). [Source:Uniprot/SWISSPROT;Acc:Q13591] [ENST00000382496]
0,40	0,00007	TMEM98	NM_015544	Homo sapiens transmembrane protein 98 (TMEM98), transcript variant 1, mRNA [NM_015544]
0,40	0,00000	RAET1E	NM_139165	Homo sapiens retinoic acid early transcript 1E (RAET1E), mRNA [NM_139165]

0,40	0,00000	ZNF205	NM_003456	Homo sapiens zinc finger protein 205 (ZNF205), transcript variant 1, mRNA [NM_003456]
0,41	0,00019	BARX1	NM_021570	Homo sapiens BarH-like homeobox 1 (BARX1), mRNA [NM_021570]
0,41	0,00002	SF3A2	NM_007165	Homo sapiens splicing factor 3a, subunit 2, 66kDa (SF3A2), mRNA [NM_007165]
0,41	0,00002	ARRB2	NM_004313	Homo sapiens arrestin, beta 2 (ARRB2), transcript variant 1, mRNA [NM_004313]
0,41	0,00046	AOX1	NM_001159	Homo sapiens aldehyde oxidase 1 (AOX1), mRNA [NM_001159]
0,41	0,00000	DLG2	NM_001364	Homo sapiens discs, large homolog 2, chapsyn-110 (Drosophila) (DLG2), mRNA [NM_001364]
0,41	0,00032	FOXC2	NM_005251	Homo sapiens forkhead box C2 (MFH-1, mesenchyme forkhead 1) (FOXC2), mRNA [NM_005251]
0,41	0,00004	SCD5	NM_001037582	Homo sapiens stearoyl-CoA desaturase 5 (SCD5), transcript variant 1, mRNA [NM_001037582]
0,41	0,00000	TRH	NM_007117	Homo sapiens thyrotropin-releasing hormone (TRH), mRNA [NM_007117]
0,41	0,00062	GABARAPL1	NM_031412	Homo sapiens GABA(A) receptor-associated protein like 1 (GABARAPL1), mRNA [NM_031412]
0,41	0,00043	IGSF9	NM_020789	Homo sapiens immunoglobulin superfamily, member 9 (IGSF9), mRNA [NM_020789]
0,41	0,00263	SULT1A1	NM_177529	Homo sapiens sulfotransferase family, cytosolic, 1A, phenol-preferring, member 1 (SULT1A1), transcript variant 2, mRNA [NM_177529]
0,41	0,00000	KCNK1	NM_002245	Homo sapiens potassium channel, subfamily K, member 1 (KCNK1), mRNA [NM_002245]
0,41	0,00000	HEPACAM	ENST00000298251	hepatocyte cell adhesion molecule [Source:RefSeq_peptide;Acc:NP_689935] [ENST00000298251]
0,41	0,00014	KIRREL3	ENST00000278934	Kin of IRRE-like protein 3 precursor (Kin of irregular chiasm-like protein 3) (Nephrin-like 2). [Source:Uniprot/SWISSPROT;Acc:Q8IZU9] [ENST00000278934]
0,41	0,00004	IRF5	NM_002200	Homo sapiens interferon regulatory factor 5 (IRF5), transcript variant 1, mRNA [NM_002200]
0,41	0,00006	UCN2	NM_033199	Homo sapiens urocortin 2 (UCN2), mRNA [NM_033199]
0,41	0,00018	PCDHA8	NM_031856	Homo sapiens protocadherin alpha 8 (PCDHA8), transcript variant 2, mRNA [NM_031856]
0,41	0,00004	CFD	NM_001928	Homo sapiens complement factor D (adipsin) (CFD), mRNA [NM_001928]
0,41	0,00619	ENST00000299997	ENST00000299997	MGC9913 protein. [Source:Uniprot/SPTREMBL;Acc:Q96HF5] [ENST00000299997]

0,41	0,00148	JAK3	BC028068	Homo sapiens Janus kinase 3 (a protein tyrosine kinase, leukocyte), mRNA (cDNA clone MGC:39993 IMAGE:5212575), complete cds. [BC028068]
0,41	0,00002	ALDH3B1	NM_000694	Homo sapiens aldehyde dehydrogenase 3 family, member B1 (ALDH3B1), transcript variant 1, mRNA [NM_000694]
0,41	0,00001	CILP	NM_003613	Homo sapiens cartilage intermediate layer protein, nucleotide pyrophosphohydrolase (CILP), mRNA [NM_003613]
0,41	0,00001	EPHX2	NM_001979	Homo sapiens epoxide hydrolase 2, cytoplasmic (EPHX2), mRNA [NM_001979]
0,41	0,00002	TRAPPC6A	NM_024108	Homo sapiens trafficking protein particle complex 6A (TRAPPC6A), mRNA [NM_024108]
0,41	0,00001	CBX7	NM_175709	Homo sapiens chromobox homolog 7 (CBX7), mRNA [NM_175709]
0,41	0,00223	SALL4	NM_020436	Homo sapiens sal-like 4 (Drosophila) (SALL4), mRNA [NM_020436]
0,41	0,00008	MID1	NM_033290	Homo sapiens midline 1 (Opitz/BBB syndrome) (MID1), transcript variant 3, mRNA [NM_033290]
0,42	0,00000	KIAA1305	NM_025081	Homo sapiens KIAA1305 (KIAA1305), mRNA [NM_025081]
0,42	0,00005	OPLAH	NM_017570	Homo sapiens 5-oxoprolinase (ATP-hydrolysing) (OPLAH), mRNA [NM_017570]
0,42	0,00001	KCND3	ENST00000369697	Potassium voltage-gated channel subfamily D member 3 (Voltage-gated potassium channel subunit Kv4.3). [Source:Uniprot/SWISSPROT;Acc:Q9UK17] [ENST00000369697]
0,42	0,00002	EXOC3L2	NM_138568	Homo sapiens exocyst complex component 3-like 2 (EXOC3L2), mRNA [NM_138568]
0,42	0,00060	IGFBP4	NM_001552	Homo sapiens insulin-like growth factor binding protein 4 (IGFBP4), mRNA [NM_001552]
0,42	0,00002	RP5-860F19.3	BC054347	Homo sapiens KIAA1442 protein, mRNA (cDNA clone IMAGE:5502800), complete cds. [BC054347]
0,42	0,00010	KARCA1	NM_001007255	Homo sapiens kelch/ankyrin repeat containing cyclin A1 interacting protein (KARCA1), transcript variant 2, mRNA [NM_001007255]
0,42	0,00004	FSCN1	NM_003088	Homo sapiens fascin homolog 1, actin-bundling protein (Strongylocentrotus purpuratus) (FSCN1), mRNA [NM_003088]
0,42	0,00000	BMP7	ENST00000371291	Bone morphogenetic protein 7 precursor (BMP-7) (Osteogenic protein 1) (OP-1) (Eptotermin alfa). [Source:Uniprot/SWISSPROT;Acc:P18075] [ENST00000371291]
0,42	0,00004	MST1	NM_020998	Homo sapiens macrophage stimulating 1 (hepatocyte growth factor-like) (MST1), mRNA [NM_020998]
0,42	0,00000	NKD2	NM_033120	Homo sapiens naked cuticle homolog 2 (Drosophila) (NKD2), mRNA [NM_033120]

0,42	0,00006	ADRA2C	NM_000683	Homo sapiens adrenergic, alpha-2C-, receptor (ADRA2C), mRNA [NM_000683]
0,42	0,00289	PDGFRB	NM_002609	Homo sapiens platelet-derived growth factor receptor, beta polypeptide (PDGFRB), mRNA [NM_002609]
0,42	0,00632	KRT7	NM_005556	Homo sapiens keratin 7 (KRT7), mRNA [NM_005556]
0,42	0,00452	MYO5B	NM_001080467	Homo sapiens myosin VB (MYO5B), mRNA [NM_001080467]
0,42	0,00045	CARHSP1	NM_014316	Homo sapiens calcium regulated heat stable protein 1, 24kDa (CARHSP1), transcript variant 1, mRNA [NM_014316]
0,42	0,00005	RDH13	NM_138412	Homo sapiens retinol dehydrogenase 13 (all-trans/9-cis) (RDH13), mRNA [NM_138412]
0,42	0,00015	CTAGE4	AF338232	Homo sapiens CTAGE-4 protein mRNA, complete cds. [AF338232]
0,42	0,00002	P2RY2	NM_176072	Homo sapiens purinergic receptor P2Y, G-protein coupled, 2 (P2RY2), transcript variant 1, mRNA [NM_176072]
0,42	0,00004	KIAA0427	NM_014772	Homo sapiens KIAA0427 (KIAA0427), mRNA [NM_014772]
0,42	0,00000	PACSIN1	NM_020804	Homo sapiens protein kinase C and casein kinase substrate in neurons 1 (PACSIN1), mRNA [NM_020804]
0,43	0,00029	CENTG2	NM_014914	Homo sapiens centaurin, gamma 2 (CENTG2), transcript variant 2, mRNA [NM_014914]
0,43	0,00035	NP511100	NP511100	GB|AB065467.1|BAC05726.1 seven transmembrane helix receptor [Homo sapiens] [NP511100]
0,43	0,00009	MATN2	NM_030583	Homo sapiens matrilin 2 (MATN2), transcript variant 2, mRNA [NM_030583]
0,43	0,00003	PDGFRL	NM_006207	Homo sapiens platelet-derived growth factor receptor-like (PDGFRL), mRNA [NM_006207]
0,43	0,00003	CD244	NM_016382	Homo sapiens CD244 molecule, natural killer cell receptor 2B4 (CD244), mRNA [NM_016382]
0,43	0,00023	CXCL16	NM_022059	Homo sapiens chemokine (C-X-C motif) ligand 16 (CXCL16), mRNA [NM_022059]
0,43	0,00087	NID2	NM_007361	Homo sapiens nidogen 2 (osteonidogen) (NID2), mRNA [NM_007361]
0,43	0,00000	PLLP	NM_015993	Homo sapiens transmembrane 4 superfamily member 11 (plasmolipin) (TM4SF11), mRNA [NM_015993]
0,43	0,00004	AGRN	NM_198576	Homo sapiens agrin (AGRN), mRNA [NM_198576]
0,43	0,00434	SULF2	NM_018837	Homo sapiens sulfatase 2 (SULF2), transcript variant 1, mRNA [NM_018837]
0,43	0,00034	EFNA1	NM_004428	Homo sapiens ephrin-A1 (EFNA1), transcript variant 1, mRNA [NM_004428]
0,43	0,00006	SLC16A1	NM_003051	Homo sapiens solute carrier family 16, member 1 (monocarboxylic acid transporter 1) (SLC16A1), mRNA [NM_003051]
0,43	0,00001	OLFML1	NM_198474	Homo sapiens olfactomedin-like 1 (OLFML1), mRNA [NM_198474]

0,43	0,00021	DAGLB	NM_139179	Homo sapiens diacylglycerol lipase, beta (DAGLB), mRNA [NM_139179]
0,43	0,00003	FHL1	NM_001449	Homo sapiens four and a half LIM domains 1 (FHL1), mRNA [NM_001449]
0,43	0,00005	DUSP15	NM_080611	Homo sapiens dual specificity phosphatase 15 (DUSP15), transcript variant 1, mRNA [NM_080611]
0,43	0,00003	ARHGAP28	NM_001010000	Homo sapiens Rho GTPase activating protein 28 (ARHGAP28), transcript variant 1, mRNA [NM_001010000]
0,43	0,00263	NLRP2	NM_017852	Homo sapiens NLR family, pyrin domain containing 2 (NLRP2), mRNA [NM_017852]
0,43	0,00006	GRIN1	NM_021569	Homo sapiens glutamate receptor, ionotropic, N-methyl D-aspartate 1 (GRIN1), transcript variant NR1-2, mRNA [NM_021569]
0,43	0,00005	GNRH1	NM_000825	Homo sapiens gonadotropin-releasing hormone 1 (luteinizing-releasing hormone) (GNRH1), transcript variant 1, mRNA [NM_000825]
0,43	0,00023	CACNA1B	M94173	Human N-type calcium channel alpha-1 subunit mRNA, complete cds. [M94173]
0,43	0,00000	ATP6V1G2	NM_130463	Homo sapiens ATPase, H+ transporting, lysosomal 13kDa, V1 subunit G2 (ATP6V1G2), transcript variant 1, mRNA [NM_130463]
0,43	0,00001	ZNF687	NM_020832	Homo sapiens zinc finger protein 687 (ZNF687), mRNA [NM_020832]
0,43	0,00098	ALDH1B1	NM_000692	Homo sapiens aldehyde dehydrogenase 1 family, member B1 (ALDH1B1), nuclear gene encoding mitochondrial protein, mRNA [NM_000692]
0,43	0,00014	SELENBP1	NM_003944	Homo sapiens selenium binding protein 1 (SELENBP1), mRNA [NM_003944]
0,43	0,00052	SULT1A2	NM_177528	Homo sapiens sulfotransferase family, cytosolic, 1A, phenol-preferring, member 2 (SULT1A2), transcript variant 2, mRNA [NM_177528]
0,43	0,00006	FLJ21963	NM_024560	Homo sapiens FLJ21963 protein (FLJ21963), mRNA [NM_024560]
0,43	0,00001	HOXA5	NM_019102	Homo sapiens homeobox A5 (HOXA5), mRNA [NM_019102]
0,43	0,00045	FAM131C	NM_182623	Homo sapiens family with sequence similarity 131, member C (FAM131C), mRNA [NM_182623]
0,43	0,00035	MAP3K8	NM_005204	Homo sapiens mitogen-activated protein kinase kinase kinase 8 (MAP3K8), mRNA [NM_005204]
0,43	0,00013	PPME1	NM_016147	Homo sapiens protein phosphatase methylesterase 1 (PPME1), mRNA [NM_016147]
0,43	0,00002	TEPP	NM_199456	Homo sapiens testis/prostate/placenta-expressed protein (TEPP), transcript variant 2, mRNA [NM_199456]
0,43	0,00000	NFYB	NM_006166	Homo sapiens nuclear transcription factor Y, beta (NFYB), mRNA [NM_006166]
0,43	0,00029	CTSF	NM_003793	Homo sapiens cathepsin F (CTSF), mRNA [NM_003793]

0,43	0,00001	ENSA	NM_207042	Homo sapiens endosulfine alpha (ENSA), transcript variant 1, mRNA [NM_207042]
0,43	0,00001	MAP1LC3A	NM_032514	Homo sapiens microtubule-associated protein 1 light chain 3 alpha (MAP1LC3A), transcript variant 1, mRNA [NM_032514]
0,43	0,00000	TMEM108	NM_023943	Homo sapiens transmembrane protein 108 (TMEM108), mRNA [NM_023943]
0,43	0,00318	IGJ	NM_144646	Homo sapiens immunoglobulin J polypeptide, linker protein for immunoglobulin alpha and mu polypeptides (IGJ), mRNA [NM_144646]
0,44	0,00001	PRR5	NM_015366	Homo sapiens proline rich 5 (renal) (PRR5), transcript variant 2, mRNA [NM_015366]
0,44	0,00000	PRSS16	NM_005865	Homo sapiens protease, serine, 16 (thymus) (PRSS16), mRNA [NM_005865]
0,44	0,00028	SEMA6B	NM_032108	Homo sapiens sema domain, transmembrane domain (TM), and cytoplasmic domain, (semaphorin) 6B (SEMA6B), transcript variant SEMA6B.3, mRNA [NM_032108]
0,44	0,00021	HSPA1A	NM_005345	Homo sapiens heat shock 70kDa protein 1A (HSPA1A), mRNA [NM_005345]
0,44	0,00094	RETSAT	NM_017750	Homo sapiens retinol saturase (all-trans-retinol 13,14-reductase) (RETSAT), mRNA [NM_017750]
0,44	0,00002	SMARCD3	NM_003078	Homo sapiens SWI/SNF related, matrix associated, actin dependent regulator of chromatin, subfamily d, member 3 (SMARCD3), transcript variant 2, mRNA [NM_003078]
0,44	0,00013	OPA3	NM_025136	Homo sapiens optic atrophy 3 (autosomal recessive, with chorea and spastic paraplegia) (OPA3), transcript variant 2, mRNA [NM_025136]
0,44	0,00014	ITPR1	NM_002222	Homo sapiens inositol 1,4,5-triphosphate receptor, type 1 (ITPR1), mRNA [NM_002222]
0,44	0,00001	GLYATL1	NM_080661	Homo sapiens glycine-N-acyltransferase-like 1 (GLYATL1), mRNA [NM_080661]
0,44	0,00004	ABHD4	NM_022060	Homo sapiens abhydrolase domain containing 4 (ABHD4), mRNA [NM_022060]
0,44	0,00006	ANKRD15	NM_153186	Homo sapiens ankyrin repeat domain 15 (ANKRD15), transcript variant 2, mRNA [NM_153186]
0,44	0,00007	MAN1C1	NM_020379	Homo sapiens mannosidase, alpha, class 1C, member 1 (MAN1C1), mRNA [NM_020379]
0,44	0,00004	GPC3	NM_004484	Homo sapiens glypican 3 (GPC3), mRNA [NM_004484]
0,44	0,00000	PIK3CD	NM_005026	Homo sapiens phosphoinositide-3-kinase, catalytic, delta polypeptide (PIK3CD), mRNA [NM_005026]
0,44	0,00002	SEMA3E	NM_012431	Homo sapiens sema domain, immunoglobulin domain (Ig), short basic domain, secreted, (semaphorin) 3E (SEMA3E), mRNA [NM_012431]

0,44	0,00002	PPM1J	NM_005167	Homo sapiens protein phosphatase 1J (PP2C domain containing) (PPM1J), mRNA [NM_005167]
0,44	0,00000	ORMDL3	NM_139280	Homo sapiens ORM1-like 3 (S. cerevisiae) (ORMDL3), mRNA [NM_139280]
0,44	0,00032	EEF2	NM_001961	Homo sapiens eukaryotic translation elongation factor 2 (EEF2), mRNA [NM_001961]
0,44	0,00011	FAM120C	NM_017848	Homo sapiens family with sequence similarity 120C (FAM120C), mRNA [NM_017848]
0,44	0,00002	LY6E	NM_002346	Homo sapiens lymphocyte antigen 6 complex, locus E (LY6E), mRNA [NM_002346]
0,44	0,00024	GJB2	NM_004004	Homo sapiens gap junction protein, beta 2, 26kDa (GJB2), mRNA [NM_004004]
0,44	0,00000	STIM1	NM_003156	Homo sapiens stromal interaction molecule 1 (STIM1), mRNA [NM_003156]
0,44	0,00002	NELL1	NM_006157	Homo sapiens NEL-like 1 (chicken) (NELL1), mRNA [NM_006157]
0,44	0,00023	CTAGE5	NM_203356	Homo sapiens CTAGE family, member 5 (CTAGE5), transcript variant 4, mRNA [NM_203356]
0,44	0,00121	CEBPD	NM_005195	Homo sapiens CCAAT/enhancer binding protein (C/EBP), delta (CEBPD), mRNA [NM_005195]
0,45	0,00067	PCDHB11	NM_018931	Homo sapiens protocadherin beta 11 (PCDHB11), mRNA [NM_018931]
0,45	0,00001	CKB	NM_001823	Homo sapiens creatine kinase, brain (CKB), mRNA [NM_001823]
0,45	0,00002	KRT80	NM_182507	Homo sapiens keratin 80 (KRT80), transcript variant 1, mRNA [NM_182507]
0,45	0,00001	PH-4	NM_017732	Homo sapiens hypoxia-inducible factor prolyl 4-hydroxylase (PH-4), transcript variant 2, mRNA [NM_017732]
0,45	0,00002	YPEL1	NM_013313	Homo sapiens yippee-like 1 (Drosophila) (YPEL1), mRNA [NM_013313]
0,45	0,00013	KIAA1505	NM_020879	Homo sapiens KIAA1505 protein (KIAA1505), mRNA [NM_020879]
0,45	0,00006	LCE1C	NM_178351	Homo sapiens late cornified envelope 1C (LCE1C), mRNA [NM_178351]
0,45	0,00084	PEX10	NM_002617	Homo sapiens peroxisome biogenesis factor 10 (PEX10), transcript variant 2, mRNA [NM_002617]
0,45	0,00007	RASSF4	NM_032023	Homo sapiens Ras association (RalGDS/AF-6) domain family 4 (RASSF4), mRNA [NM_032023]
0,45	0,00000	BTG2	NM_006763	Homo sapiens BTG family, member 2 (BTG2), mRNA [NM_006763]
0,45	0,00001	PYGM	ENST00000377444	Glycogen phosphorylase, muscle form (EC 2.4.1.1) (Myophosphorylase). [Source:Uniprot/SWISSPROT;Acc:P11217] [ENST00000377444]

0,45	0,00000	CXCL10	NM_001565	Homo sapiens chemokine (C-X-C motif) ligand 10 (CXCL10), mRNA [NM_001565]
0,45	0,00012	SLC35E2	NM_182838	Homo sapiens solute carrier family 35, member E2 (SLC35E2), mRNA [NM_182838]
0,45	0,00028	GSDML	NM_018530	Homo sapiens gasdermin-like (GSDML), transcript variant 2, mRNA [NM_018530]
0,45	0,00005	SDC4	NM_002999	Homo sapiens syndecan 4 (SDC4), mRNA [NM_002999]
0,45	0,00018	ZFHX2	AB051549	Homo sapiens mRNA for KIAA1762 protein, partial cds. [AB051549]
0,45	0,00025	PLXNB1	NM_002673	Homo sapiens plexin B1 (PLXNB1), mRNA [NM_002673]
0,45	0,00011	TMEM97	NM_014573	Homo sapiens transmembrane protein 97 (TMEM97), mRNA [NM_014573]
0,45	0,00001	FOXD3	NM_012183	Homo sapiens forkhead box D3 (FOXD3), mRNA [NM_012183]
0,45	0,00420	WWC1	NM_015238	Homo sapiens WW and C2 domain containing 1 (WWC1), mRNA [NM_015238]
0,45	0,00025	IGFBP6	NM_002178	Homo sapiens insulin-like growth factor binding protein 6 (IGFBP6), mRNA [NM_002178]
0,45	0,00113	SALL2	NM_005407	Homo sapiens sal-like 2 (Drosophila) (SALL2), mRNA [NM_005407]
0,45	0,00001	MYCN	NM_005378	Homo sapiens v-myc myelocytomatosis viral related oncogene, neuroblastoma derived (avian) (MYCN), mRNA [NM_005378]
0,45	0,00107	CNIH2	NM_182553	Homo sapiens cornichon homolog 2 (Drosophila) (CNIH2), mRNA [NM_182553]
0,45	0,00000	MMP24	NM_006690	Homo sapiens matrix metallopeptidase 24 (membrane-inserted) (MMP24), mRNA [NM_006690]
0,45	0,00044	ARRDC4	NM_183376	Homo sapiens arrestin domain containing 4 (ARRDC4), mRNA [NM_183376]
0,45	0,00003	NY-REN-7	NM_173663	Homo sapiens NY-REN-7 antigen (NY-REN-7), mRNA [NM_173663]
0,45	0,00003	MYOD1	NM_002478	Homo sapiens myogenic differentiation 1 (MYOD1), mRNA [NM_002478]
0,45	0,00093	RNF122	NM_024787	Homo sapiens ring finger protein 122 (RNF122), mRNA [NM_024787]
0,45	0,00000	FOSL2	ENST00000379616	Fos-related antigen 2. [Source:Uniprot/SWISSPROT;Acc:P15408] [ENST00000379616]
0,45	0,00001	OGFR	NM_007346	Homo sapiens opioid growth factor receptor (OGFR), mRNA [NM_007346]
0,45	0,00000	ANXA11	ENST00000360615	Annexin A11 (Annexin XI) (Calcyclin-associated annexin 50) (CAP-50) (56 kDa autoantigen). [Source:Uniprot/SWISSPROT;Acc:P50995] [ENST00000360615]
0,45	0,00000	SLC37A3	NM_207113	Homo sapiens solute carrier family 37 (glycerol-3-phosphate transporter), member 3 (SLC37A3), transcript variant 1, mRNA [NM_207113]

0,46	0,00023	MTL5	NM_004923	Homo sapiens metallothionein-like 5, testis-specific (tesmin) (MTL5), transcript variant 1, mRNA [NM_004923]
0,46	0,00001	TP53INP2	NM_021202	Homo sapiens tumor protein p53 inducible nuclear protein 2 (TP53INP2), mRNA [NM_021202]
0,46	0,00025	DISP1	NM_032890	Homo sapiens dispatched homolog 1 (Drosophila) (DISP1), mRNA [NM_032890]
0,46	0,00000	CABC1	NM_020247	Homo sapiens chaperone, ABC1 activity of bc1 complex homolog (S. pombe) (CABC1), nuclear gene encoding mitochondrial protein, mRNA [NM_020247]
0,46	0,00011	GCHFR	NM_005258	Homo sapiens GTP cyclohydrolase I feedback regulator (GCHFR), mRNA [NM_005258]
0,46	0,00002	DHCR24	NM_014762	Homo sapiens 24-dehydrocholesterol reductase (DHCR24), mRNA [NM_014762]
0,46	0,00005	HAGHL	NM_032304	Homo sapiens hydroxyacylglutathione hydrolase-like (HAGHL), transcript variant 2, mRNA [NM_032304]
0,46	0,00001	PYGM	NM_005609	Homo sapiens phosphorylase, glycogen; muscle (McArdle syndrome, glycogen storage disease type V) (PYGM), mRNA [NM_005609]
0,46	0,00000	TGFB3	NM_003239	Homo sapiens transforming growth factor, beta 3 (TGFB3), mRNA [NM_003239]
0,46	0,00001	ARTN	NM_057091	Homo sapiens artemin (ARTN), transcript variant 2, mRNA [NM_057091]
0,46	0,00002	RPS6KA3	NM_004586	Homo sapiens ribosomal protein S6 kinase, 90kDa, polypeptide 3 (RPS6KA3), mRNA [NM_004586]
0,46	0,00004	ATAD3C	NM_001039211	Homo sapiens ATPase family, AAA domain containing 3C (ATAD3C), mRNA [NM_001039211]
0,46	0,00001	TMPRSS3	NM_032401	Homo sapiens transmembrane protease, serine 3 (TMPRSS3), transcript variant B, mRNA [NM_032401]
0,46	0,00026	DLGAP1	NM_004746	Homo sapiens discs, large (Drosophila) homolog-associated protein 1 (DLGAP1), transcript variant alpha, mRNA [NM_004746]
0,46	0,00000	EDG4	NM_004720	Homo sapiens endothelial differentiation, lysophosphatidic acid G-protein-coupled receptor, 4 (EDG4), mRNA [NM_004720]
0,46	0,00002	HHAT	NM_018194	Homo sapiens hedgehog acyltransferase (HHAT), mRNA [NM_018194]
0,46	0,00000	NELL2	NM_006159	Homo sapiens NEL-like 2 (chicken) (NELL2), mRNA [NM_006159]
0,46	0,00231	DKFZP586H2123	NM_015430	Homo sapiens regeneration associated muscle protease (DKFZP586H2123), transcript variant 1, mRNA [NM_015430]
0,46	0,00068	RBP4	NM_006744	Homo sapiens retinol binding protein 4, plasma (RBP4), mRNA [NM_006744]
0,46	0,00000	SPRR2C	NR_003062	Homo sapiens small proline-rich protein 2C (SPRR2C) on chromosome 1 [NR_003062]

0,46	0,00002	CASKIN1	NM_020764	Homo sapiens CASK interacting protein 1 (CASKIN1), mRNA [NM_020764]
0,46	0,00019	DAB2IP	NM_032552	Homo sapiens DAB2 interacting protein (DAB2IP), transcript variant 1, mRNA [NM_032552]
0,46	0,00009	BAI1	NM_001702	Homo sapiens brain-specific angiogenesis inhibitor 1 (BAI1), mRNA [NM_001702]
0,46	0,00001	LLGL2	NM_001015002	Homo sapiens lethal giant larvae homolog 2 (Drosophila) (LLGL2), transcript variant 2, mRNA [NM_001015002]
0,46	0,00068	SNRPN	NM_022807	Homo sapiens small nuclear ribonucleoprotein polypeptide N (SNRPN), transcript variant 4, mRNA [NM_022807]
0,46	0,00001	ACTN4	NM_004924	Homo sapiens actinin, alpha 4 (ACTN4), mRNA [NM_004924]
0,46	0,00452	ANKRD35	NM_144698	Homo sapiens ankyrin repeat domain 35 (ANKRD35), mRNA [NM_144698]
0,46	0,00746	AF159295	AF159295	Homo sapiens serine/threonine protein kinase Kp78 splice variant CTAK75a mRNA, complete cds. [AF159295]
0,46	0,00002	MARCKSL1	NM_023009	Homo sapiens MARCKS-like 1 (MARCKSL1), mRNA [NM_023009]
0,46	0,00002	IGFALS	NM_004970	Homo sapiens insulin-like growth factor binding protein, acid labile subunit (IGFALS), mRNA [NM_004970]
0,47	0,00024	ANKRD55	NM_024669	Homo sapiens ankyrin repeat domain 55 (ANKRD55), transcript variant 1, mRNA [NM_024669]
0,47	0,00036	SYNGR2	BC105992	Homo sapiens synaptogyrin 2, mRNA (cDNA clone MGC:102914 IMAGE:4746277), complete cds. [BC105992]
0,47	0,00001	FAM119A	NM_145280	Homo sapiens family with sequence similarity 119, member A (FAM119A), mRNA [NM_145280]
0,47	0,00010	GEFT	NM_182947	Homo sapiens RAC/CDC42 exchange factor (GEFT), transcript variant 1, mRNA [NM_182947]
0,47	0,00002	SLC15A3	NM_016582	Homo sapiens solute carrier family 15, member 3 (SLC15A3), mRNA [NM_016582]
0,47	0,00073	GALNTL2	NM_054110	Homo sapiens UDP-N-acetyl-alpha-D-galactosamine:polypeptide N-acetylgalactosaminyltransferase-like 2 (GALNTL2), mRNA [NM_054110]
0,47	0,00000	HADH	NM_005327	Homo sapiens hydroxyacyl-Coenzyme A dehydrogenase (HADH), nuclear gene encoding mitochondrial protein, mRNA [NM_005327]
0,47	0,00020	RNF175	NM_173662	Homo sapiens ring finger protein 175 (RNF175), mRNA [NM_173662]
0,47	0,00004	NLF2	NM_001007595	Homo sapiens nuclear localized factor 2 (NLF2), mRNA [NM_001007595]
0,47	0,00853	BTBD11	NM_152322	Homo sapiens BTB (POZ) domain containing 11 (BTBD11), transcript variant 1, mRNA [NM_152322]

0,47	0,00002	IMMP2L	NM_032549	Homo sapiens IMP2 inner mitochondrial membrane peptidase-like (S. cerevisiae) (IMMP2L), mRNA [NM_032549]
0,47	0,00003	C1QTNF1	NM_198594	Homo sapiens C1q and tumor necrosis factor related protein 1 (C1QTNF1), mRNA [NM_198594]
0,47	0,00006	LOH11CR2A	NM_014622	Homo sapiens loss of heterozygosity, 11, chromosomal region 2, gene A (LOH11CR2A), transcript variant 1, mRNA [NM_014622]
0,47	0,00192	GPR78	NM_080819	Homo sapiens G protein-coupled receptor 78 (GPR78), mRNA [NM_080819]
0,47	0,00563	EMILIN2	NM_032048	Homo sapiens elastin microfibril interfacer 2 (EMILIN2), mRNA [NM_032048]
0,47	0,00083	NOXA1	NM_006647	Homo sapiens NADPH oxidase activator 1 (NOXA1), mRNA [NM_006647]
0,47	0,00009	ADAM15	NM_207191	Homo sapiens ADAM metallopeptidase domain 15 (metargidin) (ADAM15), transcript variant 1, mRNA [NM_207191]
0,47	0,00006	ZNF395	NM_018660	Homo sapiens zinc finger protein 395 (ZNF395), mRNA [NM_018660]
0,47	0,00015	RGS16	NM_002928	Homo sapiens regulator of G-protein signalling 16 (RGS16), mRNA [NM_002928]
0,47	0,00010	PBXIP1	NM_020524	Homo sapiens pre-B-cell leukemia homeobox interacting protein 1 (PBXIP1), mRNA [NM_020524]
0,47	0,00011	DDR1	NM_013994	Homo sapiens discoidin domain receptor family, member 1 (DDR1), transcript variant 3, mRNA [NM_013994]
0,47	0,00004	CKMT1B	NM_020990	Homo sapiens creatine kinase, mitochondrial 1B (CKMT1B), nuclear gene encoding mitochondrial protein, mRNA [NM_020990]
0,47	0,00183	ID3	NM_002167	Homo sapiens inhibitor of DNA binding 3, dominant negative helix-loop-helix protein (ID3), mRNA [NM_002167]
0,47	0,00005	LFNG	NM_001040168	Homo sapiens LFNG O-fucosylpeptide 3-beta-N-acetylglucosaminyltransferase (LFNG), transcript variant 2, mRNA [NM_001040168]
0,47	0,00000	FOSL2	NM_005253	Homo sapiens FOS-like antigen 2 (FOSL2), mRNA [NM_005253]
0,47	0,00000	SP5	NM_001003845	Homo sapiens Sp5 transcription factor (SP5), mRNA [NM_001003845]
0,47	0,00001	ST14	NM_021978	Homo sapiens suppression of tumorigenicity 14 (colon carcinoma) (ST14), mRNA [NM_021978]
0,47	0,00591	NFE2L1	L24123	Homo sapiens NRF1 protein (NRF1) mRNA. [L24123]
0,47	0,00570	PLCE1	NM_016341	Homo sapiens phospholipase C, epsilon 1 (PLCE1), mRNA [NM_016341]
0,47	0,00000	ABCB1	NM_000927	Homo sapiens ATP-binding cassette, sub-family B (MDR/TAP), member 1 (ABCB1), mRNA [NM_000927]
0,47	0,00000	LSR	NM_205834	Homo sapiens lipolysis stimulated lipoprotein receptor (LSR), transcript variant 2, mRNA [NM_205834]

0,47	0,00008	GHR	NM_000163	Homo sapiens growth hormone receptor (GHR), mRNA [NM_000163]
0,47	0,00745	ENST00000330640	ENST00000330640	KIAA1244 (KIAA1244), mRNA [Source:RefSeq_dna;Acc:NM_020340] [ENST00000330640]
0,47	0,00005	SVEP1	NM_153366	Homo sapiens sushi, von Willebrand factor type A, EGF and pentraxin domain containing 1 (SVEP1), mRNA [NM_153366]
0,47	0,00001	WSCD1	NM_015253	Homo sapiens KIAA0523 protein (KIAA0523), mRNA [NM_015253]
0,48	0,00001	MESP1	NM_018670	Homo sapiens mesoderm posterior 1 homolog (mouse) (MESP1), mRNA [NM_018670]
0,48	0,00004	AIM1L	NM_017977	Homo sapiens absent in melanoma 1-like (AIM1L), mRNA [NM_017977]
0,48	0,00013	SHB	NM_003028	Homo sapiens Src homology 2 domain containing adaptor protein B (SHB), mRNA [NM_003028]
0,48	0,00107	AP2M1	NM_004068	Homo sapiens adaptor-related protein complex 2, mu 1 subunit (AP2M1), transcript variant 1, mRNA [NM_004068]
0,48	0,00122	LRFN1	ENST00000248668	Leucine-rich repeat and fibronectin type III domain-containing protein 1 (Fragment). [Source:Uniprot/SPTREMBL;Acc:Q9P244] [ENST00000248668]
0,48	0,00036	ZNF330	NM_014487	Homo sapiens zinc finger protein 330 (ZNF330), mRNA [NM_014487]
0,48	0,00000	NANOS1	NM_199461	Homo sapiens nanos homolog 1 (Drosophila) (NANOS1), transcript variant 1, mRNA [NM_199461]
0,48	0,00078	ARL14	NM_025047	Homo sapiens ADP-ribosylation factor-like 14 (ARL14), mRNA [NM_025047]
0,48	0,00077	IL28RA	NM_170743	Homo sapiens interleukin 28 receptor, alpha (interferon, lambda receptor) (IL28RA), transcript variant 1, mRNA [NM_170743]
0,48	0,00021	AMIGO1	ENST00000369864	Amphoterin-induced protein 1 precursor (AMIGO-1) (Alivin-2). [Source:Uniprot/SWISSPROT;Acc:Q86WK6] [ENST00000369864]
0,48	0,00095	IFI6	NM_022873	Homo sapiens interferon, alpha-inducible protein 6 (IFI6), transcript variant 3, mRNA [NM_022873]
0,48	0,00050	NLF1	NM_207322	Homo sapiens nuclear localized factor 1 (NLF1), mRNA [NM_207322]
0,48	0,00000	UTS2	NM_021995	Homo sapiens urotensin 2 (UTS2), transcript variant 1, mRNA [NM_021995]
0,48	0,00001	SLC2A12	NM_145176	Homo sapiens solute carrier family 2 (facilitated glucose transporter), member 12 (SLC2A12), mRNA [NM_145176]
0,48	0,00008	LBH	NM_030915	Homo sapiens limb bud and heart development homolog (mouse) (LBH), mRNA [NM_030915]
0,48	0,00021	OSBPL3	NM_015550	Homo sapiens oxysterol binding protein-like 3 (OSBPL3), transcript variant 1, mRNA [NM_015550]
0,48	0,00001	VAV1	NM_005428	Homo sapiens vav 1 oncogene (VAV1), mRNA [NM_005428]

0,48	0,00625	RAMP1	NM_005855	Homo sapiens receptor (G protein-coupled) activity modifying protein 1 (RAMP1), mRNA [NM_005855]
0,48	0,00065	MGAT4A	NM_012214	Homo sapiens mannosyl (alpha-1,3-)-glycoprotein beta-1,4-N-acetylglucosaminyltransferase, isozyme A (MGAT4A), mRNA [NM_012214]
0,48	0,00004	LLGL2	NM_004524	Homo sapiens lethal giant larvae homolog 2 (Drosophila) (LLGL2), transcript variant 1, mRNA [NM_004524]
0,48	0,00858	IL6ST	NM_002184	Homo sapiens interleukin 6 signal transducer (gp130, oncostatin M receptor) (IL6ST), transcript variant 1, mRNA [NM_002184]
0,48	0,00000	PTPN6	NM_002831	Homo sapiens protein tyrosine phosphatase, non-receptor type 6 (PTPN6), transcript variant 1, mRNA [NM_002831]
0,48	0,00037	FBXO34	AF531436	Homo sapiens CGI-301 protein mRNA, complete cds. [AF531436]
0,48	0,00002	KLHL23	NM_144711	Homo sapiens kelch-like 23 (Drosophila) (KLHL23), mRNA [NM_144711]
0,48	0,00001	ALDH6A1	NM_005589	Homo sapiens aldehyde dehydrogenase 6 family, member A1 (ALDH6A1), nuclear gene encoding mitochondrial protein, mRNA [NM_005589]
0,48	0,00014	ISG20	NM_002201	Homo sapiens interferon stimulated exonuclease gene 20kDa (ISG20), mRNA [NM_002201]
0,48	0,00001	TINAGL1	NM_022164	Homo sapiens tubulointerstitial nephritis antigen-like 1 (TINAGL1), mRNA [NM_022164]
0,48	0,00539	AS3MT	NM_020682	Homo sapiens arsenic (+3 oxidation state) methyltransferase (AS3MT), mRNA [NM_020682]
0,48	0,00005	ASS1	NM_054012	Homo sapiens argininosuccinate synthetase 1 (ASS1), transcript variant 2, mRNA [NM_054012]
0,48	0,00023	TLE1	NM_005077	Homo sapiens transducin-like enhancer of split 1 (E(sp1) homolog, Drosophila) (TLE1), mRNA [NM_005077]
0,48	0,00056	FAM47C	NM_001013736	Homo sapiens family with sequence similarity 47, member C (FAM47C), mRNA [NM_001013736]
0,48	0,00001	X92493	X92493	H.sapiens mRNA for STM-7 protein. [X92493]
0,49	0,00014	ANXA9	NM_003568	Homo sapiens annexin A9 (ANXA9), mRNA [NM_003568]
0,49	0,00009	WIT1	NM_015855	Homo sapiens Wilms tumor upstream neighbor 1 (WIT1), mRNA [NM_015855]
0,49	0,00000	WNT6	NM_006522	Homo sapiens wingless-type MMTV integration site family, member 6 (WNT6), mRNA [NM_006522]
0,49	0,00000	RNF10	NM_014868	Homo sapiens ring finger protein 10 (RNF10), mRNA [NM_014868]
0,49	0,00022	REPS2	NM_004726	Homo sapiens RALBP1 associated Eps domain containing 2 (REPS2), transcript variant 1, mRNA [NM_004726]
0,49	0,00020	NXN	NM_022463	Homo sapiens nucleoredoxin (NXN), mRNA [NM_022463]

0,49	0,00010	CMTM8	NM_178868	Homo sapiens CKLF-like MARVEL transmembrane domain containing 8 (CMTM8), mRNA [NM_178868]
0,49	0,00420	CDKN2B	NM_078487	Homo sapiens cyclin-dependent kinase inhibitor 2B (p15, inhibits CDK4) (CDKN2B), transcript variant 2, mRNA [NM_078487]
0,49	0,00017	CPA1	NM_001868	Homo sapiens carboxypeptidase A1 (pancreatic) (CPA1), mRNA [NM_001868]
0,49	0,00136	SEC14L4	NM_174977	Homo sapiens SEC14-like 4 (S. cerevisiae) (SEC14L4), mRNA [NM_174977]
0,49	0,00000	EPB41L4B	NM_018424	Homo sapiens erythrocyte membrane protein band 4.1 like 4B (EPB41L4B), transcript variant 1, mRNA [NM_018424]
0,49	0,00005	GABRB3	NM_000814	Homo sapiens gamma-aminobutyric acid (GABA) A receptor, beta 3 (GABRB3), transcript variant 1, mRNA [NM_000814]
0,49	0,00050	CPXM1	NM_019609	Homo sapiens carboxypeptidase X (M14 family), member 1 (CPXM1), mRNA [NM_019609]
0,49	0,00006	CMIP	NM_198390	Homo sapiens c-Maf-inducing protein (CMIP), transcript variant C-mip, mRNA [NM_198390]
0,49	0,00001	RAB6B	NM_016577	Homo sapiens RAB6B, member RAS oncogene family (RAB6B), mRNA [NM_016577]
0,49	0,00036	PRPF19	NM_014502	Homo sapiens PRP19/PSO4 pre-mRNA processing factor 19 homolog (S. cerevisiae) (PRPF19), mRNA [NM_014502]
0,49	0,00000	GFRA4	NM_022139	Homo sapiens GDNF family receptor alpha 4 (GFRA4), transcript variant 1, mRNA [NM_022139]
0,49	0,00006	FUCA1	NM_000147	Homo sapiens fucosidase, alpha-L- 1, tissue (FUCA1), mRNA [NM_000147]
0,49	0,00011	PPFIBP2	NM_003621	Homo sapiens PTPRF interacting protein, binding protein 2 (liprin beta 2) (PPFIBP2), mRNA [NM_003621]
0,49	0,00000	STX11	NM_003764	Homo sapiens syntaxin 11 (STX11), mRNA [NM_003764]
0,49	0,00000	CALCOCO1	NM_020898	Homo sapiens calcium binding and coiled-coil domain 1 (CALCOCO1), mRNA [NM_020898]
0,49	0,00001	ARHGEF5	NM_005435	Homo sapiens Rho guanine nucleotide exchange factor (GEF) 5 (ARHGEF5), mRNA [NM_005435]
0,49	0,00003	KHDRBS3	NM_006558	Homo sapiens KH domain containing, RNA binding, signal transduction associated 3 (KHDRBS3), mRNA [NM_006558]
0,49	0,00059	LMOD1	NM_012134	Homo sapiens leiomodin 1 (smooth muscle) (LMOD1), mRNA [NM_012134]
0,49	0,00149	ALDH3A2	NM_000382	Homo sapiens aldehyde dehydrogenase 3 family, member A2 (ALDH3A2), transcript variant 2, mRNA [NM_000382]
0,49	0,00000	SLITRK2	NM_032539	Homo sapiens SLIT and NTRK-like family, member 2 (SLITRK2), mRNA [NM_032539]

0,49	0,00052	GPRC5B	NM_016235	Homo sapiens G protein-coupled receptor, family C, group 5, member B (GPRC5B), mRNA [NM_016235]
0,49	0,00125	PAPPA	NM_002581	Homo sapiens pregnancy-associated plasma protein A, pappalysin 1 (PAPPA), mRNA [NM_002581]
0,49	0,00403	CLGN	NM_004362	Homo sapiens calmegin (CLGN), mRNA [NM_004362]
0,49	0,00015	EPSTI1	ENST00000313624	epithelial stromal interaction 1 isoform 2 [Source:RefSeq_peptide;Acc:NP_150280] [ENST00000313624]
0,49	0,00008	STAP2	NM_001013841	Homo sapiens signal-transducing adaptor protein-2 (STAP2), transcript variant 2, mRNA [NM_001013841]
0,49	0,00000	PDK2	NM_002611	Homo sapiens pyruvate dehydrogenase kinase, isozyme 2 (PDK2), mRNA [NM_002611]
0,49	0,00094	KCTD14	NM_023930	Homo sapiens potassium channel tetramerisation domain containing 14 (KCTD14), mRNA [NM_023930]
0,49	0,00498	PYY2	NR_003064	Homo sapiens peptide YY, 2 (seminalplasmin) (PYY2) on chromosome 17 [NR_003064]
0,49	0,00002	MID1	ENST00000380780	Midline-1 (EC 6.3.2.-) (Tripartite motif-containing protein 18) (Putative transcription factor XPRF) (Midin) (RING finger protein 59) (Midline 1 RING finger protein). [Source:Uniprot/SWISSPROT;Acc:O15344] [ENST00000380780]
0,49	0,00001	NUB1	NM_016118	Homo sapiens negative regulator of ubiquitin-like proteins 1 (NUB1), mRNA [NM_016118]
0,49	0,00000	WDFY4	BC032420	Homo sapiens WDFY family member 4, mRNA (cDNA clone MGC:40604 IMAGE:5221804), complete cds. [BC032420]
0,49	0,00016	RDM1	NM_145654	Homo sapiens RAD52 motif 1 (RDM1), transcript variant 1, mRNA [NM_145654]
0,49	0,00061	IFI27	NM_005532	Homo sapiens interferon, alpha-inducible protein 27 (IFI27), mRNA [NM_005532]
0,49	0,00036	FBXO16	NM_172366	Homo sapiens F-box protein 16 (FBXO16), mRNA [NM_172366]
0,49	0,00008	PTMS	NM_002824	Homo sapiens parathymosin (PTMS), mRNA [NM_002824]
0,49	0,00013	TSPAN10	NM_031945	Homo sapiens tetraspanin 10 (TSPAN10), mRNA [NM_031945]
0,49	0,00056	GPR56	NM_201525	Homo sapiens G protein-coupled receptor 56 (GPR56), transcript variant 3, mRNA [NM_201525]
0,49	0,00074	SLC30A2	NM_001004434	Homo sapiens solute carrier family 30 (zinc transporter), member 2 (SLC30A2), transcript variant 1, mRNA [NM_001004434]
0,49	0,00817	PSG7	NM_002783	Homo sapiens pregnancy specific beta-1-glycoprotein 7 (PSG7), mRNA [NM_002783]
0,49	0,00478	CCDC71	NM_022903	Homo sapiens coiled-coil domain containing 71 (CCDC71), mRNA [NM_022903]

0,49	0,00007	USP2	BC041366	Homo sapiens ubiquitin specific peptidase 2, mRNA (cDNA clone MGC:43844 IMAGE:5273400), complete cds. [BC041366]
0,49	0,00006	RHOB	NM_004040	Homo sapiens ras homolog gene family, member B (RHOB), mRNA [NM_004040]
0,49	0,00001	CACNA2D3	AF516696	Homo sapiens voltage-gated calcium channel alpha(2)delta-3 subunit mRNA, complete cds. [AF516696]
0,49	0,00034	TNFRSF21	NM_014452	Homo sapiens tumor necrosis factor receptor superfamily, member 21 (TNFRSF21), mRNA [NM_014452]
0,49	0,00054	TLR1	NM_003263	Homo sapiens toll-like receptor 1 (TLR1), mRNA [NM_003263]
0,50	0,00239	SULT1A4	NM_001017389	Homo sapiens sulfotransferase family, cytosolic, 1A, phenol-preferring, member 4 (SULT1A4), transcript variant 1, mRNA [NM_001017389]
0,50	0,00004	TMEM118	NM_032814	Homo sapiens transmembrane protein 118 (TMEM118), mRNA [NM_032814]
0,50	0,00046	GABARAPL3	AF180519	Homo sapiens GABA-A receptor-associated protein mRNA, complete cds. [AF180519]
0,50	0,00009	CTNNBIP1	NM_020248	Homo sapiens catenin, beta interacting protein 1 (CTNNBIP1), transcript variant 1, mRNA [NM_020248]
0,50	0,00000	KCNH2	NM_172056	Homo sapiens potassium voltage-gated channel, subfamily H (eag-related), member 2 (KCNH2), transcript variant 2, mRNA [NM_172056]
0,50	0,00023	TNFAIP2	NM_006291	Homo sapiens tumor necrosis factor, alpha-induced protein 2 (TNFAIP2), mRNA [NM_006291]
0,50	0,00011	GMDS	NM_001500	Homo sapiens GDP-mannose 4,6-dehydratase (GMDS), mRNA [NM_001500]
0,50	0,00023	LAMA3	NM_198129	Homo sapiens laminin, alpha 3 (LAMA3), transcript variant 1, mRNA [NM_198129]
0,50	0,00078	VLDLR	NM_003383	Homo sapiens very low density lipoprotein receptor (VLDLR), transcript variant 1, mRNA [NM_003383]
0,50	0,00168	HIST2H2AA3	NM_003516	Homo sapiens histone cluster 2, H2aa3 (HIST2H2AA3), mRNA [NM_003516]
0,50	0,00000	DEAF1	NM_021008	Homo sapiens deformed epidermal autoregulatory factor 1 (Drosophila) (DEAF1), mRNA [NM_021008]
0,50	0,00036	MLLT4	NM_001040001	Homo sapiens myeloid/lymphoid or mixed-lineage leukemia (trithorax homolog, Drosophila); translocated to, 4 (MLLT4), transcript variant 1, mRNA [NM_001040001]
0,50	0,00000	KLK6	NM_001012964	Homo sapiens kallikrein-related peptidase 6 (KLK6), transcript variant B, mRNA [NM_001012964]
0,50	0,00027	PHYHD1	NM_174933	Homo sapiens phytanoyl-CoA dioxygenase domain containing 1 (PHYHD1), mRNA [NM_174933]
0,50	0,00448	HOXB9	NM_024017	Homo sapiens homeobox B9 (HOXB9), mRNA [NM_024017]

0,50	0,00010	CYP7B1	NM_004820	Homo sapiens cytochrome P450, family 7, subfamily B, polypeptide 1 (CYP7B1), mRNA [NM_004820]
0,50	0,00016	LDLR	NM_000527	Homo sapiens low density lipoprotein receptor (familial hypercholesterolemia) (LDLR), mRNA [NM_000527]
0,50	0,00007	SOX3	NM_005634	Homo sapiens SRY (sex determining region Y)-box 3 (SOX3), mRNA [NM_005634]
0,50	0,00004	MTAC2D1	NM_152332	Homo sapiens membrane targeting (tandem) C2 domain containing 1 (MTAC2D1), mRNA [NM_152332]
0,50	0,00014	NDRG2	NM_201535	Homo sapiens NDRG family member 2 (NDRG2), transcript variant 1, mRNA [NM_201535]

Supplementary Table S7. Microarray induced genes in Non-Epithelioid vs. Epithelioid.

Ratio NoEpi vs Epi	NoEpi vs Epi FDR	Gene Symbol	Systematic Name	Description
7,32	0,00081	KRT34	NM_021013	Homo sapiens keratin 34 (KRT34), mRNA [NM_021013]
6,35	0,00037	CDH13	NM_001257	Homo sapiens cadherin 13, H-cadherin (heart) (CDH13), mRNA [NM_001257]
6,09	0,00039	COL6A3	NM_004369	Homo sapiens collagen, type VI, alpha 3 (COL6A3), transcript variant 1, mRNA [NM_004369]
6,02	0,00038	ACTG2	NM_001615	Homo sapiens actin, gamma 2, smooth muscle, enteric (ACTG2), mRNA [NM_001615]
5,91	0,00005	TNFSF4	NM_003326	Homo sapiens tumor necrosis factor (ligand) superfamily, member 4 (tax-transcriptionally activated glycoprotein 1, 34kDa) (TNFSF4), mRNA [NM_003326]
5,88	0,00201	THBS1	NM_003246	Homo sapiens thrombospondin 1 (THBS1), mRNA [NM_003246]
5,86	0,00120	COL13A1	NM_005203	Homo sapiens collagen, type XIII, alpha 1 (COL13A1), transcript variant 1, mRNA [NM_005203]
5,85	0,00038	S100A4	NM_002961	Homo sapiens S100 calcium binding protein A4 (S100A4), transcript variant 1, mRNA [NM_002961]
5,66	0,00004	MTHFD2	NM_006636	Homo sapiens methylenetetrahydrofolate dehydrogenase (NADP+ dependent) 2, methenyltetrahydrofolate cyclohydrolase (MTHFD2), nuclear gene encoding mitochondrial protein, transcript variant 1, mRNA [NM_006636]
5,54	0,00357	FST	NM_013409	Homo sapiens follistatin (FST), transcript variant FST344, mRNA [NM_013409]
5,28	0,00008	PSAT1	NM_058179	Homo sapiens phosphoserine aminotransferase 1 (PSAT1), transcript variant 1, mRNA [NM_058179]
5,07	0,00919	TNC	NM_002160	Homo sapiens tenascin C (hexabrachion) (TNC), mRNA [NM_002160]
4,58	0,00541	CTHRC1	NM_138455	Homo sapiens collagen triple helix repeat containing 1 (CTHRC1), mRNA [NM_138455]
4,56	0,00005	ARHGAP22	NM_021226	Homo sapiens Rho GTPase activating protein 22 (ARHGAP22), mRNA [NM_021226]
4,44	0,00300	TIMP3	NM_000362	Homo sapiens TIMP metallopeptidase inhibitor 3 (Sorsby fundus dystrophy, pseudoinflammatory) (TIMP3), mRNA [NM_000362]
4,31	0,00483	SPP1	NM_000582	Homo sapiens secreted phosphoprotein 1 (osteopontin, bone sialoprotein I, early T-lymphocyte activation 1) (SPP1), transcript variant 2, mRNA [NM_000582]

4,30	0,00093	CAV1	NM_001753	Homo sapiens caveolin 1, caveolae protein, 22kDa (CAV1), mRNA [NM_001753]
4,23	0,00213	ZNF652	NM_014897	Homo sapiens zinc finger protein 652 (ZNF652), mRNA [NM_014897]
4,22	0,00078	SULF1	NM_015170	Homo sapiens sulfatase 1 (SULF1), mRNA [NM_015170]
4,21	0,00095	LTBP1	NM_206943	Homo sapiens latent transforming growth factor beta binding protein 1 (LTBP1), transcript variant 1, mRNA [NM_206943]
4,20	0,00102	TNFAIP6	NM_007115	Homo sapiens tumor necrosis factor, alpha-induced protein 6 (TNFAIP6), mRNA [NM_007115]
4,12	0,00339	NEFM	NM_005382	Homo sapiens neurofilament, medium polypeptide 150kDa (NEFM), mRNA [NM_005382]
4,03	0,00283	HMGA2	NM_003483	Homo sapiens high mobility group AT-hook 2 (HMGA2), transcript variant 1, mRNA [NM_003483]
3,80	0,00271	EDIL3	NM_005711	Homo sapiens EGF-like repeats and discoidin I-like domains 3 (EDIL3), mRNA [NM_005711]
3,73	0,00081	GPC6	ENST00000377047	Glypican-6 precursor. [Source:Uniprot/SWISSPROT;Acc:Q9Y625] [ENST00000377047]
3,67	0,00059	TEK	NM_000459	Homo sapiens TEK tyrosine kinase, endothelial (venous malformations, multiple cutaneous and mucosal) (TEK), mRNA [NM_000459]
3,60	0,00004	CCL26	NM_006072	Homo sapiens chemokine (C-C motif) ligand 26 (CCL26), mRNA [NM_006072]
3,56	0,00037	SYNPO	ENST00000307662	Synaptopodin. [Source:Uniprot/SWISSPROT;Acc:Q8N3V7] [ENST00000307662]
3,52	0,00006	POPDC3	NM_022361	Homo sapiens popeye domain containing 3 (POPDC3), mRNA [NM_022361]
3,50	0,00144	ITGBL1	NM_004791	Homo sapiens integrin, beta-like 1 (with EGF-like repeat domains) (ITGBL1), mRNA [NM_004791]
3,40	0,00646	FHOD3	NM_025135	Homo sapiens formin homology 2 domain containing 3 (FHOD3), mRNA [NM_025135]
3,37	0,00725	SLC2A1	NM_006516	Homo sapiens solute carrier family 2 (facilitated glucose transporter), member 1 (SLC2A1), mRNA [NM_006516]
3,37	0,00033	SRPX2	NM_014467	Homo sapiens sushi-repeat-containing protein, X-linked 2 (SRPX2), mRNA [NM_014467]
3,37	0,00185	MST150	NM_032947	Homo sapiens MSTP150 (MST150), mRNA [NM_032947]
3,35	0,00940	THBS2	L12350	Human thrombospondin 2 (THBS2) mRNA, complete cds. [L12350]
3,26	0,00005	MLPH	NM_024101	Homo sapiens melanophilin (MLPH), transcript variant 1, mRNA [NM_024101]

3,26	0,00234	TGFB2	ENST00000366930	Transforming growth factor beta-2 precursor (TGF-beta-2) (Glioblastoma-derived T-cell suppressor factor) (G-TSF) (BSC-1 cell growth inhibitor) (Polyergin) (Cetermin). [Source:Uniprot/SWISSPROT;Acc:P61812] [ENST00000366930]
3,22	0,00012	OSBPL6	NM_032523	Homo sapiens oxysterol binding protein-like 6 (OSBPL6), transcript variant 1, mRNA [NM_032523]
3,22	0,00034	ELTD1	BC025721	Homo sapiens EGF, latrophilin and seven transmembrane domain containing 1, mRNA (cDNA clone MGC:34204 IMAGE:5229055), complete cds. [BC025721]
3,18	0,00071	ADAMTS4	NM_005099	Homo sapiens ADAM metallopeptidase with thrombospondin type 1 motif, 4 (ADAMTS4), mRNA [NM_005099]
3,16	0,00903	CD44	NM_000610	Homo sapiens CD44 molecule (Indian blood group) (CD44), transcript variant 1, mRNA [NM_000610]
3,14	0,00450	PITX2	NM_153426	Homo sapiens paired-like homeodomain transcription factor 2 (PITX2), transcript variant 2, mRNA [NM_153426]
3,11	0,00341	HNT	NM_016522	Homo sapiens neurotrimin (HNT), transcript variant 1, mRNA [NM_016522]
3,09	0,00009	KCNN4	NM_002250	Homo sapiens potassium intermediate/small conductance calcium-activated channel, subfamily N, member 4 (KCNN4), mRNA [NM_002250]
3,07	0,00960	GPAM	NM_020918	Homo sapiens glycerol-3-phosphate acyltransferase, mitochondrial (GPAM), mRNA [NM_020918]
3,06	0,00079	ARHGDIB	NM_001175	Homo sapiens Rho GDP dissociation inhibitor (GDI) beta (ARHGDIB), mRNA [NM_001175]
3,03	0,00234	PPP1R3C	NM_005398	Homo sapiens protein phosphatase 1, regulatory (inhibitor) subunit 3C (PPP1R3C), mRNA [NM_005398]
3,00	0,00362	TGFBI	NM_000358	Homo sapiens transforming growth factor, beta-induced, 68kDa (TGFBI), mRNA [NM_000358]
2,98	0,00398	CCND1	NM_053056	Homo sapiens cyclin D1 (CCND1), mRNA [NM_053056]
2,95	0,00756	MGC23985	NM_206966	Homo sapiens similar to AVLV472 (MGC23985), mRNA [NM_206966]
2,92	0,00091	RGMB	NM_001012761	Homo sapiens RGM domain family, member B (RGMB), transcript variant 1, mRNA [NM_001012761]
2,91	0,00265	VIM	NM_003380	Homo sapiens vimentin (VIM), mRNA [NM_003380]
2,88	0,00839	G0S2	NM_015714	Homo sapiens G0/G1switch 2 (G0S2), mRNA [NM_015714]
2,85	0,00102	ENST00000376155	ENST00000376155	integrin, beta-like 1 (with EGF-like repeat domains) [Source:RefSeq_peptide;Acc:NP_004782] [ENST00000376155]
2,85	0,00387	XG	NM_175569	Homo sapiens Xg blood group (XG), mRNA [NM_175569]
2,78	0,00169	DOCK10	NM_014689	Homo sapiens dedicator of cytokinesis 10 (DOCK10), mRNA [NM_014689]

2,77	0,00297	UCHL1	NM_004181	Homo sapiens ubiquitin carboxyl-terminal esterase L1 (ubiquitin thiolesterase) (UCHL1), mRNA [NM_004181]
2,74	0,00043	ARNTL2	AF256215	Homo sapiens cycle-like factor CLIF mRNA, complete cds. [AF256215]
2,71	0,00010	DLEU1	NR_002605	Homo sapiens deleted in lymphocytic leukemia, 1 (DLEU1) on chromosome 13 [NR_002605]
2,69	0,00078	TIMP1	NM_003254	Homo sapiens TIMP metallopeptidase inhibitor 1 (TIMP1), mRNA [NM_003254]
2,68	0,00444	ATM	NM_000051	Homo sapiens ataxia telangiectasia mutated (includes complementation groups A, C and D) (ATM), transcript variant 1, mRNA [NM_000051]
2,67	0,00089	RCN3	NM_020650	Homo sapiens reticulocalbin 3, EF-hand calcium binding domain (RCN3), mRNA [NM_020650]
2,67	0,00787	E2F7	NM_203394	Homo sapiens E2F transcription factor 7 (E2F7), mRNA [NM_203394]
2,66	0,00139	GNG11	NM_004126	Homo sapiens guanine nucleotide binding protein (G protein), gamma 11 (GNG11), mRNA [NM_004126]
2,65	0,00590	EMX2OS	NR_002791	Homo sapiens empty spiracles homeobox 2 opposite strand (EMX2OS) on chromosome 10 [NR_002791]
2,65	0,00022	CAV3	NM_001234	Homo sapiens caveolin 3 (CAV3), transcript variant 2, mRNA [NM_001234]
2,64	0,00040	WNT5B	NM_030775	Homo sapiens wingless-type MMTV integration site family, member 5B (WNT5B), transcript variant 2, mRNA [NM_030775]
2,63	0,00505	EMP1	NM_001423	Homo sapiens epithelial membrane protein 1 (EMP1), mRNA [NM_001423]
2,63	0,00430	LPXN	NM_004811	Homo sapiens leupaxin (LPXN), mRNA [NM_004811]
2,61	0,00004	RHOJ	NM_020663	Homo sapiens ras homolog gene family, member J (RHOJ), mRNA [NM_020663]
2,58	0,00572	SNAI1	NM_005985	Homo sapiens snail homolog 1 (Drosophila) (SNAI1), mRNA [NM_005985]
2,51	0,00085	PAX8	NM_003466	Homo sapiens paired box gene 8 (PAX8), transcript variant PAX8A, mRNA [NM_003466]
2,50	0,00079	LZTS1	NM_021020	Homo sapiens leucine zipper, putative tumor suppressor 1 (LZTS1), mRNA [NM_021020]
2,50	0,00481	PHLDA2	NM_003311	Homo sapiens pleckstrin homology-like domain, family A, member 2 (PHLDA2), mRNA [NM_003311]
2,47	0,00160	TMEM132B	NM_052907	Homo sapiens transmembrane protein 132B (TMEM132B), mRNA [NM_052907]
2,47	0,00765	SMYD3	NM_022743	Homo sapiens SET and MYND domain containing 3 (SMYD3), mRNA [NM_022743]

2,45	0,00218	PRSS23	NM_007173	Homo sapiens protease, serine, 23 (PRSS23), mRNA [NM_007173]
2,43	0,00072	NRXN3	NM_004796	Homo sapiens neurexin 3 (NRXN3), transcript variant alpha, mRNA [NM_004796]
2,43	0,00188	LOC401152	NM_001001701	Homo sapiens HCV F-transactivated protein 1 (LOC401152), mRNA [NM_001001701]
2,42	0,00259	TREM1	NM_018643	Homo sapiens triggering receptor expressed on myeloid cells 1 (TREM1), mRNA [NM_018643]
2,41	0,00435	PRNP	NM_000311	Homo sapiens prion protein (p27-30) (Creutzfeldt-Jakob disease, Gerstmann-Strausler-Scheinker syndrome, fatal familial insomnia) (PRNP), transcript variant 1, mRNA [NM_000311]
2,40	0,00104	SGCD	NM_000337	Homo sapiens sarcoglycan, delta (35kDa dystrophin-associated glycoprotein) (SGCD), transcript variant 1, mRNA [NM_000337]
2,40	0,00004	SSR3	NM_007107	Homo sapiens signal sequence receptor, gamma (translocon-associated protein gamma) (SSR3), mRNA [NM_007107]
2,39	0,00435	TSLP	NM_033035	Homo sapiens thymic stromal lymphopoietin (TSLP), transcript variant 1, mRNA [NM_033035]
2,38	0,00011	ADAMTS6	NM_197941	Homo sapiens ADAM metallopeptidase with thrombospondin type 1 motif, 6 (ADAMTS6), mRNA [NM_197941]
2,35	0,00038	SLC7A11	NM_014331	Homo sapiens solute carrier family 7, (cationic amino acid transporter, y+ system) member 11 (SLC7A11), mRNA [NM_014331]
2,33	0,00120	NTF3	NM_002527	Homo sapiens neurotrophin 3 (NTF3), mRNA [NM_002527]
2,33	0,00039	HRH1	NM_000861	Homo sapiens histamine receptor H1 (HRH1), mRNA [NM_000861]
2,27	0,00017	ADAMTSL1	NM_052866	Homo sapiens ADAMTS-like 1 (ADAMTSL1), transcript variant 2, mRNA [NM_052866]
2,25	0,00183	PDGFA	NM_002607	Homo sapiens platelet-derived growth factor alpha polypeptide (PDGFA), transcript variant 1, mRNA [NM_002607]
2,23	0,00301	ABTB2	NM_145804	Homo sapiens ankyrin repeat and BTB (POZ) domain containing 2 (ABTB2), mRNA [NM_145804]
2,22	0,00017	CHIC2	NM_012110	Homo sapiens cysteine-rich hydrophobic domain 2 (CHIC2), mRNA [NM_012110]
2,22	0,00048	ADCY7	NM_001114	Homo sapiens adenylate cyclase 7 (ADCY7), mRNA [NM_001114]
2,22	0,00582	P2RY6	NM_176798	Homo sapiens pyrimidinergic receptor P2Y, G-protein coupled, 6 (P2RY6), transcript variant 2, mRNA [NM_176798]
2,20	0,00995	PKIA	NM_006823	Homo sapiens protein kinase (cAMP-dependent, catalytic) inhibitor alpha (PKIA), transcript variant 6, mRNA [NM_006823]
2,20	0,00175	MYB	NM_005375	Homo sapiens v-myb myeloblastosis viral oncogene homolog (avian) (MYB), mRNA [NM_005375]
2,19	0,00263	FBN1	NM_000138	Homo sapiens fibrillin 1 (FBN1), mRNA [NM_000138]

2,16	0,00568	ENST00000372871	ENST00000372871	Ribosomal protein L7-like 1. [Source:Uniprot/SWISSPROT;Acc:Q6DKI1] [ENST00000372871]
2,15	0,00166	SEC61G	NM_014302	Homo sapiens Sec61 gamma subunit (SEC61G), transcript variant 1, mRNA [NM_014302]
2,15	0,00047	ENG	NM_000118	Homo sapiens endoglin (Osler-Rendu-Weber syndrome 1) (ENG), mRNA [NM_000118]
2,15	0,00243	AKR1C3	NM_003739	Homo sapiens aldo-keto reductase family 1, member C3 (3-alpha hydroxysteroid dehydrogenase, type II) (AKR1C3), mRNA [NM_003739]
2,14	0,00063	PYCR1	NM_006907	Homo sapiens pyrroline-5-carboxylate reductase 1 (PYCR1), transcript variant 1, mRNA [NM_006907]
2,14	0,00079	KLF12	NM_007249	Homo sapiens Kruppel-like factor 12 (KLF12), mRNA [NM_007249]
2,13	0,00277	NRP1	NM_003873	Homo sapiens neuropilin 1 (NRP1), transcript variant 1, mRNA [NM_003873]
2,13	0,00584	COL6A2	NM_001849	Homo sapiens collagen, type VI, alpha 2 (COL6A2), transcript variant 2C2, mRNA [NM_001849]
2,12	0,00278	ITGB1	NM_002211	Homo sapiens integrin, beta 1 (fibronectin receptor, beta polypeptide, antigen CD29 includes MDF2, MSK12) (ITGB1), transcript variant 1A, mRNA [NM_002211]
2,12	0,00244	PFKP	NM_002627	Homo sapiens phosphofructokinase, platelet (PFKP), mRNA [NM_002627]
2,11	0,00140	ASNS	NM_001673	Homo sapiens asparagine synthetase (ASNS), transcript variant 2, mRNA [NM_001673]
2,11	0,00741	ZNF264	NM_003417	Homo sapiens zinc finger protein 264 (ZNF264), mRNA [NM_003417]
2,11	0,00017	TNS1	NM_022648	Homo sapiens tensin 1 (TNS1), mRNA [NM_022648]
2,10	0,00166	FNDC3B	BC012204	Homo sapiens fibronectin type III domain containing 3B, mRNA (cDNA clone IMAGE:3882800), complete cds. [BC012204]
2,10	0,00026	TMEM55A	NM_018710	Homo sapiens transmembrane protein 55A (TMEM55A), mRNA [NM_018710]
2,10	0,00137	IGF2	NM_001007139	Homo sapiens insulin-like growth factor 2 (somatomedin A) (IGF2), transcript variant 2, mRNA [NM_001007139]
2,10	0,00005	ANGPT1	NM_001146	Homo sapiens angiopoietin 1 (ANGPT1), mRNA [NM_001146]
2,09	0,00038	DGKI	NM_004717	Homo sapiens diacylglycerol kinase, iota (DGKI), mRNA [NM_004717]
2,08	0,00038	RPL27	NM_000988	Homo sapiens ribosomal protein L27 (RPL27), mRNA [NM_000988]
2,08	0,00465	FAM46A	NM_017633	Homo sapiens family with sequence similarity 46, member A (FAM46A), mRNA [NM_017633]

2,06	0,00123	NR3C1	NM_000176	Homo sapiens nuclear receptor subfamily 3, group C, member 1 (glucocorticoid receptor) (NR3C1), transcript variant 5, mRNA [NM_000176]
2,06	0,00160	ECM2	NM_001393	Homo sapiens extracellular matrix protein 2, female organ and adipocyte specific (ECM2), mRNA [NM_001393]
2,05	0,00139	S100A10	NM_002966	Homo sapiens S100 calcium binding protein A10 (S100A10), mRNA [NM_002966]
2,05	0,00188	CALU	NM_001219	Homo sapiens calumenin (CALU), mRNA [NM_001219]
2,05	0,00168	CD274	ENST00000381577	Programmed cell death 1 ligand 1 precursor (Programmed death ligand 1) (PD-L1) (PDCD1 ligand 1) (B7 homolog 1) (B7-H1) (CD274 antigen). [Source:Uniprot/SWISSPROT;Acc:Q9NZQ7] [ENST00000381577]
2,05	0,00511	ZNF429	NM_001001415	Homo sapiens zinc finger protein 429 (ZNF429), mRNA [NM_001001415]
2,04	0,00570	GPR68	NM_003485	Homo sapiens G protein-coupled receptor 68 (GPR68), mRNA [NM_003485]
2,03	0,00217	TNIP3	NM_024873	Homo sapiens TNFAIP3 interacting protein 3 (TNIP3), mRNA [NM_024873]
2,02	0,00041	CDC42EP3	NM_006449	Homo sapiens CDC42 effector protein (Rho GTPase binding) 3 (CDC42EP3), mRNA [NM_006449]
2,02	0,00056	CHN1	NM_001822	Homo sapiens chimerin (chimaerin) 1 (CHN1), transcript variant 1, mRNA [NM_001822]
2,02	0,00276	STK17A	NM_004760	Homo sapiens serine/threonine kinase 17a (STK17A), mRNA [NM_004760]
2,01	0,00177	ARL6IP6	NM_152522	Homo sapiens ADP-ribosylation-like factor 6 interacting protein 6 (ARL6IP6), mRNA [NM_152522]
2,00	0,00995	GRB14	NM_004490	Homo sapiens growth factor receptor-bound protein 14 (GRB14), mRNA [NM_004490]

Supplementary Table S8. Microarray repressed genes in Non-Epithelioid vs. Epithelioid.

Ratio NoEpi vs Epi	NoEpi vs Epi FDR	Gene Symbol	Systematic Name	Description
0,07	0,00088	SEPP1	NM_005410	Homo sapiens selenoprotein P, plasma, 1 (SEPP1), mRNA [NM_005410]
0,14	0,00012	PLA2G7	NM_005084	Homo sapiens phospholipase A2, group VII (platelet-activating factor acetylhydrolase, plasma) (PLA2G7), mRNA [NM_005084]
0,15	0,00029	OGDHL	NM_018245	Homo sapiens oxoglutarate dehydrogenase-like (OGDHL), mRNA [NM_018245]
0,15	0,00055	EGFL6	NM_015507	Homo sapiens EGF-like-domain, multiple 6 (EGFL6), mRNA [NM_015507]
0,17	0,00328	RPESP	NM_153225	Homo sapiens RPE-spondin (RPESP), mRNA [NM_153225]
0,18	0,00878	NMU	NM_006681	Homo sapiens neuromedin U (NMU), mRNA [NM_006681]
0,19	0,00354	SLC40A1	NM_014585	Homo sapiens solute carrier family 40 (iron-regulated transporter), member 1 (SLC40A1), mRNA [NM_014585]
0,20	0,00015	GLT25D2	NM_015101	Homo sapiens glycosyltransferase 25 domain containing 2 (GLT25D2), mRNA [NM_015101]
0,20	0,00259	NDRG4	NM_022910	Homo sapiens NDRG family member 4 (NDRG4), mRNA [NM_022910]
0,20	0,00412	THBD	NM_000361	Homo sapiens thrombomodulin (THBD), mRNA [NM_000361]
0,20	0,00022	ENPP1	NM_006208	Homo sapiens ectonucleotide pyrophosphatase/phosphodiesterase 1 (ENPP1), mRNA [NM_006208]
0,21	0,00338	HTRA4	NM_153692	Homo sapiens HtrA serine peptidase 4 (HTRA4), mRNA [NM_153692]
0,21	0,00026	SALL4	NM_020436	Homo sapiens sal-like 4 (Drosophila) (SALL4), mRNA [NM_020436]
0,22	0,00688	ADM	NM_001124	Homo sapiens adrenomedullin (ADM), mRNA [NM_001124]
0,22	0,00105	KLK11	NM_144947	Homo sapiens kallikrein-related peptidase 11 (KLK11), transcript variant 2, mRNA [NM_144947]
0,23	0,00442	CDH1	NM_004360	Homo sapiens cadherin 1, type 1, E-cadherin (epithelial) (CDH1), mRNA [NM_004360]
0,23	0,00016	ITGB2	NM_000211	Homo sapiens integrin, beta 2 (complement component 3 receptor 3 and 4 subunit) (ITGB2), mRNA [NM_000211]
0,23	0,00441	NAT8L	NM_178557	Homo sapiens N-acetyltransferase 8-like (NAT8L), mRNA [NM_178557]
0,23	0,00141	MAF	NM_005360	Homo sapiens v-maf musculoaponeurotic fibrosarcoma oncogene homolog (avian) (MAF), transcript variant 1, mRNA [NM_005360]
0,24	0,00020	CPVL	NM_031311	Homo sapiens carboxypeptidase, vitellogenic-like (CPVL), transcript variant 1, mRNA [NM_031311]
0,24	0,00435	ABLIM2	NM_032432	Homo sapiens actin binding LIM protein family, member 2 (ABLIM2), mRNA [NM_032432]
0,26	0,00396	GKN1	NM_019617	Homo sapiens gastrokine 1 (GKN1), mRNA [NM_019617]

0,26	0,00203	SLC7A7	NM_003982	Homo sapiens solute carrier family 7 (cationic amino acid transporter, y+ system), member 7 (SLC7A7), mRNA [NM_003982]
0,27	0,00238	CFB	NM_001710	Homo sapiens complement factor B (CFB), mRNA [NM_001710]
0,27	0,00382	GBP3	NM_018284	Homo sapiens guanylate binding protein 3 (GBP3), mRNA [NM_018284]
0,27	0,00144	KDR	NM_002253	Homo sapiens kinase insert domain receptor (a type III receptor tyrosine kinase) (KDR), mRNA [NM_002253]
0,28	0,00053	AIF1	NM_004847	Homo sapiens allograft inflammatory factor 1 (AIF1), transcript variant 2, mRNA [NM_004847]
0,28	0,00213	HABP4	NM_014282	Homo sapiens hyaluronan binding protein 4 (HABP4), mRNA [NM_014282]
0,29	0,00126	TESC	NM_017899	Homo sapiens tescalcin (TESC), mRNA [NM_017899]
0,29	0,00076	ALDH1A1	NM_000689	Homo sapiens aldehyde dehydrogenase 1 family, member A1 (ALDH1A1), mRNA [NM_000689]
0,30	0,00008	KCNQ1	NM_000218	Homo sapiens potassium voltage-gated channel, KQT-like subfamily, member 1 (KCNQ1), transcript variant 1, mRNA [NM_000218]
0,30	0,00027	COL8A1	NM_001850	Homo sapiens collagen, type VIII, alpha 1 (COL8A1), transcript variant 1, mRNA [NM_001850]
0,30	0,00989	ID4	NM_001546	Homo sapiens inhibitor of DNA binding 4, dominant negative helix-loop-helix protein (ID4), mRNA [NM_001546]
0,30	0,00031	RAB11FIP1	NM_001002233	Homo sapiens RAB11 family interacting protein 1 (class I) (RAB11FIP1), transcript variant 2, mRNA [NM_001002233]
0,30	0,00160	AY090769	AY090769	Homo sapiens ribosomal protein S18/S6-like mRNA, complete sequence. [AY090769]
0,31	0,00202	TMEM37	NM_183240	Homo sapiens transmembrane protein 37 (TMEM37), mRNA [NM_183240]
0,31	0,00023	ENPP2	NM_006209	Homo sapiens ectonucleotide pyrophosphatase/phosphodiesterase 2 (autotaxin) (ENPP2), transcript variant 1, mRNA [NM_006209]
0,31	0,00446	DMKN	NM_033317	Homo sapiens dermokine (DMKN), transcript variant 2, mRNA [NM_033317]
0,31	0,00060	SEMA5A	NM_003966	Homo sapiens sema domain, seven thrombospondin repeats (type 1 and type 1-like), transmembrane domain (TM) and short cytoplasmic domain, (semaphorin) 5A (SEMA5A), mRNA [NM_003966]
0,31	0,00037	TCEAL2	NM_080390	Homo sapiens transcription elongation factor A (SII)-like 2 (TCEAL2), mRNA [NM_080390]
0,32	0,00323	SPINT2	NM_021102	Homo sapiens serine peptidase inhibitor, Kunitz type, 2 (SPINT2), mRNA [NM_021102]
0,32	0,00037	UPK3B	NM_030570	Homo sapiens uroplakin 3B (UPK3B), transcript variant 1, mRNA [NM_030570]
0,32	0,00525	MAF	AF055376	Homo sapiens short form transcription factor C-MAF (c-maf) mRNA, complete cds. [AF055376]
0,32	0,00103	MAOB	NM_000898	Homo sapiens monoamine oxidase B (MAOB), nuclear gene encoding mitochondrial protein, mRNA [NM_000898]

0,32	0,00017	GNPDA1	NM_005471	Homo sapiens glucosamine-6-phosphate deaminase 1 (GNPDA1), mRNA [NM_005471]
0,32	0,00713	MARCO	NM_006770	Homo sapiens macrophage receptor with collagenous structure (MARCO), mRNA [NM_006770]
0,33	0,00015	ETS2	NM_005239	Homo sapiens v-ets erythroblastosis virus E26 oncogene homolog 2 (avian) (ETS2), mRNA [NM_005239]
0,33	0,00005	ITIH5	NM_030569	Homo sapiens inter-alpha (globulin) inhibitor H5 (ITIH5), transcript variant 1, mRNA [NM_030569]
0,33	0,00023	ENPP5	NM_021572	Homo sapiens ectonucleotide pyrophosphatase/phosphodiesterase 5 (putative function) (ENPP5), mRNA [NM_021572]
0,33	0,00191	IGJ	NM_144646	Homo sapiens immunoglobulin J polypeptide, linker protein for immunoglobulin alpha and mu polypeptides (IGJ), mRNA [NM_144646]
0,33	0,00131	SEMA5A	ENST00000382496	Semaphorin-5A precursor (Semaphorin F) (Sema F). [Source:Uniprot/SWISSPROT;Acc:Q13591] [ENST00000382496]
0,34	0,00070	FRAS1	NM_025074	Homo sapiens Fraser syndrome 1 (FRAS1), mRNA [NM_025074]
0,34	0,00158	ZNF331	NM_018555	Homo sapiens zinc finger protein 331 (ZNF331), transcript variant 1, mRNA [NM_018555]
0,34	0,00160	NPR1	NM_000906	Homo sapiens natriuretic peptide receptor A/guanylate cyclase A (atrionatriuretic peptide receptor A) (NPR1), mRNA [NM_000906]
0,34	0,00748	HP	NM_005143	Homo sapiens haptoglobin (HP), mRNA [NM_005143]
0,34	0,00041	MX1	NM_002462	Homo sapiens myxovirus (influenza virus) resistance 1, interferon-inducible protein p78 (mouse) (MX1), mRNA [NM_002462]
0,34	0,00118	KLK10	NM_002776	Homo sapiens kallikrein-related peptidase 10 (KLK10), transcript variant 1, mRNA [NM_002776]
0,34	0,00328	CRB2	NM_173689	Homo sapiens crumbs homolog 2 (Drosophila) (CRB2), mRNA [NM_173689]
0,34	0,00324	KCNB1	NM_004975	Homo sapiens potassium voltage-gated channel, Shab-related subfamily, member 1 (KCNB1), mRNA [NM_004975]
0,34	0,00017	SLC39A1	NM_014437	Homo sapiens solute carrier family 39 (zinc transporter), member 1 (SLC39A1), mRNA [NM_014437]
0,35	0,00026	EFNA1	NM_004428	Homo sapiens ephrin-A1 (EFNA1), transcript variant 1, mRNA [NM_004428]
0,35	0,00665	NOX1	NM_013955	Homo sapiens NADPH oxidase 1 (NOX1), transcript variant NOH-1Lv, mRNA [NM_013955]
0,35	0,00005	KCNK1	NM_002245	Homo sapiens potassium channel, subfamily K, member 1 (KCNK1), mRNA [NM_002245]
0,36	0,00964	HHIP	NM_022475	Homo sapiens hedgehog interacting protein (HHIP), mRNA [NM_022475]
0,37	0,00117	MAP3K8	NM_005204	Homo sapiens mitogen-activated protein kinase kinase kinase 8 (MAP3K8), mRNA [NM_005204]
0,37	0,00026	SEMA3E	NM_012431	Homo sapiens sema domain, immunoglobulin domain (Ig), short basic domain, secreted, (semaphorin) 3E (SEMA3E), mRNA [NM_012431]

0,37	0,00168	CFH	NM_000186	Homo sapiens complement factor H (CFH), transcript variant 1, mRNA [NM_000186]
0,37	0,00095	SMPD3	NM_018667	Homo sapiens sphingomyelin phosphodiesterase 3, neutral membrane (neutral sphingomyelinase II) (SMPD3), mRNA [NM_018667]
0,37	0,00741	ANXA8	BC008813	Homo sapiens annexin A8, mRNA (cDNA clone MGC:10405 IMAGE:3958020), complete cds. [BC008813]
0,37	0,00381	RC3H1	NM_172071	Homo sapiens ring finger and CCCH-type zinc finger domains 1 (RC3H1), mRNA [NM_172071]
0,38	0,00086	STOX2	NM_020225	Homo sapiens storkhead box 2 (STOX2), mRNA [NM_020225]
0,38	0,00075	C2	NM_000063	Homo sapiens complement component 2 (C2), mRNA [NM_000063]
0,38	0,00357	BEX2	NM_032621	Homo sapiens brain expressed X-linked 2 (BEX2), mRNA [NM_032621]
0,38	0,00047	ENPP1	ENST00000367994	Ectonucleotide pyrophosphatase/phosphodiesterase 1 (E-NPP 1) (Phosphodiesterase I/nucleotide pyrophosphatase 1) (Plasma-cell membrane glycoprotein PC-1) [Includes: Alkaline phosphodiesterase I (EC 3.1.4.1); Nucleotide pyrophosphatase (EC 3.6.1.9)...
0,38	0,00125	KCNH2	NM_000238	Homo sapiens potassium voltage-gated channel, subfamily H (eag-related), member 2 (KCNH2), transcript variant 1, mRNA [NM_000238]
0,38	0,00621	FGF18	NM_003862	Homo sapiens fibroblast growth factor 18 (FGF18), mRNA [NM_003862]
0,38	0,00009	NDRG2	NM_201535	Homo sapiens NDRG family member 2 (NDRG2), transcript variant 1, mRNA [NM_201535]
0,38	0,00031	SASH1	NM_015278	Homo sapiens SAM and SH3 domain containing 1 (SASH1), mRNA [NM_015278]
0,38	0,00023	LRP3	NM_002333	Homo sapiens low density lipoprotein receptor-related protein 3 (LRP3), mRNA [NM_002333]
0,39	0,00872	SNX10	NM_013322	Homo sapiens sorting nexin 10 (SNX10), mRNA [NM_013322]
0,39	0,00104	PPP1R9A	NM_017650	Homo sapiens protein phosphatase 1, regulatory (inhibitor) subunit 9A (PPP1R9A), mRNA [NM_017650]
0,39	0,00724	ITIH5	ENST00000256861	inter-alpha trypsin inhibitor heavy chain precursor 5 isoform 1 [Source:RefSeq_peptide;Acc:NP_085046] [ENST00000256861]
0,39	0,00634	RAMP1	NM_005855	Homo sapiens receptor (G protein-coupled) activity modifying protein 1 (RAMP1), mRNA [NM_005855]
0,39	0,00404	SRCRB4D	NM_080744	Homo sapiens scavenger receptor cysteine rich domain containing, group B (4 domains) (SRCRB4D), mRNA [NM_080744]
0,39	0,00855	WWC1	NM_015238	Homo sapiens WW and C2 domain containing 1 (WWC1), mRNA [NM_015238]
0,39	0,00048	GPRC5C	NM_022036	Homo sapiens G protein-coupled receptor, family C, group 5, member C (GPRC5C), transcript variant 1, mRNA [NM_022036]
0,39	0,00055	NTRK2	NM_001007097	Homo sapiens neurotrophic tyrosine kinase, receptor, type 2 (NTRK2), transcript variant b, mRNA [NM_001007097]

0,39	0,00044	ZNF330	NM_014487	Homo sapiens zinc finger protein 330 (ZNF330), mRNA [NM_014487]
0,39	0,00616	CD244	NM_016382	Homo sapiens CD244 molecule, natural killer cell receptor 2B4 (CD244), mRNA [NM_016382]
0,39	0,00029	CXADR	NM_001338	Homo sapiens coxsackie virus and adenovirus receptor (CXADR), mRNA [NM_001338]
0,39	0,00036	SHC2	NM_012435	Homo sapiens SHC (Src homology 2 domain containing) transforming protein 2 (SHC2), mRNA [NM_012435]
0,40	0,00216	TJP2	NM_004817	Homo sapiens tight junction protein 2 (zona occludens 2) (TJP2), transcript variant 1, mRNA [NM_004817]
0,40	0,00111	FBP1	NM_000507	Homo sapiens fructose-1,6-bisphosphatase 1 (FBP1), mRNA [NM_000507]
0,40	0,00812	KIAA0746	NM_015187	Homo sapiens KIAA0746 protein (KIAA0746), mRNA [NM_015187]
0,40	0,00282	VIPR2	NM_003382	Homo sapiens vasoactive intestinal peptide receptor 2 (VIPR2), mRNA [NM_003382]
0,40	0,00833	BEX1	NM_018476	Homo sapiens brain expressed, X-linked 1 (BEX1), mRNA [NM_018476]
0,40	0,00446	DKFZP564O0823	NM_015393	Homo sapiens DKFZP564O0823 protein (DKFZP564O0823), mRNA [NM_015393]
0,40	0,00452	PLEKHG3	NM_015549	Homo sapiens pleckstrin homology domain containing, family G (with RhoGef domain) member 3 (PLEKHG3), mRNA [NM_015549]
0,41	0,00422	MUC16	NM_024690	Homo sapiens mucin 16, cell surface associated (MUC16), mRNA [NM_024690]
0,41	0,00056	ABHD4	NM_022060	Homo sapiens abhydrolase domain containing 4 (ABHD4), mRNA [NM_022060]
0,41	0,00031	A2BP1	NM_018723	Homo sapiens ataxin 2-binding protein 1 (A2BP1), transcript variant 4, mRNA [NM_018723]
0,41	0,00037	RHBDL1	NM_003961	Homo sapiens rhomboid, veinlet-like 1 (Drosophila) (RHBDL1), mRNA [NM_003961]
0,41	0,00086	BAZ2A	NM_013449	Homo sapiens bromodomain adjacent to zinc finger domain, 2A (BAZ2A), mRNA [NM_013449]
0,41	0,00254	DSCR1L1	NM_005822	Homo sapiens Down syndrome critical region gene 1-like 1 (DSCR1L1), mRNA [NM_005822]
0,41	0,00145	CSRP2	NM_001321	Homo sapiens cysteine and glycine-rich protein 2 (CSRP2), mRNA [NM_001321]
0,41	0,00140	XRN2	NM_012255	Homo sapiens 5'-3' exoribonuclease 2 (XRN2), mRNA [NM_012255]
0,41	0,00055	CAND2	NM_012298	Homo sapiens cullin-associated and neddylation-dissociated 2 (putative) (CAND2), mRNA [NM_012298]
0,41	0,00035	NMNAT3	NM_178177	Homo sapiens nicotinamide nucleotide adenylyltransferase 3 (NMNAT3), mRNA [NM_178177]
0,41	0,00369	CGN	NM_020770	Homo sapiens cingulin (CGN), mRNA [NM_020770]

0,41	0,00242	ARHGEF16	NM_014448	Homo sapiens Rho guanine exchange factor (GEF) 16 (ARHGEF16), mRNA [NM_014448]
0,41	0,00393	ADFP	NM_001122	Homo sapiens adipose differentiation-related protein (ADFP), mRNA [NM_001122]
0,41	0,00624	TXNRD1	NM_003330	Homo sapiens thioredoxin reductase 1 (TXNRD1), transcript variant 1, mRNA [NM_003330]
0,41	0,00136	CRISPLD1	NM_031461	Homo sapiens cysteine-rich secretory protein LCCL domain containing 1 (CRISPLD1), mRNA [NM_031461]
0,41	0,00067	CCDC125	NM_176816	Homo sapiens coiled-coil domain containing 125 (CCDC125), mRNA [NM_176816]
0,41	0,00146	EEF2	NM_001961	Homo sapiens eukaryotic translation elongation factor 2 (EEF2), mRNA [NM_001961]
0,42	0,00848	ABCG1	NM_207630	Homo sapiens ATP-binding cassette, sub-family G (WHITE), member 1 (ABCG1), transcript variant 1, mRNA [NM_207630]
0,42	0,00040	FAM110B	NM_147189	Homo sapiens family with sequence similarity 110, member B (FAM110B), mRNA [NM_147189]
0,42	0,00129	SYNPO	NM_007286	Homo sapiens synaptopodin (SYNPO), mRNA [NM_007286]
0,42	0,00017	LZTS2	NM_032429	Homo sapiens leucine zipper, putative tumor suppressor 2 (LZTS2), mRNA [NM_032429]
0,42	0,00020	STON2	NM_033104	Homo sapiens stonin 2 (STON2), mRNA [NM_033104]
0,42	0,00017	SPRY1	NM_199327	Homo sapiens sprouty homolog 1, antagonist of FGF signaling (Drosophila) (SPRY1), transcript variant 2, mRNA [NM_199327]
0,43	0,00230	FOXQ1	NM_033260	Homo sapiens forkhead box Q1 (FOXQ1), mRNA [NM_033260]
0,43	0,00183	MYLIP	NM_013262	Homo sapiens myosin regulatory light chain interacting protein (MYLIP), mRNA [NM_013262]
0,43	0,00096	CTAGE4	AF338232	Homo sapiens CTAGE-4 protein mRNA, complete cds. [AF338232]
0,43	0,00269	DAAM2	NM_015345	Homo sapiens dishevelled associated activator of morphogenesis 2 (DAAM2), mRNA [NM_015345]
0,43	0,00008	DFNA5	NM_004403	Homo sapiens deafness, autosomal dominant 5 (DFNA5), mRNA [NM_004403]
0,43	0,00058	MAN1C1	NM_020379	Homo sapiens mannosidase, alpha, class 1C, member 1 (MAN1C1), mRNA [NM_020379]
0,43	0,00046	OPLAH	NM_017570	Homo sapiens 5-oxoprolinase (ATP-hydrolysing) (OPLAH), mRNA [NM_017570]
0,43	0,00985	ART5	NM_053017	Homo sapiens ADP-ribosyltransferase 5 (ART5), transcript variant 1, mRNA [NM_053017]
0,43	0,00089	SLC7A8	NM_182728	Homo sapiens solute carrier family 7 (cationic amino acid transporter, y+ system), member 8 (SLC7A8), transcript variant 2, mRNA [NM_182728]
0,43	0,00034	NPC1	NM_000271	Homo sapiens Niemann-Pick disease, type C1 (NPC1), mRNA [NM_000271]

0,43	0,00037	MMP11	NM_005940	Homo sapiens matrix metallopeptidase 11 (stromelysin 3) (MMP11), mRNA [NM_005940]
0,43	0,00227	MACF1	NM_012090	Homo sapiens microtubule-actin crosslinking factor 1 (MACF1), transcript variant 1, mRNA [NM_012090]
0,44	0,00038	ENST00000324745	ENST00000324745	Homo sapiens mRNA for FLJ00388 protein. [AK090467]
0,44	0,00133	PTGDS	NM_000954	Homo sapiens prostaglandin D2 synthase 21kDa (brain) (PTGDS), mRNA [NM_000954]
0,44	0,00107	DNASE2	NM_001375	Homo sapiens deoxyribonuclease II, lysosomal (DNASE2), mRNA [NM_001375]
0,44	0,00668	DAB2	NM_001343	Homo sapiens disabled homolog 2, mitogen-responsive phosphoprotein (Drosophila) (DAB2), mRNA [NM_001343]
0,44	0,00049	HOOK1	NM_015888	Homo sapiens hook homolog 1 (Drosophila) (HOOK1), mRNA [NM_015888]
0,44	0,00839	DACT2	NM_214462	Homo sapiens dapper, antagonist of beta-catenin, homolog 2 (Xenopus laevis) (DACT2), mRNA [NM_214462]
0,44	0,00669	KISS1R	NM_032551	Homo sapiens KISS1 receptor (KISS1R), mRNA [NM_032551]
0,44	0,00546	GABARAPL1	NM_031412	Homo sapiens GABA(A) receptor-associated protein like 1 (GABARAPL1), mRNA [NM_031412]
0,44	0,00103	TJP2	NM_201629	Homo sapiens tight junction protein 2 (zona occludens 2) (TJP2), transcript variant 2, mRNA [NM_201629]
0,44	0,00905	IL6R	NM_000565	Homo sapiens interleukin 6 receptor (IL6R), transcript variant 1, mRNA [NM_000565]
0,44	0,00282	RNF175	NM_173662	Homo sapiens ring finger protein 175 (RNF175), mRNA [NM_173662]
0,44	0,00187	FLT3	NM_004119	Homo sapiens fms-related tyrosine kinase 3 (FLT3), mRNA [NM_004119]
0,44	0,00269	FAM19A5	NM_015381	Homo sapiens family with sequence similarity 19 (chemokine (C-C motif)-like), member A5 (FAM19A5), transcript variant 2, mRNA [NM_015381]
0,44	0,00006	RHOBTB2	NM_015178	Homo sapiens Rho-related BTB domain containing 2 (RHOBTB2), mRNA [NM_015178]
0,44	0,00146	K03200	K03200	Human melanoma-associated antigen p97 (melanotransferrin) mRNA, 3' flank. [K03200]
0,44	0,00188	LRP2	NM_004525	Homo sapiens low density lipoprotein-related protein 2 (LRP2), mRNA [NM_004525]
0,45	0,00096	THC2603259	THC2603259	Q96IM5_HUMAN (Q96IM5) RAB7B protein, complete [THC2603259]
0,45	0,00102	FAM110C	NM_001077710	Homo sapiens family with sequence similarity 110, member C (FAM110C), mRNA [NM_001077710]
0,45	0,00573	AMHR2	NM_020547	Homo sapiens anti-Mullerian hormone receptor, type II (AMHR2), mRNA [NM_020547]
0,45	0,00189	MUM1L1	NM_152423	Homo sapiens melanoma associated antigen (mutated) 1-like 1 (MUM1L1), mRNA [NM_152423]

0,45	0,00148	HSPA1A	NM_005345	Homo sapiens heat shock 70kDa protein 1A (HSPA1A), mRNA [NM_005345]
0,45	0,00430	FAAH2	NM_174912	Homo sapiens fatty acid amide hydrolase 2 (FAAH2), mRNA [NM_174912]
0,45	0,00074	IFIH1	NM_022168	Homo sapiens interferon induced with helicase C domain 1 (IFIH1), mRNA [NM_022168]
0,45	0,00141	USP33	NM_201626	Homo sapiens ubiquitin specific peptidase 33 (USP33), transcript variant 3, mRNA [NM_201626]
0,45	0,00188	NAP5	NM_207363	Homo sapiens Nck-associated protein 5 (NAP5), transcript variant 1, mRNA [NM_207363]
0,45	0,00773	ENST0000037 1276	ENST00000371276	Tetratricopeptide repeat protein 22 (TPR repeat protein 22). [Source:Uniprot/SWISSPROT;Acc:Q5TAA0] [ENST00000371276]
0,45	0,00271	MST1	NM_020998	Homo sapiens macrophage stimulating 1 (hepatocyte growth factor-like) (MST1), mRNA [NM_020998]
0,45	0,00158	ADAM9	NM_001005845	Homo sapiens ADAM metallopeptidase domain 9 (meltrin gamma) (ADAM9), transcript variant 2, mRNA [NM_001005845]
0,45	0,00055	ZNF395	NM_018660	Homo sapiens zinc finger protein 395 (ZNF395), mRNA [NM_018660]
0,45	0,00039	DDR1	NM_013994	Homo sapiens discoidin domain receptor family, member 1 (DDR1), transcript variant 3, mRNA [NM_013994]
0,45	0,00145	CTAGE5	NM_203356	Homo sapiens CTAGE family, member 5 (CTAGE5), transcript variant 4, mRNA [NM_203356]
0,45	0,00269	NOXA1	NM_006647	Homo sapiens NADPH oxidase activator 1 (NOXA1), mRNA [NM_006647]
0,45	0,00269	FOXE1	X94553	H.sapiens HFKH4 mRNA for fork head like protein. [X94553]
0,46	0,00380	LAMA5	NM_005560	Homo sapiens laminin, alpha 5 (LAMA5), mRNA [NM_005560]
0,46	0,00736	THC2734788	THC2734788	T28605 EST48922 Human Spleen Homo sapiens cDNA 5' end similar to proto-oncogene junD (GB:X51346) (HT:1498), mRNA sequence [T28605]
0,46	0,00015	TMCO4	NM_181719	Homo sapiens transmembrane and coiled-coil domains 4 (TMCO4), mRNA [NM_181719]
0,46	0,00080	BMP4	NM_001202	Homo sapiens bone morphogenetic protein 4 (BMP4), transcript variant 1, mRNA [NM_001202]
0,46	0,00113	TMEFF2	AB004064	Homo sapiens mRNA for tomoregulin, complete cds. [AB004064]
0,46	0,00181	KARCA1	NM_001007255	Homo sapiens kelch/ankyrin repeat containing cyclin A1 interacting protein (KARCA1), transcript variant 2, mRNA [NM_001007255]
0,46	0,00208	AFG3L2	NM_006796	Homo sapiens AFG3 ATPase family gene 3-like 2 (yeast) (AFG3L2), nuclear gene encoding mitochondrial protein, mRNA [NM_006796]
0,46	0,00717	BZRAP1	NM_004758	Homo sapiens benzodiazapine receptor (peripheral) associated protein 1 (BZRAP1), mRNA [NM_004758]
0,47	0,00389	RTN4R	NM_023004	Homo sapiens reticulon 4 receptor (RTN4R), mRNA [NM_023004]
0,47	0,00847	EIF2AK2	NM_002759	Homo sapiens eukaryotic translation initiation factor 2-alpha kinase 2 (EIF2AK2), mRNA [NM_002759]

0,47	0,00139	CENTG2	NM_014914	Homo sapiens centaurin, gamma 2 (CENTG2), transcript variant 2, mRNA [NM_014914]
0,47	0,00072	PRKCZ	NM_002744	Homo sapiens protein kinase C, zeta (PRKCZ), transcript variant 1, mRNA [NM_002744]
0,47	0,00021	LOC441294	NM_001008747	Homo sapiens similar to CTAGE6 (LOC441294), mRNA [NM_001008747]
0,47	0,00260	ENST00000376840	ENST00000376840	TBC1 domain family member 8 (Vascular Rab-GAP/TBC-containing protein) (AD 3). [Source:Uniprot/SWISSPROT;Acc:O95759] [ENST00000376840]
0,47	0,00531	SEMA3B	NM_004636	Homo sapiens sema domain, immunoglobulin domain (Ig), short basic domain, secreted, (semaphorin) 3B (SEMA3B), transcript variant 1, mRNA [NM_004636]
0,47	0,00996	STMN1	NM_203401	Homo sapiens stathmin 1/oncoprotein 18 (STMN1), transcript variant 1, mRNA [NM_203401]
0,47	0,00105	CFI	NM_000204	Homo sapiens complement factor I (CFI), mRNA [NM_000204]
0,47	0,00026	LLGL2	NM_001015002	Homo sapiens lethal giant larvae homolog 2 (Drosophila) (LLGL2), transcript variant 2, mRNA [NM_001015002]
0,47	0,00261	MYO5C	NM_018728	Homo sapiens myosin VC (MYO5C), mRNA [NM_018728]
0,47	0,00208	PIP5K2A	NM_005028	Homo sapiens phosphatidylinositol-4-phosphate 5-kinase, type II, alpha (PIP5K2A), mRNA [NM_005028]
0,47	0,00008	PAQR6	NM_024897	Homo sapiens progestin and adipoQ receptor family member VI (PAQR6), transcript variant 1, mRNA [NM_024897]
0,47	0,00313	PPARGC1B	NM_133263	Homo sapiens peroxisome proliferator-activated receptor gamma, coactivator 1 beta (PPARGC1B), mRNA [NM_133263]
0,47	0,00405	LOH11CR2A	NM_014622	Homo sapiens loss of heterozygosity, 11, chromosomal region 2, gene A (LOH11CR2A), transcript variant 1, mRNA [NM_014622]
0,47	0,00989	ANXA3	NM_005139	Homo sapiens annexin A3 (ANXA3), mRNA [NM_005139]
0,47	0,00235	RIPK3	NM_006871	Homo sapiens receptor-interacting serine-threonine kinase 3 (RIPK3), mRNA [NM_006871]
0,47	0,00055	X03757	X03757	Human mRNA fragment for Na/K-ATPase alpha subunit. [X03757]
0,48	0,00673	TRIM63	NM_032588	Homo sapiens tripartite motif-containing 63 (TRIM63), mRNA [NM_032588]
0,48	0,00076	SLC35E2	NM_182838	Homo sapiens solute carrier family 35, member E2 (SLC35E2), mRNA [NM_182838]
0,48	0,00532	RAB7B	NM_177403	Homo sapiens RAB7B, member RAS oncogene family (RAB7B), mRNA [NM_177403]
0,48	0,00503	EVX1	NM_001989	Homo sapiens even-skipped homeobox 1 (EVX1), mRNA [NM_001989]
0,48	0,00043	FAM84B	NM_174911	Homo sapiens family with sequence similarity 84, member B (FAM84B), mRNA [NM_174911]
0,48	0,00076	HIBADH	NM_152740	Homo sapiens 3-hydroxyisobutyrate dehydrogenase (HIBADH), mRNA [NM_152740]

0,48	0,00673	KIRREL2	NM_199180	Homo sapiens kin of IRRE like 2 (Drosophila) (KIRREL2), transcript variant 3, mRNA [NM_199180]
0,48	0,00144	MOP-1	AB014771	Homo sapiens mRNA for MOP-1, complete cds. [AB014771]
0,48	0,00282	MERTK	U08023	Human cellular proto-oncogene (c-mer) mRNA, complete cds. [U08023]
0,48	0,00398	HEXA	NM_000520	Homo sapiens hexosaminidase A (alpha polypeptide) (HEXA), mRNA [NM_000520]
0,48	0,00034	CACNA1H	NM_021098	Homo sapiens calcium channel, voltage-dependent, T type, alpha 1H subunit (CACNA1H), transcript variant 1, mRNA [NM_021098]
0,48	0,00497	ALDH3B1	NM_000694	Homo sapiens aldehyde dehydrogenase 3 family, member B1 (ALDH3B1), transcript variant 1, mRNA [NM_000694]
0,49	0,00196	B3GALT4	NM_003782	Homo sapiens UDP-Gal:betaGlcNAc beta 1,3-galactosyltransferase, polypeptide 4 (B3GALT4), mRNA [NM_003782]
0,49	0,00051	SERPING1	NM_000062	Homo sapiens serpin peptidase inhibitor, clade G (C1 inhibitor), member 1, (angioedema, hereditary) (SERPING1), transcript variant 1, mRNA [NM_000062]
0,49	0,00038	USP51	NM_201286	Homo sapiens ubiquitin specific peptidase 51 (USP51), mRNA [NM_201286]
0,49	0,00209	EXPH5	NM_015065	Homo sapiens exophilin 5 (EXPH5), mRNA [NM_015065]
0,49	0,00043	MTA3	ENST00000282366	Metastasis-associated protein MTA3. [Source:Uniprot/SWISSPROT;Acc:Q9BTC8] [ENST00000282366]
0,49	0,00139	PCDHA8	NM_031856	Homo sapiens protocadherin alpha 8 (PCDHA8), transcript variant 2, mRNA [NM_031856]
0,49	0,00181	NFIL3	NM_005384	Homo sapiens nuclear factor, interleukin 3 regulated (NFIL3), mRNA [NM_005384]
0,49	0,00635	SLC27A2	NM_003645	Homo sapiens solute carrier family 27 (fatty acid transporter), member 2 (SLC27A2), mRNA [NM_003645]
0,49	0,00061	NEIL1	NM_024608	Homo sapiens nei endonuclease VIII-like 1 (E. coli) (NEIL1), mRNA [NM_024608]
0,49	0,00614	CDKN1C	NM_000076	Homo sapiens cyclin-dependent kinase inhibitor 1C (p57, Kip2) (CDKN1C), mRNA [NM_000076]
0,49	0,00034	STAT1	NM_139266	Homo sapiens signal transducer and activator of transcription 1, 91kDa (STAT1), transcript variant beta, mRNA [NM_139266]
0,49	0,00935	BMP7	NM_001719	Homo sapiens bone morphogenetic protein 7 (osteogenic protein 1) (BMP7), mRNA [NM_001719]
0,49	0,00145	PAK6	NM_020168	Homo sapiens p21(CDKN1A)-activated kinase 6 (PAK6), mRNA [NM_020168]
0,49	0,00187	SMARCD3	NM_003078	Homo sapiens SWI/SNF related, matrix associated, actin dependent regulator of chromatin, subfamily d, member 3 (SMARCD3), transcript variant 2, mRNA [NM_003078]
0,49	0,00315	CD79A	NM_001783	Homo sapiens CD79a molecule, immunoglobulin-associated alpha (CD79A), transcript variant 1, mRNA [NM_001783]

0,49	0,00669	PIP5K1B	NM_003558	Homo sapiens phosphatidylinositol-4-phosphate 5-kinase, type I, beta (PIP5K1B), transcript variant 2, mRNA [NM_003558]
0,49	0,00029	FUCA1	NM_000147	Homo sapiens fucosidase, alpha-L- 1, tissue (FUCA1), mRNA [NM_000147]
0,49	0,00104	PDGFD	NM_025208	Homo sapiens platelet derived growth factor D (PDGFD), transcript variant 1, mRNA [NM_025208]
0,49	0,00138	SPSB1	NM_025106	Homo sapiens splA/ryanodine receptor domain and SOCS box containing 1 (SPSB1), mRNA [NM_025106]
0,49	0,00188	ATP10D	NM_020453	Homo sapiens ATPase, Class V, type 10D (ATP10D), mRNA [NM_020453]
0,49	0,00446	GPRC5B	NM_016235	Homo sapiens G protein-coupled receptor, family C, group 5, member B (GPRC5B), mRNA [NM_016235]
0,50	0,00561	RNF122	NM_024787	Homo sapiens ring finger protein 122 (RNF122), mRNA [NM_024787]
0,50	0,00152	ATP1A4	NM_144699	Homo sapiens ATPase, Na+/K+ transporting, alpha 4 polypeptide (ATP1A4), transcript variant 1, mRNA [NM_144699]
0,50	0,00086	TSPAN3	NM_005724	Homo sapiens tetraspanin 3 (TSPAN3), transcript variant 1, mRNA [NM_005724]
0,50	0,00704	FLJ41603	NM_001001669	Homo sapiens FLJ41603 protein (FLJ41603), mRNA [NM_001001669]
0,50	0,00027	CBX7	NM_175709	Homo sapiens chromobox homolog 7 (CBX7), mRNA [NM_175709]
0,50	0,00284	BMP2K	AB015331	Homo sapiens HRIHFB2017 mRNA, partial cds. [AB015331]
0,50	0,00694	TNIK	AB011123	Homo sapiens mRNA for KIAA0551 protein, partial cds. [AB011123]
0,50	0,00115	SOCS2	NM_003877	Homo sapiens suppressor of cytokine signaling 2 (SOCS2), mRNA [NM_003877]
0,50	0,00979	CPXM1	NM_019609	Homo sapiens carboxypeptidase X (M14 family), member 1 (CPXM1), mRNA [NM_019609]
0,50	0,00509	SPATA2L	NM_152339	Homo sapiens spermatogenesis associated 2-like (SPATA2L), mRNA [NM_152339]
0,50	0,00122	C3AR1	NM_004054	Homo sapiens complement component 3a receptor 1 (C3AR1), mRNA [NM_004054]
0,50	0,00160	SCARNA17	NR_003003	Homo sapiens small Cajal body-specific RNA 17 (SCARNA17) on chromosome 18 [NR_003003]
0,50	0,00188	CTNNAL1	NM_003798	Homo sapiens catenin (cadherin-associated protein), alpha-like 1 (CTNNAL1), mRNA [NM_003798]

Supplementary Table S9. Canonical Pathways in Epithelioid phenotype.

Ingenuity Canonical Pathways	-log(p-value)	Ratio	z-score	Molecules
Agranulocyte Adhesion and Diapedesis	4,12E00	7,43E-02	NaN	IL1A,IL18,MYH2,CLDN15,CLDN1,CXCL14,MYH3,PODXL,CCL26,CXCL2,CXCL6,MMP1,MYL7
Glioblastoma Multiforme Signaling	3,05E00	6,9E-02	-2,530	CDKN2A,PLCB4,PLCE1,WNT10A,DIRAS3,WNT2B,PLCB1,WNT4,ITPR1,PDGFRB
PI3K Signaling in B Lymphocytes	2,98E00	7,32E-02	-1,667	FOS,PLCB4,IL4R,PLCE1,C3,DAPP1,PLCB1,VAV1,ITPR1
Cellular Effects of Sildenafil (Viagra)	2,95E00	7,26E-02	NaN	PLCB4,PLCE1,MYH2,PPP1R12B,MYLPF,MYH3,PLCB1,ITPR1,MYL7
FXR/RXR Activation	2,93E00	7,2E-02	NaN	PPARG,APOE,HPR,IL1A,IL18,C3,APOA1,VTN,CLU
Role of Osteoblasts, Osteoclasts and Chondrocytes in Rheumatoid Arthritis	2,76E00	5,61E-02	NaN	FOS,IL1A,IL18,CTSK,WNT10A,DKK3,WNT2B,WNT4,SFRP1,DKK1,ADAMTS5,MMP1
Melatonin Degradation II	2,6E00	5E-01	NaN	MAOB,IL4I1
NAD Biosynthesis III	2,6E00	5E-01	NaN	NMNAT2,NAMPT
Phenylalanine Degradation IV (Mammalian, via Side Chain)	2,57E00	2,14E-01	NaN	MAOB,HPD,IL4I1
Role of Macrophages, Fibroblasts and Endothelial Cells in Rheumatoid Arthritis	2,56E00	4,88E-02	NaN	FOS,PLCB4,IL1A,IL18,PLCE1,WNT10A,DKK3,WNT2B,PLCB1,WNT4,SFRP1,DKK1,CREB5,MMP1
LXR/RXR Activation	2,43E00	6,61E-02	-1,414	APOE,HPR,IL1A,IL18,C3,APOA1,VTN,CLU
Hepatic Fibrosis / Hepatic Stellate Cell Activation	2,34E00	5,52E-02	NaN	IL1A,IL4R,MYH2,COL4A6,COL9A3,MYH3,COL27A1,MMP1,PDGFRB,MYL7
Putrescine Degradation III	2,32E00	1,76E-01	NaN	MAOB,ALDH1A1,IL4I1
Cholecystokinin/Gastrin-mediated Signaling	2,3E00	6,93E-02	-1,134	FOS,PLCB4,IL1A,IL18,DIRAS3,PLCB1,ITPR1
VDR/RXR Activation	2,28E00	7,79E-02	NaN	IGFBP6,WT1,HES1,VDR,THBD,CST6
Tryptophan Degradation X (Mammalian, via Tryptamine)	2,24E00	1,67E-01	NaN	MAOB,ALDH1A1,IL4I1
Thrombin Signaling	2,24E00	5,35E-02	-1,897	PLCB4,GATA5,PLCE1,PPP1R12B,F2R,MYLPF,DIRAS3,PLCB1,ITPR1,MYL7
Sphingosine-1-phosphate Signaling	2,14E00	6,48E-02	-1,633	PLCB4,PLCE1,DIRAS3,SPHK1,PLCB1,ASAH1,PDGFRB
Granulocyte Adhesion and Diapedesis	2,12E00	5,45E-02	NaN	IL1A,IL18,CLDN15,CLDN1,CXCL14,CCL26,CXCL2,CXCL6,MMP1
Inhibition of Matrix Metalloproteases	2,12E00	1,05E-01	NaN	TIMP3,ADAM12,TFPI2,MMP1
Acute Phase Response Signaling	2,07E00	5,36E-02	-0,816	FOS,ITIH3,IL1A,IL18,HP,C3,APOA1,CFB,RBP1

PCP pathway	2,04E00	8,06E-02	-0,447	RSPO3,WNT10A,WNT2B,CTHRC1,WNT4
Dopamine Degradation	1,99E00	1,36E-01	NaN	MAOB,ALDH1A1,IL4I1
Atherosclerosis Signaling	1,89E00	5,79E-02	NaN	APOE,IL1A,IL18,APOA1,PLA2G7,CLU,MMP1
ILK Signaling	1,88E00	4,97E-02	-1,414	FOS,CDH1,MYH2,SNAI2,LIMS1,DIRAS3,MYH3,CREB5,MYL7
Chemokine Signaling	1,88E00	7,35E-02	-0,447	FOS,PLCB4,PPP1R12B,PLCB1,NOX1
Prostanoid Biosynthesis	1,85E00	2,22E-01	NaN	PTGS1,PTGDS
UDP-N-acetyl-D-galactosamine Biosynthesis II	1,85E00	2,22E-01	NaN	GNPDA1,GALE
GPCR-Mediated Integration of Enteroendocrine Signaling Exemplified by an L Cell	1,83E00	7,14E-02	NaN	PLCB4,PLCE1,NMB,PLCB1,ITPR1
D-myo-inositol (1,4,5)-Trisphosphate Biosynthesis	1,79E00	1,15E-01	NaN	PLCB4,PLCE1,PLCB1
phagosome formation	1,71E00	5,88E-02	NaN	PLCB4,PLCE1,SCARA3,DIRAS3,VTN,PLCB1
UDP-N-acetyl-D-galactosamine Biosynthesis I	1,68E00	1E00	NaN	GALE
Aryl Hydrocarbon Receptor Signaling	1,65E00	5,19E-02	NaN	CDKN2A,FOS,IL1A,CCND2,ALDH1A1,ALDH8A1,AHR
Tight Junction Signaling	1,65E00	4,82E-02	NaN	FOS,MYH2,CLDN15,CLDN1,CGN,MYH3,JAM2,MYL7
Wnt/β-catenin Signaling	1,65E00	4,82E-02	-1,633	CDKN2A,CDH1,WNT10A,DKK3,WNT2B,WNT4,SFRP1,DKK1
Retinoate Biosynthesis I	1,63E00	1E-01	NaN	ALDH1A1,ALDH8A1,RBP1
Androgen Biosynthesis	1,61E00	1,67E-01	NaN	CYP17A1,HSD17B14
Calcium Signaling	1,59E00	4,71E-02	NaN	RCAN1,TNNT1,MYH2,MYH3,ITPR1,CREB5,CHRNA3,MYL7
Phospholipases	1,57E00	7,27E-02	NaN	PLCB4,PLCE1,PLCB1,PLA2G7
Wnt/Ca+ pathway	1,57E00	7,27E-02	-2,000	PLCB4,PLCE1,PLCB1,CREB5
NAD biosynthesis II (from tryptophan)	1,54E00	1,54E-01	NaN	TDO2,NMNAT2
Histamine Degradation	1,54E00	1,54E-01	NaN	HNMT,ALDH1A1
LPS/IL-1 Mediated Inhibition of RXR Function	1,53E00	4,33E-02	NaN	APOE,MAOB,IL1A,IL18,ALDH1A1,ALDH8A1,HS6ST2,HS3ST3A1,IL4I1
Glioma Invasiveness Signaling	1,52E00	7,02E-02	0,000	TIMP3,F2R,DIRAS3,VTN
Noradrenaline and Adrenaline Degradation	1,52E00	9,09E-02	NaN	MAOB,ALDH1A1,IL4I1
Actin Cytoskeleton Signaling	1,51E00	4,29E-02	-1,134	MYH2,CYFIP2,PPP1R12B,F2R,MYLPF,FGF9,MYH3,VAV1,MYL7
Sperm Motility	1,5E00	5,26E-02	-1,633	NPPB,PLCB4,PLCE1,PLCB1,ITPR1,PLA2G7
Gα12/13 Signaling	1,46E00	5,13E-02	0,000	CDH1,F2R,MYLPF,CDH10,VAV1,MYL7
Coagulation System	1,45E00	8,57E-02	NaN	F2R,THBD,PLAT

CXCR4 Signaling	1,42E00	4,64E-02	-2,449	FOS,PLCB4,MYLPF,DIRAS3,PLCB1,ITPR1,MYL7
PPAR Signaling	1,41E00	5,56E-02	0,447	PPARG,FOS,IL1A,IL18,PDGFRB
Cardiac Hypertrophy Signaling	1,4E00	4,09E-02	-2,828	NKX2-5,PLCB4,PLCE1,ADRA2A,MYLPF,DIRAS3,PLCB1,ADRA2C,MYL7
Phospholipase C Signaling	1,29E00	3,9E-02	-2,121	PLCB4,PLCE1,PPP1R12B,MYLPF,DIRAS3,PLCB1,ITPR1,CREB5,MYL7
IL-10 Signaling	1,28E00	5,88E-02	NaN	FOS,IL1A,IL18,IL4R
Ovarian Cancer Signaling	1,27E00	4,62E-02	NaN	CDKN2A,WNT10A,FGF9,WNT2B,PTGS1,WNT4
Basal Cell Carcinoma Signaling	1,26E00	5,8E-02	-2,000	WNT10A,WNT2B,WNT4,HHIP
Neuropathic Pain Signaling In Dorsal Horn Neurons	1,26E00	5,05E-02	-1,342	FOS,PLCB4,PLCE1,PLCB1,ITPR1
IL-12 Signaling and Production in Macrophages	1,26E00	4,58E-02	NaN	PPARG,APOE,FOS,IL18,APOA1,CLU
Integrin Signaling	1,24E00	4E-02	-0,816	CAPN6,PPP1R12B,TSPAN7,LIMS1,ITGA11,DIRAS3,ITGA7,MYL7
Cardiomyocyte Differentiation via BMP Receptors	1,24E00	1,05E-01	NaN	NPPB,NKX2-5
Serotonin Receptor Signaling	1,23E00	6,98E-02	NaN	MAOB,HTR2B,IL4I1
Endothelin-1 Signaling	1,22E00	4,17E-02	-1,134	FOS,PLCB4,PLCE1,PTGS1,PLCB1,ITPR1,PLA2G7
N-acetylglucosamine Degradation I	1,22E00	3,33E-01	NaN	GNPDA1
Dendritic Cell Maturation	1,21E00	4,14E-02	-1,890	PLCB4,IL1A,IL18,PLCE1,FSCN1,PLCB1,CREB5
RhoGDI Signaling	1,17E00	4,07E-02	1,134	CDH1,PPP1R12B,ARHGDIG,MYLPF,DIRAS3,CDH10,MYL7
Heparan Sulfate Biosynthesis (Late Stages)	1,11E00	6,25E-02	NaN	HS6ST2,EXTL1,HS3ST3A1
Arsenate Detoxification I (Glutaredoxin)	1,1E00	2,5E-01	NaN	AS3MT
Retinoate Biosynthesis II	1,1E00	2,5E-01	NaN	RBP1
N-acetylglucosamine Degradation II	1,1E00	2,5E-01	NaN	GNPDA1
Gαq Signaling	1,09E00	4,14E-02	-1,633	RGS2,PLCB4,HTR2B,DIRAS3,PLCB1,ITPR1
Regulation of the Epithelial-Mesenchymal Transition Pathway	1,07E00	3,85E-02	NaN	CDH1,SNAI2,WNT10A,FGF9,WNT2B,WNT4,PDGFRB
CCR3 Signaling in Eosinophils	1,07E00	4,42E-02	NaN	PLCB4,PPP1R12B,PLCB1,CCL26,ITPR1
Synaptic Long Term Potentiation	1,03E00	4,31E-02	-2,236	PLCB4,PLCE1,PLCB1,ITPR1,CREB5
GPCR-Mediated Nutrient Sensing in Enteroendocrine Cells	1,03E00	4,82E-02	NaN	PLCB4,PLCE1,PLCB1,ITPR1
HMGB1 Signaling	1,01E00	4,24E-02	-0,447	FOS,IL1A,IL18,DIRAS3,PLAT

p70S6K Signaling	1,01E00	4,24E-02	-0,447	PLCB4,IL4R,PLCE1,F2R,PLCB1
P2Y Purigenic Receptor Signaling Pathway	1,01E00	4,24E-02	-1,342	FOS,PLCB4,PLCE1,PLCB1,CREB5
Creatine-phosphate Biosynthesis	1E00	2E-01	NaN	CKB
Serine Biosynthesis	1E00	2E-01	NaN	PHGDH
NAD Salvage Pathway III	1E00	2E-01	NaN	NMNAT2
Lysine Degradation II	1E00	2E-01	NaN	AASS
Galactose Degradation I (Leloir Pathway)	1E00	2E-01	NaN	GALE
Tyrosine Degradation I	1E00	2E-01	NaN	HPD
Bladder Cancer Signaling	9,87E-01	4,65E-02	NaN	CDKN2A,CDH1,FGF9,MMP1
Heparan Sulfate Biosynthesis	9,77E-01	5,45E-02	NaN	HS6ST2,EXTL1,HS3ST3A1
Superpathway of Melatonin Degradation	9,77E-01	5,45E-02	NaN	MAOB,CYP2J2,IL4I1
Regulation of Actin-based Motility by Rho	9,73E-01	4,6E-02	-1,000	PPP1R12B,MYLPF,DIRAS3,MYL7
UVA-Induced MAPK Signaling	9,73E-01	4,6E-02	NaN	FOS,PLCB4,PLCE1,PLCB1
Leukocyte Extravasation Signaling	9,71E-01	3,63E-02	NaN	TIMP3,CLDN15,CLDN1,JAM2,VAV1,NOX1,MMP1
Axonal Guidance Signaling	9,66E-01	3,04E-02	NaN	PLCB4,PLCE1,ADAM12,KALRN,WNT10A,MYLPF,WNT2B,PLCB1,WNT4,HHIP,ADAMTS5,ADAM8,MYL7
Dopamine-DARPP32 Feedback in cAMP Signaling	9,66E-01	3,82E-02	-1,633	KCNJ8,PLCB4,PLCE1,PLCB1,ITPR1,CREB5
Serotonin Degradation	9,6E-01	5,36E-02	NaN	MAOB,ALDH1A1,IL4I1
Signaling by Rho Family GTPases	9,5E-01	3,43E-02	-1,414	FOS,CDH1,PPP1R12B,MYLPF,DIRAS3,CDH10,NOX1,MYL7
NAD Biosynthesis from 2-amino-3-carboxymuconate Semialdehyde	9,28E-01	1,67E-01	NaN	NMNAT2
Ceramide Degradation	9,28E-01	1,67E-01	NaN	ASAH1
GNRH Signaling	9,07E-01	3,94E-02	-1,342	FOS,PLCB4,PLCB1,ITPR1,CREB5
Molecular Mechanisms of Cancer	8,94E-01	3,06E-02	NaN	CDKN2A,FOS,PLCB4,CDH1,CCND2,WNT10A,DIRAS3,WNT2B,PLCB1,WNT4,CDKN2B
Cdc42 Signaling	8,86E-01	3,88E-02	-0,447	FOS,PPP1R12B,MYLPF,VAV1,MYL7
G Protein Signaling Mediated by Tubby	8,71E-01	6,45E-02	NaN	PLCB4,PLCB1
Antioxidant Action of Vitamin C	8,71E-01	4,21E-02	1,000	PLCB4,PLCE1,PLCB1,PLA2G7
Human Embryonic Stem Cell Pluripotency	8,66E-01	3,82E-02	NaN	WNT10A,WNT2B,SPHK1,WNT4,PDGFRB

Glucose and Glucose-1-phosphate Degradation	8,66E-01	1,43E-01	NaN	RGN
Superpathway of Serine and Glycine Biosynthesis I	8,66E-01	1,43E-01	NaN	PHGDH
Tryptophan Degradation to 2-amino-3-carboxymuconate Semialdehyde	8,66E-01	1,43E-01	NaN	TDO2
Eicosanoid Signaling	8,63E-01	4,84E-02	NaN	PTGS1,PTGDS,PLA2G7
Estrogen-Dependent Breast Cancer Signaling	8,48E-01	4,76E-02	NaN	FOS,CREB5,HSD17B14
Cell Cycle: G1/S Checkpoint Regulation	8,48E-01	4,76E-02	NaN	CDKN2A,CCND2,CDKN2B
p53 Signaling	8,36E-01	4,08E-02	NaN	CDKN2A,WT1,CCND2,SNAI2
Protein Kinase A Signaling	8,31E-01	2,97E-02	-1,000	AKAP12,HIST1H1C,PLCB4,PLCE1,MYH2,PTPN13,MYLPF,PLCB1,ITPR1,CREB5,MYL7
Synaptic Long Term Depression	8,18E-01	3,68E-02	-1,342	PLCB4,PLCE1,PLCB1,ITPR1,PLA2G7
Airway Pathology in Chronic Obstructive Pulmonary Disease	8,12E-01	1,25E-01	NaN	MMP1
Sphingosine and Sphingosine-1-phosphate Metabolism	8,12E-01	1,25E-01	NaN	ASAH1
Glucocorticoid Biosynthesis	8,12E-01	1,25E-01	NaN	CYP17A1
Oncostatin M Signaling	8,07E-01	5,88E-02	NaN	TIMP3,MMP1
Role of NFAT in Cardiac Hypertrophy	7,98E-01	3,41E-02	-1,633	RCAN1,NKX2-5,PLCB4,PLCE1,PLCB1,ITPR1
Xenobiotic Metabolism Signaling	7,88E-01	3,12E-02	NaN	MAOB,IL1A,ALDH1A1,ALDH8A1,HS6ST2,HS3ST3A1,AHR,IL4I1
IL-17A Signaling in Fibroblasts	7,87E-01	5,71E-02	NaN	FOS,MMP1
Melatonin Signaling	7,78E-01	4,41E-02	NaN	PLCB4,PLCE1,PLCB1
Complement System	7,68E-01	5,56E-02	NaN	C3,CFB
Epithelial Adherens Junction Signaling	7,55E-01	3,5E-02	NaN	CDH1,MYH2,SNAI2,MYH3,MYL7
Estrogen Biosynthesis	7,49E-01	5,41E-02	NaN	CYP2J2,HSD17B14
IL-8 Signaling	7,45E-01	3,28E-02	-1,633	FOS,CDH1,CCND2,DIRAS3,NOX1,MYL7
fMLP Signaling in Neutrophils	7,41E-01	3,74E-02	-2,000	PLCB4,PLCB1,ITPR1,NOX1
Corticotropin Releasing Hormone Signaling	7,41E-01	3,74E-02	NaN	FOS,IVL,ITPR1,CREB5
Clathrin-mediated Endocytosis Signaling	7,37E-01	3,26E-02	NaN	APOE,APOA1,F2R,SH3GL3,FGF9,CLU
Role of Wnt/GSK-3β Signaling in the Pathogenesis of Influenza	7,28E-01	4,17E-02	NaN	WNT10A,WNT2B,WNT4

IL-4 Signaling	7,28E-01	4,17E-02	NaN	IL4R,IL13RA1,HMGA1
Embryonic Stem Cell Differentiation into Cardiac Lineages	7,24E-01	1E-01	NaN	NKX2-5
Role of NANOG in Mammalian Embryonic Stem Cell Pluripotency	7,22E-01	3,67E-02	NaN	WNT10A,WNT2B,WNT4,ZFP42
Leptin Signaling in Obesity	7,16E-01	4,11E-02	NaN	PLCB4,PLCE1,PLCB1
Toll-like Receptor Signaling	7,16E-01	4,11E-02	NaN	FOS,IL1A,IL18
Thyroid Cancer Signaling	7,14E-01	5,13E-02	NaN	PPARG,CDH1
BMP signaling pathway	7,04E-01	4,05E-02	NaN	NKX2-5,FST,GREM1
Colorectal Cancer Metastasis Signaling	6,91E-01	3,03E-02	-0,378	FOS,CDH1,WNT10A,DIRAS3,WNT2B,WNT4,MMP1
Aldosterone Signaling in Epithelial Cells	6,9E-01	3,31E-02	-2,000	PLCB4,CRYAB,PLCE1,PLCB1,ITPR1
Gap Junction Signaling	6,9E-01	3,31E-02	NaN	PLCB4,HTR2B,PLCE1,PLCB1,ITPR1
Role of Hypercytokinemia/hyperchemokinemia in the Pathogenesis of Influenza	6,82E-01	4,88E-02	NaN	IL1A,IL18
Role of IL-17F in Allergic Inflammatory Airway Diseases	6,82E-01	4,88E-02	NaN	CREB5,CXCL6
Cyclins and Cell Cycle Regulation	6,7E-01	3,9E-02	NaN	CDKN2A,CCND2,CDKN2B
PDGF Signaling	6,7E-01	3,9E-02	NaN	FOS,SPHK1,PDGFRB
Melanoma Signaling	6,66E-01	4,76E-02	NaN	CDKN2A,CDH1
Dermatan Sulfate Biosynthesis (Late Stages)	6,66E-01	4,76E-02	NaN	HS6ST2,HS3ST3A1
14-3-3-mediated Signaling	6,58E-01	3,45E-02	-1,000	FOS,PLCB4,PLCE1,PLCB1
Glycogen Degradation III	6,53E-01	8,33E-02	NaN	MGAM
iNOS Signaling	6,51E-01	4,65E-02	NaN	FOS,HMGA1
Graft-versus-Host Disease Signaling	6,36E-01	4,55E-02	NaN	IL1A,IL18
Chondroitin Sulfate Biosynthesis (Late Stages)	6,36E-01	4,55E-02	NaN	HS6ST2,HS3ST3A1
Role of IL-17A in Psoriasis	6,23E-01	7,69E-02	NaN	CXCL6
Chondroitin Sulfate Degradation (Metazoa)	6,23E-01	7,69E-02	NaN	HEXA
Guanosine Nucleotides Degradation III	6,23E-01	7,69E-02	NaN	AOX1
nNOS Signaling in Neurons	6,08E-01	4,35E-02	NaN	CAPN6,DLG2

DNA Double-Strand Break Repair by Non-Homologous End Joining	5,95E-01	7,14E-02	NaN	XRCC4
Urate Biosynthesis/Inosine 5'-phosphate Degradation	5,95E-01	7,14E-02	NaN	AOX1
Dermatan Sulfate Degradation (Metazoa)	5,95E-01	7,14E-02	NaN	HEXA
Colanic Acid Building Blocks Biosynthesis	5,95E-01	7,14E-02	NaN	GALE
Nicotine Degradation III	5,95E-01	4,26E-02	NaN	CYP2J2,AOX1
FGF Signaling	5,89E-01	3,53E-02	NaN	FGF9,ITPR1,CREB5
Role of NFAT in Regulation of the Immune Response	5,83E-01	3,01E-02	0,000	RCAN1,FOS,PLCB4,PLCB1,ITPR1
TGF-β Signaling	5,7E-01	3,45E-02	NaN	NKX2-5,FOS,VDR
The Visual Cycle	5,7E-01	6,67E-02	NaN	RBP1
Oxidative Ethanol Degradation III	5,7E-01	6,67E-02	NaN	ALDH1A1
CREB Signaling in Neurons	5,64E-01	2,96E-02	-2,236	PLCB4,PLCE1,PLCB1,ITPR1,CREB5
G-Protein Coupled Receptor Signaling	5,61E-01	2,76E-02	NaN	RGS2,PLCB4,HTR2B,ADRA2A,PLCB1,ADRA2C,CREB5
PAK Signaling	5,61E-01	3,41E-02	NaN	MYLPF,PDGFRB,MYL7
Factors Promoting Cardiogenesis in Vertebrates	5,52E-01	3,37E-02	NaN	NPPB,NKX2-5,DKK1
Extrinsic Prothrombin Activation Pathway	5,46E-01	6,25E-02	NaN	THBD
Adenosine Nucleotides Degradation II	5,46E-01	6,25E-02	NaN	AOX1
Fatty Acid α-oxidation	5,46E-01	6,25E-02	NaN	ALDH1A1
Sertoli Cell-Sertoli Cell Junction Signaling	5,39E-01	2,89E-02	NaN	CDH1,CLDN15,CLDN1,CGN,JAM2
Adipogenesis pathway	5,34E-01	3,03E-02	NaN	PPARG,CDKN2A,TXNIP,RBP1
Role of Cytokines in Mediating Communication between Immune Cells	5,34E-01	3,85E-02	NaN	IL1A,IL18
Chondroitin Sulfate Biosynthesis	5,34E-01	3,85E-02	NaN	HS6ST2,HS3ST3A1
Granzyme A Signaling	5,24E-01	5,88E-02	NaN	HIST1H1C
Nicotine Degradation II	5,22E-01	3,77E-02	NaN	CYP2J2,AOX1
Unfolded protein response	5,22E-01	3,77E-02	NaN	PPARG,PDIA2
Role of IL-17A in Arthritis	5,11E-01	3,7E-02	NaN	CXCL6,MMP1

Production of Nitric Oxide and Reactive Oxygen Species in Macrophages	5,04E-01	2,79E-02	-0,447	APOE,FOS,APOA1,DIRAS3,CLU
Dermatan Sulfate Biosynthesis	5,01E-01	3,64E-02	NaN	HS6ST2,HS3ST3A1
T Cell Receptor Signaling	4,95E-01	3,12E-02	NaN	FOS,PAG1,VAV1
D-myo-inositol-5-phosphate Metabolism	4,94E-01	2,9E-02	NaN	PLCB4,PLCE1,PTPN13,PLCB1
Actin Nucleation by ARP-WASP Complex	4,9E-01	3,57E-02	NaN	PPP1R12B,DIRAS3
EGF Signaling	4,9E-01	3,57E-02	NaN	FOS,ITPR1
Glioma Signaling	4,87E-01	3,09E-02	NaN	CDKN2A,CDKN2B,PDGFRB
Huntington's Disease Signaling	4,86E-01	2,65E-02	-1,000	PLCB4,CAPN6,SH3GL3,PLCB1,ITPR1,CREB5
GADD45 Signaling	4,84E-01	5,26E-02	NaN	CCND2
Purine Nucleotides Degradation II (Aerobic)	4,84E-01	5,26E-02	NaN	AOX1
Ethanol Degradation IV	4,84E-01	5,26E-02	NaN	ALDH1A1
Tryptophan Degradation III (Eukaryotic)	4,66E-01	5E-02	NaN	TDO2
NAD Salvage Pathway II	4,49E-01	4,76E-02	NaN	NMNAT2
HGF Signaling	4,37E-01	2,88E-02	NaN	CDKN2A,FOS,ETS2
Polyamine Regulation in Colon Cancer	4,33E-01	4,55E-02	NaN	PPARG
ERK5 Signaling	4,25E-01	3,17E-02	NaN	FOS,CREB5
Differential Regulation of Cytokine Production in Intestinal Epithelial Cells by IL-17A and IL-17F	4,18E-01	4,35E-02	NaN	IL1A
Role of Tissue Factor in Cancer	4,18E-01	2,8E-02	NaN	PDIA2,PLCB1,MMP1
CD40 Signaling	4,16E-01	3,12E-02	NaN	FOS,PTGS1
Renin-Angiotensin Signaling	4,11E-01	2,78E-02	NaN	FOS,ITPR1,NOX1
Non-Small Cell Lung Cancer Signaling	4,08E-01	3,08E-02	NaN	CDKN2A,ITPR1
Bupropion Degradation	4,03E-01	4,17E-02	NaN	CYP2J2
T Helper Cell Differentiation	3,92E-01	2,99E-02	NaN	IL18,IL4R
Neurotrophin/TRK Signaling	3,92E-01	2,99E-02	NaN	FOS,CREB5
IL-17A Signaling in Gastric Cells	3,9E-01	4E-02	NaN	FOS

GDNF Family Ligand-Receptor Interactions	3,84E-01	2,94E-02	NaN	FOS,ITPR1
CD28 Signaling in T Helper Cells	3,81E-01	2,65E-02	NaN	FOS,VAV1,ITPR1
Acetone Degradation I (to Methylglyoxal)	3,77E-01	3,85E-02	NaN	CYP2J2
IL-6 Signaling	3,64E-01	2,59E-02	NaN	FOS,IL1A,IL18
Caveolar-mediated Endocytosis Signaling	3,62E-01	2,82E-02	NaN	ITGA11,ITGA7
p38 MAPK Signaling	3,59E-01	2,56E-02	NaN	IL1A,IL18,CREB5
phagosome maturation	3,54E-01	2,54E-02	NaN	LAMP2,ATP6V1G2,NOX1
TNFR2 Signaling	3,53E-01	3,57E-02	NaN	FOS
Intrinsic Prothrombin Activation Pathway	3,53E-01	3,57E-02	NaN	THBD
PPARα/RXRα Activation	3,5E-01	2,42E-02	NaN	PLCB4,PLCE1,APOA1,PLCB1
Ephrin B Signaling	3,49E-01	2,74E-02	NaN	KALRN,VAV1
Role of Pattern Recognition Receptors in Recognition of Bacteria and Viruses	3,48E-01	2,52E-02	NaN	IL1A,IL18,C3
RhoA Signaling	3,43E-01	2,5E-02	NaN	PPP1R12B,MYLPF,MYL7
Role of p14/p19ARF in Tumor Suppression	3,42E-01	3,45E-02	NaN	CDKN2A
Sonic Hedgehog Signaling	3,42E-01	3,45E-02	NaN	HHIP
cAMP-mediated signaling	3,33E-01	2,31E-02	-2,000	AKAP12,RGS2,ADRA2A,ADRA2C,CREB5
Retinol Biosynthesis	3,31E-01	3,33E-02	NaN	RBP1
Ethanol Degradation II	3,31E-01	3,33E-02	NaN	ALDH1A1
B Cell Receptor Signaling	3,24E-01	2,34E-02	-1,000	DAPP1,PAG1,VAV1,CREB5
Dopamine Receptor Signaling	3,23E-01	2,6E-02	NaN	MAOB,IL4I1
4-1BB Signaling in T Lymphocytes	3,21E-01	3,23E-02	NaN	TNFRSF9
Regulation of IL-2 Expression in Activated and Anergic T Lymphocytes	3,17E-01	2,56E-02	NaN	FOS,VAV1
Ceramide Signaling	3,05E-01	2,5E-02	NaN	FOS,SPHK1
Circadian Rhythm Signaling	3,02E-01	3,03E-02	NaN	CREB5
AMPK Signaling	3,01E-01	2,26E-02	NaN	ADRA2A,ADRA2C,CREB5,CHRNA3
Altered T Cell and B Cell Signaling in Rheumatoid Arthritis	2,99E-01	2,47E-02	NaN	IL1A,IL18

Communication between Innate and Adaptive Immune Cells	2,93E-01	2,44E-02	NaN	IL1A,IL18
Cell Cycle Regulation by BTG Family Proteins	2,84E-01	2,86E-02	NaN	HOXB9
α-Adrenergic Signaling	2,77E-01	2,35E-02	NaN	ADRA2A,ITPR1
autophagy	2,76E-01	2,78E-02	NaN	LAMP2
eNOS Signaling	2,74E-01	2,22E-02	NaN	AQP9,ITPR1,CHRNA3
Notch Signaling	2,68E-01	2,7E-02	NaN	HES1
Superpathway of Inositol Phosphate Compounds	2,68E-01	2,15E-02	NaN	PLCB4,PLCE1,PTPN13,PLCB1
ERK/MAPK Signaling	2,68E-01	2,15E-02	0,000	PPARG,FOS,ETS2,CREB5
RAR Activation	2,65E-01	2,14E-02	NaN	FOS,ALDH1A1,RBP1,MMP1
April Mediated Signaling	2,61E-01	2,63E-02	NaN	FOS
Crosstalk between Dendritic Cells and Natural Killer Cells	2,57E-01	2,25E-02	NaN	IL18,FSCN1
IL-1 Signaling	2,48E-01	2,2E-02	NaN	FOS,IL1A
B Cell Activating Factor Signaling	2,46E-01	2,5E-02	NaN	FOS
Neuroprotective Role of THOP1 in Alzheimer's Disease	2,46E-01	2,5E-02	NaN	MME
MIF Regulation of Innate Immunity	2,39E-01	2,44E-02	NaN	FOS
Mechanisms of Viral Exit from Host Cells	2,39E-01	2,44E-02	NaN	SH3GL3
Autoimmune Thyroid Disease Signaling	2,33E-01	2,38E-02	NaN	TG
Hematopoiesis from Pluripotent Stem Cells	2,33E-01	2,38E-02	NaN	IL1A
UVC-Induced MAPK Signaling	2,33E-01	2,38E-02	NaN	FOS
Role of Oct4 in Mammalian Embryonic Stem Cell Pluripotency	2,14E-01	2,22E-02	NaN	ETS2
TNFR1 Signaling	2,03E-01	2,13E-02	NaN	FOS
Ephrin A Signaling	1,98E-01	2,08E-02	NaN	VAV1

Supplementary Table S10. Canonical Pathways in Non-Epithelioid phenotype.

Ingenuity Canonical Pathways	-log(p-value)	Ratio	z-score	Molecules
Agranulocyte Adhesion and Diapedesis	7,77E00	1,77E-01	NaN	IL1A,MMP3,CLDN15,MMP15,SDC4,MMP24,CXCL10,HRH1,EZR,CXCL14,PODXL,ACTG2,MMP1,MMP17,ITGA4,ITGB1,ITGA2,MMP2,MYL7,IL33,CXCL16,ITGB2,IL18,MYH2,CLDN1,MYH3,IL1B,ITGA1,CLDN14,CXCL2,MMP9
Hepatic Fibrosis / Hepatic Stellate Cell Activation	7,41E00	1,71E-01	NaN	IGFBP4,COL8A2,IL1A,COL4A6,FGF2,PDGFA,COL8A1,IL6,BCL2,COL16A1,VEGFA,COL1A2,TGFB2,PDGFRA,KLF12,COL27A1,MMP1,PDGFRB,MMP2,BAX,MYL7,MYH2,COL6A3,COL13A1,MYH3,TGFB3,IL1B,KDR,PDGFD,MMP9,COL7A1
Human Embryonic Stem Cell Pluripotency	6,44E00	1,83E-01	NaN	NTF3,BMP4,KLK3,FZD3,FGF2,PDGFA,WNT2B,WNT6,FOXD3,INHBA,NTRK2,WNT10A,UTF1,PDGFRA,SPHK1,TGFB3,TGFB2,WNT4,BMP7,PIK3CD,PDGFD,WNT5B,PDGFRB,SALL4
Dopamine Degradation	5,79E00	4,09E-01	NaN	ALDH1B1,MAOB,ALDH3A2,SULT1A1,ALDH1A2,SULT1A3/SULT1A4,ALDH3B1,SULT1A2,IL4I1
Granulocyte Adhesion and Diapedesis	5,61E00	1,58E-01	NaN	IL1A,MMP3,CLDN15,MMP15,SDC4,MMP24,CXCL10,HRH1,EZR,CXCL14,MMP17,MMP1,ITGA4,ITGB1,ITGA2,MMP2,IL33,ITGB2,CXCL16,IL18,CLDN1,IL1B,ITGA1,CLDN14,CXCL2,MMP9
Axonal Guidance Signaling	5,56E00	1,15E-01	NaN	TUBA1B,ADAMTS7,NTF3,BMP4,PDGFA,FZD3,SEMA6B,WNT6,ABLIM1,PRKCZ,EPHB6,VEGFA,GNG11,NFAT5,PLCE1,SRGAP1,PLXNB1,WNT4,ABLIM2,SEMA3B,WNT5B,ADAMTS4,ITGA4,SEMA3E,ITGB1,PAPPA,PAK6,KALRN,ADAM15,MYLPF,SEMA5A,CHP1,ITGA2,WNT2B,MMP2,HHIP,EFNA1,MYL7,NTRK2,ADAMTS6,WNT10A,TUBB6,ADAM12,BMP7,PIK3CD,PDGFD,GLI1,MMP9,NRP1
Role of Osteoblasts, Osteoclasts and Chondrocytes in Rheumatoid Arthritis	5,3E00	1,4E-01	NaN	IL1A,BMP4,MMP3,FZD3,WNT6,IL6,BCL2,NFAT5,DKK3,WNT4,WNT5B,MMP1,ADAMTS4,ITGB1,CTSK,SPP1,CHP1,WNT2B,ITGA2,IL33,FOS,IL18,WNT10A,IL1B,BMP7,PIK3CD,DKK1,SFRP1,CSF2,IL11
Inhibition of Matrix Metalloproteases	4,41E00	2,63E-01	NaN	ADAM12,MMP3,MMP15,THBS2,MMP2,TFPI2,MMP17,MMP9,MMP24,MMP1
Regulation of the Epithelial-Mesenchymal Transition Pathway	4,37E00	1,37E-01	NaN	SNAI2,FZD3,FGF2,FGF9,SNAI1,WNT2B,PARD6B,WNT6,MMP2,HIF1A,FOXC2,CDH1,WNT10A,FGF18,ZEB2,TGFB3,TGFB2,WNT4,PIK3CD,PDGFD,JAK3,WNT5B,MMP9,HMGA2,PDGFRB
VDR/RXR Activation	3,98E00	1,82E-01	0,816	WT1,IGFBP6,SPP1,PDGFA,KLK6,THBD,PRKCZ,CXCL10,COL13A1,TGFB2,SEMA3B,VDR,CSF2,CST6

Leukocyte Extravasation Signaling	3,95E00	1,3E-01	0,218	MMP3,CLDN15,MMP15,MLLT4,NOX1,MMP24,PRKCZ,EZR,ACTG2,MMP1,MMP17,ITGA4,ITGB1,ITGA2,MMP2,ITGB2,EDIL3,CLDN1,CD44,PIK3CD,VAV1,ITGA1,CLDN14,ACTN4,MMP9
Colorectal Cancer Metastasis Signaling	3,84E00	1,21E-01	0,000	IL6ST,MMP3,FZD3,MMP15,WNT6,IL6,MMP24,VEGFA,GNG11,RHOB,TLR1,TGFB2,WNT4,WNT5B,MMP17,MMP1,WNT2B,MMP2,RHOJ,BAX,FOS,CDH1,WNT10A,TGFB3,PIK3CD,PTGS2,JAK3,MMP9
Complement System	3,83E00	2,5E-01	0,707	C4A/C4B,ITGB2,CFD,SERPING1,C3,C4BPA,CFB,CFI,CFH
Acute Phase Response Signaling	3,62E00	1,31E-01	0,277	IL6ST,SERPING1,ITIH3,IL1A,C3,IL6,NR3C1,RBP1,IL33,C4A/C4B,FOS,IL18,HP,FTL,APOA1,RBP7,C4BPA,CFB,IL1B,SOCS2,PIK3CD,RBP4
Putrescine Degradation III	3,61E00	3,53E-01	NaN	ALDH1B1,MAOB,ALDH3A2,ALDH1A2,ALDH3B1,IL4I1
RhoGDI Signaling	3,47E00	1,28E-01	0,655	ITGB1,PAK6,ARHGDIG,MYLPF,ITGA2,RHOJ,PIP5K1B,ARHGDIB,MYL7,ARHGEF5,CDH1,GNG11,PPP1R12B,RHOB,CDH3,EZR,CDH10,CD44,ARHGEF3,ACTG2,CDH13,ITGA4
Clathrin-mediated Endocytosis Signaling	3,46E00	1,25E-01	NaN	ITGB1,AP2M1,SH3BP4,STON2,F2R,FGF2,PDGFA,FGF9,SH3GL3,CHP1,VEGFA,ITGB2,ARRB2,LDLR,APOA1,FGF18,DAB2,PIK3CD,ITGB4,ACTG2,PDGFD,CLU,RBP4
Bladder Cancer Signaling	3,45E00	1,63E-01	NaN	DAPK1,MMP3,FGF2,FGF9,MMP15,MMP2,MMP24,VEGFA,CDH1,FGF18,THBS1,MMP9,MMP17,MMP1
Xenobiotic Metabolism Signaling	3,45E00	1,13E-01	NaN	IL1A,MAF,IL6,PRKCZ,HS3ST3A1,SULT1A2,MAOB,PPM1J,ALDH3A2,SULT1A3/SULT1A4,HS6ST2,HS3ST1,ALDH6A1,ALDH1B1,ABCB1,ALDH8A1,GSTO1,CYP1B1,IL4I1,HS3ST3B1,FTL,SULT1A1,ALDH1A2,IL1B,MAP3K8,ALDH3B1,PIK3CD,NRIP1,ABCC3
Tryptophan Degradation X (Mammalian, via Tryptamine)	3,45E00	3,33E-01	NaN	ALDH1B1,MAOB,ALDH3A2,ALDH1A2,ALDH3B1,IL4I1
Integrin Signaling	3,32E00	1,2E-01	0,000	ITGB1,RAP2A,CAPN6,RALA,TSPAN7,PAK6,ITGA2,RHOJ,MYL7,TSPAN3,ITGB2,PPP1R12B,RHOB,ITGA11,GRB7,CAV1,ITGA1,PIK3CD,ITGB4,ACTG2,ACTN4,ITGA7,NEDD9,ITGA4
Role of Macrophages, Fibroblasts and Endothelial Cells in Rheumatoid Arthritis	3,29E00	1,08E-01	NaN	IL6ST,IL1A,MMP3,FZD3,FGF2,PDGFA,CEBPD,WNT6,IL6,PRKCZ,VEGFA,PLCE1,NFAT5,DKK3,TLR1,WNT4,MMP1,WNT5B,ADAMTS4,CHP1,WNT2B,IL33,FOS,IL18,WNT10A,IL1B,PIK3CD,DKK1,SFRP1,PDGFD,CSF2
LXR/RXR Activation	3,28E00	1,4E-01	-3,000	IL1A,C3,VTN,ABCG1,IL6,IL33,C4A/C4B,HPR,IL18,APOA1,LDLR,IL1B,PTGS2,HADH,MMP9,CLU,RBP4
Atherosclerosis Signaling	3,28E00	1,4E-01	NaN	IL1A,MMP3,PDGFA,IL6,COL1A2,IL33,ITGB2,PLA2G4A,IL18,APOA1,IL1B,PDGFD,MMP9,CLU,MMP1,RBP4,ITGA4
Histamine Degradation	3,27E00	3,85E-01	NaN	ALDH1B1,HNMT,ALDH3A2,ALDH1A2,ALDH3B1

ILK Signaling	3,16E00	1,22E-01	-0,229	ITGB1,SNAI2,SNAI1,VIM,RHOJ,HIF1A,MYL7,VEGFA,FOS,ITGB2,CDH1,MYH2,RHOB,PPM1J,MYH3,KRT18,PIK3CD,ACTG2,ITGB4,PTGS2,ACTN4,MMP9
Aryl Hydrocarbon Receptor Signaling	3,16E00	1,33E-01	0,000	ALDH1B1,IL1A,ALDH8A1,IL6,BAX,CYP1B1,GSTO1,TGM2,FOS,CCND2,ALDH3A2,ALDH1A2,TGFB3,TGFB2,IL1B,ALDH3B1,NRIP1,ALDH6A1
Arsenate Detoxification I (Glutaredoxin)	3,15E00	7,5E-01	NaN	AS3MT,PNP,GSTO1
Phenylalanine Degradation IV (Mammalian, via Side Chain)	3,1E00	3,57E-01	NaN	MAOB,HPD,SLC27A2,ALDH3A2,IL4I1
Vitamin-C Transport	3,1E00	3,57E-01	NaN	SLC2A1,TXN,NXN,TXNRD1,GSTO1
LPS/IL-1 Mediated Inhibition of RXR Function	3,07E00	1,15E-01	NaN	ABCB1,ALDH1B1,IL1A,SLC27A2,ALDH8A1,ABCG1,GSTO1,HS3ST3A1,IL4I1,SULT1A2,IL33,HS3ST3B1,MAOB,IL18,SULT1A1,ALDH3A2,ALDH1A2,SULT1A3/SULT1A4,IL1B,HS6ST2,ALDH3B1,HS3ST1,ABCC3,ALDH6A1
Signaling by Rho Family GTPases	3,06E00	1,12E-01	-1,225	ITGB1,PAK6,MYLPF,CDC42EP5,ITGA2,VIM,RHOJ,PIP5K1B,DES,NOX1,PRKCZ,MYL7,ARHGEF5,FOS,CDH1,GNG11,PPP1R12B,RHOB,CDH3,EZR,CDH10,PIK3CD,ARHGEF3,ACTG2,CDH13,ITGA4
Oxidative Ethanol Degradation III	2,95E00	3,33E-01	NaN	ALDH1B1,ACSS3,ALDH3A2,ALDH1A2,ALDH3B1
Fatty Acid α-oxidation	2,81E00	3,12E-01	NaN	ALDH1B1,ALDH3A2,ALDH1A2,ALDH3B1,PTGS2
HIF1α Signaling	2,79E00	1,4E-01	NaN	SLC2A5,SLC2A1,MMP3,MMP15,MMP2,HIF1A,MMP24,P4HTM,VEGFA,EGLN3,PIK3CD,MMP1,MMP9,MMP17
IL-8 Signaling	2,73E00	1,15E-01	-0,218	ANGPT1,MMP2,RHOJ,BAX,NOX1,PRKCZ,MYL7,BCL2,VEGFA,FOS,ITGB2,ARRB2,CDH1,CCND2,GNG11,RHOB,PIK3CD,PTGS2,KDR,MMP9,TEK
Chondroitin Sulfate Biosynthesis (Late Stages)	2,5E00	1,82E-01	NaN	HS3ST3B1,CHSY3,SULT1A1,SULT1A3/SULT1A4,HS6ST2,HS3ST1,HS3ST3A1,SULT1A2
Guanine and Guanosine Salvage I	2,49E00	1E00	NaN	PNP,HPRT1
Thioredoxin Pathway	2,48E00	5E-01	NaN	TXN,NXN,TXNRD1
Ethanol Degradation IV	2,44E00	2,63E-01	NaN	ALDH1B1,ACSS3,ALDH3A2,ALDH1A2,ALDH3B1
Role of NANOG in Mammalian Embryonic Stem Cell Pluripotency	2,43E00	1,28E-01	-1,633	IL6ST,BMP4,FZD3,WNT2B,WNT6,FOXD3,WNT10A,WNT4,PIK3CD,BMP7,JAK3,WNT5B,SALL4,ZFP42
Paxillin Signaling	2,42E00	1,33E-01	0,000	ITGB1,ITGB2,PAK6,ITGA11,ITGA2,ITGA1,PIK3CD,ACTN4,ACTG2,ITGB4,ITGA7,PTPN12,ITGA4
Serotonin Degradation	2,37E00	1,61E-01	NaN	ALDH1B1,MAOB,ALDH3A2,SULT1A1,ALDH1A2,SULT1A3/SULT1A4,ALDH3B1,SULT1A2,IL4I1

PDGF Signaling	2,37E00	1,43E-01	-0,905	FOS,PDGFA,PDGFRA,CAV1,SPHK1,PIK3CD,PDGFD,JAK3,CAV3,INPP5D,PDGFRB
Actin Cytoskeleton Signaling	2,34E00	1,05E-01	-1,000	ITGB1,F2R,PAK6,FGF2,PDGFA,FGF9,MYLPF,ITGA2,PIP5K1B,MYL7,MYH2,PPP1R12B,CYFIP2,FGF18,EZR,MYH3,VAV1,PIK3CD,ACTG2,ACTN4,PDGFD,ITGA4
Virus Entry via Endocytic Pathways	2,33E00	1,35E-01	NaN	ITGB1,ITGB2,AP2M1,ITGA2,CAV1,ITGA1,PIK3CD,ITGB4,ACTG2,CXADR,PRKCZ,ITGA4
Glioma Invasiveness Signaling	2,32E00	1,58E-01	1,000	F2R,RHOB,VTN,CD44,RHOJ,MMP2,PIK3CD,PLAU,MMP9
Molecular Mechanisms of Cancer	2,3E00	9,19E-02	NaN	RAP2A,BMP4,RALA,FZD3,WNT6,HIF1A,CDKN2B,PRKCZ,BCL2,HHAT,RHOB,TGFB2,WNT4,ARHGEF3,WNT5B,ITGA4,ITGB1,PAK6,WNT2B,ITGA2,RHOJ,BAX,FOS,ARHGEF5,CDH1,CCND2,WNT10A,TGFB3,BMP7,PIK3CD,CFLAR,JAK3,GLI1
phagosome formation	2,27E00	1,27E-01	NaN	ITGB1,VTN,ITGA2,RHOJ,INPP5D,PRKCZ,PLCE1,SCARA3,RHOB,TLR1,PIK3CD,MARCO,ITGA4
Basal Cell Carcinoma Signaling	2,25E00	1,45E-01	-1,897	BMP4,WNT10A,FZD3,WNT2B,WNT4,WNT6,BMP7,HHIP,GLI1,WNT5B
Retinoate Biosynthesis I	2,2E00	2E-01	NaN	RBP7,RDH10,ALDH1A2,ALDH8A1,RDH13,RBP1
Wnt/β-catenin Signaling	2,16E00	1,08E-01	-0,535	FZD3,WNT2B,TLE1,WNT6,CDH1,WNT10A,DKK3,CDH3,PPM1J,TGFB2,TGFB3,CD44,WNT4,SOX18,SFRP1,DKK1,WNT5B,SOX3
Caveolar-mediated Endocytosis Signaling	2,16E00	1,41E-01	NaN	ITGB1,ITGB2,ITGA11,ITGA2,CAV1,ITGA1,ITGB4,ACTG2,ITGA7,ITGA4
Role of Pattern Recognition Receptors in Recognition of Bacteria and Viruses	2,09E00	1,18E-01	-0,378	PTX3,IL18,IL1A,NLRP3,C3,TLR1,TGFB3,TGFB2,IL1B,PIK3CD,IL6,CSF2,PRKCZ,IL11
Airway Pathology in Chronic Obstructive Pulmonary Disease	2,07E00	3,75E-01	NaN	MMP2,MMP9,MMP1
Chondroitin Sulfate Biosynthesis	2,05E00	1,54E-01	NaN	HS3ST3B1,CHSY3,SULT1A1,SULT1A3/SULT1A4,HS6ST2,HS3ST1,HS3ST3A1,SULT1A2
Dermatan Sulfate Biosynthesis (Late Stages)	2,04E00	1,67E-01	NaN	HS3ST3B1,SULT1A1,SULT1A3/SULT1A4,HS6ST2,HS3ST1,HS3ST3A1,SULT1A2
p53 Signaling	2E00	1,22E-01	-0,333	WT1,CCND2,SNAI2,THBS1,ADCK3,ADGRB1,RPRM,PIK3CD,HIF1A,BAX,BCL2,SERPINE2
Noradrenaline and Adrenaline Degradation	1,99E00	1,82E-01	NaN	ALDH1B1,MAOB,ALDH3A2,ALDH1A2,ALDH3B1,IL4I1
Regulation of Actin-based Motility by Rho	1,97E00	1,26E-01	0,302	ITGB1,PPP1R12B,PAK6,RHOB,MYLPF,ITGA2,RHOJ,ACTG2,PIP5K1B,MYL7,ITGA4
PAK Signaling	1,94E00	1,25E-01	-0,302	ITGB1,PAK6,PDGFA,MYLPF,ITGA2,PDGFRA,PIK3CD,PDGFD,PDGFRB,MYL7,ITGA4
Oncostatin M Signaling	1,93E00	1,76E-01	0,816	MT2A,IL6ST,MMP3,PLAU,JAK3,MMP1

Dermatan Sulfate Biosynthesis	1,91E00	1,45E-01	NaN	HS3ST3B1,CHSY3,SULT1A1,SULT1A3/SULT1A4,HS6ST2,HS3ST1,HS3ST3A1,SULT1A2
PPAR Signaling	1,87E00	1,22E-01	-0,905	IL33,FOS,IL1A,IL18,PDGFA,PDGFRA,IL1B,PTGS2,NRIP1,PDGFD,PDGFRB
MSP-RON Signaling Pathway	1,83E00	1,52E-01	NaN	ITGB2,KLK3,MST1,PIK3CD,ACTG2,MST1R,PRKCZ
nNOS Signaling in Neurons	1,83E00	1,52E-01	-2,000	GRIN1,CAPN6,GRIN2D,CHP1,DLG2,RASD1,PRKCZ
Ovarian Cancer Signaling	1,78E00	1,08E-01	NaN	FZD3,FGF9,WNT2B,MMP2,WNT6,BCL2,VEGFA,WNT10A,CD44,WNT4,PIK3CD,PTGS2,WNT5B,MMP9
Germ Cell-Sertoli Cell Junction Signaling	1,77E00	1,03E-01	NaN	ITGB1,TUBA1B,PAK6,ITGA2,MLLT4,RHOJ,CDH1,RHOB,TUBB6,TGFB3,TGFB2,MAP3K8,PIK3CD,ACTG2,JUP,ACTN4
HMGB1 Signaling	1,76E00	1,1E-01	1,387	HMGB1,FOS,IL18,IL1A,RHOB,TGFB2,TGFB3,IL1B,PIK3CD,RHOJ,IL6,CSF2,IL11
PTEN Signaling	1,76E00	1,1E-01	0,832	ITGB1,ITGA2,DDR1,PRKCZ,INPP5D,BCL2,NTRK2,GHR,PDGFRA,PIK3CD,KDR,PDGFRB,ITGA4
TREM1 Signaling	1,75E00	1,29E-01	1,000	ITGB1,TREM1,IL18,NLRP3,TLR1,IL1B,IL6,NLRP2,CSF2
Retinoate Biosynthesis II	1,74E00	5E-01	NaN	RBP7,RBP1
Melatonin Degradation II	1,74E00	5E-01	NaN	MAOB,IL4I1
NAD Biosynthesis III	1,74E00	5E-01	NaN	NMNAT3,NAMPT
Heparan Sulfate Biosynthesis (Late Stages)	1,73E00	1,46E-01	NaN	HS3ST3B1,SULT1A1,SULT1A3/SULT1A4,HS6ST2,HS3ST1,HS3ST3A1,SULT1A2
Glioblastoma Multiforme Signaling	1,72E00	1,03E-01	-1,807	PLCE1,WNT10A,RHOB,FZD3,PDGFA,WNT2B,PDGFRA,WNT4,PIK3CD,WNT6,RHOJ,ITPR1,PDGFD,WNT5B,PDGFRB
Sertoli Cell-Sertoli Cell Junction Signaling	1,68E00	9,83E-02	NaN	ITGB1,TUBA1B,CLDN15,ITGA2,MLLT4,CDH1,TUBB6,CLDN1,CGN,TGFB3,CLDN14,MAP3K8,ACTG2,JUP,SPTAN1,ACTN4,ITGA4
Thyroid Cancer Signaling	1,65E00	1,54E-01	NaN	CXCL10,NTF3,CDH1,NTRK2,KLK3,GDNF
Amyotrophic Lateral Sclerosis Signaling	1,65E00	1,13E-01	NaN	PRPH,VEGFA,GRIN1,CAPN6,CACNA1E,GDNF,GRIN2D,NEFM,PIK3CD,BAX,BCL2
STAT3 Pathway	1,64E00	1,23E-01	-1,000	PTPN6,GHR,NTRK2,PDGFRA,SOCS2,KDR,DDR1,PDGFRB,BCL2
PCP pathway	1,61E00	1,29E-01	-1,134	WNT10A,FZD3,WNT2B,CTHRC1,WNT4,WNT6,SDC4,WNT5B
Phospholipase C Signaling	1,61E00	9,09E-02	-0,471	ITGB1,RALA,MYLPF,CHP1,ITGA2,RPS6KA3,CD79A,RHOJ,ITPR1,PRKCZ,MYL7,TGM2,PLA2G4A,ARHGEF5,PLCE1,GNG11,NFAT5,PPP1R12B,RHOB,ARHGEF3,ITGA4
Ethanol Degradation II	1,58E00	1,67E-01	NaN	ALDH1B1,ACSS3,ALDH3A2,ALDH1A2,ALDH3B1
TGF-β Signaling	1,58E00	1,15E-01	0,447	FOS,BMP4,TGFB3,TGFB2,BMP7,VDR,PITX2,INHBA,AMHR2,BCL2
FXR/RXR Activation	1,57E00	1,04E-01	NaN	C4A/C4B,IL33,IL18,HPR,IL1A,APOA1,C3,VTN,FBP1,IL1B,VLDLR,CLU,RBP4

Semaphorin Signaling in Neurons	1,56E00	1,35E-01	NaN	ITGB1,PAK6,RHOB,DPYSL4,PLXNB1,RHOJ,NRP1
Role of Hypercytokinemia/hyperchemokinemia in the Pathogenesis of Influenza	1,55E00	1,46E-01	NaN	IL33,CXCL10,IL1A,IL18,IL1B,IL6
Role of IL-17F in Allergic Inflammatory Airway Diseases	1,55E00	1,46E-01	1,000	CXCL10,RPS6KA3,IL1B,IL6,CSF2,IL11
Creatine-phosphate Biosynthesis	1,54E00	4E-01	NaN	CKB,CKMT1A/CKMT1B
Cdc42 Signaling	1,48E00	1,01E-01	0,905	ITGB1,RALA,MYLPF,CDC42EP5,ITGA2,PRKCZ,MYL7,FOS,CDC42BPA,PPP1R12B,VAV1,HLA-DPA1,ITGA4
Guanosine Nucleotides Degradation III	1,46E00	2,31E-01	NaN	NT5E,PNP,AOX1
Epithelial Adherens Junction Signaling	1,46E00	9,79E-02	NaN	TUBA1B,SNAI2,LMO7,SNAI1,MLLT4,MYL7,CDH1,MYH2,TUBB6,MYH3,TGFB2,ACTG2,JUP,ACTN4
Reelin Signaling in Neurons	1,44E00	1,14E-01	NaN	ITGB1,ITGB2,ARHGEF5,ITGA2,ITGA1,PIK3CD,ARHGEF3,VLDLR,ITGA4
Heparan Sulfate Biosynthesis	1,44E00	1,27E-01	NaN	HS3ST3B1,SULT1A1,SULT1A3/SULT1A4,HS6ST2,HS3ST1,HS3ST3A1,SULT1A2
Superpathway of Melatonin Degradation	1,44E00	1,27E-01	NaN	MAOB,SULT1A1,SULT1A3/SULT1A4,CYP2J2,CYP1B1,SULT1A2,IL4I1
Agrin Interactions at Neuromuscular Junction	1,43E00	1,19E-01	0,707	ITGB1,ITGB2,PAK6,ITGA2,ITGA1,ACTG2,AGRN,ITGA4
IL-12 Signaling and Production in Macrophages	1,43E00	9,92E-02	NaN	FOS,IL18,APOA1,MAF,TGFB2,TGFB3,MST1,MAP3K8,PIK3CD,MST1R,CLU,PRKCZ,RBP4
Macropinocytosis Signaling	1,4E00	1,18E-01	NaN	ITGB1,ITGB2,PDGFA,PIK3CD,ITGB4,ACTN4,PDGFD,PRKCZ
Altered T Cell and B Cell Signaling in Rheumatoid Arthritis	1,38E00	1,11E-01	NaN	IL33,IL1A,IL18,SPP1,TLR1,IL1B,CD79A,IL6,CSF2
Adenine and Adenosine Salvage III	1,38E00	3,33E-01	NaN	PNP,HPRT1
Urate Biosynthesis/Inosine 5'-phosphate Degradation	1,37E00	2,14E-01	NaN	NT5E,PNP,AOX1
Growth Hormone Signaling	1,37E00	1,16E-01	-0,707	FOS,PTPN6,GHR,IGFALS,RPS6KA3,SOCS2,PIK3CD,PRKCZ
PI3K/AKT Signaling	1,35E00	9,92E-02	0,302	ITGB1,PPM1J,GDF15,ITGA2,MAP3K8,PIK3CD,PTGS2,JAK3,INPP5D,PRKCZ,ITGA4,BCL2
Glycolysis I	1,34E00	1,67E-01	NaN	TPI1,FBP1,ALDOA,PFKP
PI3K Signaling in B Lymphocytes	1,31E00	9,76E-02	-1,732	FOS,NFAT5,PLCE1,C3,DAPP1,CHP1,VAV1,CD79A,PIK3CD,ITPR1,INPP5D,PRKCZ
The Visual Cycle	1,29E00	2E-01	NaN	RBP7,RDH10,RBP1

Tight Junction Signaling	1,27E00	9,04E-02	NaN	CLDN15,MLLT4,PRKCZ,MYL7,FOS,MYH2,CLDN1,PPM1J,CGN,TGFB2,TGFB3,MYH3,CLDN14,ACTG2,SPTAN1
TR/RXR Activation	1,27E00	1,06E-01	NaN	HP,LDLR,COL6A3,SLC2A1,TRH,PIK3CD,PFKP,HIF1A,RCAN2
Neuropathic Pain Signaling In Dorsal Horn Neurons	1,25E00	1,01E-01	-1,897	GRIN1,FOS,KCNN4,PLCE1,NTRK2,GRIN2D,PIK3CD,ITPR1,KCNH2,PRKCZ
Xanthine and Xanthosine Salvage	1,24E00	1E00	NaN	PNP
Adenosine Nucleotides Degradation II	1,22E00	1,88E-01	NaN	NT5E,PNP,AOX1
NF-κB Signaling	1,22E00	8,88E-02	-1,807	IL1A,BMP4,PRKCZ,DDR1,IL33,IL18,GHR,NTRK2,TLR1,PDGFRA,IL1B,MAP3K8,PIK3CD,KDR,PDGFRB
Tec Kinase Signaling	1,22E00	9,03E-02	-0,277	ITGB1,TNFRSF21,FOS,GNG11,RHOB,PAK6,ITGA2,VAV1,PIK3CD,RHOJ,ACTG2,JAK3,PRKCZ,ITGA4
Cholecystokinin/Gastrin-mediated Signaling	1,2E00	9,9E-02	0,632	IL33,FOS,IL1A,IL18,RHOB,IL1B,RHOJ,PTGS2,ITPR1,PRKCZ
Glucocorticoid Receptor Signaling	1,19E00	8,09E-02	NaN	CHP1,HSPA1A/HSPA1B,SLPI,IL6,KRT32,NR3C1,HSPA2,SMARCD3,BCL2,HMGB1,FOS,NFAT5,TGFB3,TGFB2,IL1B,PIK3CD,NRIP1,PTGS2,PLAU,CSF2,JAK3,MMP1
IL-15 Production	1,18E00	1,48E-01	NaN	IL6,MST1R,JAK3,PRKCZ
IL-6 Signaling	1,17E00	9,48E-02	0,905	IL33,VEGFA,IL6ST,ABCB1,FOS,IL1A,IL18,TNFAIP6,IL1B,PIK3CD,IL6
Rac Signaling	1,15E00	9,71E-02	-0,333	ITGB1,CYFIP2,PAK6,ITGA2,CD44,PIK3CD,PIP5K1B,PRKCZ,NOX1,ITGA4
Gα12/13 Signaling	1,14E00	9,4E-02	-0,905	CDH1,F2R,LPAR2,CDH3,MYLPF,CDH10,LPAR5,VAV1,PIK3CD,CDH13,MYL7
Gαq Signaling	1,14E00	8,97E-02	-0,832	HTR2B,CHP1,RGS16,RGS4,RHOJ,ITPR1,PRKCZ,HRH1,GNG11,RHOB,PIK3CD,ARHGEF25,ADRA1B
Sucrose Degradation V (Mammalian)	1,14E00	2,5E-01	NaN	TPI1,ALDOA
Glucocorticoid Biosynthesis	1,14E00	2,5E-01	NaN	CYP17A1,HSD3B1
Role of Cytokines in Mediating Communication between Immune Cells	1,12E00	1,15E-01	NaN	IL33,IL1A,IL18,IL1B,IL6,CSF2
Differential Regulation of Cytokine Production in Macrophages and T Helper Cells by IL-17A and IL-17F	1,1E00	1,67E-01	NaN	IL1B,IL6,CSF2
Pancreatic Adenocarcinoma Signaling	1,09E00	9,43E-02	0,000	VEGFA,RALA,TGFB3,TGFB2,PIK3CD,PTGS2,JAK3,CDKN2B,MMP9,BCL2

eNOS Signaling	1,07E00	8,89E-02	-1,265	VEGFA,LPAR2,HSPA1A/HSPA1B,LPAR5,CAV1,AQP1,PIK3CD,ITPR1,KDR,HSPA2,CHRNA3,PRKCZ
Mouse Embryonic Stem Cell Pluripotency	1,05E00	9,57E-02	-3,000	IL6ST,BMP4,FZD3,PIK3CD,FOXD3,JAK3,ID3,ID4,ZFP42
Sphingosine-1-phosphate Signaling	1,05E00	9,26E-02	-0,632	PLCE1,RHOB,PDGFA,PDGFRA,SPHK1,RHOJ,PIK3CD,PDGFD,SMPD3,PDGFRB
Prostanoid Biosynthesis	1,05E00	2,22E-01	NaN	PTGS2,TBXAS1
Folate Transformations I	1,05E00	2,22E-01	NaN	MTHFD2,MTR
Purine Nucleotides Degradation II (Aerobic)	1,04E00	1,58E-01	NaN	NT5E,PNP,AOX1
Regulation of Cellular Mechanics by Calpain Protease	1,03E00	1,09E-01	1,000	ITGB1,CAPN6,EZR,ITGA2,ACTN4,ITGA4
GDNF Family Ligand-Receptor Interactions	1,03E00	1,03E-01	-1,633	FOS,GFRA4,ARTN,GDNF,DOK7,PIK3CD,ITPR1
Antioxidant Action of Vitamin C	1,03E00	9,47E-02	NaN	SLC2A5,PLA2G4A,PLCE1,SLC2A1,TXN,NXN,CSF2,TXNRD1,GSTO1
Communication between Innate and Adaptive Immune Cells	1,02E00	9,76E-02	NaN	IL33,CXCL10,IL1A,IL18,TLR1,IL1B,IL6,CSF2
Cellular Effects of Sildenafil (Viagra)	1,01E00	8,87E-02	NaN	KCNN4,CACNA1E,PLCE1,MYH2,PPP1R12B,MYLPF,MYH3,ACTG2,ITPR1,KCNH2,MYL7
Renal Cell Carcinoma Signaling	1,01E00	1,01E-01	NaN	VEGFA,FOS,PAK6,SLC2A1,EGLN3,PIK3CD,HIF1A
Actin Nucleation by ARP-WASP Complex	1E00	1,07E-01	1,633	ITGB1,PPP1R12B,RHOB,ITGA2,RHOJ,ITGA4
Graft-versus-Host Disease Signaling	9,79E-01	1,14E-01	NaN	IL33,IL1A,IL18,IL1B,IL6
Inhibition of Angiogenesis by TSP1	9,67E-01	1,25E-01	0,000	VEGFA,THBS1,KDR,MMP9
Oleate Biosynthesis II (Animals)	9,65E-01	2E-01	NaN	SCD5,ALDH6A1
β-alanine Degradation I	9,54E-01	5E-01	NaN	ALDH6A1
Anandamide Degradation	9,54E-01	5E-01	NaN	FAAH2
Adenine and Adenosine Salvage I	9,54E-01	5E-01	NaN	PNP
GDP-L-fucose Biosynthesis I (from GDP-D-mannose)	9,54E-01	5E-01	NaN	GMDS
HIPPO signaling	9,48E-01	9,41E-02	1,342	WWC1,PPM1J,PPP1R3C,DLG2,CD44,MST1,FRMD6,PRKCZ
Superpathway of Inositol Phosphate Compounds	9,45E-01	8,06E-02	NaN	PTPN6,DUSP8,EPHX2,PPFIBP2,PIP5K1B,PTPN12,PXYLP1,INPP5D,PLCE1,TMEM55A,CILP,PIK3CD,PPIP5K2,PTPN22,PPFIA4
NAD Salvage Pathway II	9,38E-01	1,43E-01	NaN	NT5E,NMNAT3,PXYLP1

Ephrin Receptor Signaling	9,33E-01	8,14E-02	-0,378	ITGB1,GRIN1,KALRN,ANGPT1,PAK6,PDGFA,GRIN2D,ITGA2,EFNA1,VEGFA,EPHB6,GNG11,PDGFD,ITGA4
RAR Activation	9,31E-01	8,02E-02	NaN	RDH10,RBP1,PRKCZ,SMARCD3,VEGFA,FOS,RBP7,ALDH1A2,TGFB2,TGFB3,PIK3CD,NRIP1,RDH13,MMP1,RBP4
Circadian Rhythm Signaling	9,3E-01	1,21E-01	NaN	AVP,GRIN1,VIPR2,GRIN2D
FAK Signaling	9,26E-01	9,3E-02	NaN	ITGB1,CAPN6,PAK6,ITGA2,PIK3CD,ACTG2,TNS1,ITGA4
Hepatic Cholestasis	9,22E-01	8,23E-02	NaN	IL33,ABCB1,IL18,CYP7B1,IL1A,TGFB3,TGFB2,IL1B,IL6,ABCC3,CSF2,PRKCZ,IL11
NF-κB Activation by Viruses	9,09E-01	9,59E-02	NaN	ITGB1,ITGB2,ITGA2,ITGA1,PIK3CD,PRKCZ,ITGA4
Adipogenesis pathway	8,69E-01	8,33E-02	NaN	CTNNBIP1,FOXC2,BMP4,EGR2,FGF2,FZD3,TXNIP,CEBPD,BMP7,HIF1A,RBP1
VEGF Signaling	8,65E-01	8,99E-02	0,000	VEGFA,PTPN6,PIK3CD,HIF1A,ACTG2,ACTN4,KDR,BCL2
Coagulation System	8,61E-01	1,14E-01	0,000	F2R,PLAU,TFPI,THBD
IL-17A Signaling in Fibroblasts	8,61E-01	1,14E-01	NaN	FOS,CEBPD,IL6,MMP1
HGF Signaling	8,51E-01	8,65E-02	0,707	ITGB1,FOS,ITGA2,MAP3K8,PIK3CD,PTGS2,IL6,PRKCZ,ITGA4
Differential Regulation of Cytokine Production in Intestinal Epithelial Cells by IL-17A and IL-17F	8,49E-01	1,3E-01	NaN	IL1A,IL1B,CSF2
HER-2 Signaling in Breast Cancer	8,43E-01	9,21E-02	NaN	ITGB1,ITGB2,PARD6B,MMP2,PIK3CD,ITGB4,PRKCZ
VEGF Family Ligand-Receptor Interactions	8,43E-01	9,21E-02	0,000	VEGFA,PLA2G4A,FOS,PIK3CD,KDR,PRKCZ,NRP1
Androgen Biosynthesis	8,31E-01	1,67E-01	NaN	CYP17A1,HSD3B1
Interferon Signaling	8,29E-01	1,11E-01	-1,000	MX1,IFI6,BAX,BCL2
Melatonin Degradation I	8,06E-01	1E-01	NaN	SULT1A1,SULT1A3/SULT1A4,CYP2J2,CYP1B1,SULT1A2
Role of Tissue Factor in Cancer	7,99E-01	8,41E-02	NaN	ITGB1,VEGFA,PDIA2,ARRB2,RPS6KA3,IL1B,PIK3CD,CSF2,MMP1
Diphthamide Biosynthesis	7,9E-01	3,33E-01	NaN	EEF2
Methylglyoxal Degradation I	7,9E-01	3,33E-01	NaN	HAGHL
Ascorbate Recycling (Cytosolic)	7,9E-01	3,33E-01	NaN	GSTO1
Methionine Salvage II (Mammalian)	7,9E-01	3,33E-01	NaN	MTR
N-acetylglucosamine Degradation I	7,9E-01	3,33E-01	NaN	GNPDA2

Protein Kinase A Signaling	7,88E-01	7,03E-02	-0,816	DUSP8,PPP1R3C,DUSP6,DUSP15,PTPN12,PRKCZ,HHAT,NFAT5,GNG11,PLCE1,TGFB2,PTPRG,PTPN6,PTPRK,MYLPF,CHP1,ITPR1,MYL7,PYGM,MYH2,PTPRB,TGFB3,PTGS2,PTPRR,KDELR2,PTPN22
D-myo-inositol-5-phosphate Metabolism	7,77E-01	7,97E-02	NaN	PTPN6,PLCE1,DUSP8,TMEM55A,EPHX2,PPFIBP2,CILP,PTPN12,PXYLP1,PTPN22,PPFIA4
NAD biosynthesis II (from tryptophan)	7,74E-01	1,54E-01	NaN	TDO2,NMNAT3
Cholesterol Biosynthesis I	7,74E-01	1,54E-01	NaN	DHCR24,TM7SF2
Cholesterol Biosynthesis II (via 24,25-dihydrolanosterol)	7,74E-01	1,54E-01	NaN	DHCR24,TM7SF2
Cholesterol Biosynthesis III (via Desmosterol)	7,74E-01	1,54E-01	NaN	DHCR24,TM7SF2
3-phosphoinositide Degradation	7,63E-01	7,91E-02	NaN	PTPN6,DUSP8,TMEM55A,EPHX2,PPFIBP2,CILP,PTPN12,PXYLP1,INPP5D,PTPN22,PPFIA4
Ceramide Signaling	7,62E-01	8,75E-02	0,816	FOS,PPM1J,SPHK1,PIK3CD,SMPD3,PRKCZ,BCL2
Remodeling of Epithelial Adherens Junctions	7,56E-01	9,09E-02	NaN	TUBA1B,CDH1,RALA,TUBB6,ACTG2,ACTN4
Docosahexaenoic Acid (DHA) Signaling	7,4E-01	1,03E-01	NaN	IL1B,PIK3CD,BAX,BCL2
Netrin Signaling	7,4E-01	1,03E-01	NaN	NFAT5,CHP1,ABLIM2,ABLIM1
Neurotrophin/TRK Signaling	7,36E-01	8,96E-02	0,447	FOS,NTF3,NTRK2,KLK3,SPRY1,PIK3CD
Thrombin Signaling	7,34E-01	7,49E-02	-1,941	GATA5,F2R,MYLPF,RHOJ,ITPR1,PRKCZ,MYL7,ARHGEF5,GNG11,PLCE1,PPP1R12B,RHOB,PIK3CD,ARHGEF3
Unfolded protein response	7,33E-01	9,43E-02	NaN	PDIA2,HSPA1A/HSPA1B,CEBPD,HSPA2,BCL2
IL-10 Signaling	7,15E-01	8,82E-02	NaN	IL33,FOS,IL1A,IL18,IL1B,IL6
Cardiac Hypertrophy Signaling	7,12E-01	7,27E-02	-2,000	MYLPF,CHP1,RHOJ,IL6,MYL7,CACNA1E,GNG11,PLCE1,RHOB,ADRA2A,TGFB2,TGFB3,MAP3K8,ADRA2C,PIK3CD,ADRA1B
CDK5 Signaling	7,03E-01	8,16E-02	-0,816	ITGB1,FOSB,LAMA5,NTRK2,PPM1J,CABLES1,PPP1R3C,ITGA2
Role of NFAT in Cardiac Hypertrophy	6,81E-01	7,39E-02	-0,277	IL6ST,RCAN1,GNG11,PLCE1,CHP1,TGFB3,TGFB2,PIK3CD,ITPR1,IL6,RCAN2,PRKCZ,IL11
Phenylethylamine Degradation I	6,78E-01	2,5E-01	NaN	ALDH3A2
α-tocopherol Degradation	6,78E-01	2,5E-01	NaN	CYP4F2
Myo-inositol Biosynthesis	6,78E-01	2,5E-01	NaN	IMPA2
N-acetylglucosamine Degradation II	6,78E-01	2,5E-01	NaN	GNPDA2
Acetate Conversion to Acetyl-CoA	6,78E-01	2,5E-01	NaN	ACSS3

Intrinsic Prothrombin Activation Pathway	6,7E-01	1,07E-01	NaN	COL1A2,KLK3,THBD
Hematopoiesis from Pluripotent Stem Cells	6,62E-01	9,52E-02	NaN	IL1A,IL6,CSF2,IL11
IL-3 Signaling	6,58E-01	8,45E-02	0,000	FOS,PTPN6,CHP1,PIK3CD,INPP5D,PRKCZ
Neuregulin Signaling	6,55E-01	8,14E-02	0,000	ITGB1,DCN,GRB7,ITGA2,ERRFI1,PRKCZ,ITGA4
Production of Nitric Oxide and Reactive Oxygen Species in Macrophages	6,46E-01	7,26E-02	-1,732	FOS,PTPN6,APOA1,RHOB,PPM1J,PPP1R3C,MAP3K8,PIK3CD,RHOJ,JAK3,CLU,PRKCZ,RBP4
Thyroid Hormone Metabolism II (via Conjugation and/or Degradation)	6,4E-01	1,03E-01	NaN	SULT1A1,SULT1A3/SULT1A4,SULT1A2
Sonic Hedgehog Signaling	6,4E-01	1,03E-01	NaN	ARRB2,HHIP,GLI1
Role of Wnt/GSK-3β Signaling in the Pathogenesis of Influenza	6,4E-01	8,33E-02	-1,633	WNT10A,FZD3,WNT2B,WNT4,WNT6,WNT5B
JAK/Stat Signaling	6,4E-01	8,33E-02	0,816	FOS,PTPN6,SOCS2,PIK3CD,IL6,JAK3
IL-4 Signaling	6,4E-01	8,33E-02	NaN	PTPN6,NFAT5,PIK3CD,JAK3,NR3C1,INPP5D
Extrinsic Prothrombin Activation Pathway	6,34E-01	1,25E-01	NaN	TFPI,THBD
3-phosphoinositide Biosynthesis	6,31E-01	7,38E-02	NaN	PTPN6,DUSP8,EPHX2,PPFIBP2,CILP,PIK3CD,PIP5K1B,PTPN12,PXYLP1,PTPN22,PPFIA4
Toll-like Receptor Signaling	6,22E-01	8,22E-02	1,000	IL33,FOS,IL1A,IL18,TLR1,IL1B
Retinol Biosynthesis	6,11E-01	1E-01	NaN	RBP7,RDH10,RBP1
Aldosterone Signaling in Epithelial Cells	6,07E-01	7,28E-02	-2,236	CRYAB,PLCE1,DNAJC9,HSPA1A/HSPA1B,HSPB9,HSPB8,PIK3CD,PIP5K1B,ITPR1,HSPA2,PRKCZ
Factors Promoting Cardiogenesis in Vertebrates	6,07E-01	7,87E-02	NaN	BMP4,FZD3,TGFB3,TGFB2,BMP7,DKK1,PRKCZ
RhoA Signaling	6,04E-01	7,5E-02	-1,667	PPP1R12B,LPAR2,MYLPF,CDC42EP5,EZR,LPAR5,ACTG2,PIP5K1B,MYL7
Tetrahydrofolate Salvage from 5,10-methenyltetrahydrofolate	5,93E-01	2E-01	NaN	MTHFD2
Serine Biosynthesis	5,93E-01	2E-01	NaN	PSAT1
NAD Salvage Pathway III	5,93E-01	2E-01	NaN	NMNAT3
Citrulline-Nitric Oxide Cycle	5,93E-01	2E-01	NaN	ASS1
Tyrosine Degradation I	5,93E-01	2E-01	NaN	HPD

Role of Oct4 in Mammalian Embryonic Stem Cell Pluripotency	5,93E-01	8,89E-02	NaN	SPP1,UTF1,FOXD3,SALL4
D-myo-inositol (1,4,5,6)-Tetrakisphosphate Biosynthesis	5,79E-01	7,38E-02	NaN	PTPN6,DUSP8,EPHX2,PPFIBP2,CILP,PTPN12,PXYLP1,PTPN22,PPFIA4
D-myo-inositol (3,4,5,6)-tetrakisphosphate Biosynthesis	5,79E-01	7,38E-02	NaN	PTPN6,DUSP8,EPHX2,PPFIBP2,CILP,PTPN12,PXYLP1,PTPN22,PPFIA4
Death Receptor Signaling	5,77E-01	7,69E-02	0,378	TNFRSF21,TIPARP,SPTAN1,ACTG2,CFLAR,ARHGDIB,BCL2
Calcium Signaling	5,72E-01	7,06E-02	-0,378	RAP2A,RCAN1,GRIN1,NFAT5,MYH2,GRIN2D,CHP1,MYH3,ITPR1,RCAN2,CHRNA3,MYL7
D-myo-inositol (1,4,5)-trisphosphate Degradation	5,6E-01	1,11E-01	NaN	IMPA2,INPP5D
Role of JAK2 in Hormone-like Cytokine Signaling	5,59E-01	9,38E-02	NaN	PTPN6,GHR,SOCS2
Renin-Angiotensin Signaling	5,57E-01	7,41E-02	-1,414	FOS,PTPN6,PAK6,SHC3,PIK3CD,ITPR1,PRKCZ,NOX1
Regulation of IL-2 Expression in Activated and Anergic T Lymphocytes	5,41E-01	7,69E-02	NaN	FOS,NFAT5,CHP1,TGFB3,TGFB2,VAV1
PXR/RXR Activation	5,35E-01	7,94E-02	NaN	ABCB1,ALDH3A2,IL6,ABCC3,NR3C1
ERK5 Signaling	5,35E-01	7,94E-02	-1,000	IL6ST,FOS,RPS6KA3,MAP3K8,PRKCZ
MIF-mediated Glucocorticoid Regulation	5,35E-01	9,09E-02	NaN	PLA2G4A,PTGS2,NR3C1
Cardiomyocyte Differentiation via BMP Receptors	5,27E-01	1,05E-01	NaN	BMP4,BMP7
NAD Biosynthesis from 2-amino-3-carboxymuconate Semialdehyde	5,26E-01	1,67E-01	NaN	NMNAT3
Arginine Biosynthesis IV	5,26E-01	1,67E-01	NaN	ASS1
Urea Cycle	5,26E-01	1,67E-01	NaN	ASS1
Chondroitin and Dermatan Biosynthesis	5,26E-01	1,67E-01	NaN	CHSY3
Purine Ribonucleosides Degradation to Ribose-1-phosphate	5,26E-01	1,67E-01	NaN	PNP
Zymosterol Biosynthesis	5,26E-01	1,67E-01	NaN	TM7SF2
Glycogen Biosynthesis II (from UDP-D-Glucose)	5,26E-01	1,67E-01	NaN	GBE1

Nitric Oxide Signaling in the Cardiovascular System	5,22E-01	7,37E-02	-1,890	VEGFA,CACNA1E,CAV1,PIK3CD,ITPR1,KDR,PRKCZ
IL-9 Signaling	5,11E-01	8,82E-02	NaN	SOCS2,PIK3CD,JAK3
NRF2-mediated Oxidative Stress Response	5,03E-01	6,78E-02	-0,378	FOS,FTL,DNAJC9,MAF,HSPB8,PIK3CD,AOX1,ACTG2,TXN,PRKCZ,TXNRD1,GSTO1
Angiopoietin Signaling	5,02E-01	7,69E-02	0,000	ANGPT1,PAK6,GRB7,PIK3CD,TEK
Tryptophan Degradation III (Eukaryotic)	4,97E-01	1E-01	NaN	TDO2,HADH
Glioma Signaling	4,96E-01	7,22E-02	-0,816	PDGFA,PDGFRA,PIK3CD,PDGFD,CDKN2B,PRKCZ,PDGFRB
Cell Cycle Regulation by BTG Family Proteins	4,9E-01	8,57E-02	NaN	PPM1J,BTG2,HOXB9
Role of MAPK Signaling in the Pathogenesis of Influenza	4,72E-01	7,46E-02	NaN	CXCL10,PLA2G4A,BAX,PTGS2,BCL2
Glucose and Glucose-1-phosphate Degradation	4,71E-01	1,43E-01	NaN	RGN
Superpathway of Serine and Glycine Biosynthesis I	4,71E-01	1,43E-01	NaN	PSAT1
Inositol Pyrophosphates Biosynthesis	4,71E-01	1,43E-01	NaN	PPIP5K2
Tryptophan Degradation to 2-amino-3-carboxymuconate Semialdehyde	4,71E-01	1,43E-01	NaN	TDO2
Pregnenolone Biosynthesis	4,71E-01	1,43E-01	NaN	CYP7B1
Mineralocorticoid Biosynthesis	4,71E-01	1,43E-01	NaN	HSD3B1
autophagy	4,69E-01	8,33E-02	NaN	LAMP2,MAP1LC3A,BCL2
14-3-3-mediated Signaling	4,62E-01	6,9E-02	-0,447	FOS,TUBA1B,PLCE1,TUBB6,VIM,PIK3CD,BAX,PRKCZ
p38 MAPK Signaling	4,51E-01	6,84E-02	1,134	IL33,PLA2G4A,IL1A,IL18,RPS6KA3,TGFB3,TGFB2,IL1B
FGF Signaling	4,45E-01	7,06E-02	-0,816	PTPN6,FGF18,FGF2,FGF9,PIK3CD,ITPR1
UVB-Induced MAPK Signaling	4,44E-01	7,55E-02	-1,000	FOS,RPS6KA3,PIK3CD,PRKCZ
CXCR4 Signaling	4,39E-01	6,62E-02	-1,667	FOS,GNG11,PAK6,RHOB,MYLPF,RHOJ,PIK3CD,ITPR1,PRKCZ,MYL7
Sphingosine and Sphingosine-1-phosphate Metabolism	4,25E-01	1,25E-01	NaN	SGPP2
Histidine Degradation III	4,25E-01	1,25E-01	NaN	MTHFD2
Sphingomyelin Metabolism	4,25E-01	1,25E-01	NaN	SMPD3
CTLA4 Signaling in Cytotoxic T Lymphocytes	4,2E-01	6,9E-02	NaN	AP2M1,PTPN6,PPM1J,AP1S2,PIK3CD,PTPN22

UVA-Induced MAPK Signaling	4,2E-01	6,9E-02	-1,342	FOS,PLCE1,TIPARP,RPS6KA3,PIK3CD,SMPD3
PEDF Signaling	4,16E-01	7,04E-02	NaN	ARHGAP22,GDNF,PIK3CD,CFLAR,BCL2
Wnt/Ca+ pathway	4,13E-01	7,27E-02	0,000	NFAT5,PLCE1,FZD3,WNT5B
IL-17 Signaling	4,03E-01	6,94E-02	NaN	CXCL10,MMP3,PIK3CD,PTGS2,IL6
Systemic Lupus Erythematosus Signaling	3,99E-01	6,31E-02	NaN	IL1A,PTPN6,PRPF19,CD79A,IL6,INPP5D,IL33,SNRPN,FOS,IL18,NFAT5,IL1B,PIK3CD
Bupropion Degradation	3,95E-01	8,33E-02	NaN	CYP2J2,CYP1B1
Superpathway of D-myo-inositol (1,4,5)-trisphosphate Metabolism	3,95E-01	8,33E-02	NaN	IMPA2,INPP5D
Neuroprotective Role of THOP1 in Alzheimer's Disease	3,94E-01	7,5E-02	NaN	MME,GNRH1,MMP9
Prolactin Signaling	3,91E-01	6,85E-02	0,000	FOS,SOCS2,PIK3CD,NR3C1,PRKCZ
Dopamine-DARPP32 Feedback in cAMP Signaling	3,87E-01	6,37E-02	-2,646	GRIN1,CACNA1E,PLCE1,PPM1J,GRIN2D,PPP1R3C,KCNJ15,CHP1,ITPR1,PRKCZ
Pathogenesis of Multiple Sclerosis	3,86E-01	1,11E-01	NaN	CXCL10
UDP-N-acetyl-D-galactosamine Biosynthesis II	3,86E-01	1,11E-01	NaN	GNPDA2
fMLP Signaling in Neutrophils	3,83E-01	6,54E-02	-1,134	NFAT5,GNG11,CHP1,PIK3CD,ITPR1,PRKCZ,NOX1
Corticotropin Releasing Hormone Signaling	3,83E-01	6,54E-02	0,000	VEGFA,FOS,CRHR2,PTGS2,ITPR1,GLI1,PRKCZ
MIF Regulation of Innate Immunity	3,78E-01	7,32E-02	NaN	PLA2G4A,FOS,PTGS2
FcγRIIB Signaling in B Lymphocytes	3,78E-01	7,32E-02	NaN	CD79A,PIK3CD,INPP5D
Mechanisms of Viral Exit from Host Cells	3,78E-01	7,32E-02	NaN	SH3GL3,ACTG2,PRKCZ
IL-17A Signaling in Gastric Cells	3,74E-01	8E-02	NaN	CXCL10,FOS
Role of JAK family kinases in IL-6-type Cytokine Signaling	3,74E-01	8E-02	NaN	IL6ST,IL6
Gluconeogenesis I	3,74E-01	8E-02	NaN	FBP1,ALDOA
Natural Killer Cell Signaling	3,73E-01	6,48E-02	NaN	PTPN6,PAK6,CD244,VAV1,PIK3CD,INPP5D,PRKCZ
Myc Mediated Apoptosis Signaling	3,71E-01	6,9E-02	NaN	PIK3CD,BAX,PRKCZ,BCL2
AMPK Signaling	3,63E-01	6,21E-02	-0,378	AK5,SLC2A1,ADRA2A,EEF2,PPM1J,ADRA2C,PIK3CD,PFKP,CHRNA3,SMARCD3,ADRA1B
UVC-Induced MAPK Signaling	3,62E-01	7,14E-02	NaN	FOS,SMPD3,PRKCZ

Fcγ Receptor-mediated Phagocytosis in Macrophages and Monocytes	3,55E-01	6,45E-02	-0,816	EZR,VAV1,ACTG2,CSF2,INPP5D,PRKCZ
Antiproliferative Role of TOB in T Cell Signaling	3,54E-01	7,69E-02	NaN	TGFB3,TGFB2
D-myo-inositol (1,4,5)-Trisphosphate Biosynthesis	3,54E-01	7,69E-02	NaN	PLCE1,PIP5K1B
Acetone Degradation I (to Methylglyoxal)	3,54E-01	7,69E-02	NaN	CYP2J2,CYP1B1
Embryonic Stem Cell Differentiation into Cardiac Lineages	3,51E-01	1E-01	NaN	MESP1
NAD Phosphorylation and Dephosphorylation	3,51E-01	1E-01	NaN	PXYLP1
Glycogen Degradation II	3,51E-01	1E-01	NaN	PYGM
Histidine Degradation VI	3,51E-01	1E-01	NaN	CYP7B1
Serotonin Receptor Signaling	3,47E-01	6,98E-02	NaN	MAOB,HTR2B,IL4I1
Cyclins and Cell Cycle Regulation	3,45E-01	6,49E-02	0,447	CCND2,PPM1J,TGFB3,TGFB2,CDKN2B
Superpathway of Cholesterol Biosynthesis	3,35E-01	7,41E-02	NaN	DHCR24,TM7SF2
CCR3 Signaling in Eosinophils	3,28E-01	6,19E-02	NaN	PLA2G4A,GNG11,PPP1R12B,PAK6,PIK3CD,ITPR1,PRKCZ
CD28 Signaling in T Helper Cells	3,28E-01	6,19E-02	-0,378	FOS,PTPN6,NFAT5,CHP1,VAV1,PIK3CD,ITPR1
T Cell Receptor Signaling	3,26E-01	6,25E-02	NaN	SHB,FOS,NFAT5,PAG1,VAV1,PIK3CD
Methylglyoxal Degradation III	3,21E-01	9,09E-02	NaN	AKR1B1
IGF-1 Signaling	3,16E-01	6,19E-02	0,000	IGFBP4,FOS,IGFBP6,SOCS2,PIK3CD,PRKCZ
IL-15 Signaling	3,1E-01	6,35E-02	NaN	PIK3CD,IL6,CSF2,JAK3
Cell Cycle: G1/S Checkpoint Regulation	3,1E-01	6,35E-02	NaN	CCND2,TGFB3,TGFB2,CDKN2B
Synaptic Long Term Potentiation	3,03E-01	6,03E-02	-2,646	GRIN1,PLCE1,GRIN2D,PPP1R3C,CHP1,ITPR1,PRKCZ
Gap Junction Signaling	3,02E-01	5,96E-02	NaN	TUBA1B,HTR2B,PLCE1,TUBB6,CAV1,PIK3CD,ACTG2,ITPR1,PRKCZ
ERK/MAPK Signaling	3,02E-01	5,91E-02	0,302	ITGB1,PLA2G4A,MYCN,FOS,PAK6,PPM1J,PPP1R3C,DUSP6,ITGA2,PIK3CD,ITGA4
CD40 Signaling	2,99E-01	6,25E-02	NaN	FOS,PIK3CD,PTGS2,JAK3
Dendritic Cell Maturation	2,98E-01	5,92E-02	0,632	IL33,COL1A2,IL1A,IL18,PLCE1,FSCN1,IL1B,PIK3CD,IL6,CSF2

Hematopoiesis from Multipotent Stem Cells	2,95E-01	8,33E-02	NaN	CSF2
Glutaryl-CoA Degradation	2,95E-01	8,33E-02	NaN	HADH
Glycogen Degradation III	2,95E-01	8,33E-02	NaN	PYGM
Nicotine Degradation III	2,93E-01	6,38E-02	NaN	CYP2J2,AOX1,CYP1B1
p70S6K Signaling	2,87E-01	5,93E-02	-1,134	PLCE1,F2R,EEF2,PPM1J,CD79A,PIK3CD,PRKCZ
B Cell Receptor Signaling	2,86E-01	5,85E-02	0,000	RAP2A,PTPN6,NFAT5,DAPP1,PAG1,VAV1,MAP3K8,CD79A,PIK3CD,INPP5D
Fatty Acid β-oxidation I	2,85E-01	6,67E-02	NaN	SLC27A2,HADH
GPCR-Mediated Nutrient Sensing in Enteroendocrine Cells	2,85E-01	6,02E-02	NaN	GNG11,PLCE1,LPAR5,ITPR1,PRKCZ
Ephrin A Signaling	2,81E-01	6,25E-02	NaN	VAV1,PIK3CD,EFNA1
GABA Receptor Signaling	2,79E-01	6,06E-02	NaN	AP2M1,KCNN4,GABRB3,KCNH2
Gαi Signaling	2,72E-01	5,83E-02	-1,134	GNG11,RALA,NPR3,ADRA2A,CAV1,RGS4,ADRA2C
Fatty Acid Activation	2,71E-01	7,69E-02	NaN	SLC27A2
Chondroitin Sulfate Degradation (Metazoa)	2,71E-01	7,69E-02	NaN	CD44
Ubiquinol-10 Biosynthesis (Eukaryotic)	2,71E-01	7,69E-02	NaN	CYP7B1
CNTF Signaling	2,7E-01	6,12E-02	NaN	IL6ST,RPS6KA3,PIK3CD
Erythropoietin Signaling	2,69E-01	5,97E-02	NaN	FOS,PTPN6,PIK3CD,PRKCZ
α-Adrenergic Signaling	2,68E-01	5,88E-02	-2,000	GNG11,PYGM,ADRA2A,ITPR1,PRKCZ
iCOS-iCOSL Signaling in T Helper Cells	2,66E-01	5,83E-02	-0,447	NFAT5,CHP1,VAV1,PIK3CD,ITPR1,INPP5D
Melatonin Signaling	2,59E-01	5,88E-02	-1,000	PLCE1,RORA,GNRH1,PRKCZ
DNA Double-Strand Break Repair by Non-Homologous End Joining	2,5E-01	7,14E-02	NaN	XRCC4
Superpathway of Citrulline Metabolism	2,5E-01	7,14E-02	NaN	ASS1
Dermatan Sulfate Degradation (Metazoa)	2,5E-01	7,14E-02	NaN	CD44
Colanic Acid Building Blocks Biosynthesis	2,5E-01	7,14E-02	NaN	GMDS
Triacylglycerol Biosynthesis	2,44E-01	6,06E-02	NaN	PLPPR3,GPAM
γ-glutamyl Cycle	2,31E-01	6,67E-02	NaN	OPLAH
CDP-diacylglycerol Biosynthesis I	2,14E-01	6,25E-02	NaN	GPAM

Parkinson's Signaling	2,14E-01	6,25E-02	NaN	UCHL1
γ-linolenate Biosynthesis II (Animals)	1,99E-01	5,88E-02	NaN	SLC27A2
Mitochondrial L-carnitine Shuttle Pathway	1,99E-01	5,88E-02	NaN	SLC27A2

Supplementary Table S11. Microarray validation. A total of 17 genes, 11 induced and 6 repressed, were validated in Non-Epithelioid vs. Epithelioid group. Fold change, p-value and FDR obtained in validation are show. Normal letter: up-regulated genes; bold: down-regulated genes in microarrays.

GENE	Non-Epithelioid vs. Epithelioid		
	Fold change	p-value	FDR
GREM1	1.94	0.77	
KRT34	23.26	<0.001	0.001
CDH13	16.50	<0.001	<0.001
THBS1	14.11	<0.001	<0.001
COL6A3	11.83	<0.001	<0.001
COL13A1	6.41	0.001	0.003
FSP1	5.38	<0.001	0.001
CAV1	5.01	<0.001	<0.001
THBS2	3.22	0.001	0.003
SNAI1	2.41	0.016	0.028
CD44	1.97	0.015	0.027
**BMP4**	0.32	0.001	0.003
**KDR**	0.29	0.002	0.005
**CDH1**	0.28	0.032	0.052
**LAMA5**	0.27	0.001	0.003
**BMP7**	0.20	<0.001	0.001
**THBD**	0.19	<0.001	0.002

Supplementary Table S12. Contingency analysis. Approximately, Non-Epithelioid samples were double in high-GDPs fluids, while Epithelioid samples were slightly higher in low-GDPs fluids. Chi-square analysis was not statistically significant.

	**High-GDP**	**Low-GDP**	**Total**
**Non-Epithelioid**	30	16	46
**Epithelioid**	15	19	34
**Total**	45	35	80

p-value = 0.06

Supplementary Table S13. Baseline clinical data of patients

Parameter	Mean	SD
**Age (years)**	70.35	14.10
**Time in PD (mouths)**	11.28	8.56
**Cr-MTC (mg/ml/day)**	9.51	3.94
**Urea-MTC (mg/ml/day)**	22.98	6.00
**UF (ml/4h)**	757.11	252.69
**KT/V**	2.40	0.55
**nPCR (mg/kg/day)**	1.21	0.27
**Hb (g/dL)**	12.00	1.30
**Cr (mg/dL)**	8.00	2.78
**CCr (ml/min)**	5.77	2.59
**Urea (mg/dL)**	140.27	39.69
**Na (mEq/L)**	137.73	2.91
**K (mEq/L)**	4.29	0.55
**Albumin (g/dL)**	3.44	0.47
**Pre-Albumin (mg/dL)**	34.61	8.50
**Cause of kidney failure**	Diabetes	3
	Arterial hypertension	5
	Glomerulonephitis	3
	Tubulointerstitial nephritis	4
	Polycystic kiney disease	1
	Congenital	1
	Unknow	4
**PD tecnique**	CAPD	24
	APD	1
	OCPD	1
**Exchanges**	**Nº Exchanges**	**Patients**
	2	2
	3	10
	4	12
	5	2
**Glucose**	**High**	**Low**
	8	18
**Peritonitis**	yes: 17	no: 9
**Hemoperitoneum**	yes: 0	no: 26
**Escapes**	yes: 2	no: 24
**Sex**	**Man**	**Women**
	16	10
